# Expanding Cyanide‐Bridged Weakly Coordinating Anions Through the Brominated Silver Salt Ag[BCNB^Br^]

**DOI:** 10.1002/chem.71310

**Published:** 2026-06-27

**Authors:** Amina L. Moshtaha, Tim‐Niclas Streit, Joshua Parche, Robin Sievers, Moritz Malischewski

**Affiliations:** ^1^ Institute of Chemistry and Biochemistry – Inorganic Chemistry Freie Universität Berlin Berlin Germany

**Keywords:** boron, bromine, cyanide, fluorine, Lewis Acids, WCA

## Abstract

Weakly coordinating anions (WCAs) composed of a cyanide unit bridging two triarylborane Lewis acids are an attractive but largely unexplored complement to established tetraarylborates such as [B(C_6_F_5_)_4_]^−^ (BArF_20_). Here, we report the brominated representative [μ‐(CN)(B(C_6_F_4_Br)_3_)_2_]^−^ ([BCNB^Br^]^−^) through its readily accessible silver salt Ag[BCNB^Br^], prepared from AgCN and two equivalents of B(C_6_F_4_Br)_3_ in CH_2_Cl_2_. Ag[BCNB^Br^] provides convenient access to [BCNB^Br^]^−^ in weakly coordinating media and enables the introduction of this WCA through oxidation or halide abstraction. Its utility is demonstrated by the synthesis of a range of [BCNB^Br^]^−^ salts containing oxidizing and electrophilic cations. These results show that [BCNB^Br^]^−^ is stable under strongly oxidizing conditions, including systems reaching at least +1.14 V vs. FeCp_2_/FeCp_2_^+^. [BCNB^Br^]^−^ represents a bromine‐substituted WCA whose heavier bromine substituents enhance X‐ray scattering and facilitate crystal structure analysis of its salts.

## Graphical abstract

The brominated weakly coordinating anion [μ‐(CN)(B(C_6_F_4_Br)_3_)_2_] ([BCNB^Br^]^−^), based on two B(C_6_F_4_Br)_3_ units bridged by cyanide, is obtained as its silver salt Ag[BCNB^Br^] from CH_2_Cl_2_. Ag[BCNB^Br^] enables oxidation and halide abstraction reactions, affording diverse [BCNB^Br^]^−^ salts with oxidizing and electrophilic cations. The anion shows high oxidative stability, while bromine substituents improve x‐ray scattering and facilitate crystal structure analysis.

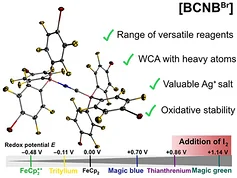

## 1 Introduction

Weakly coordinating anions (WCAs) are indispensable building blocks for accessing highly reactive cationic species and enabling their characterization. Effective WCAs combine minimal coordinating ability with high stability toward oxidizing conditions and electrophiles, typically provided by large, robust anions with delocalized charge and minimal nucleophilic sites [1].

Among the most established WCAs are halogenated tetraarylborates such as [B(C_6_F_5_)_4_]^−^ (BArF_20_) and [B(3,5‐C_6_H_3_(CF_3_)_2_)_4_]^−^ (BArF_24_) [1]. Both are widely used as counter anions in homogeneous catalysis, as supporting electrolytes in electrochemistry, and for stabilizing reactive cationic species [2, 3].

In many applications, silver salts are particularly useful reagents, since Ag^+^ enables halide abstraction and can act as a one‐electron oxidant [1]. In practice, a major limitation is that many commonly used Ag[WCA] salts are difficult to synthesize or unstable in weakly coordinating solvents [1]. Solvent‐free alkali metal salts of [BArF_24_]^−^ are synthetically [4] or even commercially available. In contrast, the corresponding Ag[BArF] salts are prepared by salt metathesis in donor solvents. Accordingly, Ag[BArF_20_] and Ag[BArF_24_] are typically isolated as solvates containing strongly coordinating donor solvents (e.g., Et_2_O) [5, 6]. Furthermore, both salts exhibit limited stability in *ortho*‐difluorobenzene (*o*DFB) and CH_2_Cl_2_, decomposing to triarylboranes BR_3_ and oligomeric silver organyls AgR [5, 6]. This has prompted the development of arene‐stabilized species, such as [Ag(*arene*)_3_][BArF_20_] and [Ag(benzene)_3_][BArF_24_], although enhanced stability in CH_2_Cl_2_ was only observed for Ag[BArF_20_] solvates [5, 7]. By contrast, some donor‐free Ag^+^ salts of WCAs are available, like Ag[Al(OC(CF_3_)_3_)_4_], which is synthesized by salt metathesis in SO_2_ [1, 8].

A less commonly used class of boron‐based WCAs features cyanide anions bridging two triarylborane units [1]. While the parent anion, [μ‐(CN)(B(C_6_H_5_)_3_)_2_]^−^ ([BCNB^H^]^−^), was first reported by Lippard in 1981, perfluorination affords the more robust anion, [μ‐(CN)(B(C_6_F_5_)_3_)_2_]^−^ ([BCNB]^−^), which is recognized as a valuable WCA due to its very low basicity and the pronounced tendency of its salts to crystallize [9, 10]. [BCNB]^−^ was established primarily through Bochmann's report of the tritylium salt in 1999. Further, a related system of the diborane(6) dianion [B_2_(CN)_6_]^2‒^ framework decorated with BCF, yielding the WCA [B_2_{CNB(C_6_F_5_)_3_}_6_]^2‒^ was recently published, illustrating the broader concept of generating WCAs through coordinating boron‐based Lewis acids to cyanide or related compounds [11]. Despite the early works of Bochmann, however, [BCNB]^−^ chemistry has remained largely unexplored since then, with no silver salt having been reported [10, 12]. We recently revisited this platform and developed a straightforward synthesis of the silver salt Ag[BCNB] from commercially available AgCN and B(C_6_F_5_)_3_ in weakly coordinating solvents (e.g., CH_2_Cl_2_ or *o*DFB), enabling direct follow‐up chemistry and providing access to “naked” Ag^+^ as evidenced by coordination of the extremely weak Lewis base Ni(CO)_4_ [13].

Despite the many advantages of highly fluorinated tetraarylborates, this class of WCAs comprises only light elements, which can provide limited x‐ray scattering and complicate scXRD characterization of salts of reactive cations. Tetraarylborates incorporating heavier halogens are rarely described and not widely used in WCA chemistry. The chlorinated borate [B(3,5‐C_6_H_3_Cl_2_)_4_]^−^ can reduce disorder compared to CF_3_‐substituted arylborates. However, only alkali metal or ammonium salts, but no silver salt, were reported [14, 15]. The perhalogenated borates [B(4‐C_6_F_4_I)_4_]^−^ and [B(4‐C_6_F_4_Br)_4_]^−^ have so far only been isolated as lithium salts from donor solvents [16, 17].

As we had recently reported the synthesis of the soft Lewis superacid B(C_6_F_4_Br)_3_ [18], we wondered whether reaction with AgCN could give Ag[μ‐(CN)(B(C_6_F_4_Br)_3_)_2_] (Ag[BCNB^Br^]), featuring a total of six *para*‐bromine substituents. If successful, the resulting silver salt would provide a convenient entry to a family of oxidizing/electrophilic ionic reagents bearing the brominated WCA, enabling access to reactive cations, while the heavy Br substituents are expected to improve x‐ray scattering and thereby facilitate scXRD characterization of the resulting salts.

## 2 Results and Discussion

### 2.1 Syntheses

We herein report the new silver salt Ag[BCNB^Br^], a *para*‐brominated analogue of the previously described perfluorinated compound Ag[BCNB] [13]. Incorporation of bromine provides a heavy‐atom variant of this WCA, and we evaluate the silver salt as a practical reagent for subsequent introduction of [BCNB^Br^]^−^. Ag[BCNB^Br^] is readily prepared from AgCN and 2.08 equivalents of the Lewis acid B(C_6_F_4_Br)_3_ (BCF^Br^) in CH_2_Cl_2_ at room temperature (Scheme 1, left). Upon stirring for several hours, the initially insoluble AgCN dissolves completely, indicating full conversion. Consequently, no filtration is required during the work‐up, and removal of the solvent followed by drying affords Ag[BCNB^Br^] as a white solid in 85% yield. Notably, traces of CH_2_Cl_2_ remain coordinated to Ag^+^, even after drying, which is reflected in the solid‐state structure.

**SCHEME 1:**
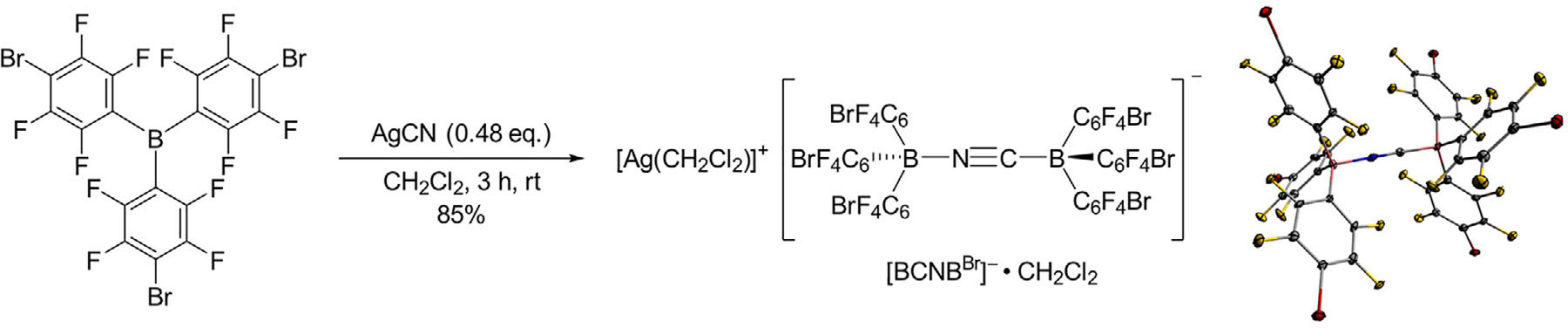
Synthesis of [Ag(CH_2_Cl_2_)][μ‐(CN)(B(C_6_F_4_Br)_3_)_2_]•CH_2_Cl_2_ from AgCN with BCF^Br^ in CH_2_Cl_2_ (left) and solid‐state structure of the [BCNB^Br^]^‒^ anion (right). Solvent molecules and Ag^+^ are omitted for clarity. Ellipsoids are depicted with 50% probability level. Color code: pink – boron, grey – carbon, yellow – fluorine, red – bromine, blue – nitrogen.

Crystallization by layering a CH_2_Cl_2_ solution with *n*‐pentane provided single crystals suitable for scXRD (Scheme 1, right). The compound was identified as the dichloromethane solvate [Ag(CH_2_Cl_2_)][μ‐(CN)(B(C_6_F_4_Br)_3_)_2_]•CH_2_Cl_2_.

Ag[BCNB^Br^] was analyzed using IR and NMR spectroscopy. The IR spectrum agrees qualitatively with the calculated wavenumbers (see Supporting Information), and displays the characteristic cyanide stretching band at *ṽ*(CN) = 2289 cm^−1^, similar to Ag[BCNB] (*ṽ*(CN) = 2290 cm^−1^) [13]. The ^19^F NMR spectrum shows two sets of signals corresponding to the carbon‐ and nitrogen‐coordinated BCF^Br^ units, respectively. The −131.6 and −132.8 ppm, while the *meta*‐fluorine atoms are high field shifted to −137.3 and −137.6 ppm. The chemical shift differences (Δδ) between the respective *meta‐* and *ortho‐*fluorine signals of the distinct cyanide‐bound BCF^Br^ units are 1.1 ppm (*ortho*‐F) and 0.3 ppm (*meta*‐F), similar to Ag[BCNB] [13]. In the ^11^B NMR spectrum, the signals of the tetracoordinated boron centers are found at −14.6 and −24.2 ppm, which is in the same range as Ag[BCNB] [13]. The broad downfield signal corresponds to the nitrogen‐bound boron. In contrast, the sharper, high field‐shifted signal corresponds to the carbon‐bound boron. In the ^13^C NMR spectrum, all signals corresponding to the BCF^Br^ Lewis acid are present, whereas the cyanide carbon signal is not observed. Synthesized with the correct stoichiometry, Ag[BCNB^Br^] is obtained cleanly and can be used without further purification. In contrast, excess AgCN promotes formation of persistent monoadducts, such as Ag[CN(B(C_6_F_4_Br)_3_)] and Ag[NC(B(C_6_F_4_Br)_3_)], which are difficult to remove. Monitoring the reaction and supplementing Lewis acid as needed is therefore recommended. Excess Lewis acid, however, typically introduces impurities due to its pronounced moisture sensitivity. Traces of water already lead to hydrolysis of B(C_6_F_4_Br)_3_ to B(C_6_F_4_Br)_3_·H_2_O, which subsequently decomposes to products including BrF_4_C_6_H, which can be observed in the ^19^F NMR spectra. Importantly, these impurities arise primarily from the Lewis acid during the synthesis, rather than hydrolysis of the [BCNB^Br^] anion. If hydrolysis products are present, the product can be dried under elevated temperature, ending up with solely traces of BrF_4_C_6_H. Alternatively, purification of Ag[BCNB^Br^] by recrystallisation using a mixture of CH_2_Cl_2_ and either toluene or benzene is effective. Notably, the aromatic solvent remains bound to Ag^+^ upon isolation and drying, yielding solvates of the type [Ag(*arene*)][BCNB^Br^]. Both solvates were analyzed by NMR spectroscopy, with the ^1^H NMR spectrum confirming the presence of coordinating benzene or toluene. The ^19^F NMR spectra of both solvates show fluorine signals and shift differences (δΔ) similar to those of the [Ag(CH_2_Cl_2_)]^+^ solvate. The IR spectrum of the toluene solvate shows the cyanide stretching band at *ṽ*(CN) = 2297 cm^−1^, which is only slightly blue‐shifted compared to the dichloromethane solvate. Single crystals of the toluene solvate [Ag(toluene)_3_][BCNB^Br^] were obtained by cooling an Ag[BCNB^Br^] solution in CH_2_Cl_2_/toluene to −70°C.

Highly electrophilic Ag^+^ salts act as strong oxidants in weakly coordinating solvents [19]. We therefore investigated the reactivity of Ag[BCNB^Br^] as a powerful oxidizer and assessed the oxidative stability of the [BCNB^Br^]^−^ anion by isolating the corresponding oxidized salts.

This was realized by treating CH_2_Cl_2_ solutions of Ag[BCNB^Br^] with equimolar amounts of different reducing agents spanning a wide potential window (Scheme 2). The products were characterized by scXRD and IR spectroscopy. In the IR spectra, all isolated salts display the bridging cyanide band around *ṽ*(CN) = 2300 cm^−1^. Mild reducing agents such as decamethylferrocene FeCp*_2_ (*E*_1/2_ = −0.48 V vs. FeCp_2_/FeCp_2_^+^) [20] and ferrocene FeCp_2_ (*E*_1/2_ = 0 V) [20] were oxidized immediately, as indicated by formation of a dark suspension (FeCp*_2_^+^) or a deep blue solution (FeCp_2_^+^), accompanied by the formation of a dark precipitate of elemental silver. Single crystals suitable for scXRD were obtained by vapor diffusion of *n*‐pentane into a solution of [FeCp*_2_][BCNB^Br^] in *o*DFB, confirming the presence of the decamethylferrocenium cation. Similarly, the much weaker reducing agent N(4‐BrC_6_H_4_)_3_ (*E*_1/2_ = +0.70 V) [20] was oxidized by Ag[BCNB^Br^], instantly forming a deep blue solution of the corresponding “magic blue” salt N(4‐BrC_6_H_4_)_3_[BCNB^Br^]. Single crystals were obtained by cooling a CH_2_Cl_2_ solution to −70°C. The obtained salt can be stored under inert conditions and used as a strong oxidizer to introduce [BCNB^Br^]^−^ as a WCA via oxidation with “magic blue”. These results demonstrate the stability of the WCA under oxidative conditions up to +0.70 V. When using the slightly weaker reducing agent thianthrene (*E*_1/2_ = +0.86 V) [20], no oxidation was observed upon addition to the Ag[BCNB^Br^] solution, as indicated by the absence of a colour change. This highlights the limited oxidizing strength of Ag[BCNB^Br^], which is typical of silver‐based oxidizers and does not reflect the oxidative stability of the [BCNB^Br^]^−^ anion. Nevertheless, it highlights constraints of the Ag^+^ oxidizing strength under slightly coordinating conditions in polar solvents. To further investigate the stability of the anion by introduction of different oxidizing cations, elemental iodine I_2_ was added to the mixture of Ag[BCNB^Br^] and thianthrene, exploiting the increased oxidizing strength of the synergistic Ag/I_2_ system [21]. Upon iodine addition, the mixture turned intense violet‐pink, and a white precipitate of AgI formed. The thianthrenium salt was isolated by filtering the mixture, removing the solvent, and washing with *n*‐pentane to remove excess iodine. Single crystals were obtained by vapor diffusion of *n*‐pentane into a CH_2_Cl_2_ solution of [C_6_H_4_S_2_C_6_H_4_][BCNB^Br^].

**SCHEME 2:**
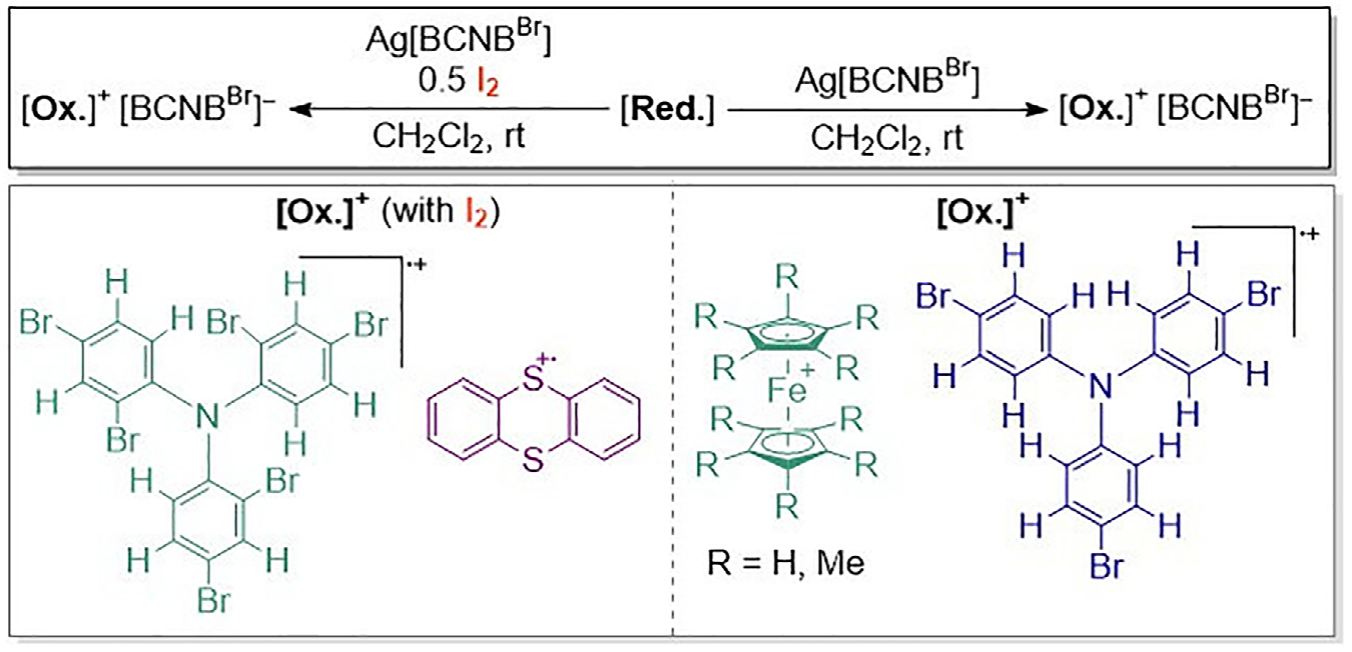
Oxidation of reducing agents [Red.] with Ag[BCNB^Br^] (right) and for weaker reducing agents with Ag[BCNB^Br^] and I_2_ (left), yielding the salts [Ox.][BCNB^Br^]. The oxidized cations are depicted below.

In this study, the weakest reducing agent that could be oxidized by Ag[BCNB^Br^] in combination with I_2_ was N(2,4‐Br_2_C_6_H_3_)_3_ (*E*_1/2_ = +1.14 V) [20]. As with thianthrene, oxidation (and the corresponding visible colour change to a dark green solution) occurred only upon the addition of iodine. Following an analogous work‐up, IR spectroscopy was used to analyze the “magic green” salt, and single crystals were obtained by vapor diffusion of *n*‐pentane into a CH_2_Cl_2_ solution. Overall, the oxidation reactions demonstrate an oxidizing strength of at least +0.70 V for Ag[BCNB^Br^], which can be increased to at least +1.14 V when combined with I_2_. The isolation of various [BCNB^Br^]^‒^ salts proves that the [BCNB^Br^]^−^ anion is stable under oxidative conditions up to at least +1.14 V (Figure 1). The scope of the reactions performed highlights Ag[BCNB^Br^] as a useful reagent for generating reactive cations while introducing the stable WCA [BCNB^Br^]^−^. Furthermore, the work‐up is easily performed by filtration as the only byproducts are insoluble elemental silver or silver iodide.

**FIGURE 1:**
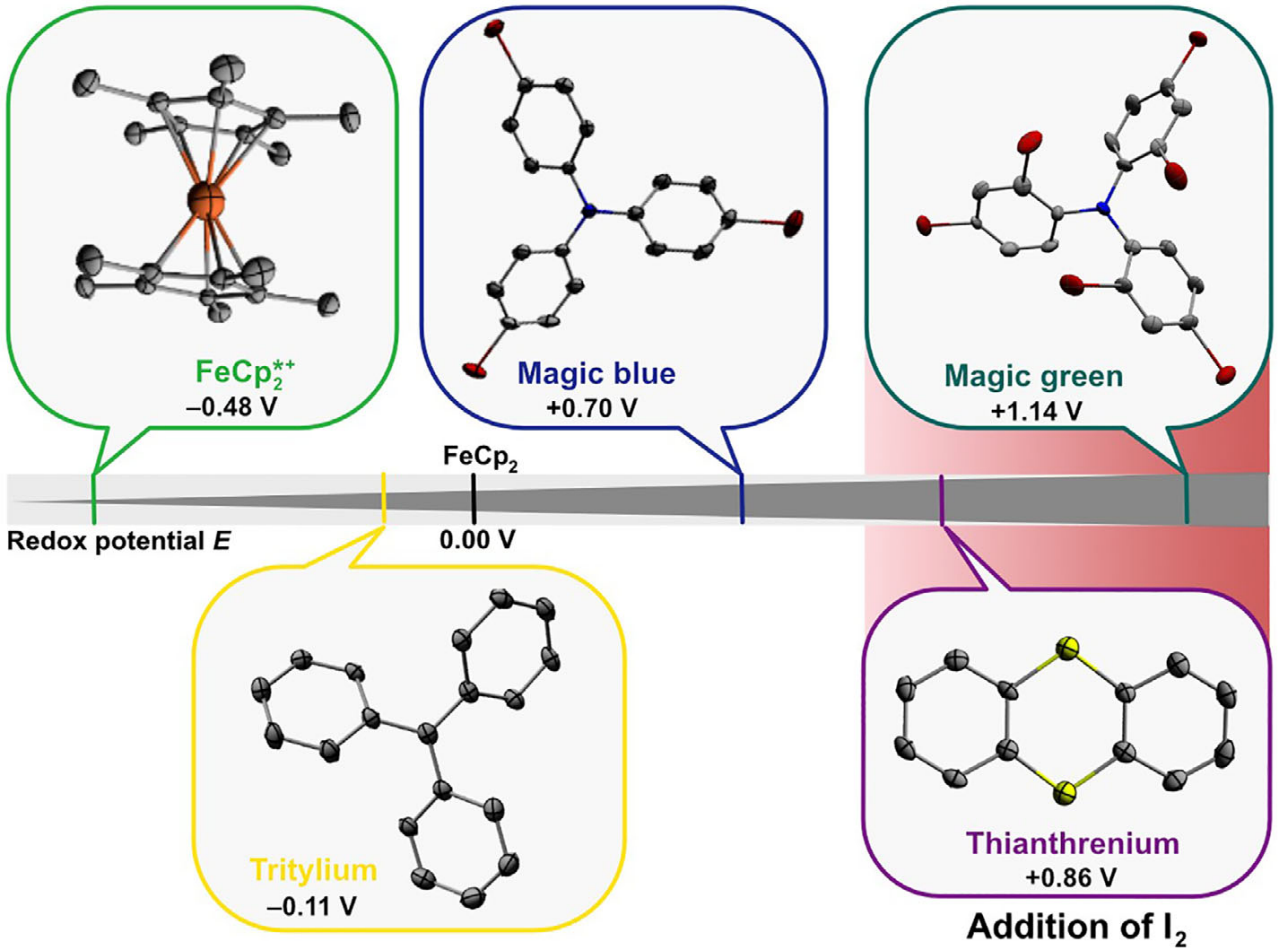
Redox potentials of the oxidized cations, illustrating the oxidizing strength of Ag[BCNB^Br^] and oxidative stability of the WCA. At increased potentials above +0.86 V, oxidation is only possible with Ag[BCNB^Br^] in combination with I_2_.

Since tritylium salts offer a high solubility and are useful reagents for halide‐ or hydride‐abstractions, it was targeted to obtain the corresponding [BCNB^Br^]^−^ salt, analogous to the literature known Ph_3_C[BCNB], published by Bochmann et al. [10]. The synthesis of Ph_3_C[BCNB^Br^] was realized in two steps: After the silver salt was generated from AgCN and B(C_6_F_4_Br)_3_ in CH_2_Cl_2_, trityl chloride was added to the reaction mixture, leading to the formation of an intense yellow solution and precipitation of AgCl (Scheme 3, top). Pure Ph_3_C[BCNB^Br^] was obtained in 66% yield by concentrating the filtered reaction mixture, layering it with *n*‐pentane, and allowing diffusion at −24°C.

**SCHEME 3:**
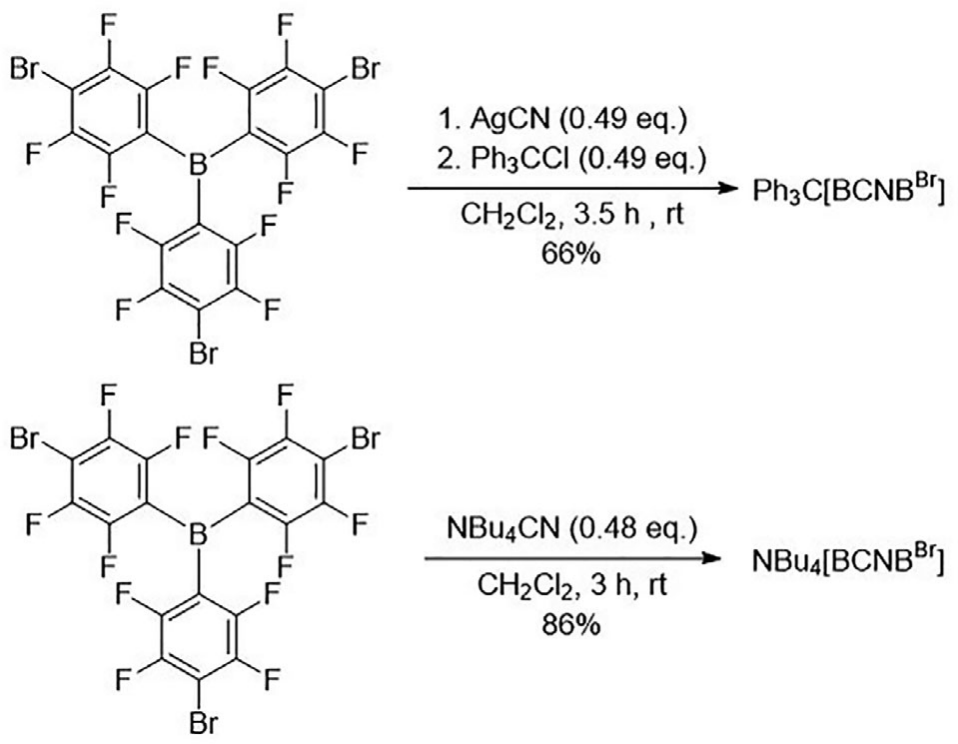
Synthesis of Ph_3_C[BCNB^Br^] and NBu_4_[BCNB^Br^].

Hence, purification of the tritylium salt proved more facile than that of the silver salt from CH_2_Cl_2_. IR and NMR analyses were performed, and the anion data are consistent with those of the other synthesized salts. The bridging cyanide band at *ṽ*(CN) = 2303 cm^‒1^ is almost identical to that of the corresponding [BCNB]^‒^ salt (*ṽ*(CN) = 2305 cm^−1^) [10]. The presence of the tritylium cation was confirmed by ^1^H and ^13^C NMR spectroscopy, which showed all the characteristic tritylium signals [11]. Single crystals of Ph_3_C[BCNB^Br^] were obtained by layering a highly concentrated CH_2_Cl_2_ solution with *n*‐pentane and cooling to −24°C.

Finally, to access a redox‐inactive and light‐insensitive counter‐cation with high solubility, we synthesized the *n*‐tetrabutylammonium salt NBu_4_[BCNB^Br^] by stirring NBu_4_CN with 2.08 equivalents of B(C_6_F_4_Br)_3_ in CH_2_Cl_2_ for 3 h (Scheme 3, bottom). The product was obtained as a beige solid in 86% yield. Similar to the tritylium salt, the NBu_4_^+^ salt is easily purified by recrystallization from CH_2_Cl_2_ layered with *n*‐pentane. The product was analyzed by IR and NMR spectroscopy, and the ^1^H NMR spectrum confirmed the introduction of the NBu_4_^+^ cation. The [BCNB^Br^]^−^ signals in the ^19^F and ^11^B NMR spectra resemble those of the other salts; however, the boron signals are slightly downfield‐shifted by 0.5 (B─N) and 0.3 (B─C) ppm compared to [Ag(CH_2_Cl_2_)][BCNB^Br^]. Single crystals of NBu_4_[BCNB^Br^] suitable for scXRD were obtained from a solution in *o*DCB by vapor diffusion with *n*‐pentane.

Overall, the isolated [BCNB^Br^]^−^ salts (NBu_4_^+^, ferrocenium, Ag^+^, tritylium, “magic blue”, thianthrenium, and “magic green”) constitute a versatile and readily accessible toolkit for introducing [BCNB^Br^]^−^ via salt metathesis, oxidation, and halide/hydride abstraction.

### 2.2 Crystal Structures

Across all isolated salts and solvates, the [BCNB^Br^]^−^ anion exhibits highly consistent bond lengths, and the bond lengths of the bridging cyanide unit are similar to those of the perfluorinated analogue [BCNB]^−^ [13]. However, the linearity of the BCNB unit and the C─B─B─C torsion angles of the BCF^Br^ unit differ for the crystallized salts. In [Ag(CH_2_Cl_2_)][μ‐(CN)(B(C_6_F_4_Br)_3_)_2_]•CH_2_Cl_2_, the two bridged BCF^Br^ units adopt a staggered arrangement, analogous to the fluorinated [Ag(*o*DFB)_3_][BCNB], with torsion angles of approximately −60° in both structures [13]. The “magic blue” and “magic green” salts display intermediate torsion angles around −30°, whereas the remaining [BCNB^Br^]^−^ salts are best described as eclipsed, spanning angles from −18.1° to 7.7°, with the minimum torsion angle found in the thianthrenium salt. These results indicate substantial conformational flexibility of the molecular framework, reflecting adaptability to solid‐state packing with counter cations, alongside pronounced rigidity of the C_6_F_5_ groups compared to the more readily disordered CF_3_ groups found, for example, in perfluoroalkoxyaluminates. The silver salt Ag[BCNB^Br^] was crystallized as a dichloromethane solvate [Ag(CH_2_Cl_2_)][μ‐(CN)(B(C_6_F_4_Br)_3_)_2_]•CH_2_Cl_2_, which crystallized in the space group *P1*, and as a solvate of stronger coordinating toluene [Ag(toluene)_3_][BCNB^Br^], which crystallized in the space group *P*2_1_/*n* (Figures 2 and 3). In the CH_2_Cl_2_‐solvate, the silver cation is hexacoordinated by six halogen atoms, four of which represent weak anion‐cation contacts (Figure 2, right). Interactions between cation and anion are limited to the three Ag‒Br contacts (2.712(1) to 2.803(2) Å) and one Ag‒F (2.603(6) Å) interaction. One CH_2_Cl_2_ molecule coordinates via both chlorine atoms as a chelating‐ligand to the cation, forming two Ag‒Cl contacts (2.857(4) and 3.017(4) Å). The second CH_2_Cl_2_ molecule interacts with the [BCNB^Br^]^‒^ anion via Cl‒F and Cl‒Br contacts. Each [BCNB^Br^]^−^ unit coordinates four distinct Ag^+^ cations via four of the six C_6_F_4_Br‐groups. These Ag‒anion interactions lead to a polymeric network in the solid state. Further intermolecular interactions are observed between the [BCNB^Br^]^−^ units via F‒F, Br‒F, and Br‒Br contacts.

**FIGURE 2:**
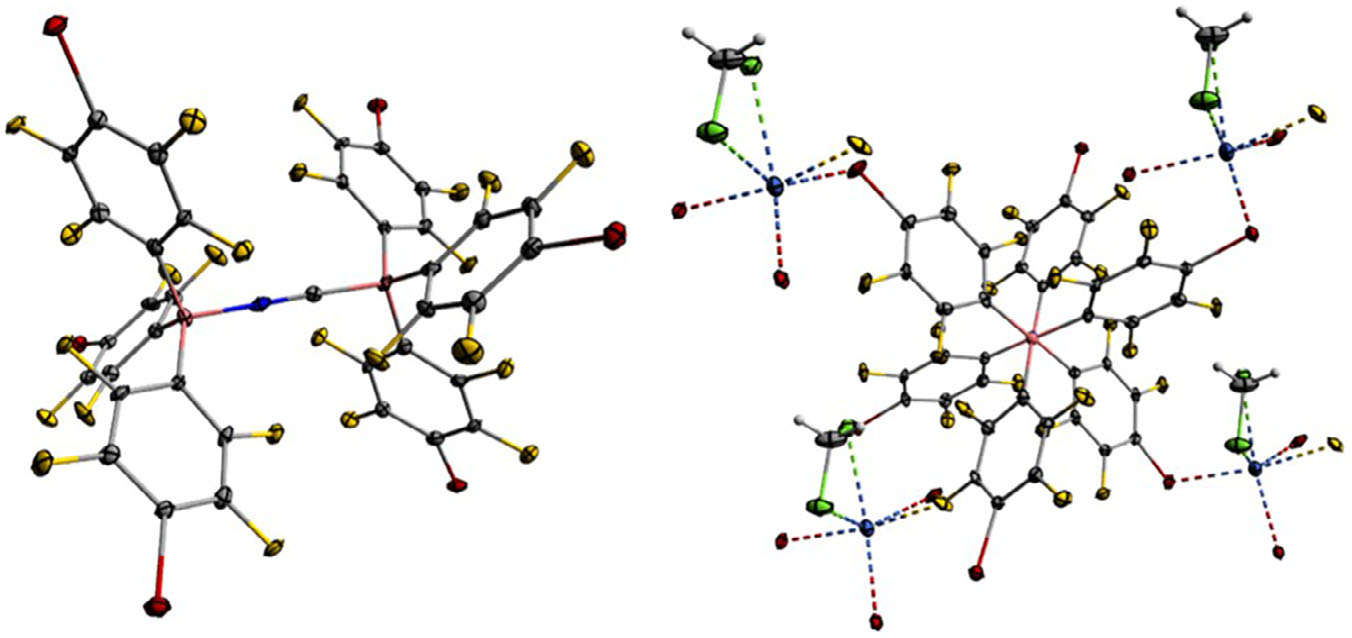
Molecular structure of [Ag(CH_2_Cl_2_)][μ‐(CN)(B(C_6_F_4_Br)_3_)_2_]•CH_2_Cl_2_ in the solid state. The [BCNB^Br^]^−^ anion structure is shown (left, CH_2_Cl_2_ and Ag^+^ are omitted for clarity), as well as the full structure with halogen‐coordinated Ag^+^ cations and co‐crystallized CH_2_Cl_2_ (right). Ellipsoids are depicted with 50% probability level. Color code: pink – boron, grey – carbon, white ‒ hydrogen, green ‒ chlorine, yellow – fluorine, red – bromine, blue ‒ nitrogen, bright blue ‒ silver.

**FIGURE 3:**
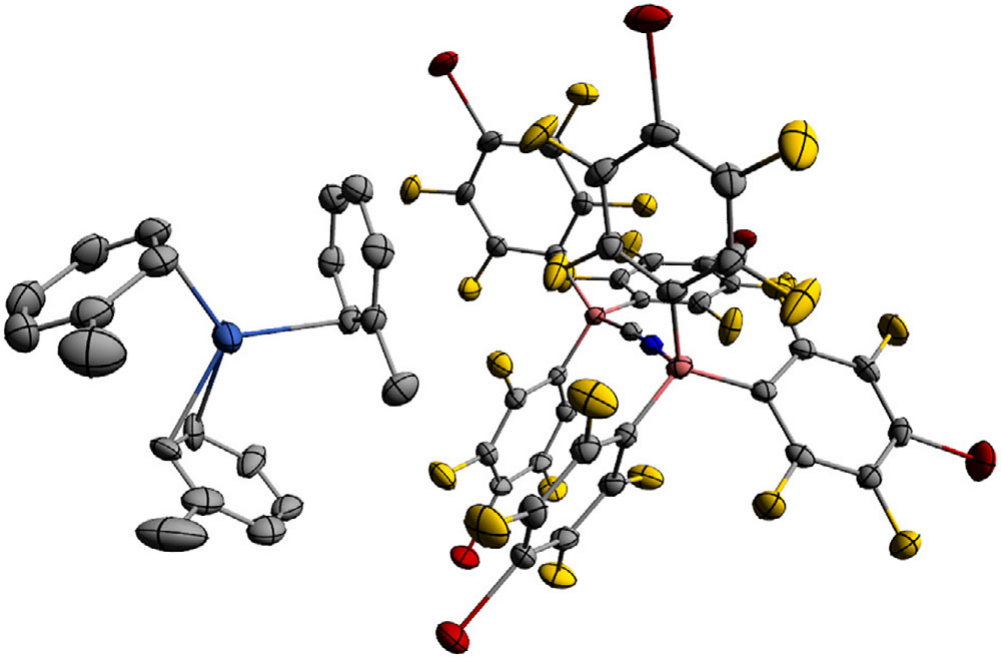
Molecular structure of [Ag(toluene)_3_][BCNB^Br^] in the solid state. Disorder and hydrogen atoms are omitted for clarity. Ellipsoids are depicted with 50% probability level. Color code: pink – boron, grey – carbon, yellow – fluorine, red – bromine, blue ‒ nitrogen, bright blue ‒ silver.

In the toluene‐solvate, the silver cation is coordinated in η^4^‐fashion by three toluene molecules, yielding in total 12 Ag─C contacts with *d*(Ag‒C) smaller than the sum of the van der Waals (vdW) radii (Figure 3). The Ag─C bond lengths, as well as the Ag–plane and Ag─centroid distances, are similar on average to those in other toluene solvates of Ag[WCA] salts, such as [BArF_20_]^−^ [6]. In this solvate, the Ag^+^ cation is solely coordinated by the toluene molecules, and interactions between the cationic [Ag(toluene)_3_]^+^ unit and the anion are limited to the secondary coordination sphere, involving C─F, H─F, C─C, and H─C contacts between toluene and [BCNB^Br^]^−^. This contrasts with the CH_2_Cl_2_ solvate, where the silver cation is not only coordinated by one solvent molecule (as a chelating ligand), but exhibits additional cation‐anion interactions (Ag‒F and Ag‒Br).

The analogous perfluorinated salt Ag[BCNB] has so far only been crystallized as a solvate of the weakly coordinating solvent *o*DFB, where the silver cation is coordinated by three *o*DFB molecules in an η^4^‐fashion [13]. This compound exhibits, similar to [Ag(toluene)_3_][BCNB^Br^], a total of 12 Ag─C contacts between the aromatic solvent and the cation. The corresponding bond lengths and Ag‒arene planes/centroid distances are also similar. However, in [Ag(*o*DFB)_3_][BCNB], the silver cation further interacts with the [BCNB]^−^ anion via two additional Ag‒F contacts, which leads to a polymeric chain structure in the solid state, similar to the CH_2_Cl_2_ solvate of Ag[BCNB^Br^]. Comparing both polymeric structures [Ag(*o*DFB)_3_][BCNB] and [Ag(CH_2_Cl_2_)_2_][BCNB^Br^], the latter exhibits additional Ag─Br contacts, yielding not a chain structure, but a more complex polymeric network. In this context, the additional cation‐anion interactions (three Ag─Br and one Ag─F) are best explained by the preference of the soft Lewis acid Ag^+^ for heavier halogen atoms. Comparing the arene solvates of both silver salts, a possible explanation for the lack of direct Ag‒anion contacts in the toluene solvate is the increased donor strength of toluene compared to CH_2_Cl_2_ or *o*DFB. In the toluene solvate, no polymeric chain or network is formed through Ag─X (X = F, Br) contacts between cation and anion, in contrast to the other silver salts discussed above. Instead, the [BCNB^Br^]^−^ anions are interacting with each other through Br‒F, F‒F, C‒F, Br‒C, and Br‒Br contacts, while the cationic [Ag(toluene)_3_]^+^ fragment predominantly resides in the voids created by this anion framework, not exhibiting any direct Ag‒anion contacts. A striking feature of [Ag(toluene)_3_][BCNB^Br^] is a directional aggregation of the *para*‐bromine atoms into distorted octahedral (Br)_6_ motifs (see SI). Within these aggregates, only two Br─Br distances (3.446 and 3.634 Å) fall below the sum of the vdW radii (ΣR_vdW_ = 3.7 Å), while the remaining bromine separations are mostly in the range from 3.7 to 4.0 Å.

Two distinct distorted (Br)_6_ octahedra occur, each formed by aggregation of three bromine atoms. Both distinct octahedra are stacked alternately to form a channel composed of discrete, noncrosslinked (Br)_6_ units. Although the individual (Br)_6_ aggregates are not interconnected, this channel resembles the intermolecular Br‒Br contacts previously reported for the free Lewis acid BCF^Br^, that were described as a “bromine tunnel” [18].

These bromine‐aggregation motifs are a recurring feature in several of the [BCNB^Br^]^−^ solid‐state structures, and similar bromine octahedral arrangements are observed in the FeCp*_2_^+^ and in the “magic green” salt. The decamethylferrocenium salt crystallized in the space group C2/c, with an Fe‒Cp* distance of *d*(Fe‒C_5,plane_) = 1.71 Å observed in the cation. In the solid state, the Cp* ligand is oriented towards the B(Ar)_3_‐unit, with several H‒F and C‒F contacts between the cation and anion. The cations and anions are stacked alternately, and between these alternating chains, the *para*‐bromine atoms point towards each other, building a distorted octahedral motif (Figure 4). Within this (Br)_6_ octahedron, three Br‒Br distances (3.561, 3.633, and 3.671 Å) are below the sum of the vdW radii, while the others are slightly longer (d(Br‒Br) < 4.0 Å). The described bromine octahedra are not interconnected.

**FIGURE 4:**
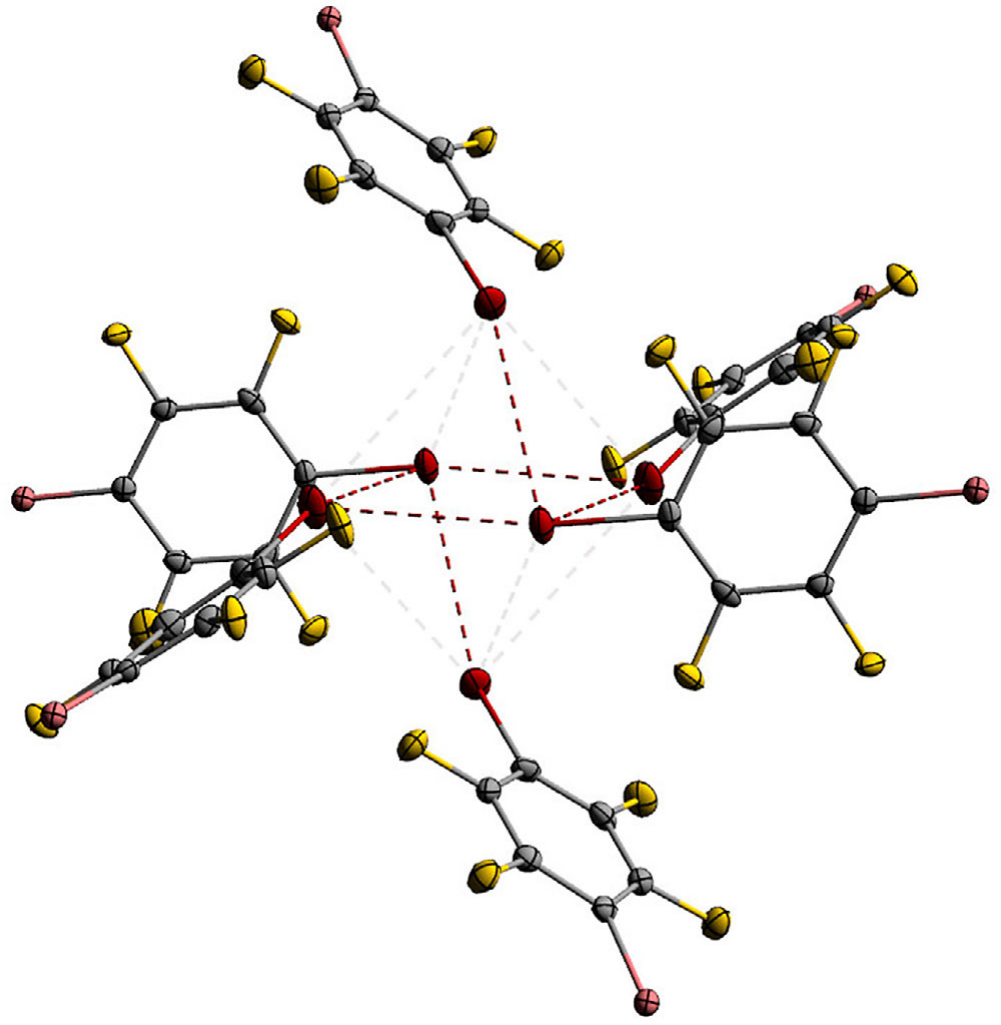
Bromine aggregation in the solid‐state structure of [FeCp*_2_][BCNB^Br^]. Intermolecular Br‒Br contacts below the sum of the van der Waals radii (ΣR_vdW_ = 3.7 Å) are depicted in red dashed lines; all longer distances are pale red. Ellipsoids are depicted with 50% probability level. Color code: pink – boron, grey – carbon, yellow – fluorine, red – bromine.

For N(2,4‐Br_2_C_6_H_3_)_3_[BCNB^Br^], which crystallizes in the space group *P* $\bar{1}$, a slightly different kind of bromine aggregation was found. While interactions between the “magic green” cation and [BCNB^Br^]^‒^ via contacts below the sum of the vdW radii are limited to Br‒F, H‒F and C‒F, an aggregation of the *para*‐bromine atoms of the cation with those of the anion is visible. Three *para*‐bromine atoms from different cation molecules are arranged in one layer. Three bromine atoms from the [BCNB^Br^]^‒^ anion form the subsequent layer, which is rotated by 60°. The arrangement of the six bromine atoms resembles a distorted octahedron, and the only Br‒Br contacts below the sum of the vdW radii found in this motif are formed between the cationic *para*‐bromine atoms (3.683 and 3.634 Å). While classification as weak interactions is debatable for the longer distances, this bromine aggregation clearly forms a repeating motif in the packing of the BCF^Br^ Lewis acid [18] and [BCNB^Br^]^−^ salts. This suggests that *para*‐bromination introduces additional, potentially structure‐directing packing preferences relative to Ag[BCNB]. Such interactions may be beneficial for crystallizing reactive cations when other intermolecular forces are weak and poorly directional, which is typical of WCA salts. In the remaining structures, no pronounced bromine aggregation motifs are observed; instead, salts with large, planar organic cations adopt more conventional alternating cation–anion stacks, as found for the tritylium and thianthrenium salts.

The tritylium salt crystallizes in the space group *P* $\bar{1}$, similarly to the corresponding [BCNB]^−^ salt. The geometrical parameters are similar to those reported for tritylium[BCNB] by Bochmann et al. [10]. In the solid state, alternating cation‐anion stacking is observed. These cation‐anion chains are formed through numerous contacts (C‒C, H‒F, and C‒F). Neighbouring chains are linked via halogen‒halogen contacts between the anions and further by cation‐cation interactions. The overall packing seems similar to that of Ph_3_C[BCNB] [10].

The thianthrenium[BCNB^Br^] salt crystallizes in the space group *P* $\bar{1}$, and the expected planarity of the thianthrenium radical cation is clearly observed [22]. Similar to the tritylium salt, an alternating cation–anion stacking arrangement is found in the crystal structure. As observed in some other thianthrenium salts, the cations in the present structure form pairs; however, rather than adopting a directly cofacial arrangement, they are slipped with respect to each other. As a result, only two short C‒C contacts (3.377 Å) are present, while no significant S‒S contacts are observed (d(S‒S) > 6.643 Å). The planar thianthrenium unit is located between two B(Ar)_3_ units, one approximately centered and the other slightly offset. Cation‐anion interaction involves S‒F, H‒F, H‒Br, and C‒F contacts.

## 3 Conclusion

We report a convenient synthesis for the silver salt Ag[BCNB^Br^], which incorporates the novel, weakly coordinating anion [BCNB^Br^]^−^. Ag[BCNB^Br^] is readily prepared in the weakly coordinating solvent CH_2_Cl_2_, and solvates with more strongly coordinating solvents were isolated, such as [Ag(toluene)_3_][BCNB^Br^]. Redox experiments demonstrate the effective oxidation strength of Ag[BCNB^Br^], and the stability of the anion under oxidative conditions up to at least +1.14 V (vs. FeCp_2_/FeCp_2_^+^). A series of oxidizing salts [*Ox*.][BCNB^Br^] ([*Ox*.] = FeCp_2_^+^, FeCp*_2_^+^, Ph_3_C^+^, “magic blue”, thianthrenium, “magic green”) was isolated, covering redox potentials from −0.48 V to +1.14 V. In addition, the redox‐inactive and highly soluble salt NBu_4_[BCNB^Br^] was synthesized. The straightforward preparation and stability in weakly coordinating solvents make Ag[BCNB^Br^] a valuable reagent, and the isolated series of [BCNB^Br^]^‒^ salts provides a toolbox for generating reactive cations through salt metathesis, oxidation, or halide/hydride abstraction. Incorporation of heavy bromine atoms improves diffraction quality, yielding crystals that are more readily measured by scXRD than those of the perfluorinated [BCNB]^‒^ analogue. Moreover, the facile crystallization of [BCNB^Br^]^−^ salts [23] and the frequent occurrence of bromine aggregation motifs suggest that bromination not only facilitates structural analysis but may also promote crystallization through additional, structure‐directing non‐covalent interactions.

## Conflicts of Interest

The authors declare no conflicts of interest.

## Supporting information




**Supporting File 1**: The authors have cited additional references within the Supporting Information [24, 25, 26, 27, 28, 29, 30, 31, 32, 33, 34, 35, 36, 37].

**Supporting File 2**: chem71310‐sup‐0002‐cif.zip

## Data Availability

The data that support the findings of this study are available in the Supporting Information of this article.

## References

[1] I. M. Riddlestone , A. Kraft , J. Schaefer , and I. Krossing , “Taming the Cationic Beast:Novel Developments in the Synthesis and Application of Weakly Coordinating Anions,” Angewandte Chemie International Edition 57 (2018): 13982–14024. 10.1002/anie.201710782 29266644

[2] I. Krossing and I. Raabe , “Noncoordinating Anions—Fact or Fiction? A Survey of Likely Candidates,” Angewandte Chemie International Edition 43 (2004): 2066–2090. 10.1002/anie.200300620 15083452

[3] E. Y. X. Chen and S. J. Lancaster , “Weakly Coordinating Anions: Highly Fluorinated Borates,” in Comprehensive Inorganic Chemistry II, ed. K. Poeppelmeier , 2nd ed . (Elsevier, 2013), 707–754, 10.1016/B978-0-08-097774-4.00127-3.

[4] A. J. Martínez‐Martínez and A. S. Weller , “Solvent‐Free Anhydrous Li^+^, Na^+^ and K^+^ Salts of [B(3,5‐(CF_3_)_2_C_6_H_3_)_4_]‒, [BAr^F^ _4_]‒. Improved Synthesis and Solid‐State Structures,” Dalton Transactions 48 (2019): 3551–3554. 10.1039/c9dt00235a 30762068

[5] A. McSkimming , “On ‘Ag[BAr^F^ _4_]’ and an Expedient Synthesis of [Ag(C_6_H_6_)_3_][BAr^F^ _4_],” Organometallics 44 (2025): 1630–1632.

[6] M. Kuprat , M. Lehmann , A. Schulz , and A. Villinger , “Synthesis of Pentafluorophenyl Silver by Means of Lewis Acid Catalysis: Structure of Silver Solvent Complexes,” Organometallics 29 (2010): 1421–1427.

[7] M. F. Ibad , A. Schulz , and A. Villinger , “Facile Route to Silver Triarene Borate Salts, [Ag(arene)_3_][B(C_6_F_5_)_4_]: Thermodynamics, Structure, and Bonding,” Organometallics 38 (2019): 1445–1458.

[8] I. Krossing , “The Facile Preparation of Weakly Coordinating Anions: Structure and Characterisation of Silverpolyfluoroalkoxyaluminates AgAl(ORF)_4_, Calculation of the Alkoxide Ion Affinity,” Chemistry ‒ A European Journal 7 (2001): 490–502. 10.1002/1521-3765(20010119)7:2<490::aid-chem490>3.0.co;2-i 11271536

[9] C. M. Giandomenico , J. C. Dewan , and S. J. Lippard , “Synthesis, Structure, and Reactions of [Mo(SnCl_3_)(CN‐*t*‐Bu)_6_][(Ph_3_B)_2_CN], Containing a Potentially Useful New Anion for Crystallizations,” Journal of the American Chemical Society 103 (1981): 1407–1412.

[10] S. J. Lancaster , D. A. Walker , M. Thornton‐Pett , and M. Bochmann , “New Weakly Coordinating Counter Anions for High Activity Polymerisation Catalysts: [(C_6_F_5_)_3_B‐CN‐B(C_6_F_5_)_3_]‒ and [Ni{CNB(C_6_F_5_)_3_}_4_]_2_‒,” *Chemical Communications* (1999): 1533–1534.

[11] L. Zapf and M. Finze , “Lewis Acid Decorated Hexacyanodiborane(6) Dianion,” Angewandte Chemie International Edition 63 (2024): e202401681. 10.1002/anie.202401681 38530744

[12] J. Zhou , S. J. Lancaster , D. A. Walker , S. Beck , M. Thornton‐Pett , and M. Bochmann , “Synthesis, Structures, and Reactivity of Weakly Coordinating Anions With Delocalized Borate Structure: The Assessment of Anion Effects in Metallocene Polymerization Catalysts,” Journal of the American Chemical Society 123 (2001): 223–237. 10.1021/ja002820h 11456508

[13] W. R. Berg , A. L. Moshtaha , R. Sievers , et al., “A Facile Route to ‘Naked’ Ag^+^ Ions Enabling the Coordination of the Weak Lewis Base Ni(CO)_4_ ,” Chemical Science 17 (2026): 406–411. 10.1039/d5sc05589j PMC12613210 41245758

[14] R. Anulewicz‐Ostrowska , T. Kliś , D. Krajewski , B. Lewandowski , and J. Serwatowski , “Synthesis of some Halogenated Tetraarylborates,” Tetrahedron Letters 44 (2003): 7329–7331.

[15] A. B. Chaplin and A. S. Weller , “B(3,5‐C_6_H_3_Cl_2_)_4_]‒ as a Useful Anion for Organometallic Chemistry,” European Journal of Inorganic Chemistry 2010 (2010): 5124–5128.

[16] S. Fischer , J. Schmidt , P. Strauch , and A. Thomas , “An Anionic Microporous Polymer Network Prepared by the Polymerization of Weakly Coordinating Anions,” Angewandte Chemie International Edition 52 (2013): 12174–12178. 10.1002/anie.201303045 24151252

[17] Y. Okhassova , R. Sroka , N. Chaoui , et al., “Weakly Coordinating Anionic Microporous Polymer Networks as Scaffold for Molecular Gas Phase Hydrogenation Catalysis,” Chemcatchem 17 (2025): e202401595.

[18] A. L. Moshtaha , E. M. Peter , R. Sievers , T. N. Streit , and M. Malischewski , “(4‐bromo‐2,3,5,6‐tetrafluorophenyl)borane B(C_6_F_4_Br)_3_ via C‒Ag to C‒B Transmetalation,” Chemistry ‒ A European Journal 31 (2025): e02240. 10.1002/chem.202502240 PMC12559468 40977095

[19] C. Armbruster , M. Sellin , M. Seiler , et al., “Pushing Redox Potentials to Highly Positive Values Using Inert Fluorobenzenes and Weakly Coordinating Anions,” Nature Communications 15 (2024): 6721. 10.1038/s41467-024-50669-3 PMC11306567 39112470

[20] N. G. Connelly and W. E. Geiger , “Chemical Redox Agents for Organometallic Chemistry,” Chemical Reviews 96 (1996): 877–910. 10.1021/cr940053x 11848774

[21] P. J. Malinowski , D. Himmel , and I. Krossing , “Coordination Chemistry of Diiodine and Implications for the Oxidation Capacity of the Synergistic Ag^+^/X_2_ (X = Cl, Br, I) System,” Angewandte Chemie International Edition 55 (2016): 9262–9266. 10.1002/anie.201603913 27411163

[22] H. Bock , A. Rauschenbach , C. Näther , M. Kleine , and Z. Havlas , “Thianthren‐Radikalkation Tetrachloroaluminat,” Chemische Berichte 127 (1994): 2043–2049.

[23] Deposition Numbers 2539283 (for [Ag(CH_2_Cl_2_)_2_][BCNB^Br^]), 2539284 (for [Ag(toluene)_3_][BCNB^Br^]), 2539285 (for NBu_4_[BCNB^Br^]), 2539286 (for Ph_3_C[BCNB^Br^]), 2539287 (for FeCp*_2_[BCNB^Br^]), 2539288 (for N(4‐BrC_6_H_4_)_3_[BCNB^Br^]), 2539289 (for [C_6_H_4_S_2_C_6_H_4_][BCNB^Br^]) and 2539290 (for N(2,4‐Br_2_C_6_H_3_)_3_[BCNB^Br^]) Contain the Supplementary Crystallographic Data for This Paper. These Data are Provided Free of Charge by the Joint Cambridge Crystallographic Data Centre and Fachinformationszentrum Karlsruhe Access Structures Service.

[24] G. R. Fulmer , A. J. M. Miller , N. H. Sherden , et al., “NMR Chemical Shifts of Trace Impurities: Common Laboratory Solvents, Organics, and Gases in Deuterated Solvents Relevant to the Organometallic Chemist,” Organometallics 29 (2010): 2176–2179.

[25] R. K. Harris , E. D. Becker , S. M. Cabral de Menezes , P. Granger , R. E. Hoffman , and K. W. Zilm , “Further Conventions for NMR Shielding and Chemical Shifts (IUPAC Recommendations 2008),” Pure and Applied Chemistry 80 (2008): 59–84.

[26] MestReNova 14.3.0‐30573 (Mestrelab Research SL, 2022).

[27] OriginPro, Version (OriginLab Corporation, 2024).

[28] O. V. Dolomanov , L. J. Bourhis , R. J. Gildea , J. A. K. Howard , and H. Puschmann , “Olex2: A Complete Structure Solution, Refinement and Analysis Program,” Journal of Applied Crystallography 42 (2009): 339–341.

[29] G. M. Sheldrick , “SHELXT – Integrated Space‐group and Crystal Structure Determination,” Acta Crystallographica A 71 (2015): 3–8. 10.1107/S2053273314026370 PMC4283466 25537383

[30] G. M. Sheldrick , SHELXL Version 2014/7, Program for Crystal Structure Solution and Refinement (University of Göttingen, 2014).

[31] G. M. Sheldrick , “A Short History of SHELX,” Acta Crystallographica A 64 (2008): 112–122. 10.1107/S0108767307043930 18156677

[32] K. Brandenburg , Diamond: Crystal and Molecular Structure Visualization (Crystal Impact GbR, 2025), http://www.crystalimpact.com/diamond.

[33] Persistence of Vision Pty. Ltd ., Persistence of Vision Raytracer. Ltd. (Persistence of Vision Pty, 2004).

[34] M. J. Frisch , G. W. Trucks , H. B. Schlegel , et al., Gaussian 16, Revision C.01 (Gaussian, Inc., 2016).

[35] M. D. Hanwell , D. E. Curtis , D. C. Lonie , T. Vandermeersch , E. Zurek , and G. R. Hutchison , “Avogadro: An Advanced Semantic Chemical Editor, Visualization, and Analysis Platform,” Journal of Cheminformatics 4 (2012): 17. 10.1186/1758-2946-4-17 PMC3542060 22889332

[36] G. A. Zhurko , ChemCraft, http://www.chemcraftprog.com.

[37] M. K. Manoj , B. Brauer , and J. M. L. Martin , “Frequency and Zero‐Point Vibrational Energy Scale Factors for Double‐Hybrid Density Functionals (and Other Selected Methods): Can Anharmonic Force Fields Be Avoided?,” Journal of Physical Chemistry A 119 (2015): 1701–1714. 10.1021/jp508422u 25296165

