# Supplementary material for: Expanding Cyanide‐Bridged Weakly Coordinating Anions Through the Brominated Silver Salt Ag[BCNBBr]
Source: Chemistry. 2026 Jun 27;32(38):e71310. doi: 10.1002/chem.71310 (PMC13660483; doi:10.1002/chem.71310)
Supplement: Supplementary file 1 [file CHEM-32-e71310-s002.pdf]

Supporting Information

Expanding Cyanide-Bridged Weakly Coordinating Anions through the Brominated Silver Salt Ag[BCNB^{Br}]

Amina L. Moshtaha,[a] Tim-Niclas Streit,[a] Joshua Parche,[a] Robin Sievers,[a] and Moritz Malischewski*[a]

[a] A. L. Moshtaha, T.-N. Streit, J. Parche, Dr. R. Sievers, Dr. M. Malischewski
Institute of Chemistry and Biochemistry – Inorganic Chemistry
Freie Universität Berlin
Fabeckstr. 34/36 14195, Berlin, Germany
E-mail: moritz.malischewski@fu-berlin.de

Content

General Information 2

Synthetic procedures 4

NMR spectra 13

IR Spectra 30

Quantum Chemical Calculations 35

Crystallographic Data 39

References 57

General Information

All reactions and workups were performed in previously heated glassware under an atmosphere of argon using standard Schlenk techniques and an oil pump vacuum of 10^{-3} mbar. Room temperature (rt) refers to 20 °C. The addition of liquid reagents and solvents was done by using threefold argon-flushed disposable syringes and septa, while solids were added in a glovebox with an atmosphere of argon. Glassware was cleaned by storing in a potassium hydroxide bath for several days, rinsed with diluted hydrochloric acid and doubly deionized water and dried at 150 °C.

Solvents and reagents

Anhydrous CH_2Cl_2 and *n*-pentane were obtained from the solvent system FMBRAUN MB SPS-800 and stored over activated 3 Å mol sieves. Anhydrous toluene and benzene were stored over activated 3 Å mol sieves prior use. Deuterated solvents CD_2Cl_2 and CD_3CN were used as purchased and stored over activated 3 Å mol sieves. $B(C_6F_4Br)_3$ was synthesized according to the literature procedure and stored in a –30 °C freezer in a glovebox.[18] AgCN and NBu_4CN were purchased and stored in a glovebox. Attention: Cyanides are highly toxic and should only be handled with great care and appropriate safety equipment. All other solvents and commercially available reagents were used without further purification. The silver, magic blue, and magic green salts of [μ-(CN)($B(C_6F_4Br)_3)_2$] were stored under exclusion of light and mostly in the glovebox freezer. All other compounds were stored under inert conditions at room temperature.

Nuclear magnetic resonance (NMR) spectroscopy

^{1}H, ^{13}C, ^{19}F, and ^{11}B NMR spectra were recorded on a JEOL ECX 400 (400 MHz) spectrometer by using 6 mm Young NMR glass tubes in deuterated solvents CD_2Cl_2 and CD_3CN. All given chemical shifts in ^{1}H NMR spectra are calibrated on the resonance signals of $CDHCl_2$ contained in CD_2Cl_2 (δ = 5.32 ppm) or CD_2HCN contained in CD_3CN (δ = 1.94 ppm). [24] The ^{13}C NMR spectra are calibrated on the respective resonance signals of CD_2Cl_2 (δ = 53.84 ppm).[24] The ^{19}F and ^{11}B spectra are device-internally calibrated relative to the resonance signal of $CFCl_3$ and $BF_3 \cdot OEt_2$ according to the unified chemical shift scale.[25] All chemical shifts (δ) are given in parts per million (ppm), the coupling constants J in Hz and the signals are specified according to the multiplicity (s = singlet, d = doublet, t = triplet, q = quartet, m = multiplet, br = broad). The program MestReNova Version 14.3.0 was used to evaluate and plot the data.[26]

Infrared (IR) spectroscopy

Infrared spectra were measured on a Bruker ALPHA FT-IR spectrometer inside a glovebox equipped with a diamond ATR attachment. The software Origin 2024 was used to plot the data.[27]

X-Ray diffraction (XRD)

X-Ray data was collected on a BRUKER D8 Venture system. The measurements were performed between 100(2) and 150(2) K (see crystallographic data) using graphite-monochromated Mo K_α (λ_α = 0.71073 Å) radiation. The strategy for the data collection was evaluated by using the Smart software. The data were collected by the standard "ψ-ω scan techniques" and were scaled and reduced using Saint+software. The structures were solved by using Olex2[28] with the XT[29] structure solution program using Intrinsic Phasing and refined with the XL refinement package[30,31] using Least Squares minimization. Bond lengths and angles were measured with Diamond Crystal and Molecular Structure Visualization Version 5.0.2. [32] Drawings were generated with POV-Ray. [33] The crystal structures discussed in the manuscript have been reported as CSD Communications (deposition numbers CCDC 2539283-2539290). These data are provided free of charge by the joint Cambridge Crystallographic Data Centre and Fachinformationszentrum Karlsruhe Access Structures service www.ccdc.cam.ac.uk/structures.

Density functional theory (DFT) calculations

DFT calculations were performed with Gaussian 16.[34] Structure optimizations and frequency calculations were done using *B3LYP-D3BJ*/def2-SVP. Solutions were processed using Avogadro[35] and Chemcraft 1.8.[36] Calculated frequencies were multiplied with the scaling factor 0.9671.[37]

Synthetic procedures

Ag[BCNB^{Br}]

General considerations

All reactions with Ag[BCNB^{Br}] *were conducted under exclusion of light.*

Achieving the correct stoichiometry in the synthesis of Ag[BCNB^{Br}] is sometimes difficult on a small-scale, and deviations will be visible in the product's NMR spectra. Using too much AgCN results in the formation of the monoadducts [CN–B($C_6F_4Br)_3$]$^-$ and [NC–B($C_6F_4Br)_3$]$^-$. The corresponding ^{19}F and ^{11}B NMR signals are shown in Figures S7 and S8, respectively. (The nitrogen-bound boron signal of [CN–B($C_6F_4Br)_3$]$^-$ is difficult to discern in the ^{11}B NMR spectrum, due to the broad signals.)

Using too little AgCN results in the presence of free B($C_6F_4Br)_3$ which could be removed by washing with *n*-pentane. However, in practice, this does not always improve the purity, because other impurities may emerge. First, when working with small amounts of the crude product, washing with *n*-pentane sometimes dissolves and removes some B($C_6F_4Br)_3$, yielding traces of the monoadducts. Second, traces of water lead to the formation of B($C_6F_4Br)_3$•H_2O from the highly moisture-senstitive Lewis acid B($C_6F_4Br)_3$, which subsequently degrades into BrF_4C_6H, $(BrF_4C_6)_2BOH$ and $(BrF_4C_6)_2BOB(C_6F_4Br)_2$. This yields multiple impurities that are visible in the NMR spectra (Figure S7). The protonated degradation product, BrC_6F_4H, is therefore almost always visible in the ^{19}F NMR spectra of [Ag(CH_2Cl_2)][BCNB^{Br}] because it is not very volatile and remains in the product when washing is avoided. Importantly, these hydrolysis-derived impurities originate exclusively from decomposition of the Lewis acid precursor during synthesis or handling under trace moisture and are not formed from decomposition of the isolated anion under normal inert conditions. While standard air- and moisture-free techniques are still required for handling, the purified anion itself does not show comparable rapid or pervasive hydrolytic degradation. So far, the presence of BrC_6F_4H has not disturbed any of the conducted reactions and can be more easily removed by washing after subsequent reactions.

Complete conversion can generally be ensured by reaction control *via* ^{19}F NMR spectroscopy. Therefore, a small volume of the reaction solution is diluted with additional CH_2Cl_2. If too little AgCN was used and monoadducts are visible during reaction control, additional B($C_6F_4Br)_3$ can easily be added. Therefore, it is recommended not to use a large excess of the Lewis acid but rather to adjust the amount of B($C_6F_4Br)_3$ after reaction control.

The reaction solvent, CH_2Cl_2, remains coordinated to the silver salt even after work-up and drying. This can be seen in the NMR spectra of [Ag(CH_2Cl_2)][BCNB^{Br}] measured in CD_3CN and referenced with *o*DFB (Figure S9 and S10). It has to be mentioned, that the product seems to degrade partially upon dissolving in CD_3CN or other coordinating solvents, but was

nevertheless used to prove the presence of CH_2Cl_2. The amount of coordinated solvent varies by batch, but it is approximately 0.4 molecules of CH_2Cl_2 per silver atom.

It was investigated whether drying could improve the purity of the crude product with $B(C_6F_4Br)_3{\bullet}H_2O$, BrF_4C_6H, $(BrF_4C_6)_2BOH$ and $(BrF_4C_6)_2BOB(C_6F_4Br)_2$ present as impurities due to their higher volatility. While drying for 18 h at room temperature was not enough to evaporate the impurities, drying at an elevated temperature of 50 °C for 2 h enabled the evaporation or degradation of $B(C_6F_4Br)_3{\bullet}H_2O$, $(BrF_4C_6)_2BOH$ and $(BrF_4C_6)_2BOB(C_6F_4Br)_2$, while more protonated BrF_4C_6H was formed which is potentially easier to remove in subsequent steps (Figure S11). The amount of coordinated CH_2Cl_2 is decreased after drying, but approximately 0.1 molecules of CH_2Cl_2 are still coordinating to the silver. These results are in contrast to Ag[BCNB], the analogue with the lighter, perfluorinated Lewis acid BCF, where drying is not improving the purity, but leads to degradation of the anion [BCNB^{Br}] to the monoadducts due to higher volatility of BCF.

Table S1. Infrared and NMR spectroscopic data of several [BCNB^{Br}]$^-$ salts. NMR samples were measured in CD_2Cl_2.

	**Infrared**	**NMR**				
	$\tilde{\nu}$(CN)	^{19}F [δ in ppm]			^{11}B [δ in ppm]	
	[cm^{-1}]	*ortho*-F	*meta*-F	$\Delta\delta_{meta}$	B-N	B-C
[Ag(CH_2Cl_2)][BCNB][12]	2290	–133.42<br>–134.54	–165.54<br>–165.86		–12.8	–22.6
Tritylium[BCNB][11]	2305	–133.29<br>–134.40	–165.14<br>–165.47		–11.9	–21.7
Theoretical [BCNB^{Br}]$^{a)}$	2353					
Cation[BCNB^{Br}]						
[Ag(CH_2Cl_2)]$^+$	2289	–131.64<br>–132.78	–137.27<br>–137.57		–14.6	–24.2
[Ag(toluene)]$^+$	2297	–131.70<br>–132.84	–137.24<br>–137.53		–12.2	–21.6
[Ag(benzene)]$^+$	-	–131.62<br>–132.75	–137.28<br>–137.56		–12.1	–21.8
NBu_4^+	2308	–131.62<br>–132.76	–137.33<br>–137.62		–14.1	–23.9
Tritylium	2303	–131.61<br>–132.74	–137.25<br>–137.54		–14.3	–23.9
$FeCp_2^+$	2285					
$FeCp^*{}_2^+$	2309					
Magic Blue	2286					
Thianthrenium	2286					
Magic Green	2285					

a) measured in $CDCl_3$

[Ag(CH$_2$Cl$_2$)][BCNB^{Br}]

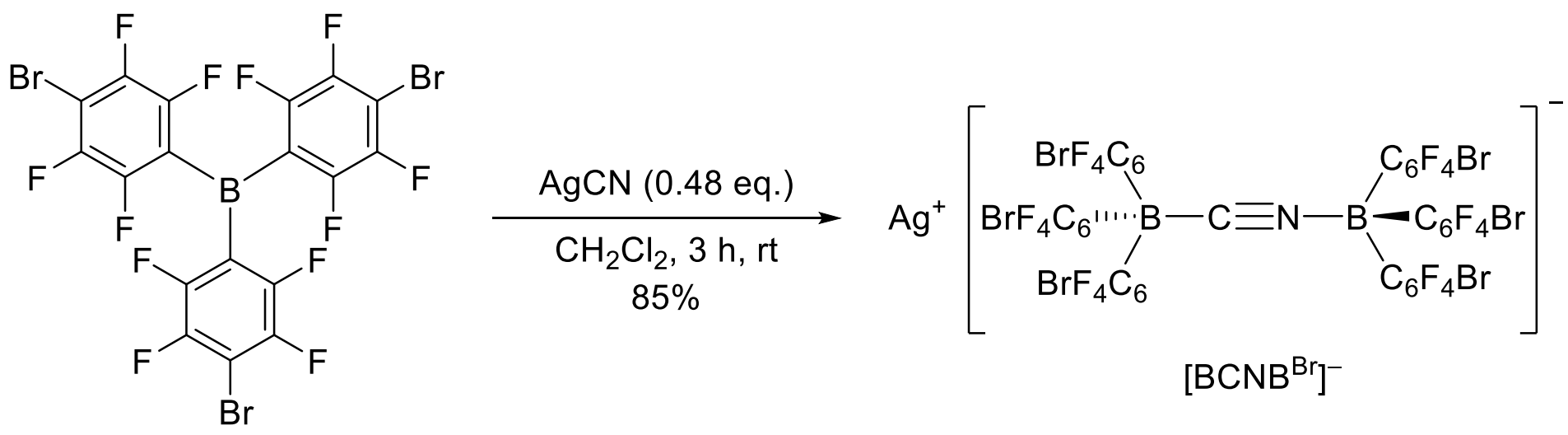

B(C$_6$F$_4$Br)$_3$ (194 mg, 0.279 mmol, 1.0 eq.) and AgCN (18.0 mg, 0.134 mmol, 0.48 eq.) were placed in a Schlenk tube, CH$_2$Cl$_2$ (4 mL) was added and the mixture was stirred at room temperature. After forming a clear solution, the reaction progress was monitored by ^{19}F and ^{11}B NMR analysis of 0.1 mL of the reaction solution diluted with additional CH$_2$Cl$_2$. After stirring for a total of 3 h, the solvent was removed under reduced pressure and the product was dried in vacuum for 30 min. [Ag(CH$_2$Cl$_2$)][BCNB^{Br}] (173 mg, 0.114 mmol, 85%) was obtained as a colourless solid.

Single crystals for scXRD analysis of [Ag(CH$_2$Cl$_2$)][µ-(CN)(B(C$_6$F$_4$Br)$_3$)$_2$]•CH$_2$Cl$_2$ were obtained by layering a CH$_2$Cl$_2$ solution with *n*-pentane at 4 °C.

^{19}F NMR (CD$_2$Cl$_2$, rt, 375.79 MHz): δ = –131.61 - –131.67 (m, 6 F, *ortho*-F), –132.76 - –132.80 (m, 6 F, *ortho*-F), –137.22 - –137.33 (m, 6 F, *meta*-F), –137.51 - –137.62 (m, 6 F, *meta*-F) ppm.

^{13}C{^{19}F} NMR (CD$_2$Cl$_2$, rt, 100.42 MHz): δ = 148.6 (*ortho*-C$_6$F$_4$Br), 144.8 (*meta*-C$_6$F$_4$Br), 122.7 (**C**–B), 97.6 (**C**–Br) ppm.

^{11}B NMR (CD$_2$Cl$_2$, rt, 128.15 MHz): δ = –14.6 (broad, B–N), –24.2 (B–C) ppm.

FT-IR (ATR) $\tilde{\nu}$ = 2289 (vw), 1626 (m), 1588 (vw), 1439 (vs), 1393 (s), 1377 (s), 1294 (w), 1241 (vs), 1116 (m), 1012 (m), 948 (vs), 927 (vs), 873 (s), 822 (s), 775(s), 748 (vs), 733 (s), 668 (vs), 605 (m), 503 (m), 486 (m), 429 (m) cm^{-1}.

IR (calculated) $\tilde{\nu}$ = 2353, 1613, 1466, 1242, 955, 937, 822, 740, 713, 655 cm^{-1}.

[Ag(toluene)][BCNB^{Br}]

Ag[BCNB^{Br}] (30 mg) was purified by recrystallization from a 2:1 mixture of toluene (3 mL) and CH_2Cl_2 (1.5 mL), which was slowly cooled to –70 °C. After isolating the colorless crystals and drying in vacuum for several hours, the toluene remains in the product, coordinating the silver. Using the same method, single crystals of [Ag(toluene)$_3$][BCNB^{Br}] were obtained for scXRD analysis.

^{19}F NMR (CD_2Cl_2, rt, 375.79 MHz): δ = –131.66 - –131.72 (m, 6 F, *ortho*-F), –132.82 - –132.86 (m, 6 F, *ortho*-F), –137.19 - –137.29 (m, 6 F, *meta*-F), –137.49 - –137.58 (m, 6 F, *meta*-F) ppm.

^{1}H NMR (CD_2Cl_2, rt, 400 MHz): δ = 7.32 – 7.17 (m, 3 H, $C_6\mathbf{H}_5CH_3$), 2.37 (s, 3 H, $C_6H_5C\mathbf{H_3}$) ppm.

^{11}B NMR (CD_2Cl_2, rt, 128.15 MHz): δ = –12.2 (broad, B–N), –21.6 (B–C) ppm.

FT-IR (ATR) $\tilde{\nu}$ = 2928 (vw), 2297 (w), 1627 (w), 1590 (w), 1489 (w), 1439 (vs), 1393 (m), 1382 (m), 1243 (s), 1081 (w), 1015 (w), 946 (s), 927 (s), 868 (m), 836 (m), 777 (w), 746 (s), 698 (w), 670 (s), 605 (w), 474 (m) cm^{-1}.

[Ag(benzene)][BCNB^{Br}]

Ag[BCNB^{Br}] (30 mg) was purified by recrystallization from a 3:1 mixture of CH_2Cl_2 (3 mL) and benzene (1 mL), which was slowly cooled to –24 °C. After isolating the colourless precipitate and drying in vacuum for several hours, the benzene remained in the product, coordinating to the silver.

^{19}F NMR (CD_2Cl_2, rt, 375.79 MHz): δ = –131.59 - –131.65 (m, 6 F, *ortho*-F), –132.73 - –132.78 (m, 6 F, *ortho*-F), –137.23 - –137.33 (m, 6 F, *meta*-F), –137.51 - –137.62 (m, 6 F, *meta*-F) ppm.

^{1}H NMR (CD_2Cl_2, rt, 400 MHz): δ = 7.44 (s, 6 H, C_6H_6) ppm.

^{11}B NMR (CD_2Cl_2, rt, 128.15 MHz): δ = –12.1 (broad, B–N), –21.8 (B–C) ppm.

$NBu_4[BCNB^{Br}]$

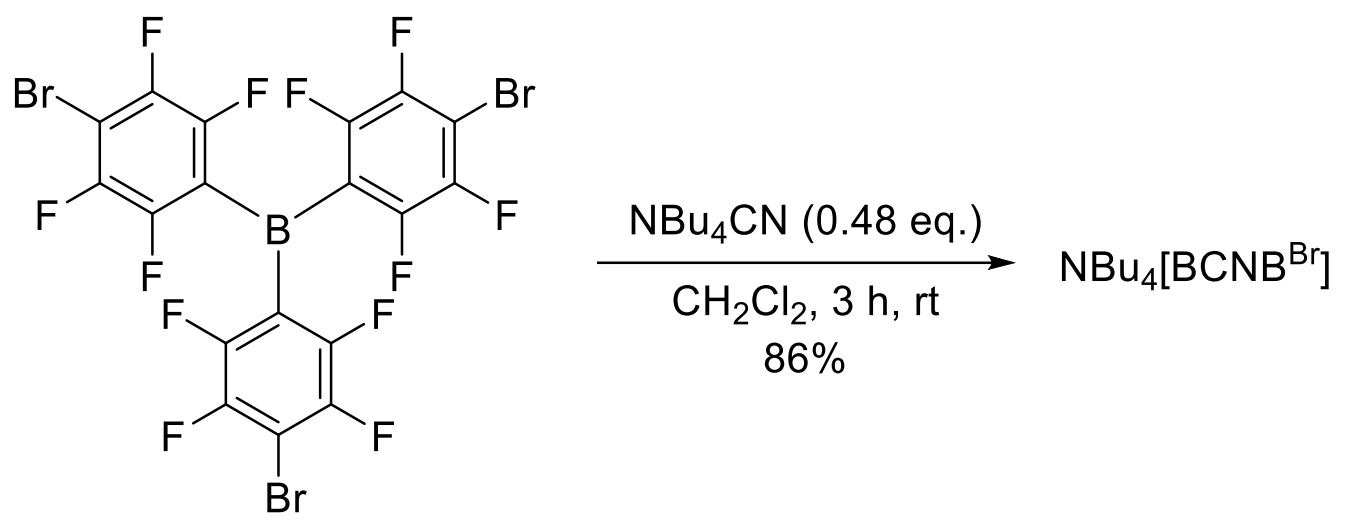

$B(C_6F_4Br)_3$ (200 mg, 0.288 mmol, 1.0 eq.) and NBu_4CN (37 mg, 0.138 mmol, 0.48 eq.) were placed in a Schlenk tube, CH_2Cl_2 (10 mL) was added and the mixture was stirred at room temperature. After forming a clear solution, the reaction progress was monitored by ^{19}F and ^{11}B NMR analysis of 0.1 mL of the reaction solution diluted with additional CH_2Cl_2. After stirring for a total of 3 h, the solvent was removed under reduced pressure, and the product was dried in vacuum for 30 min. Crude $NBu_4[BCNB^{Br}]$ (196 mg, 0.118 mmol, 86%) was obtained as a yellowish solid.

To increase the product's purity, a solution of $NBu_4[BCNB^{Br}]$ (approximately 50 mg) in CH_2Cl_2 (6 mL) was layered with *n*-pentane (6 mL). The solution was left at room temperature to allow diffusion, then cooled to –24 °C in a freezer. The purified salt (20 mg) was obtained as colorless crystals. Recrystallization by vapor diffusion or with a higher salt concentration in CH_2Cl_2 yields more product but could still contain traces of impurities.

Single crystals for scXRD analysis of $NBu_4[BCNB^{Br}]$ were obtained by vapor diffusion of *n*-pentane into an *o*DCB solution.

^{19}F NMR (CD_2Cl_2, rt, 375.79 MHz): δ = –131.59 - –131.65 (m, 6 F, *ortho*-F), –132.73 - –132.79 (m, 6 F, *ortho*-F), –137.28 - –137.39 (m, 6 F, *meta*-F), –137.57 - –137.68 (m, 6 F, *meta*-F) ppm.

$^{13}C\{^{19}F,^{1}H\}$ NMR (CD_2Cl_2, rt, 100.42 MHz): δ = 148.6 (*ortho*-C_6F_4Br), 144.8 (*meta*-C_6F_4Br), 122.6 (**C**–B), 97.6 (**C**–Br), 59.4 ($N(\mathbf{CH_2}CH_2CH_2CH_3)_4$), 24.1 ($N(CH_2\mathbf{CH_2}CH_2CH_3)_4$), 20.0 ($N(CH_2CH_2\mathbf{CH_2}CH_3)_4$), 13.6 ($N(CH_2CH_2CH_2\mathbf{CH_3})_4$) ppm.

^{11}B NMR (CD_2Cl_2, rt, 128.15 MHz): δ = –14.1 (broad, B–N), –23.9 (B–C) ppm.

^{1}H NMR (CD_2Cl_2, rt, 400 MHz): δ = 3.07 - 3.03 (m, 2 H, $N(\mathbf{CH_2}CH_2CH_2CH_3)_4$), 1.63 - 1.55 (m, 2 H, $N(CH_2\mathbf{CH_2}CH_2CH_3)_4$), 1.45 - 1.36 (m, 2 H, $N(CH_2CH_2\mathbf{CH_2}CH_3)_4$), 1.01 (t, 3 H, $N(CH_2CH_2CH_2\mathbf{CH_3})_4$) ppm.

FT-IR (ATR) $\tilde{\nu}$ = 2967 (w), 2938 (w), 2879 (w), 2308 (w), 1627 (w), 1583 (vw), 1440 (vs), 1393 (m), 1377 (m), 1309 (w), 1237 (s), 1165 (w), 1111 (w), 1063 (w), 1001 (m), 946 (s), 930 (s), 881 (m), 836 (m), 793 (w), 776 (m), 747 (s), 671 (s), 600 (m), 501 (w), 488 (w), 429 (w), 405 (w) cm^{-1}.

$Ph_3C[BCNB^{Br}]$

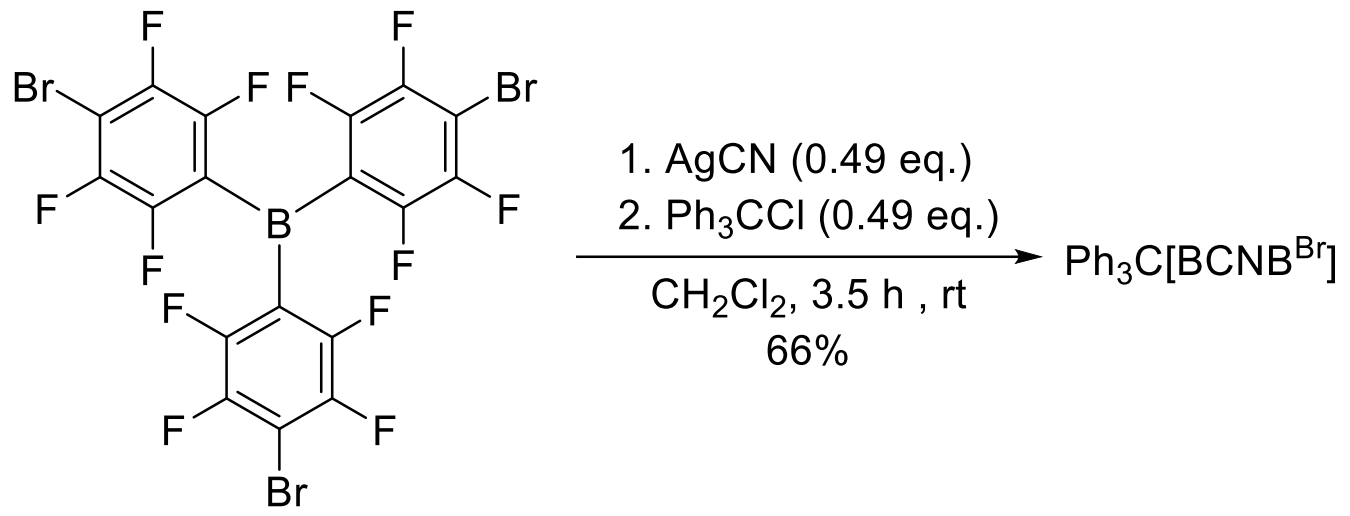

$B(C_6F_4Br)_3$ (200 mg, 0.288 mmol, 1.0 eq.) and AgCN (19.0 mg, 0.142 mmol, 0.49 eq.) were placed in a Schlenk tube, CH_2Cl_2 (10 mL) was added and the mixture was stirred at room temperature. After forming a clear solution, the reaction progress was monitored by ^{19}F and ^{11}B NMR analysis of 0.5 mL of the reaction solution diluted with additional CH_2Cl_2. After stirring for 3 h in total, Ph_3CCl (39.5 mg, 0.142 mmol, 0.49 eq.) was added to the reaction mixture, leading directly to an intense yellow solution and the formation of white a precipitate. After stirring for an additional 30 min, the solution was filtered through a syringe filter. To recrystallize the product, the solution was concentrated under reduced pressure (approximately 7 mL) and layered with *n*-pentane (30 mL). The Schlenk tube was placed in a –24 °C freezer for several days, yielding needle-shaped, yellow crystals that were isolated and dried under reduced pressure. $Ph_3C[BCNB^{Br}]$ (156 mg, 0.094 mmol, 66%) was obtained as a yellow solid.

Single crystals of $Ph_3C[BCNB^{Br}]$ for scXRD analysis were obtained similarly, by layering a more diluted solution in CH_2Cl_2 with *n*-pentane at –24 °C.

^{19}F NMR (CD_2Cl_2, rt, 375.79 MHz): δ = –131.57 - –131.66 (m, 6 F, *ortho*-F), –132.71 - –132.77 (m, 6 F, *ortho*-F), –137.20 - –137.31 (m, 6 F, *meta*-F), –137.49 - –137.60 (m, 6 F, *meta*-F) ppm.

^{13}C NMR{^1H,^{19}F} (CD_2Cl_2, rt, 100.42 MHz): δ = 211.2 ($(H_5C_6)_3$**C^+**), 148.6 (*ortho*-C_6F_4Br), 144.7 (*meta*-C_6F_4Br), 144.0 (*p*-CH), 143.1 (*o*-CH), 140.3 (*ipso*-C), 131.0 (*m*-CH), 122.7 (**C**–B), 97.6 (**C**–Br) ppm.

^{11}B NMR (CD_2Cl_2, rt, 128.15 MHz): δ = –14.3 (broad, B–N), –23.9 (B–C) ppm.

^1H NMR (CD_2Cl_2, rt, 400 MHz): δ = 8.28 – 8.23 (m, 1H, *para*-CH), 7.89 – 7.84 (m, 2 H, *m*-CH), 7.67 – 7.64 (m, 2H, *o*-CH) ppm.

FT-IR (ATR) $\tilde{\nu}$ = 2303 (w), 1625 (w), 1582 (m), 1483 (w), 1444 (vs), 1392 (m), 1375 (w), 1355 (s), 1294 (m), 1240 (m), 1186 (w), 1165 (w), 1115 (w), 996 (w), 949 (s), 930 (s), 878 (m), 836 (m), 806 (w), 767 (w), 749 (s), 733 (w), 703 (s), 672 (s), 623 (w), 607 (m), 467 (w) cm^{-1}.

Oxidation reactions with Ag[BCNB^{Br}]

$FeCp_2$[BCNB^{Br}]

$$\mathrm{Ag[BCNB^{Br}]} \xrightarrow[\text{85\%}]{\mathrm{FeCp_2}\atop \mathrm{CH_2Cl_2},\ \text{15 min, rt}} \mathrm{FeCp_2[BCNB^{Br}]}$$

Ag[BCNB^{Br}] (90 mg, 0.059 mmol, 1.0 eq.) was placed in a Schlenk tube and dissolved in CH_2Cl_2 (5 mL). $FeCp_2$ (11 mg, 0.059 mmol, 1.0 eq.) was added, forming instantly a deep blue solution, which was then stirred for approximately 15 min. The reaction mixture was filtered through a syringe filter, and the solvent was evaporated under reduced pressure. $FeCp_2$[BCNB^{Br}] (80 mg, 0.050 mmol, 85%) was obtained as a blue solid.

FT-IR (ATR) $\tilde{\nu}$ = 3116 (w), 2958 (w), 2924 (w), 2853 (w), 2285 (w), 1626 (w), 1438 (vs), 1394 (m), 1376 (m), 1239 (s), 1107 (w), 1010 (w), 947 (s), 928 (s), 874 (w), 852 (m), 835 (m), 776 (m), 748 (s), 735 (m), 668 (s), 607 (w), 407 (w), 428 (w) cm^{-1}.

$FeCp^*_2$[BCNB^{Br}]

$$\mathrm{Ag[BCNB^{Br}]} \xrightarrow[\text{74\%}]{\mathrm{FeCp^*_2}\atop \mathrm{CH_2Cl_2},\ \text{15 min, rt}} \mathrm{FeCp^*_2[BCNB^{Br}]}$$

Ag[BCNB^{Br}] (30 mg, 0.019 mmol, 1.0 eq.) was placed in a Schlenk tube and dissolved in CH_2Cl_2 (3 mL). $FeCp^*_2$ (6.5 mg, 0.020 mmol, 1.05 eq.) was added, forming instantly a dark suspension, which was then stirred for approximately 15 min. The reaction mixture was filtered through a syringe filter, and the solvent was evaporated under reduced pressure. $FeCp^*_2$[BCNB^{Br}] (25 mg, 0.014 mmol, 74%) was obtained as a dark-greenish solid.

Single crystals for scXRD analysis were obtained by vapor diffusion of *n*-pentane into a diluted solution of $FeCp^*_2$[BCNB^{Br}] in *o*DFB.

FT-IR (ATR) $\tilde{\nu}$ = 2962 (w), 2923 (w), 2829 (w), 2852 (w), 2309 (w), 1625 (w), 1582 (vw), 1450 (vs), 1437 (vs), 1378 (m), 1243 (s), 1071 (vw), 1021 (m), 945 (s), 927(s), 876 (m), 835(m), 778 (w), 749 (s), 671 (s), 597 (w), 449 (m) cm^{-1}.

$N(4\text{-}BrC_6H_4)_3[BCNB^{Br}]$

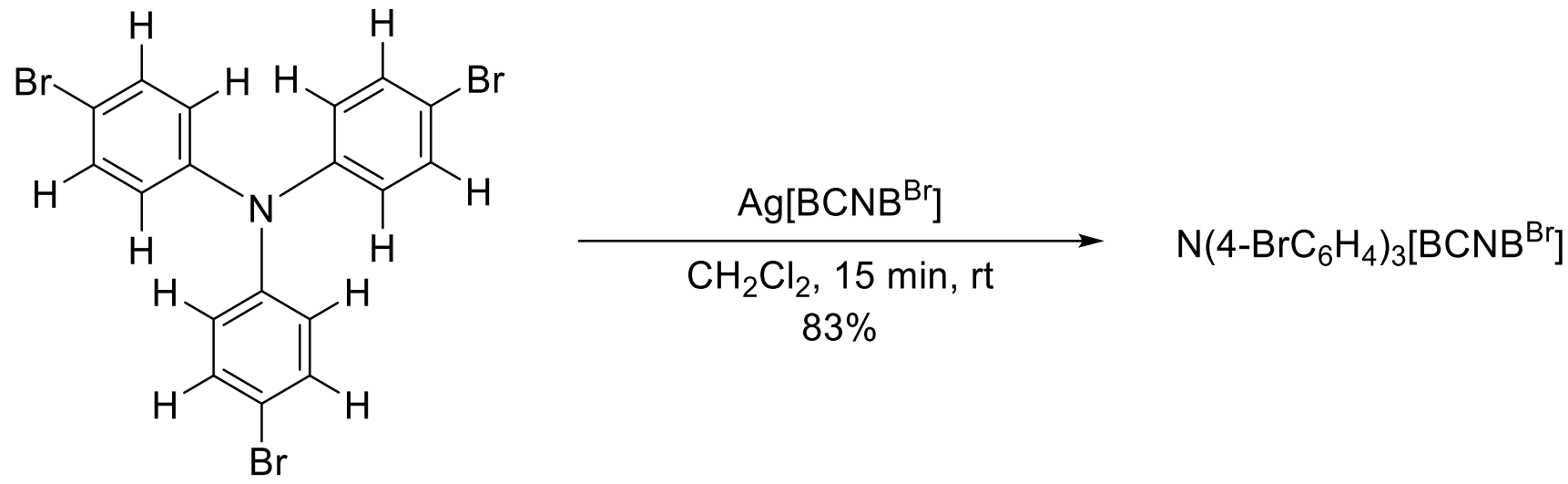

$Ag[BCNB^{Br}]$ (82 mg, 0.054 mmol, 1.0 eq.) was placed in a Schlenk tube and dissolved in CH_2Cl_2 (5 mL). $N(4\text{-}BrC_6H_4)_3$ (26 mg, 0.054 mmol, 1.0 eq.) was added, forming instantly a deep blue solution, which was then stirred for approximately 15 min. The reaction mixture was filtered through a syringe filter and the solvent was evaporated under reduced pressure. $N(4\text{-}BrC_6H_4)_3[BCNB^{Br}]$ (85 mg, 0.045 mmol, 83%) was obtained as a blue solid.

Single crystals for scXRD analysis were obtained by cooling a concentrated solution of $N(4\text{-}BrC_6H_4)_3[BCNB^{Br}]$ in CH_2Cl_2 to –70 °C.

FT-IR (ATR) $\tilde{\nu}$ = 3118 (vw), 2286 (w), 1626 (w), 1546 (w), 1484 (w), 1439 (vs), 1395 (m), 1376 (m), 1311 (w), 1239 (s), 1169 (w), 1108 (w), 1065 (m), 1002 (m), 947 (s), 927 (s), 873 (m), 853 (m), 831 (m), 776 (m), 747 (s), 733 (m), 667 (s), 606 (m), 568 (w), 507 (m), 471 (w), 428 (w) cm^{-1}.

$[C_6H_4S_2C_6H_4][BCNB^{Br}]$

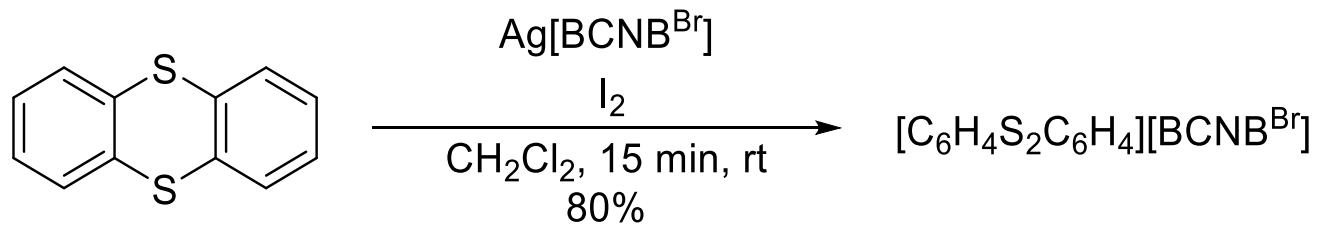

$Ag[BCNB^{Br}]$ (42 mg, 0.028 mmol, 1.0 eq.) was placed in a Schlenk tube and dissolved in CH_2Cl_2 (5 mL). Thianthrene (6 mg, 0.028 mmol, 1.0 eq.) was added, forming a clear colourless solution. After addition of I_2 (4.0 mg, 0.016 mmol, 0.57 eq.), a deep pink solution with a colorless precipitate formed, which was then stirred for approximately 15 min. The reaction mixture was filtered through a syringe filter, and the solvent was evaporated under reduced pressure. After drying the pink solid for 30 min, it was washed with *n*-pentane (3 x 2 mL), until the *n*-pentane solution remained colourless. After drying, $[C_6H_4S_2C_6H_4][BCNB^{Br}]$ (36 mg, 0.022 mmol, 80%) was obtained as a pink solid.

Single crystals for scXRD analysis were obtained by vapor diffusion of *n*-pentane into a solution of $[C_6H_4S_2C_6H_4][BCNB^{Br}]$ in CH_2Cl_2.

FT-IR (ATR) $\tilde{\nu}$ = 2953 (w), 2925 (w), 2856 (w), 2286 (w), 1626 (w), 1439 (vs), 1393 (m), 1375 (m), 1303 (w), 1240 (s), 1117 (w), 1103 (w), 1014 (m), 948 (s), 928 (s), 870 (m), 835 (m), 818 (m), 776 (m), 748 (vs), 668 (s), 605 (m), 544 (w), 528 (w), 502 (w), 473 (m), 428 (w) cm^{-1}.

$N(2,4\text{-}Br_2C_6H_3)_3[BCNB^{Br}]$

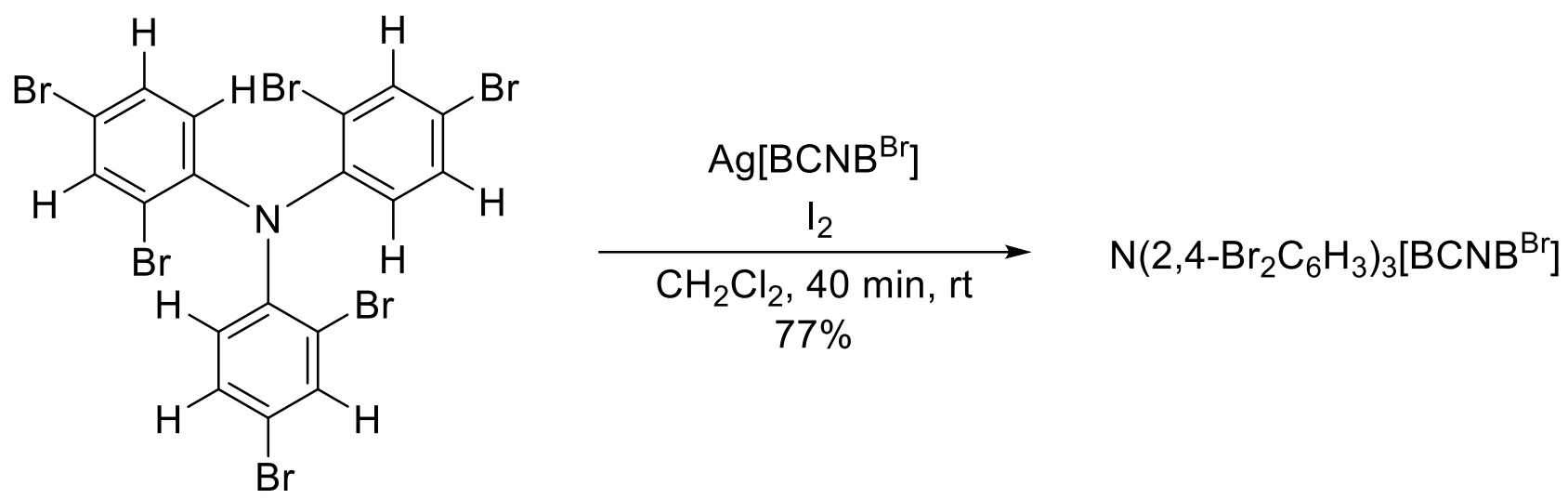

$Ag[BCNB^{Br}]$ (347 mg, 0.228 mmol, 1.0 eq.) was placed in a Schlenk tube and dissolved in CH_2Cl_2 (10 mL). $N(2,4\text{-}Br_2C_6H_3)_3$ (164 mg, 0.228 mmol, 1.0 eq.) was added, forming a clear, colourless solution. After the addition of I_2 (40 mg, 0.16 mmol, 0.70 eq.), a pink solution formed first, followed shortly by a deep green solution with a colourless precipitate. After stirring the mixture for a total of 3 h, it was filtered through a syringe filter and the solvent was evaporated under reduced pressure. After drying the green solid for 30 min, it was washed with *n*-pentane (3 x 5 mL), until the *n*-pentane solution remained colourless. After drying, $N(2,4\text{-}Br_2C_6H_3)_3[BCNB^{Br}]$ (375 mg, 0.176 mmol, 77%) was obtained as a green solid.

Single crystals for scXRD analysis were obtained by vapor diffusion of *n*-pentane into a solution of $N(2,4\text{-}Br_2C_6H_3)_3[BCNB^{Br}]$ in CH_2Cl_2.

FT-IR (ATR) $\tilde{\nu}$ = 3085 (w), 2285 (w), 1626 (w), 1541 (s), 1438 (vs), 1394 (m), 1373 (s), 1294 (w), 1264 (m), 1239 (s), 1144 (w), 1078 (s), 1069 (s), 1039 (m), 1015 (w), 947 (s), 928 (s), 876 (m), 835 (w), 813 (m), 776 (w), 747 (s), 668 (s), 605 (w), 546 (w), 525 (w), 431 (w) cm^{-1}.

NMR spectra

Ag[BCNB^{Br}]

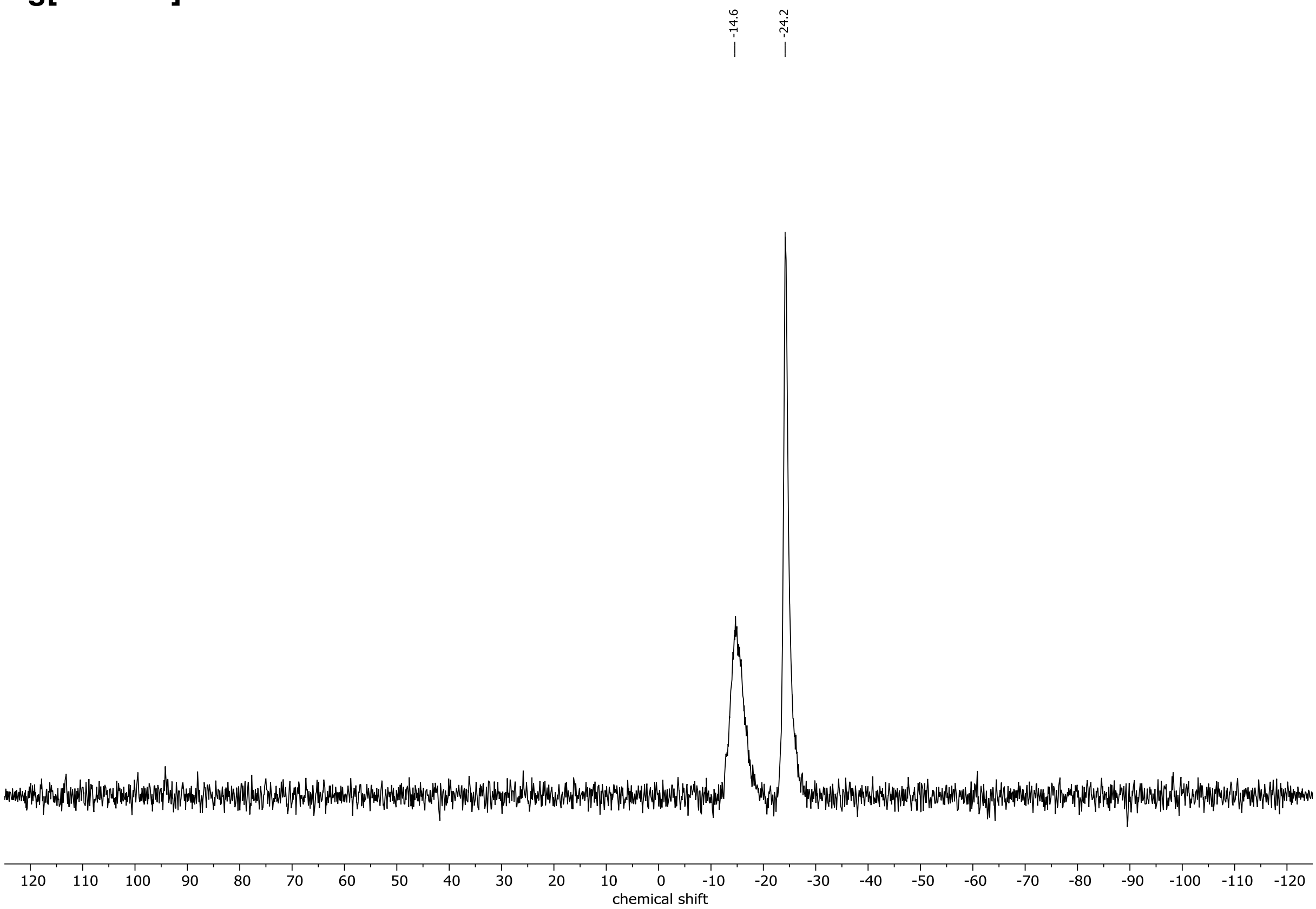

Figure S1. ^{11}B NMR (CD_2Cl_2, rt, 128.14 MHz) spectrum of Ag[BCNB^{Br}].

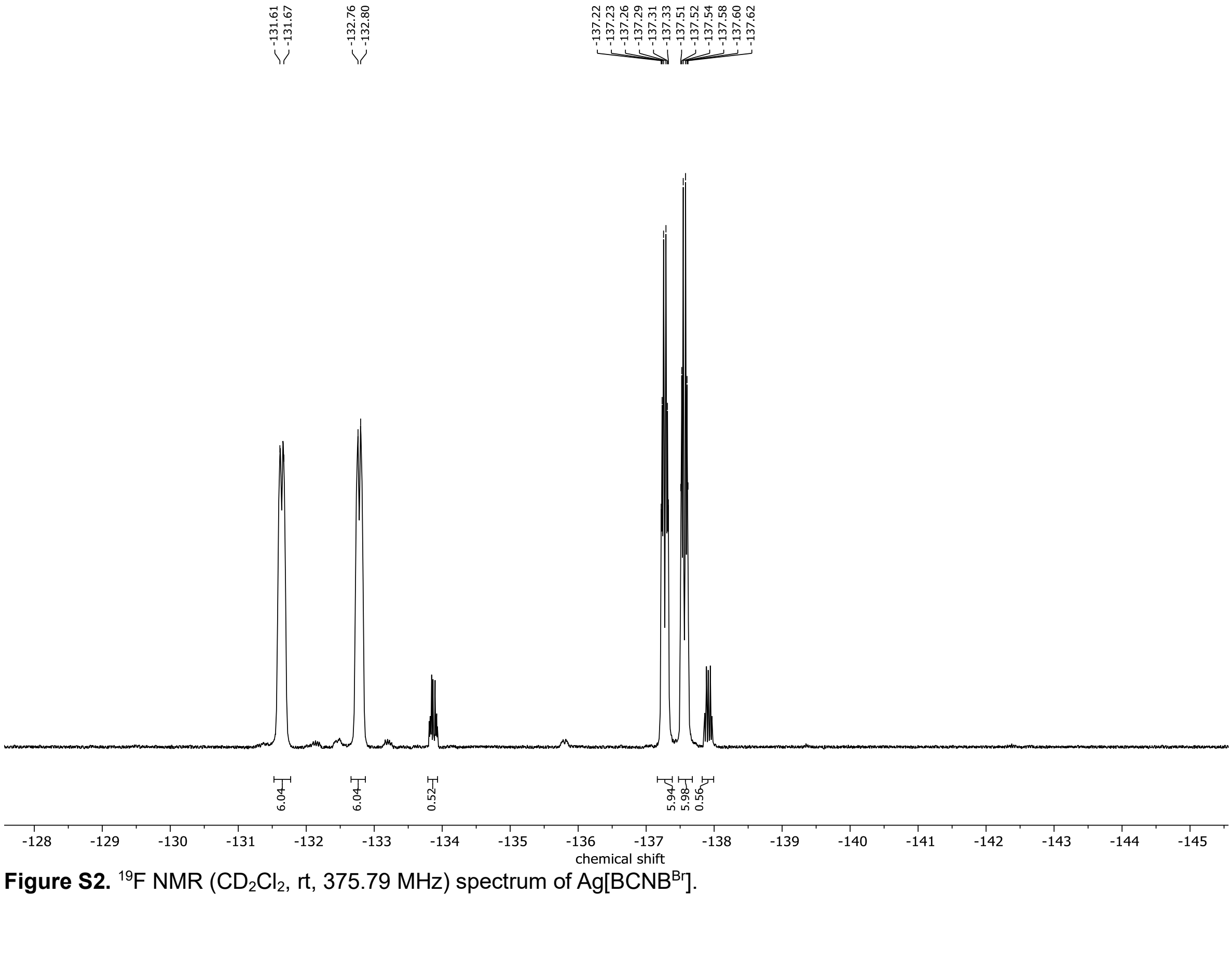

Figure S2. ^{19}F NMR (CD_2Cl_2, rt, 375.79 MHz) spectrum of Ag[BCNB^{Br}].

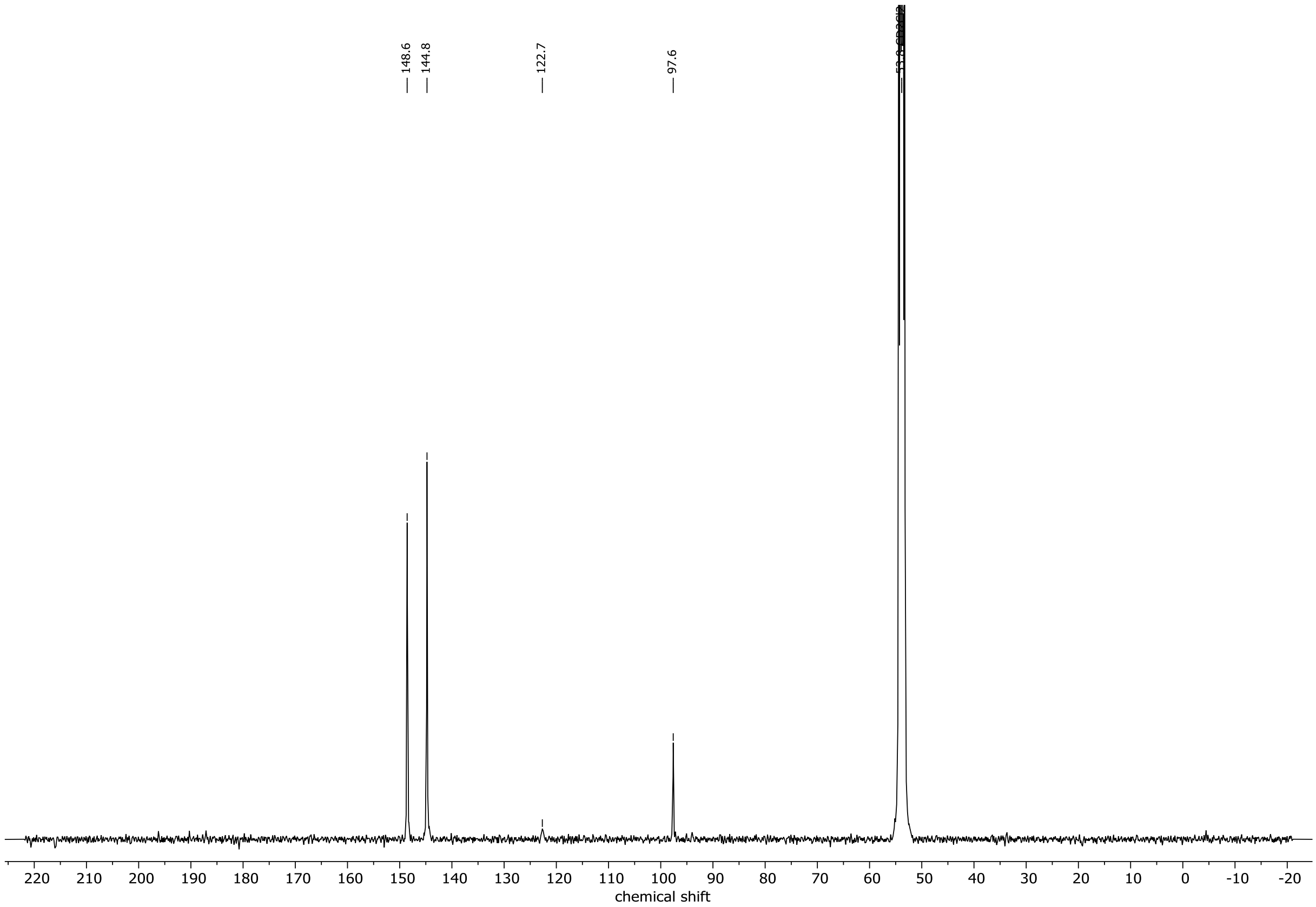

Figure S3. ^{13}C NMR{^{19}F} (CD_2Cl_2, rt, 100.42 MHz) spectrum of Ag[BCNB^{Br}].

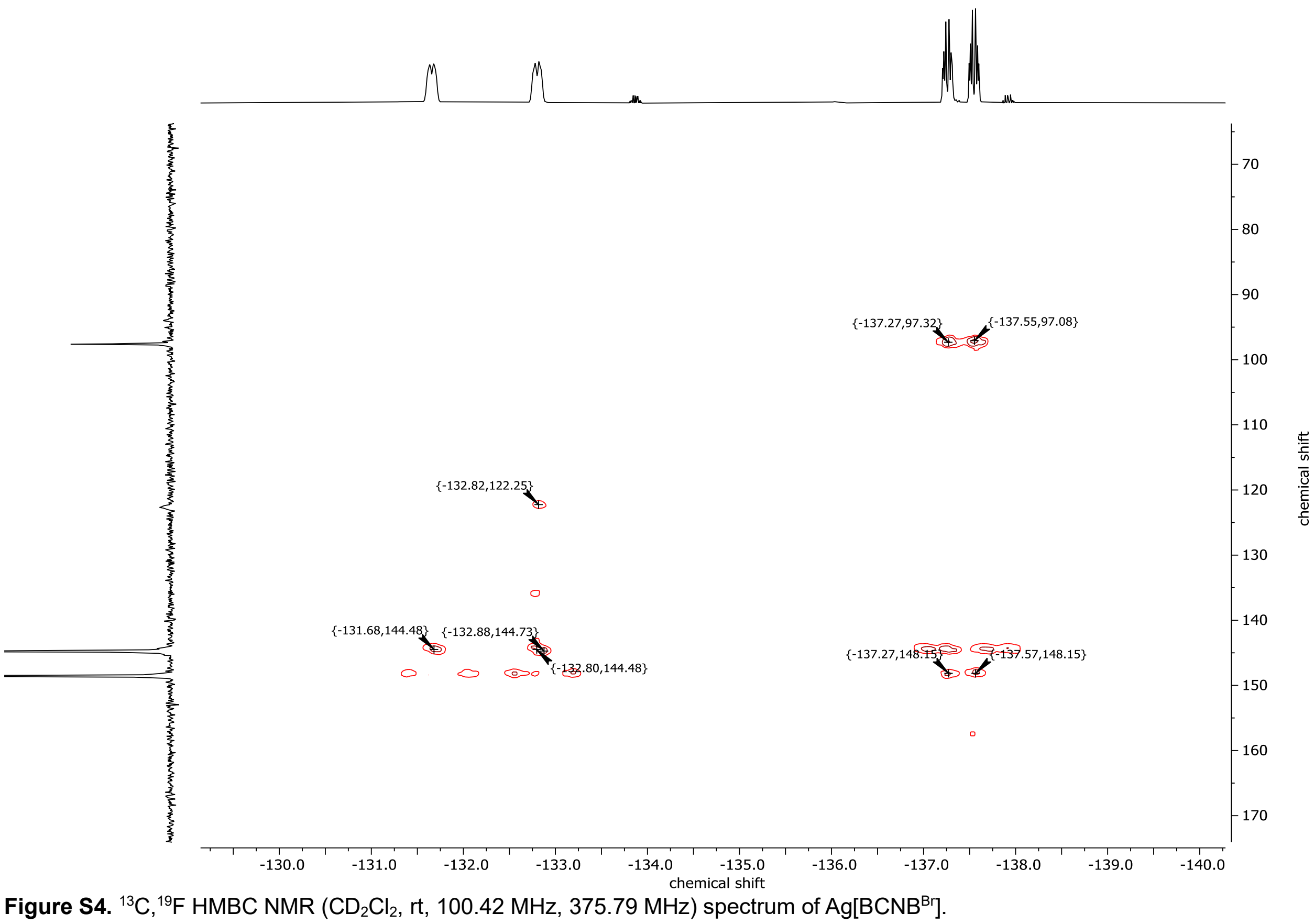

Figure S4. ^{13}C,^{19}F HMBC NMR (CD_2Cl_2, rt, 100.42 MHz, 375.79 MHz) spectrum of Ag[BCNB^{Br}].

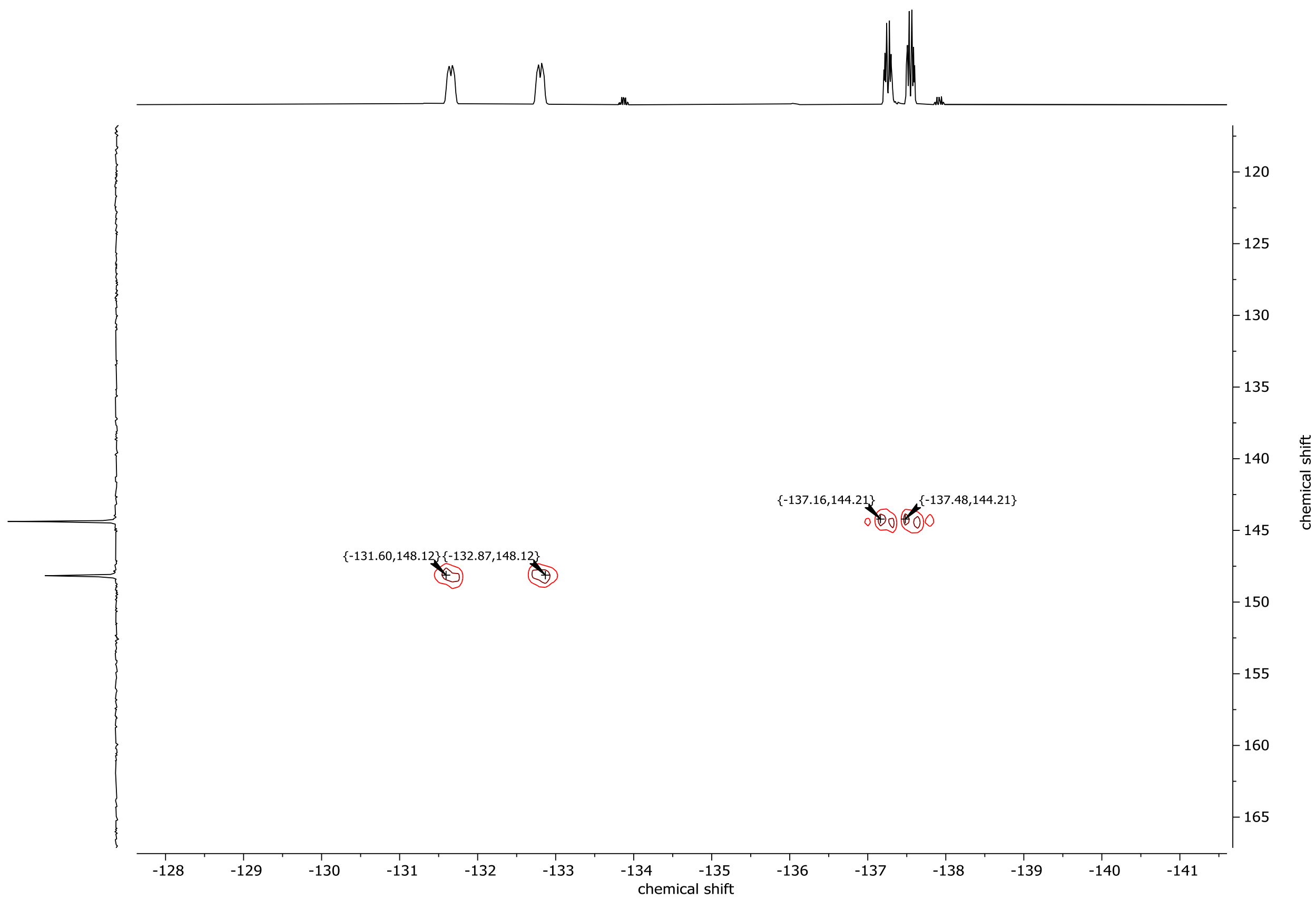

Figure S5. ^{13}C,^{19}F HMQC NMR (CD_2Cl_2, rt, 100.42 MHz, 375.79 MHz) spectrum of Ag[BCNB^{Br}].

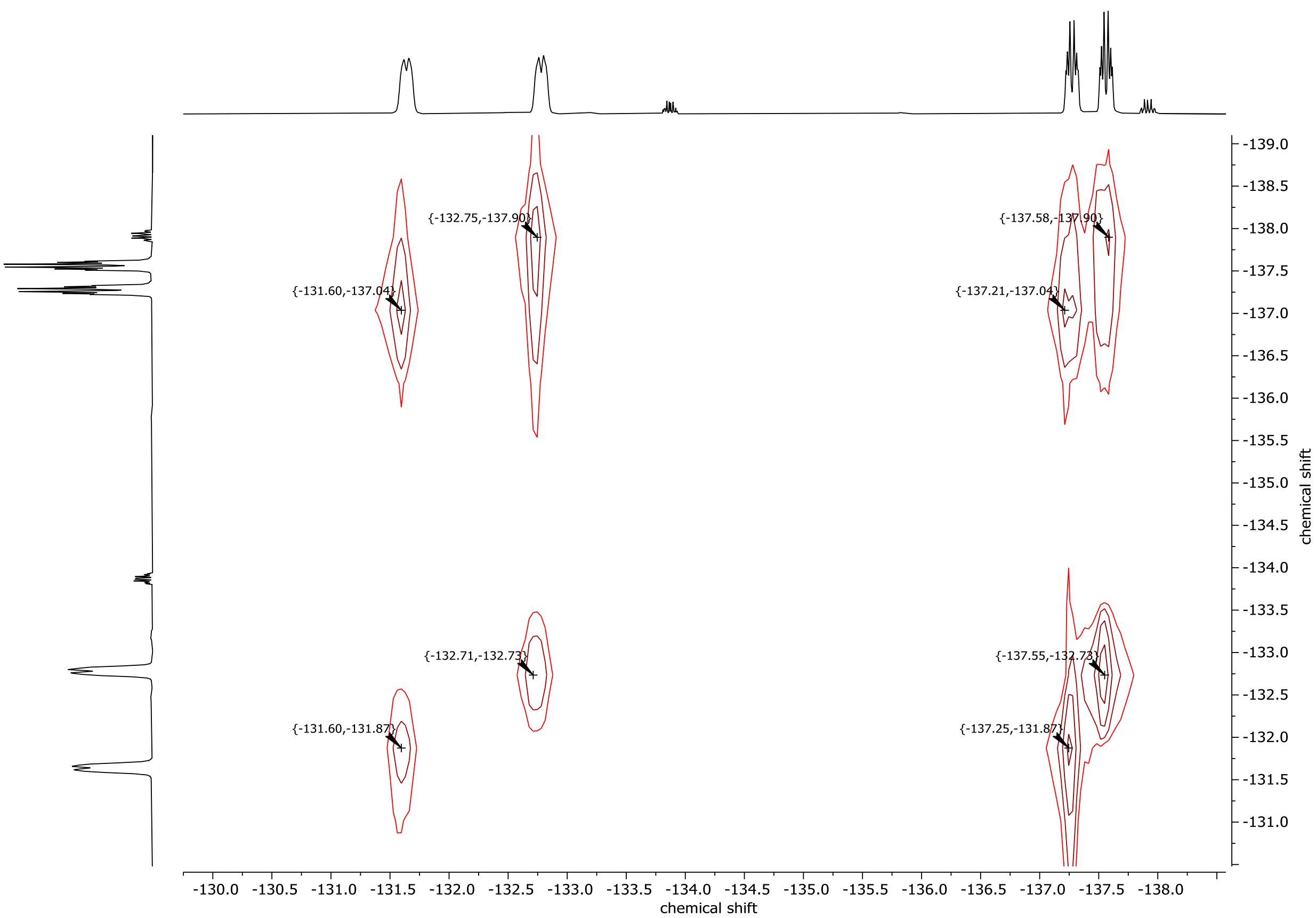

Figure S6. ^{19}F,^{19}F COSY NMR (CD_2Cl_2, rt, 375.79 MHz) spectrum of Ag[BCNB^{Br}].

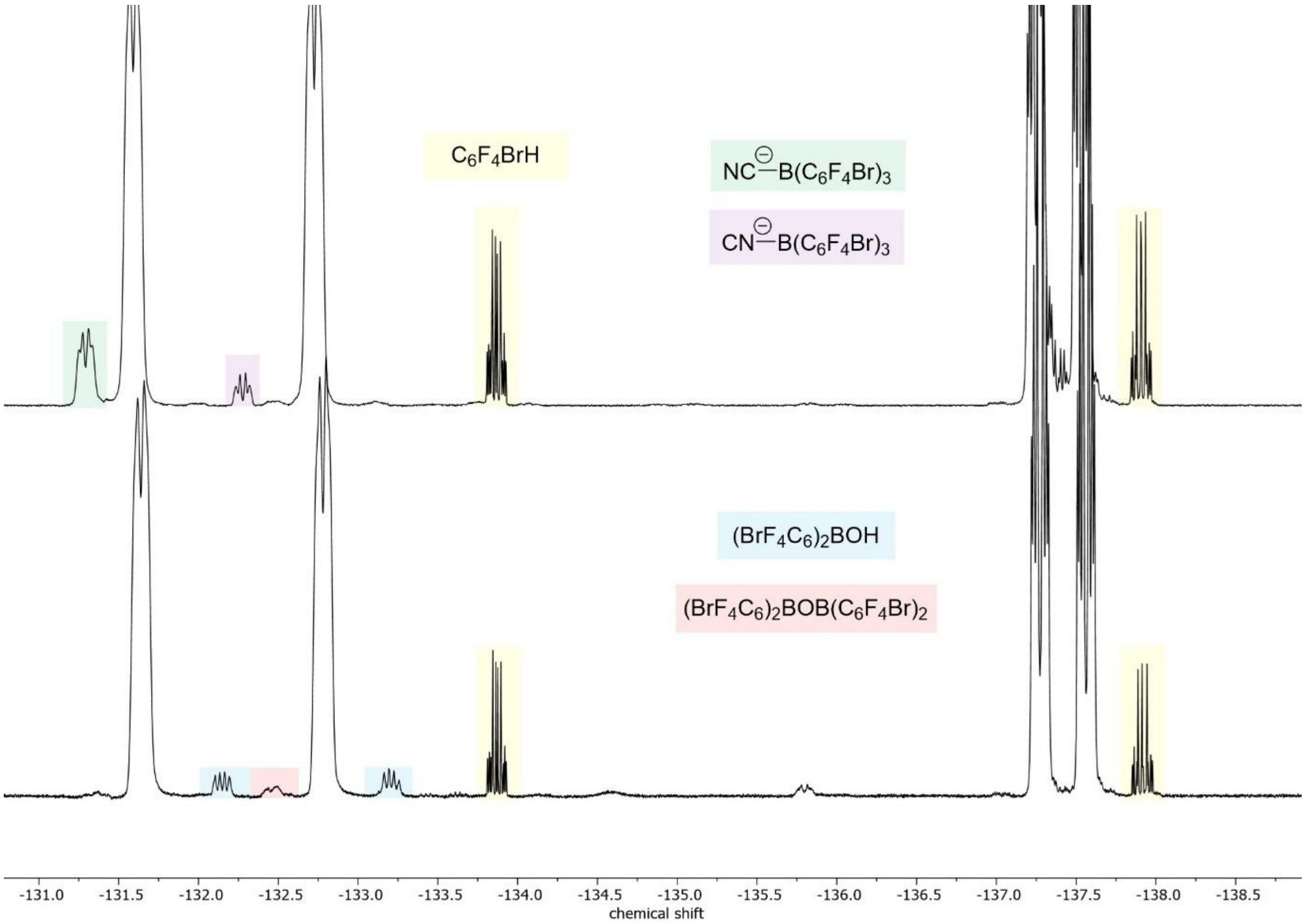

Figure S7. ^{19}F NMR (CD_2Cl_2, rt, 375.79 MHz) spectra of Ag[BCNB^{Br}] with different impurities.

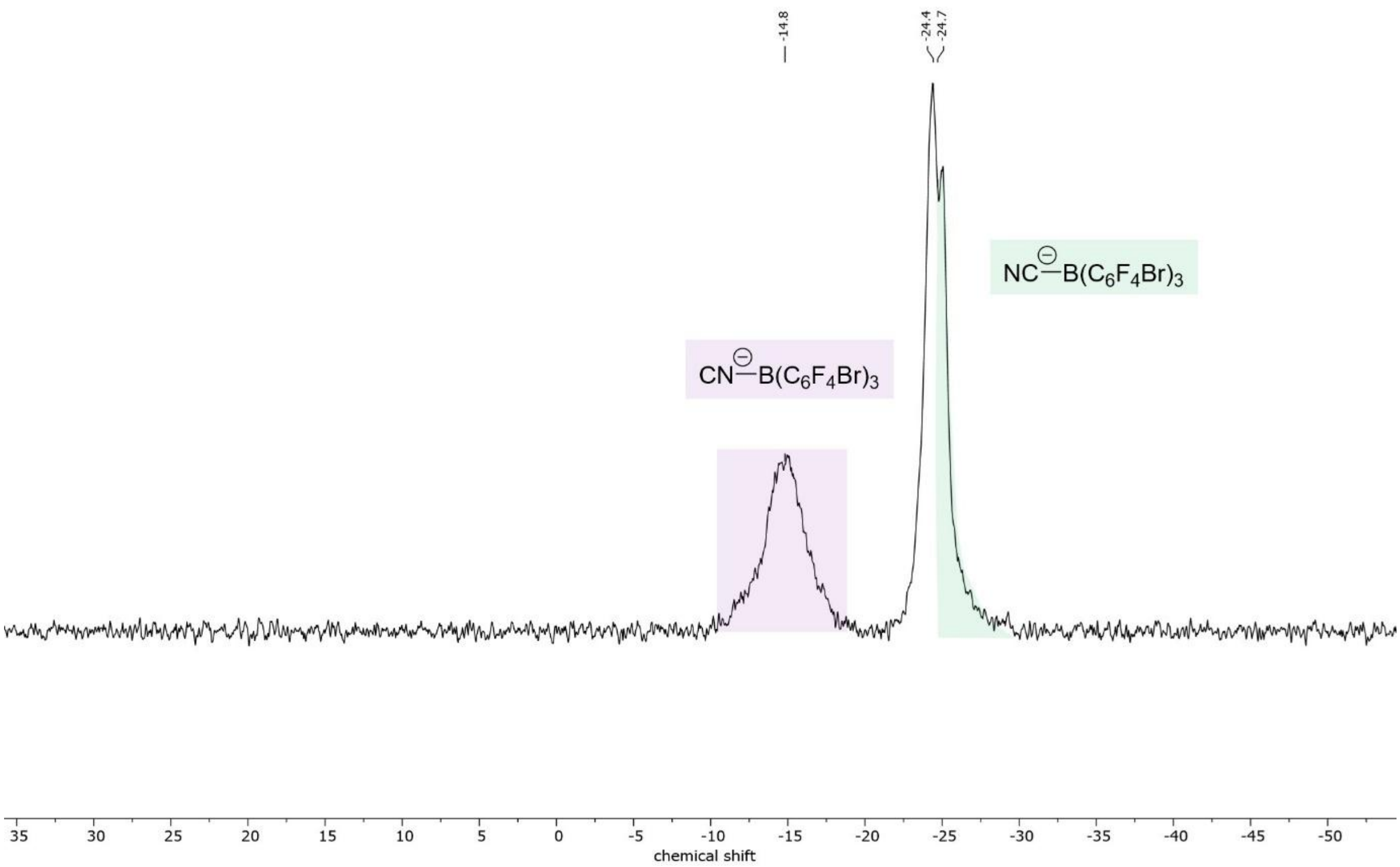

Figure S8. ^{11}B NMR (CD_2Cl_2, rt, 128.14 MHz) spectrum of Ag[BCNB^{Br}] with monoadducts as impurities.

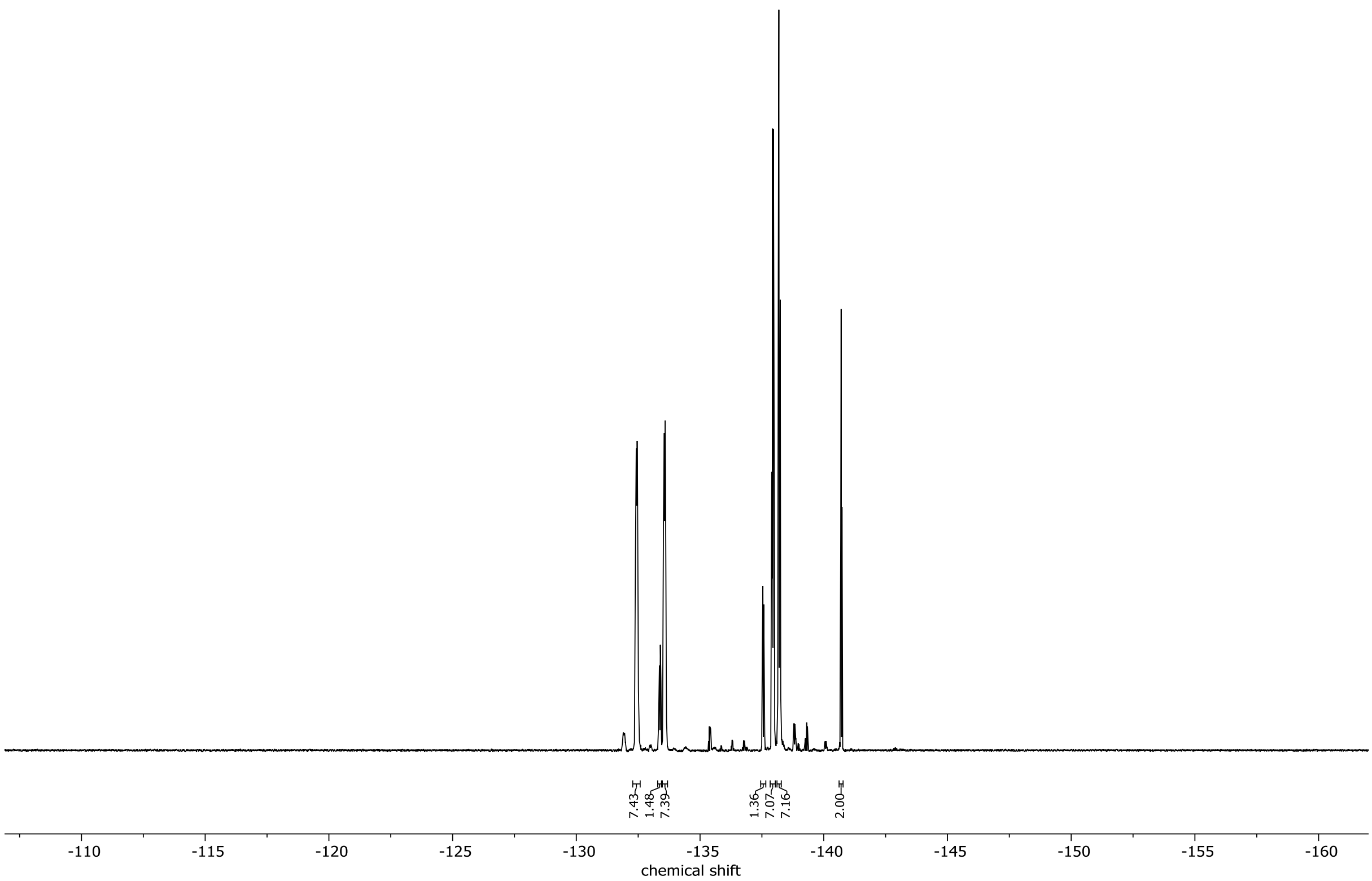

Figure S9. ^{19}F NMR (CD_3CN, rt, 375.79 MHz) spectrum of Ag[BCNB^{Br}] referenced with *o*DFB.

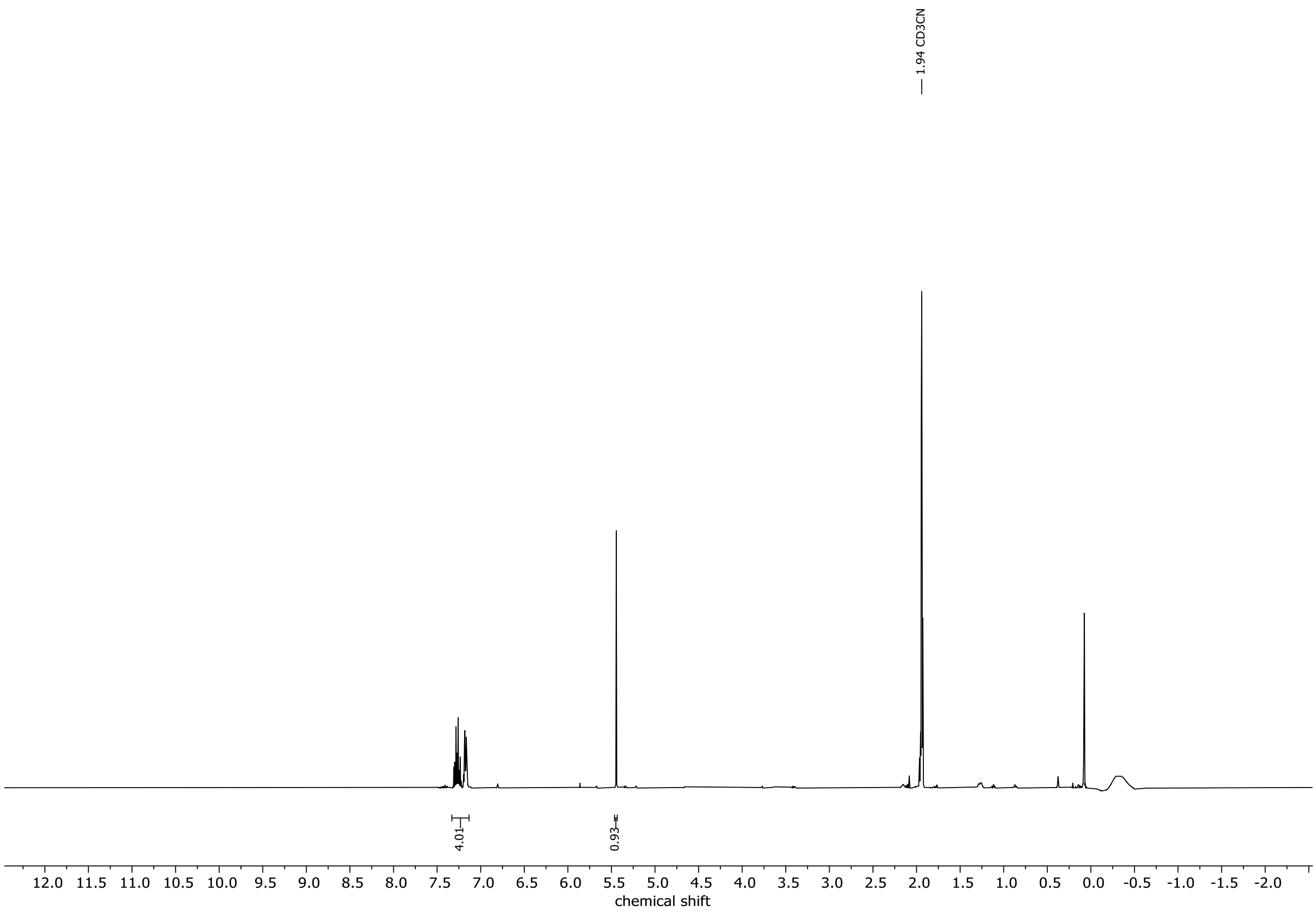

Figure S10. ^{1}H NMR (CD_3CN, rt, 400 MHz) spectrum of Ag[BCNB^{Br}] referenced with *o*DFB.

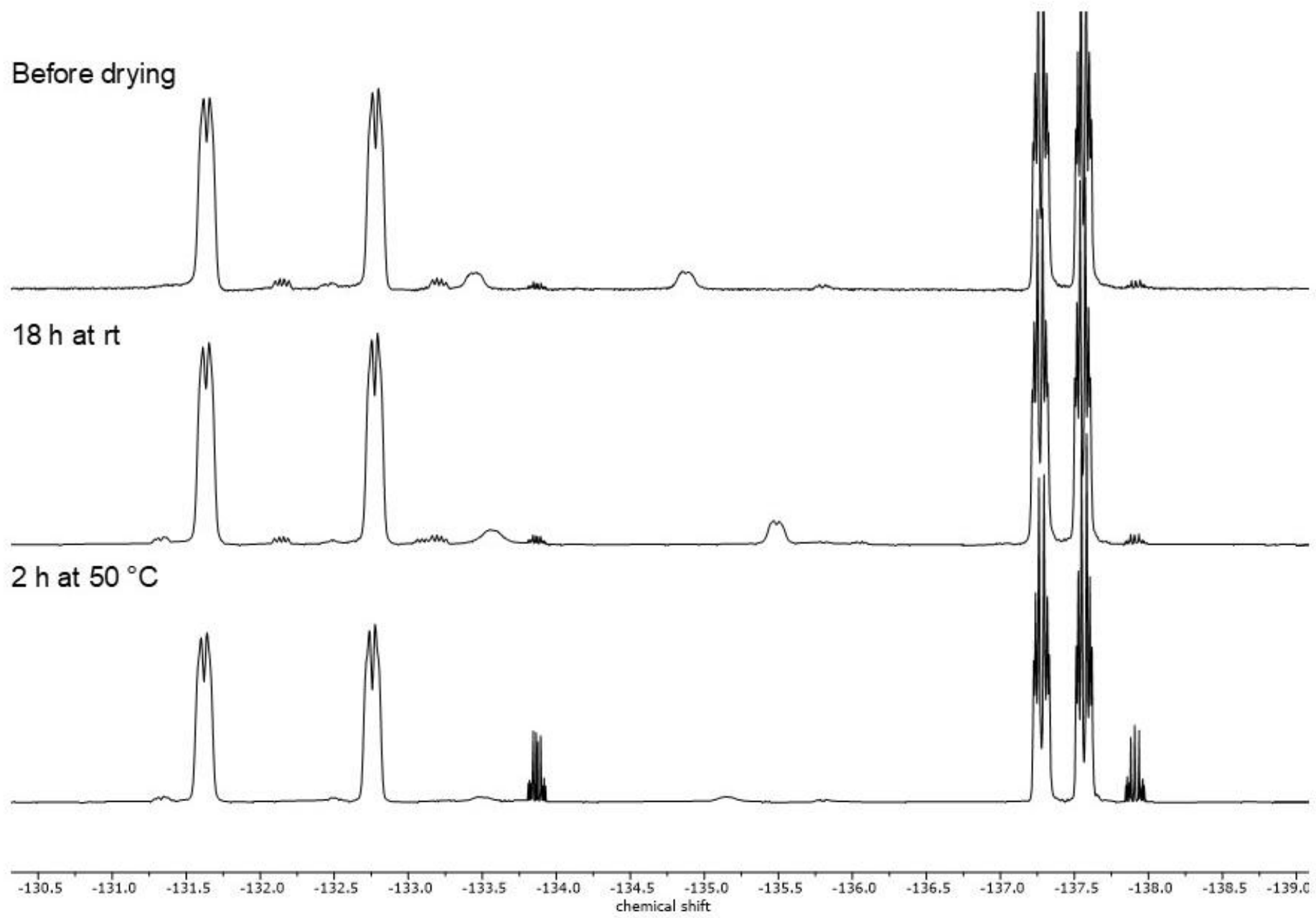

Figure S11. ^{19}F NMR (CD_2Cl_2, rt, 375.79 MHz) spectra of Ag[BCNB^{Br}] before drying, after drying for 18 h at room temperature and after drying for 2 h at 50 °C.

[Ag(toluene)][BCNB^{Br}]

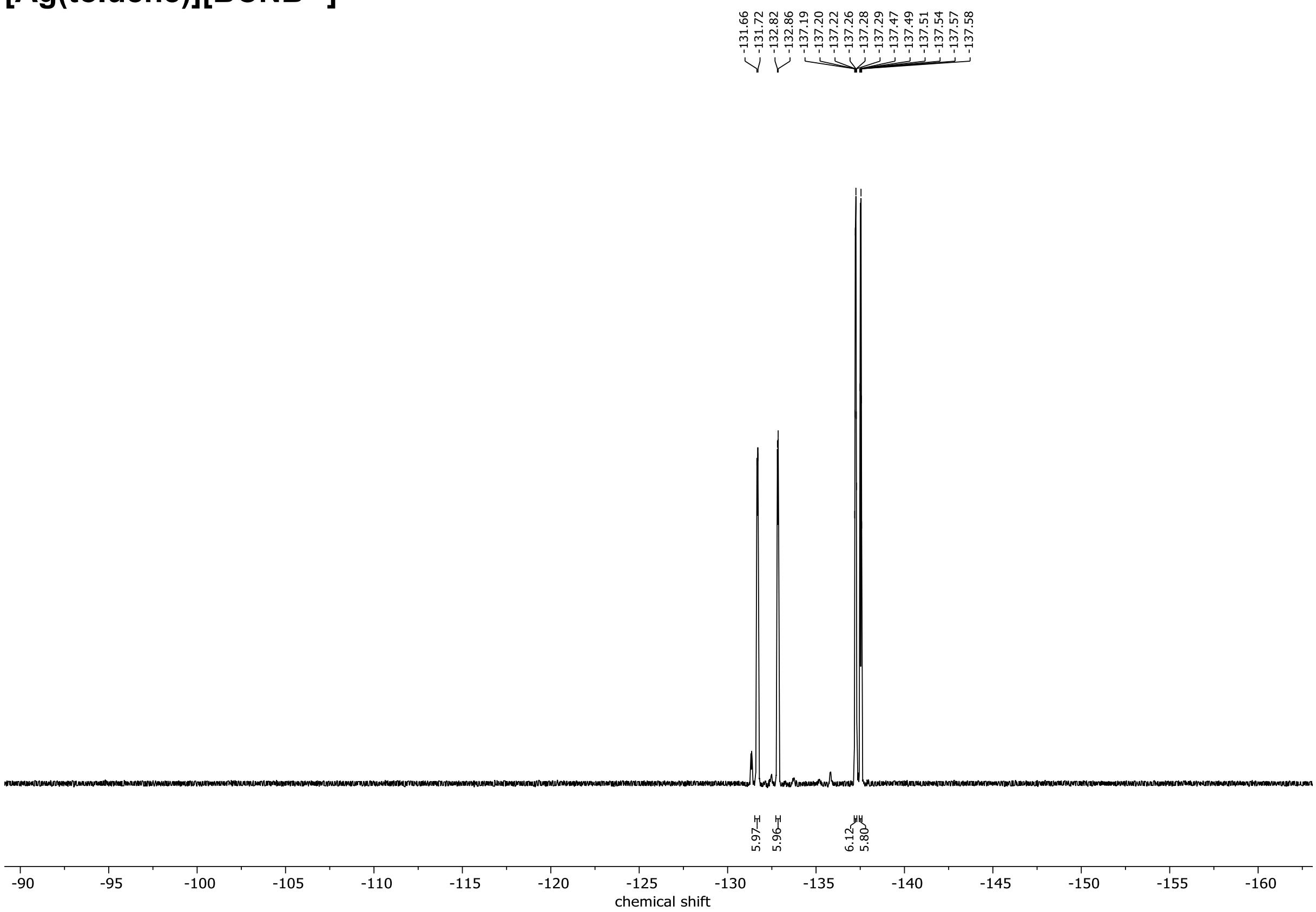

Figure S12. ^{19}F NMR (CD_2Cl_2, rt, 375.79 MHz) spectrum of [Ag(toluene)][BCNB^{Br}].

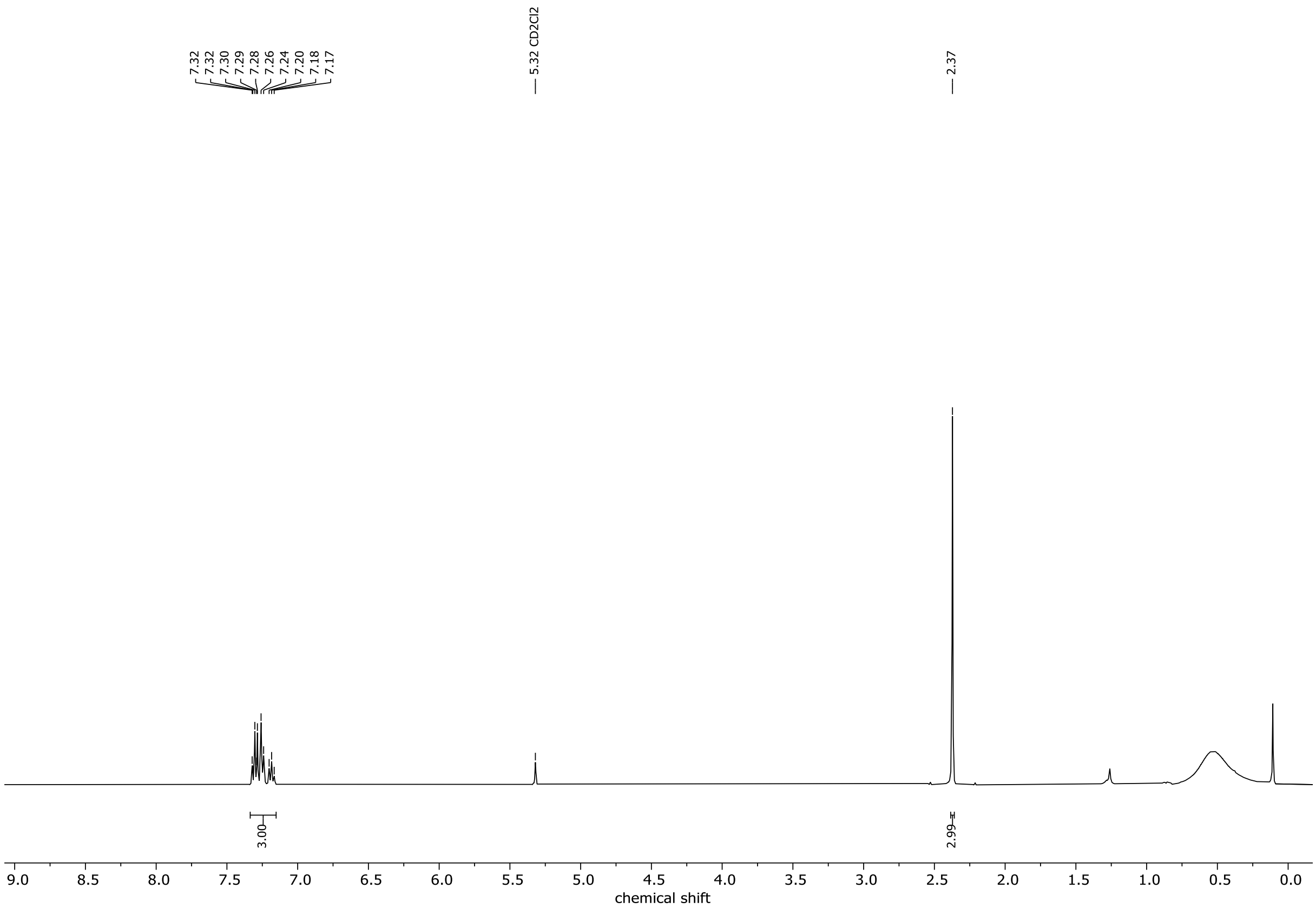

Figure S13. ^{1}H NMR (CD_2Cl_2, rt, 400 MHz) spectrum of [Ag(toluene)][BCNB^{Br}].

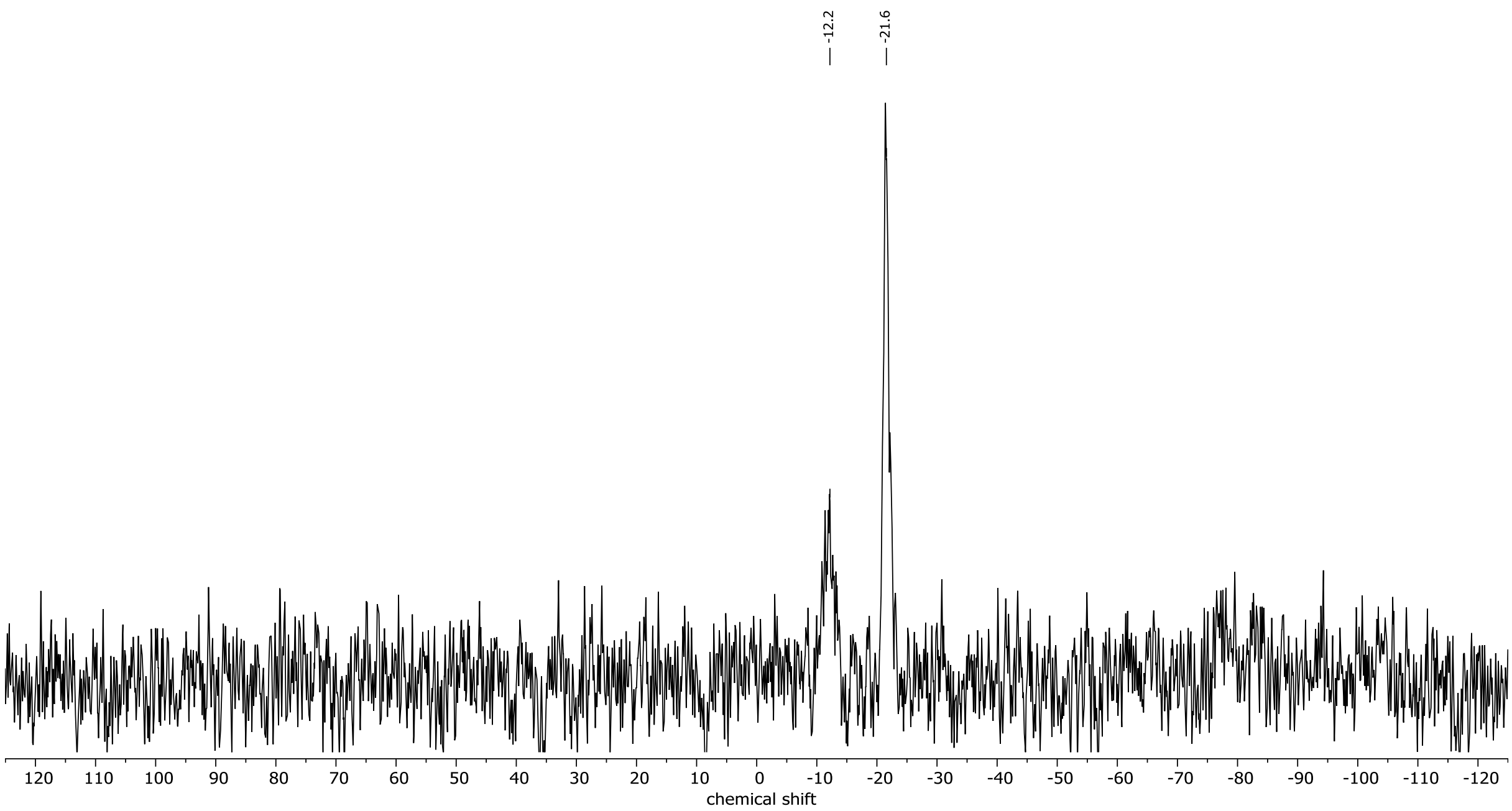

Figure S14. ^{11}B NMR (CD_2Cl_2, rt, 128.15 MHz) spectrum of [Ag(toluene)][BCNB^{Br}].

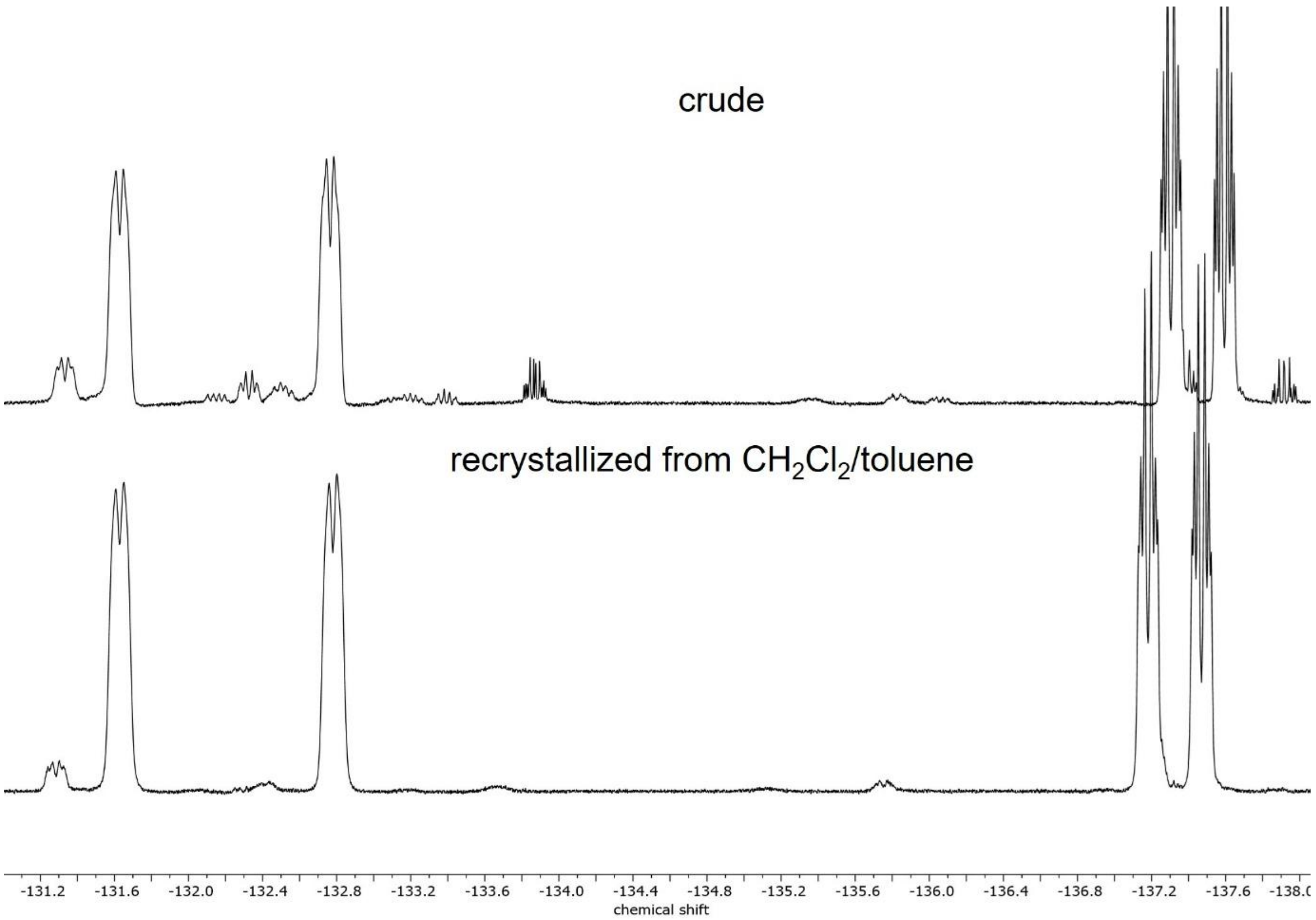

Figure S15. ^{19}F NMR (CD_2Cl_2, rt, 375.79 MHz) spectra of Ag[BCNB^{Br}] before and after recrystallization from CH_2Cl_2/toluene.

[Ag(benzene)][BCNB^{Br}]

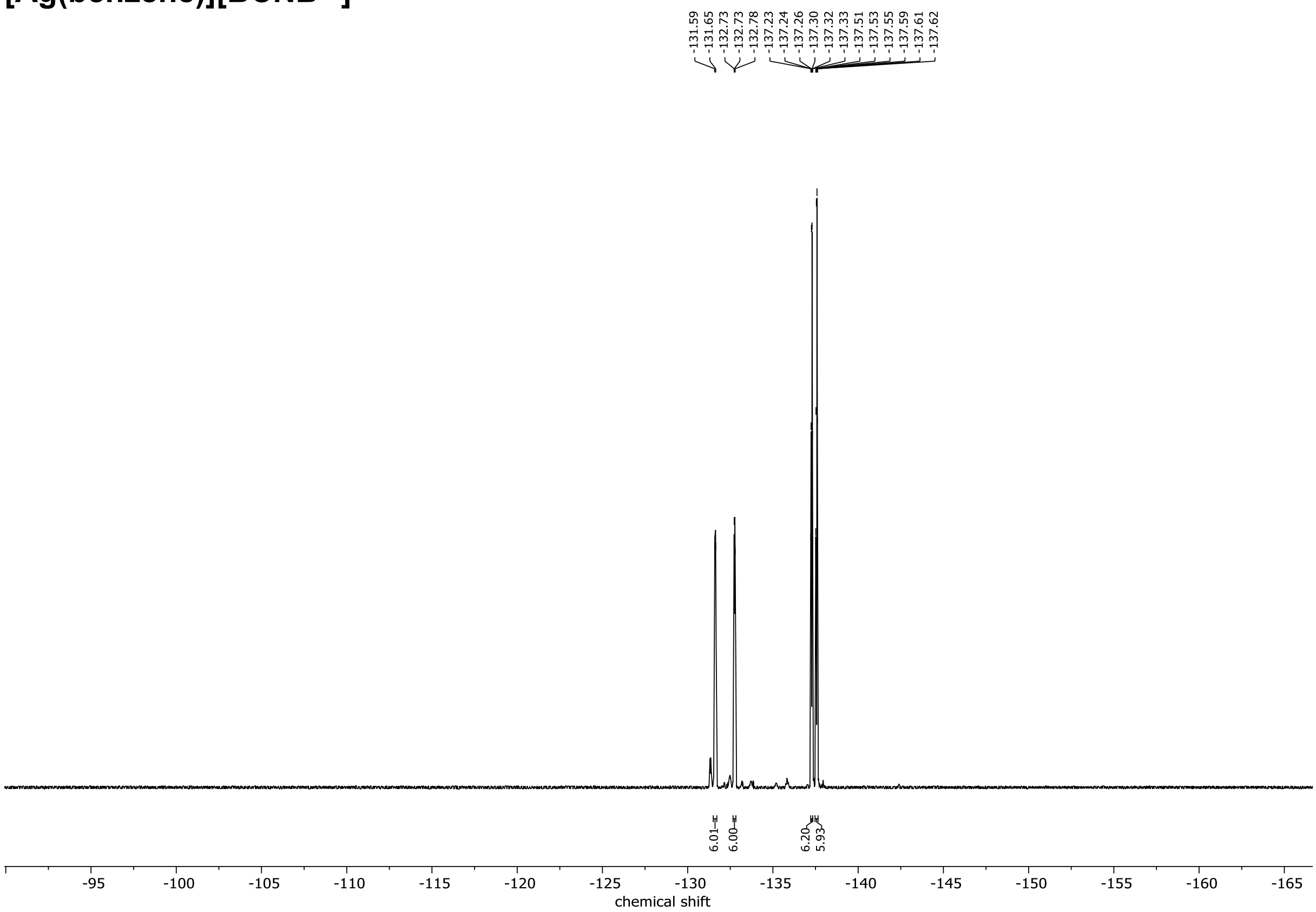

Figure S16. ^{19}F NMR (CD_2Cl_2, rt, 375.79 MHz) spectrum of [Ag(benzene)][BCNB^{Br}].

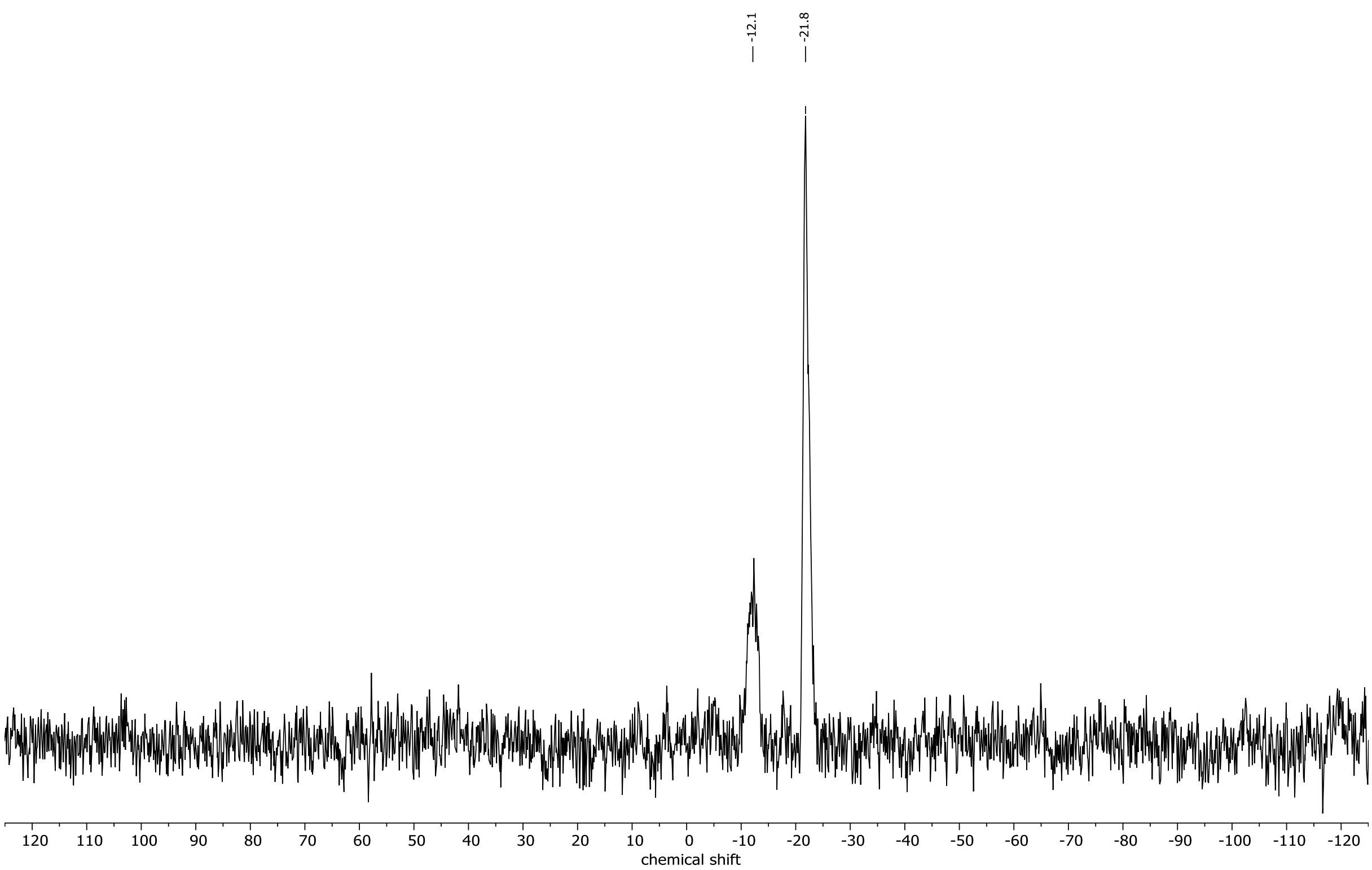

Figure S17. ^{11}B NMR (CD_2Cl_2, rt, 128.15 MHz) spectrum of [Ag(benzene)][BCNB^{Br}].

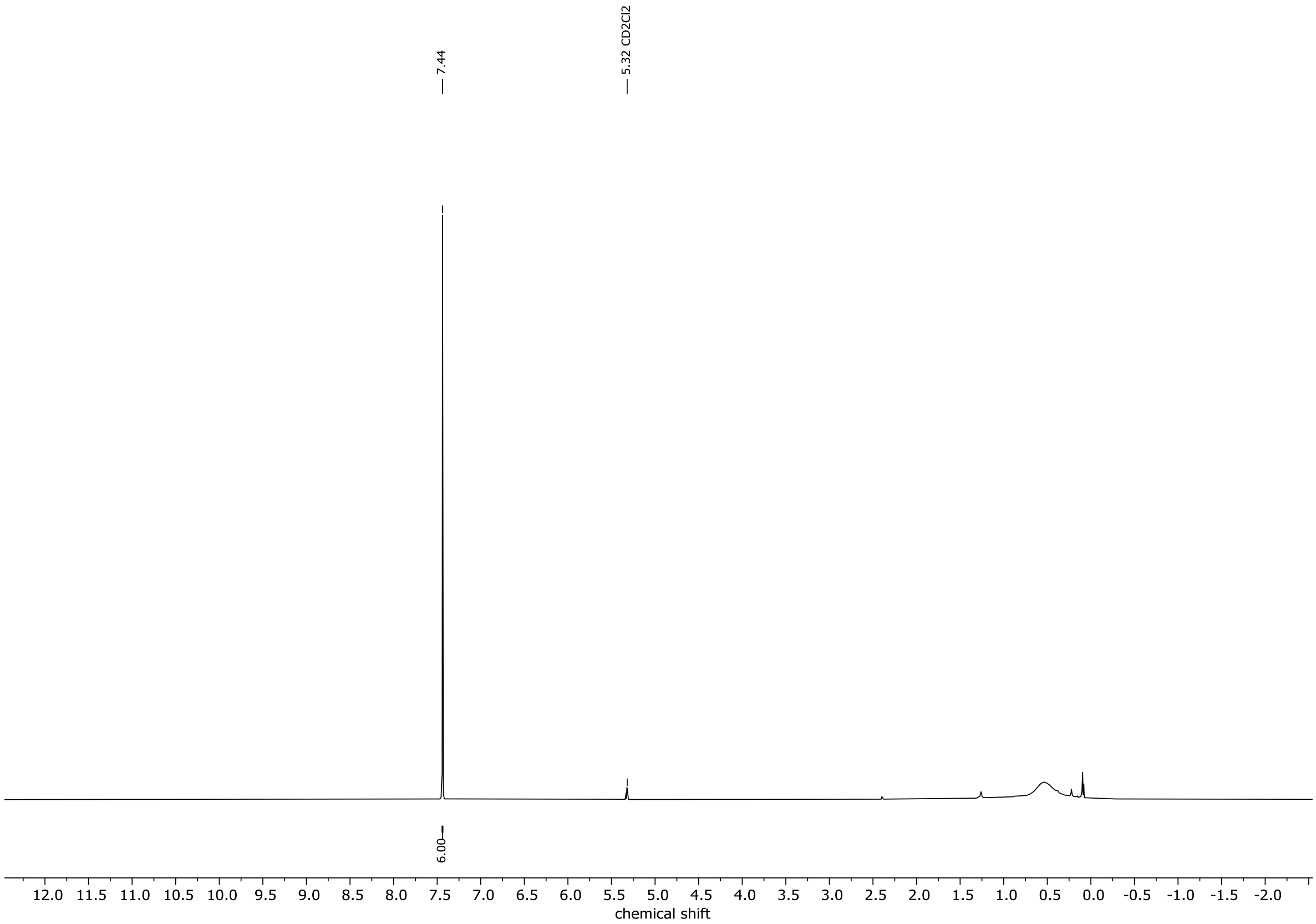

Figure S18. ^{1}H NMR (CD_2Cl_2, rt, 400 MHz) spectrum of [Ag(benzene)][BCNB^{Br}].

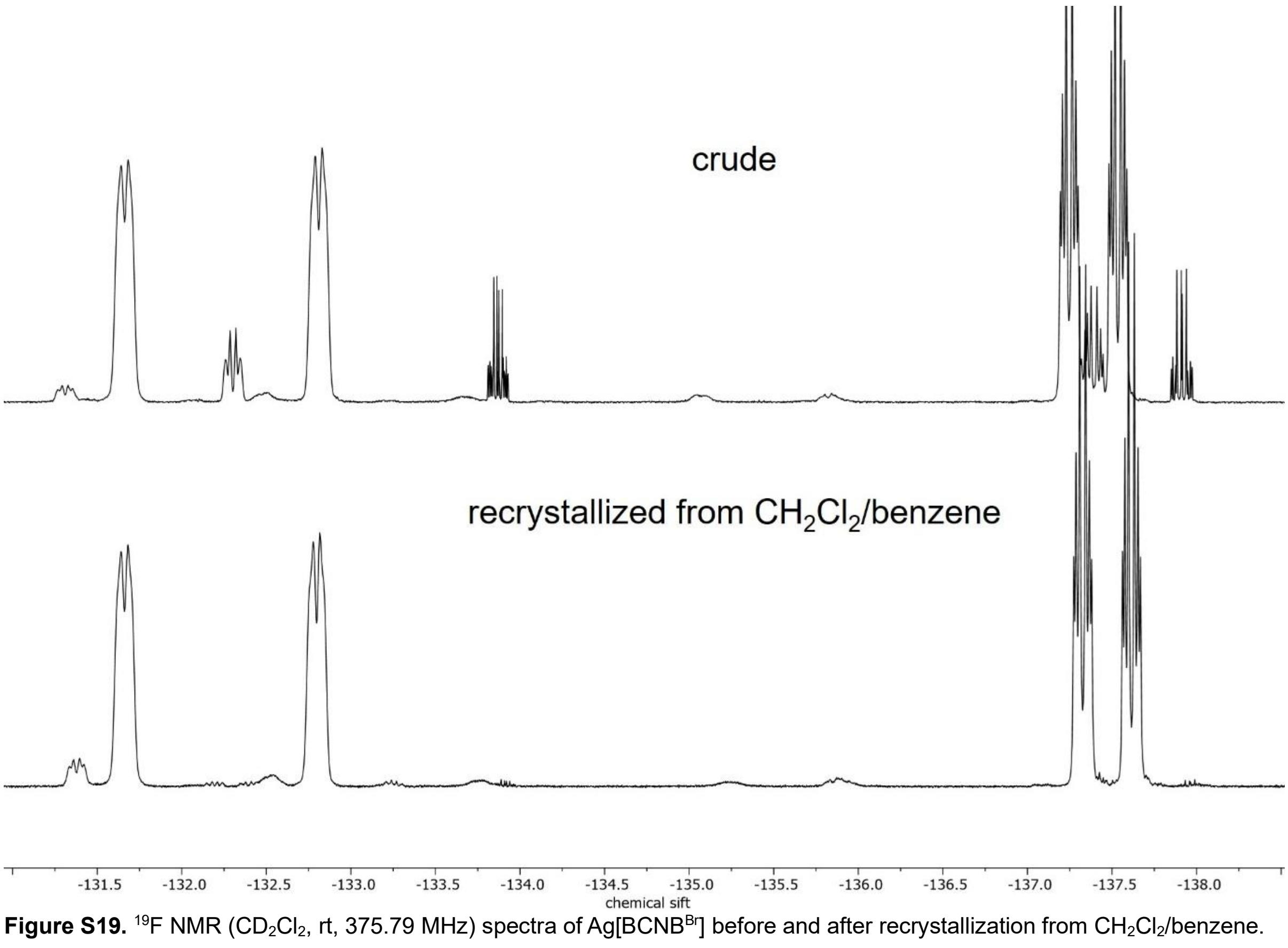

Figure S19. ^{19}F NMR (CD_2Cl_2, rt, 375.79 MHz) spectra of Ag[BCNB^{Br}] before and after recrystallization from CH_2Cl_2/benzene.

$NBu_4[BCNB^{Br}]$

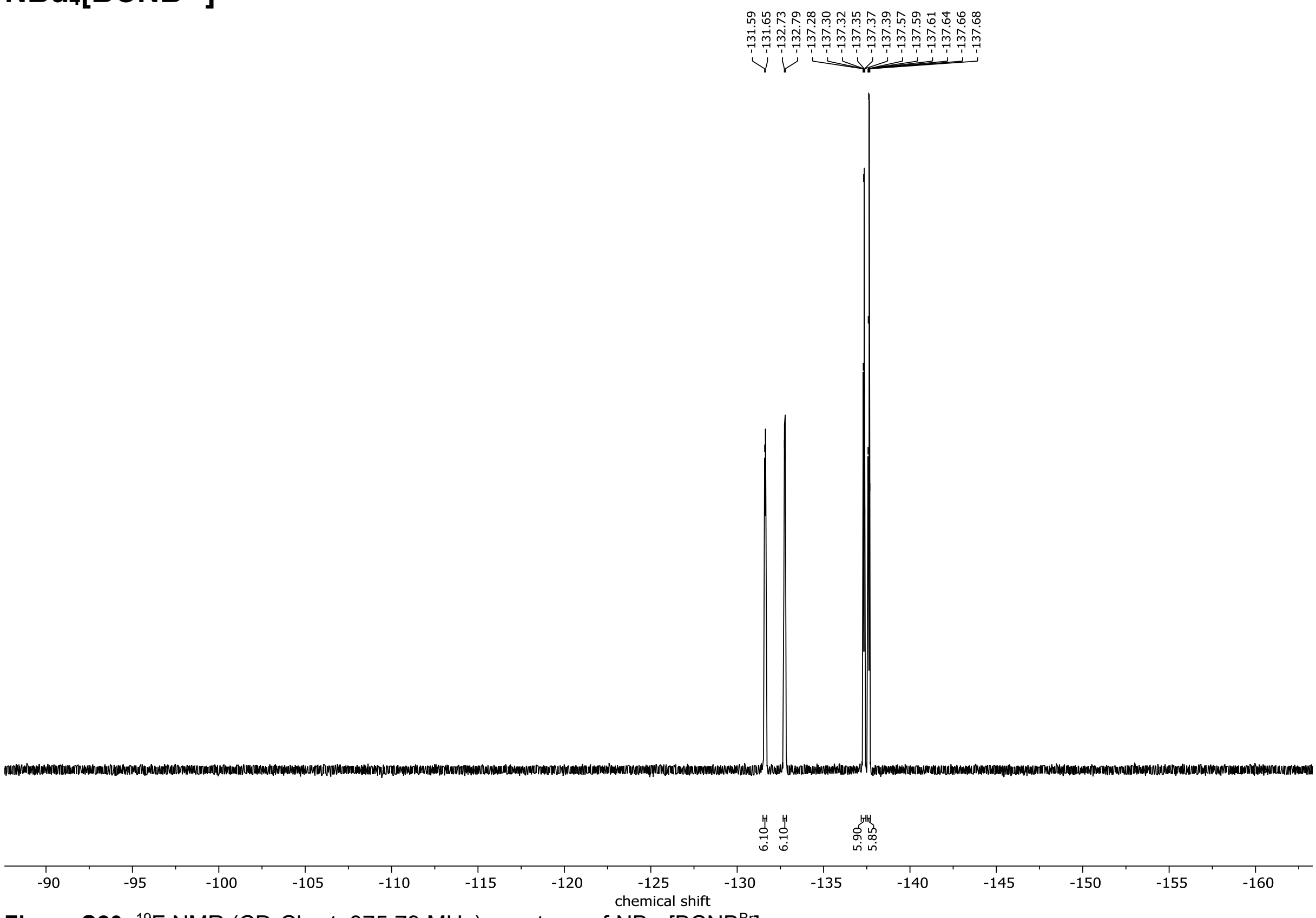

Figure S20. ^{19}F NMR (CD_2Cl_2, rt, 375.79 MHz) spectrum of $NBu_4[BCNB^{Br}]$.

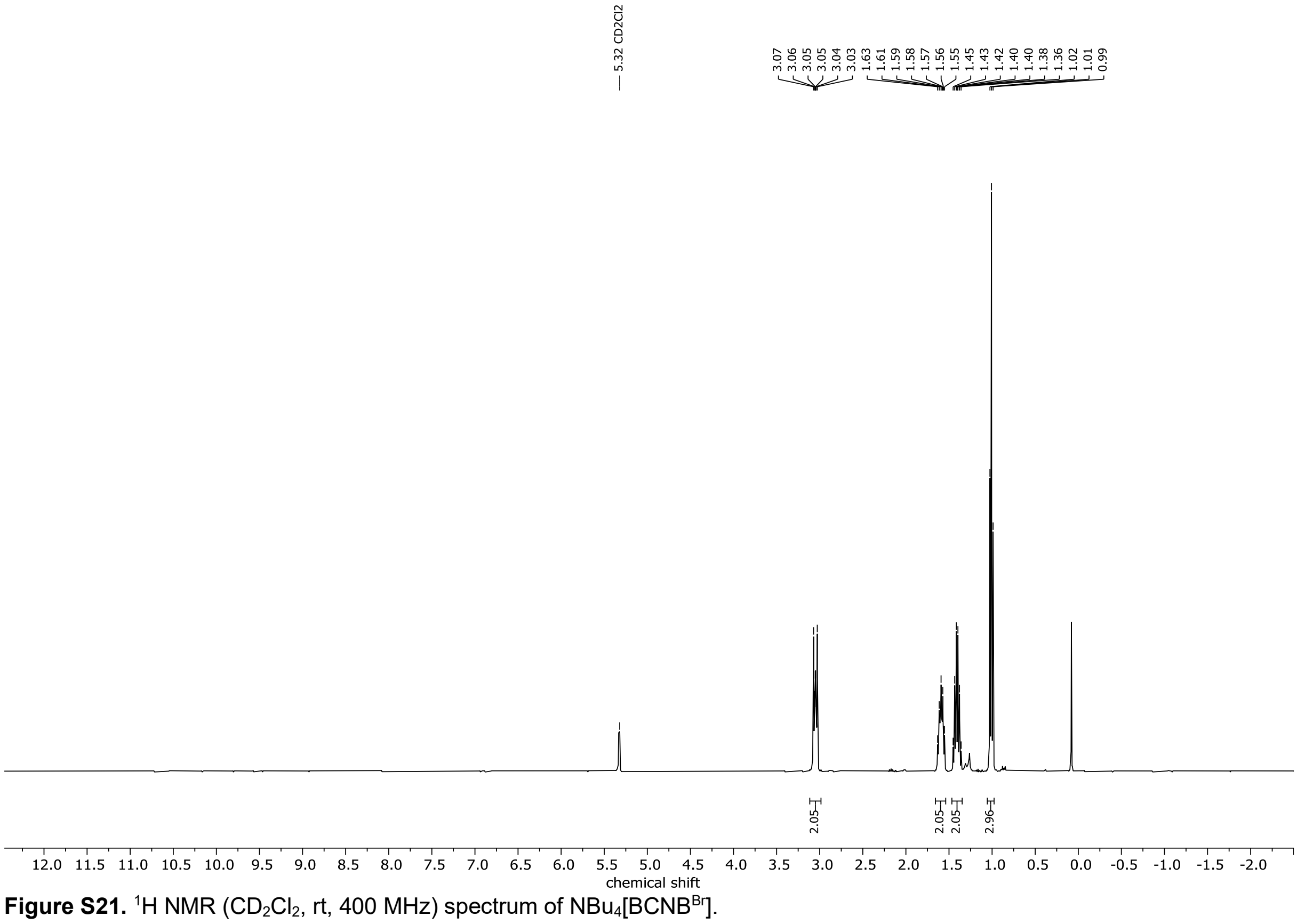

Figure S21. ^{1}H NMR (CD_2Cl_2, rt, 400 MHz) spectrum of $NBu_4[BCNB^{Br}]$.

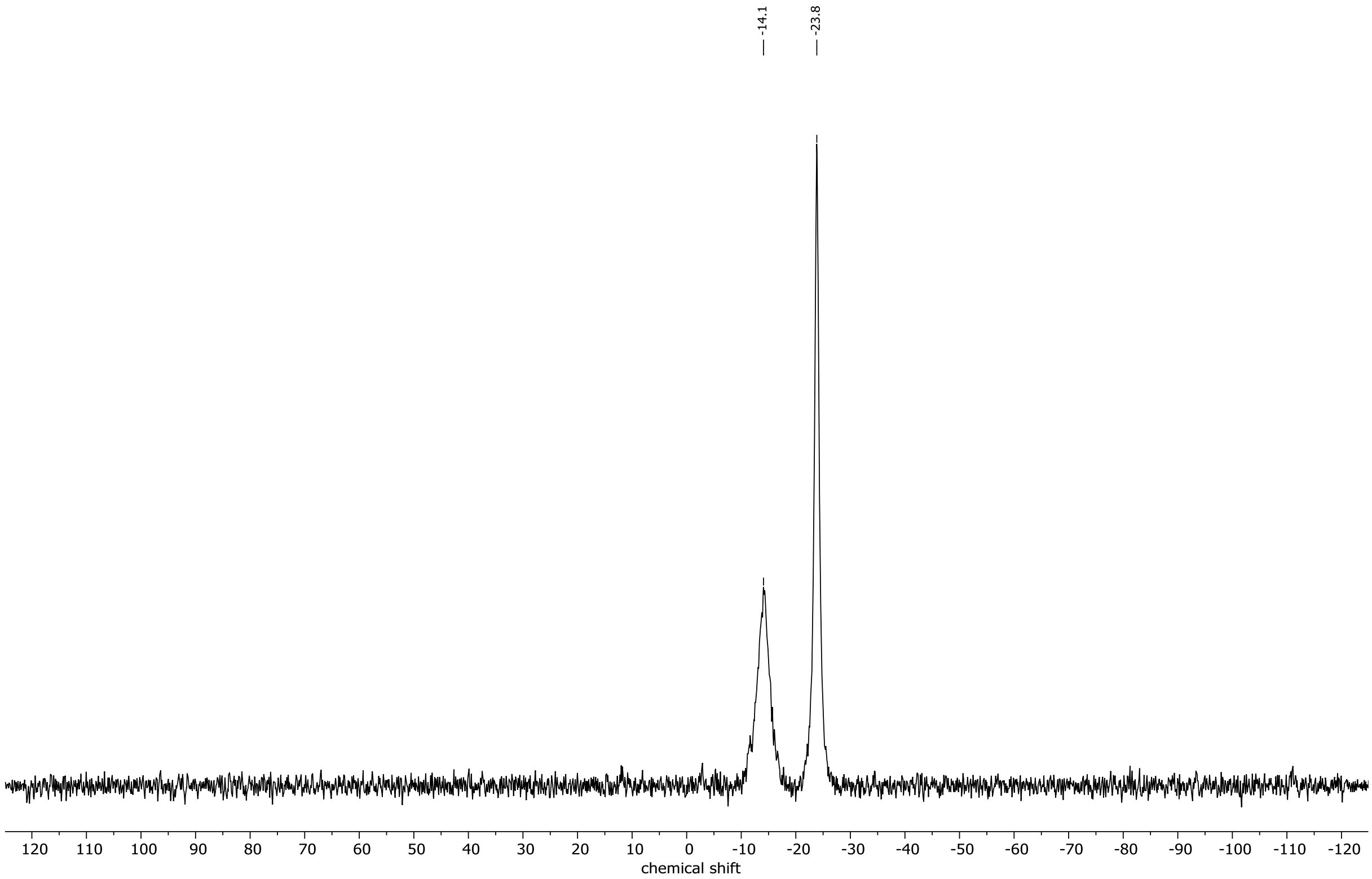

Figure S22. ^{11}B NMR (CD_2Cl_2, rt, 128.15 MHz) spectrum of $NBu_4[BCNB^{Br}]$.

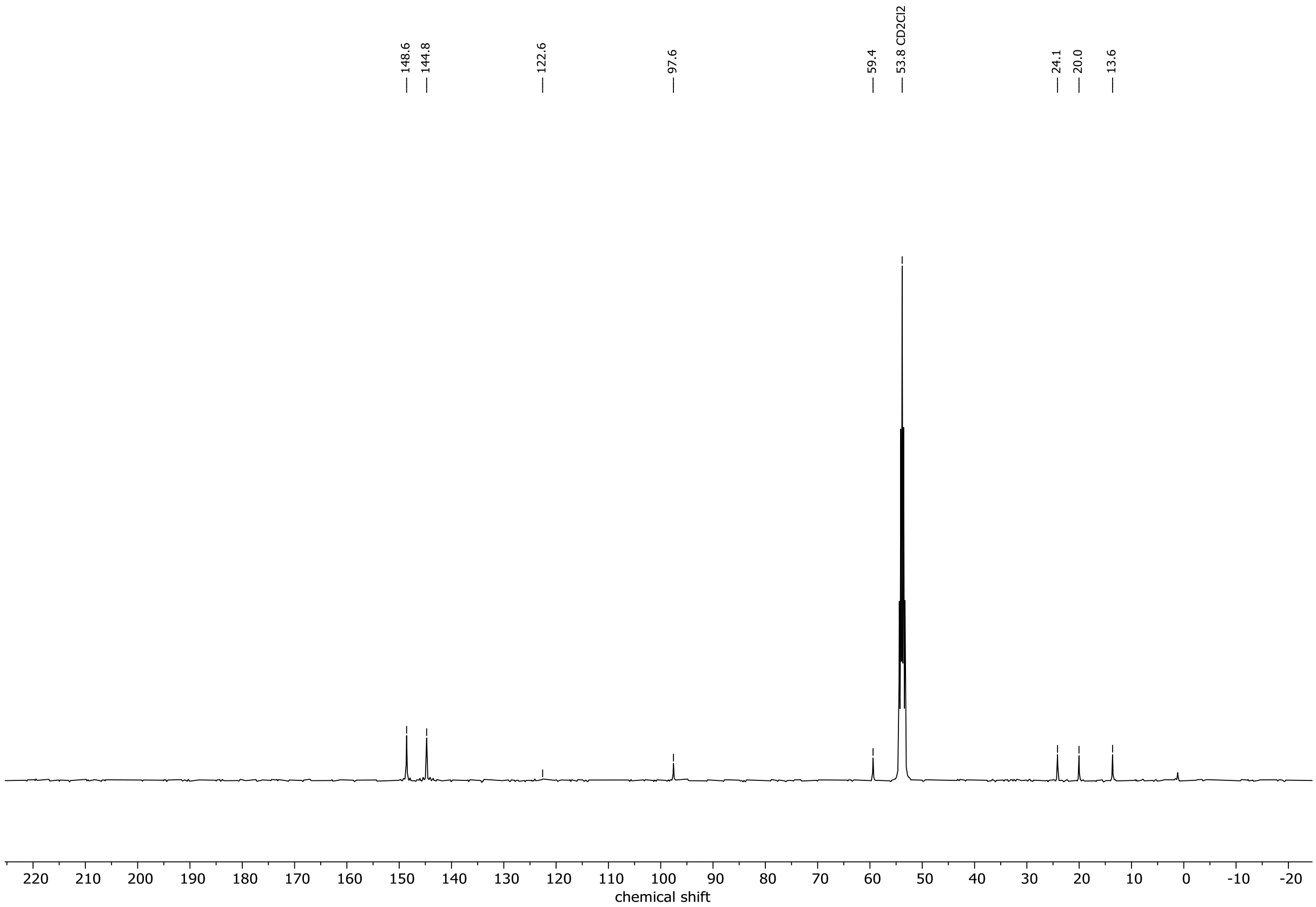

Figure S23. $^{13}C\{^1H,^{19}F\}$ NMR (CD_2Cl_2, rt, 100.42 MHz) spectrum of $NBu_4[BCNB^{Br}]$.

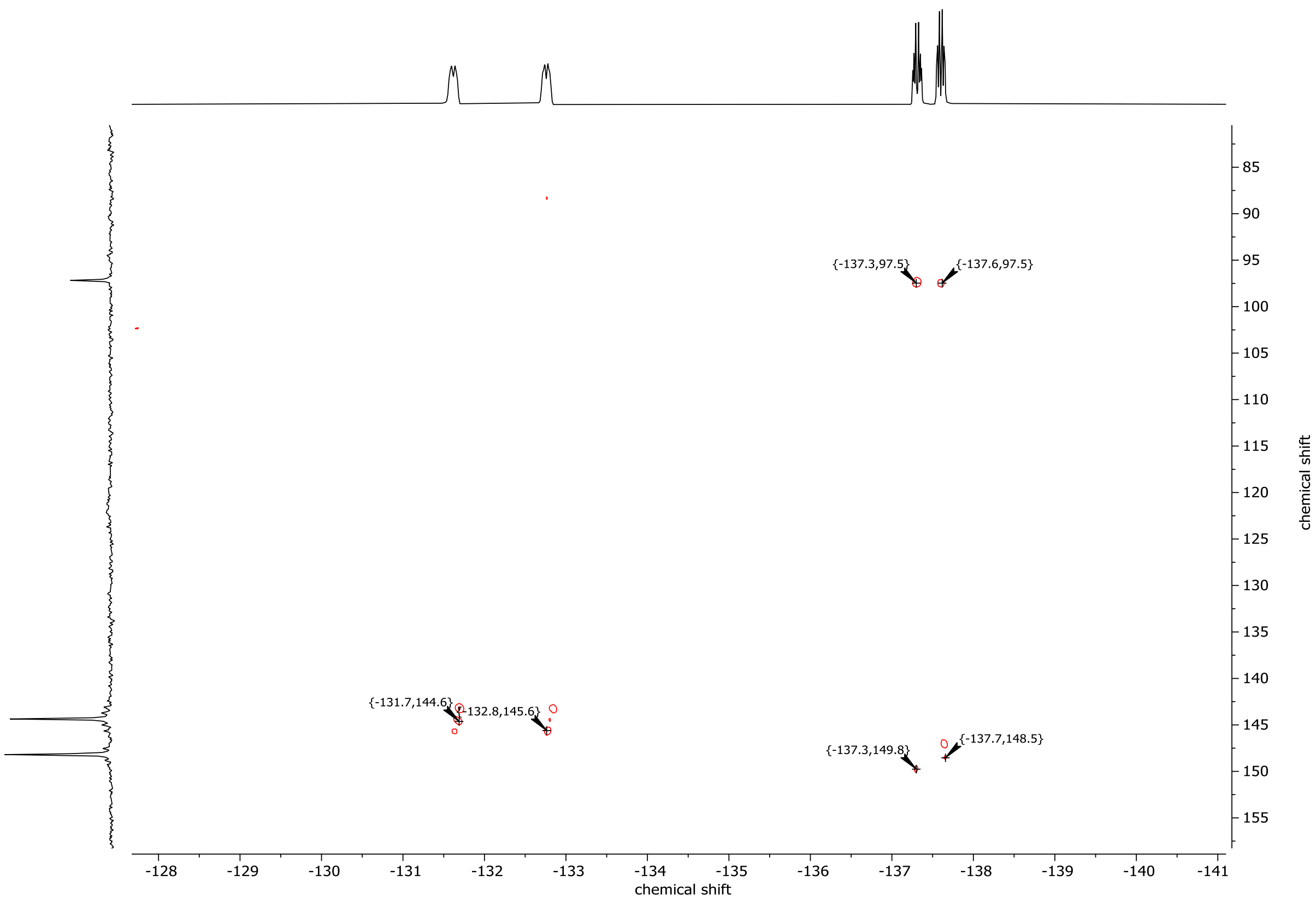

Figure S24. ^{13}C,^{19}F HMBC NMR (CD_2Cl_2, rt, 100.42 MHz, 375.79 MHz) spectrum of $NBu_4[BCNB^{Br}]$.

$Ph_3C[BCNB^{Br}]$

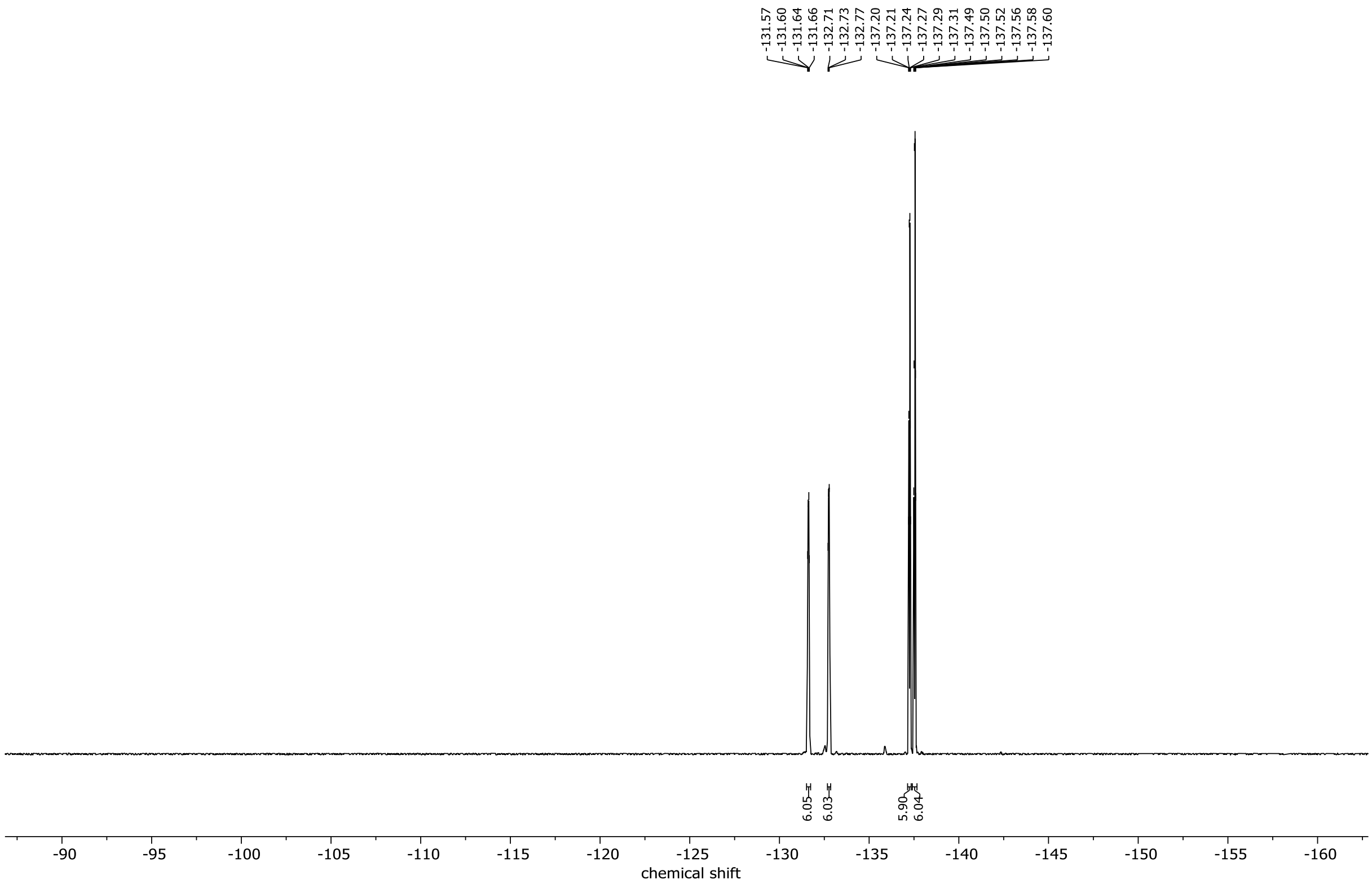

Figure S25. ^{19}F NMR (CD_2Cl_2, rt, 375.79 MHz) spectrum of $Ph_3C[BCNB^{Br}]$.

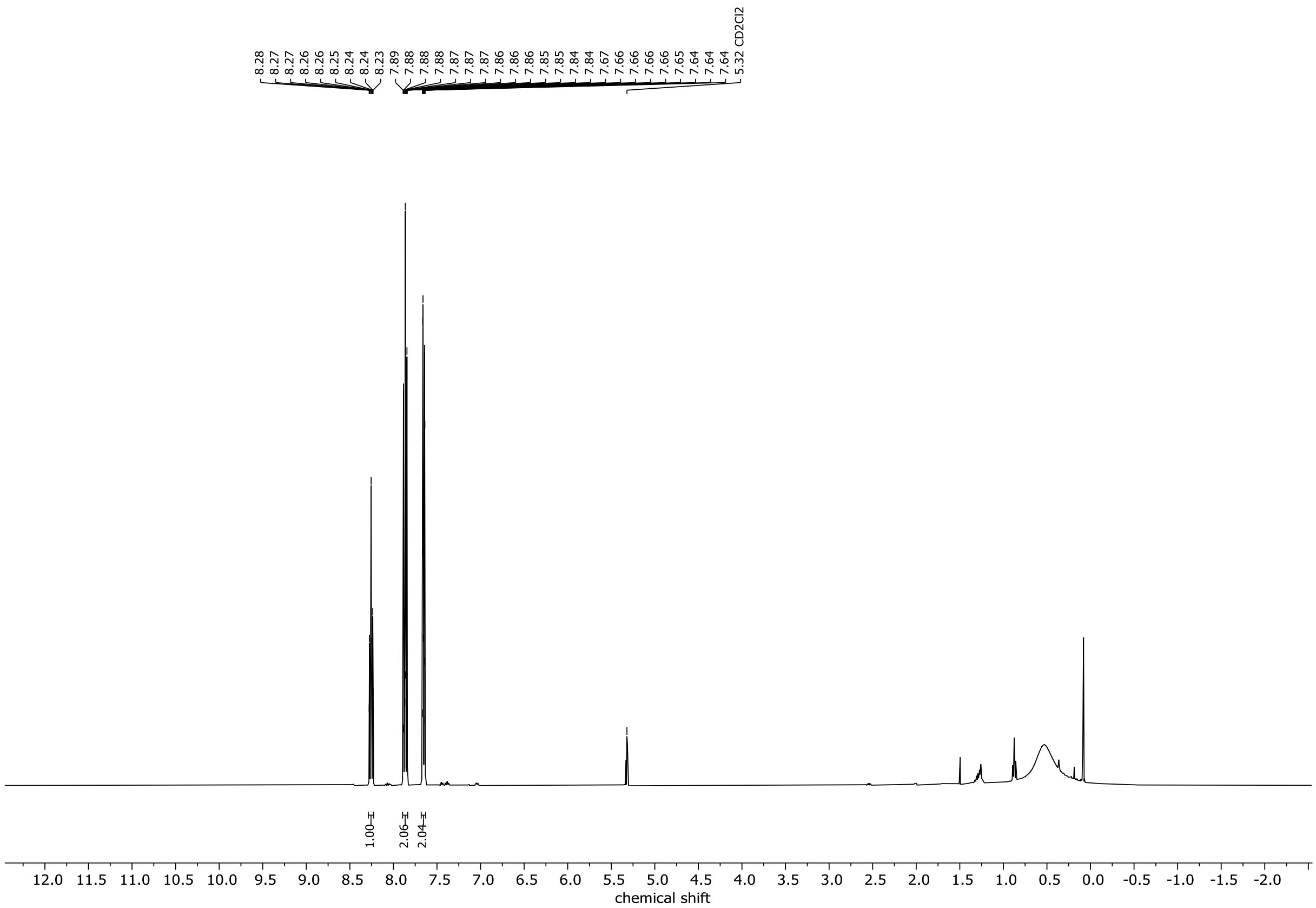

Figure S26. ^{1}H NMR (CD_2Cl_2, rt, 400 MHz) spectrum of $Ph_3C[BCNB^{Br}]$.

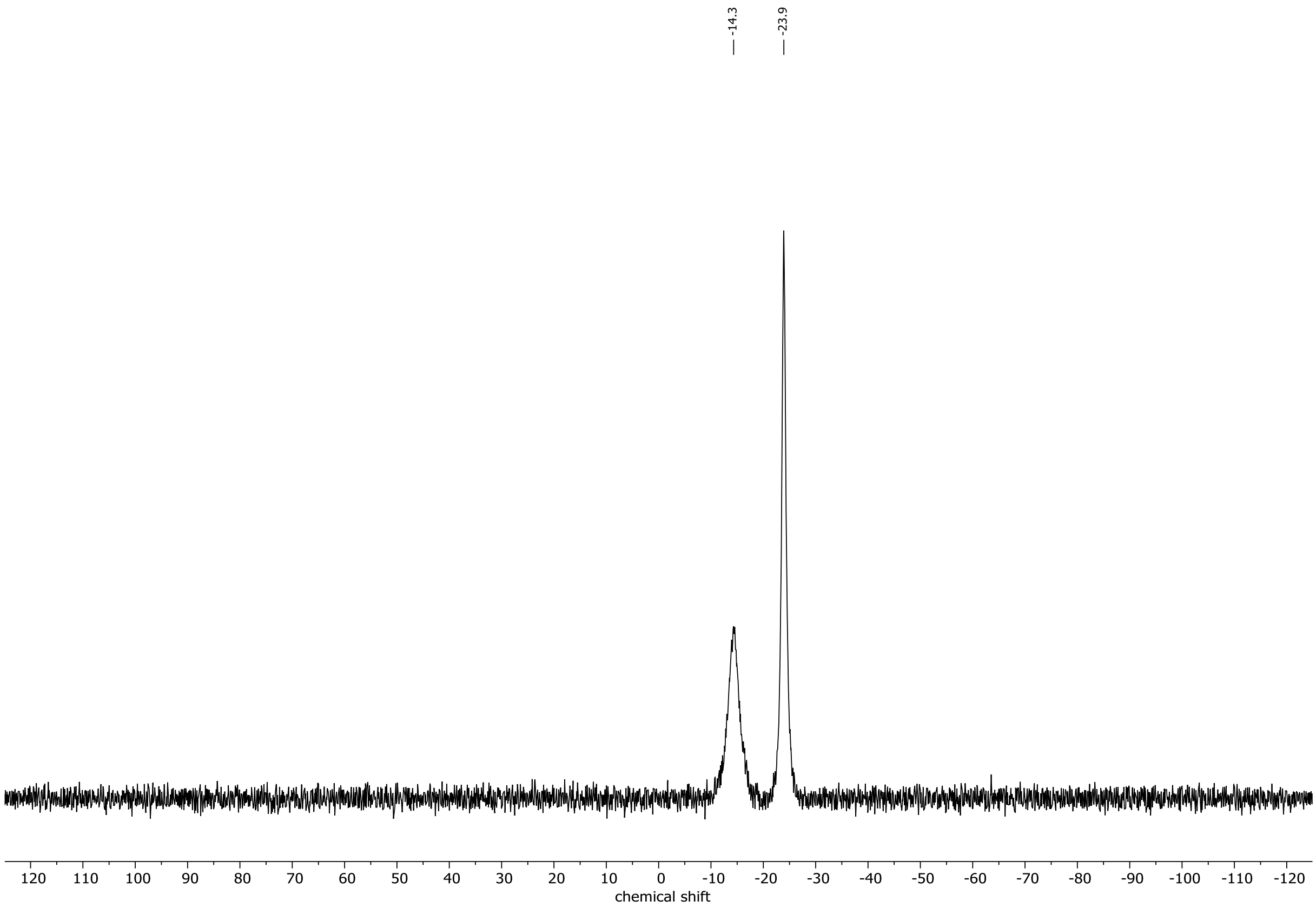

Figure S27. ^{11}B NMR (CD_2Cl_2, rt, 128.15 MHz) spectrum of $Ph_3C[BCNB^{Br}]$.

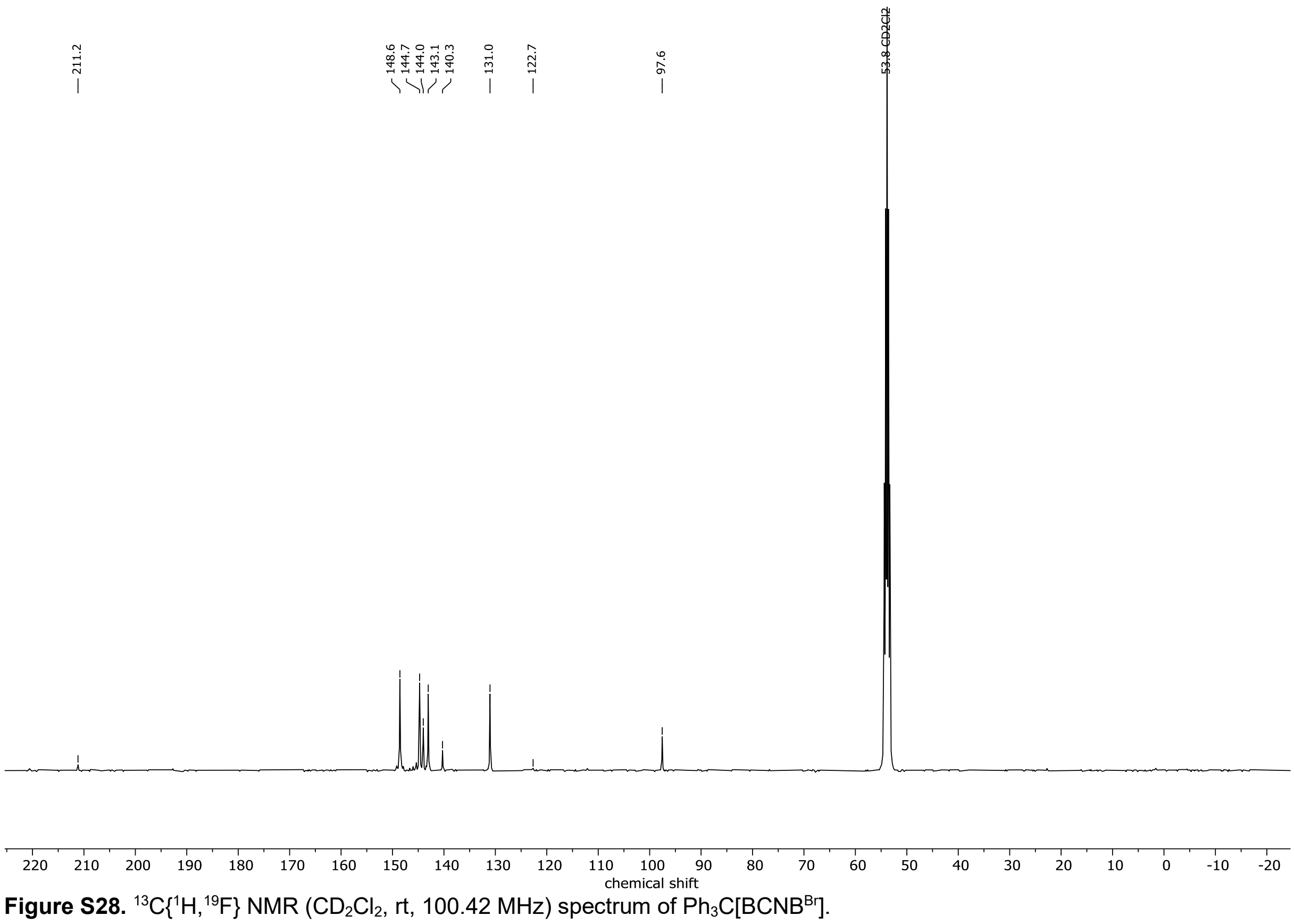

Figure S28. $^{13}C\{^1H,^{19}F\}$ NMR (CD_2Cl_2, rt, 100.42 MHz) spectrum of $Ph_3C[BCNB^{Br}]$.

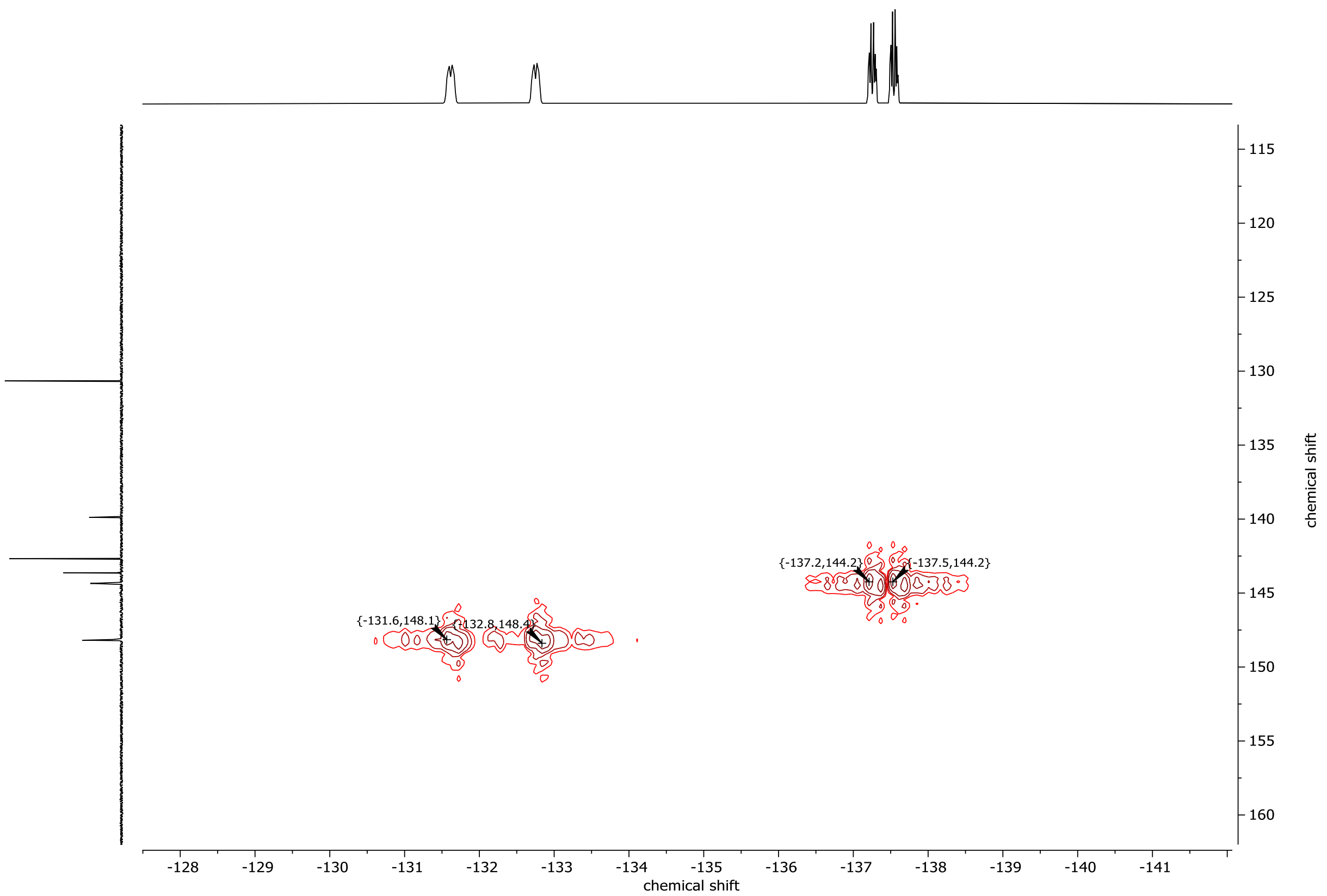

Figure S29. ^{13}C,^{19}F HMQC NMR (CD_2Cl_2, rt, 100.42 MHz, 375.79 MHz) spectrum of $Ph_3C[BCNB^{Br}]$.

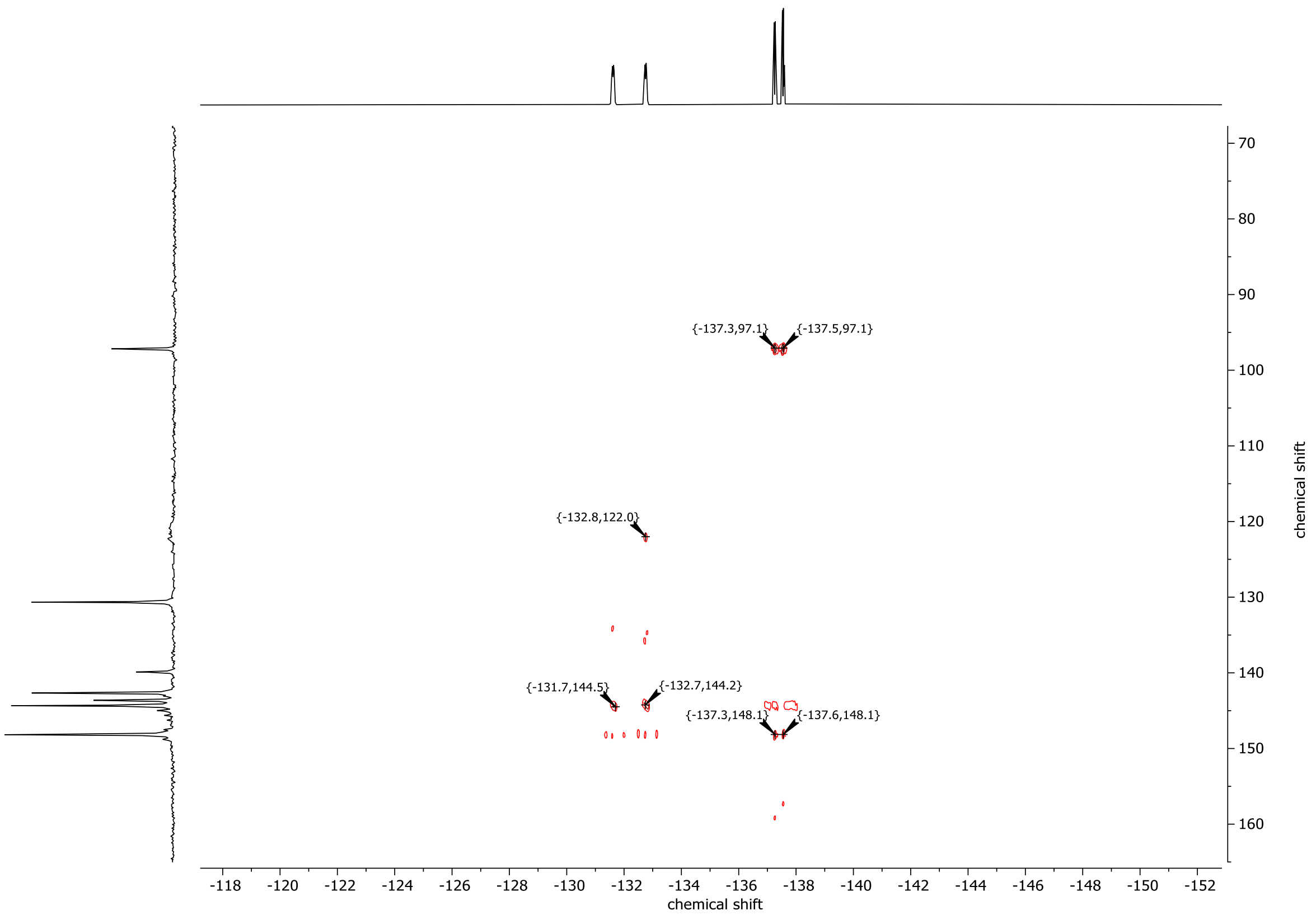

Figure S30. ^{13}C,^{19}F HMBC NMR (CD_2Cl_2, rt, 100.42 MHz, 375.79 MHz) spectrum of $Ph_3C[BCNB^{Br}]$.

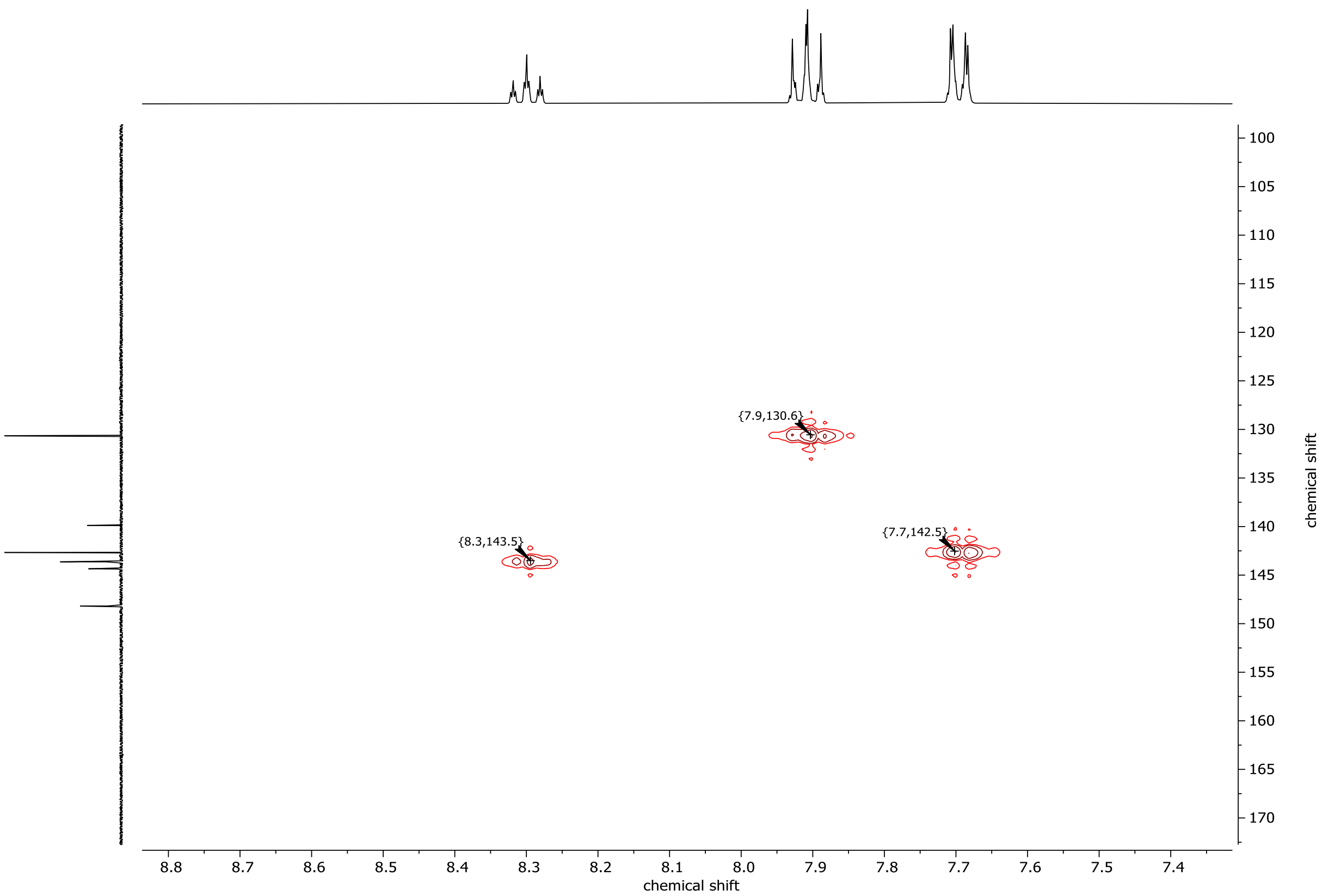

Figure S31. ^{13}C,^{1}H HMQC NMR (CD_2Cl_2, rt, 100.42 MHz, 400 MHz) spectrum of $Ph_3C[BCNB^{Br}]$.

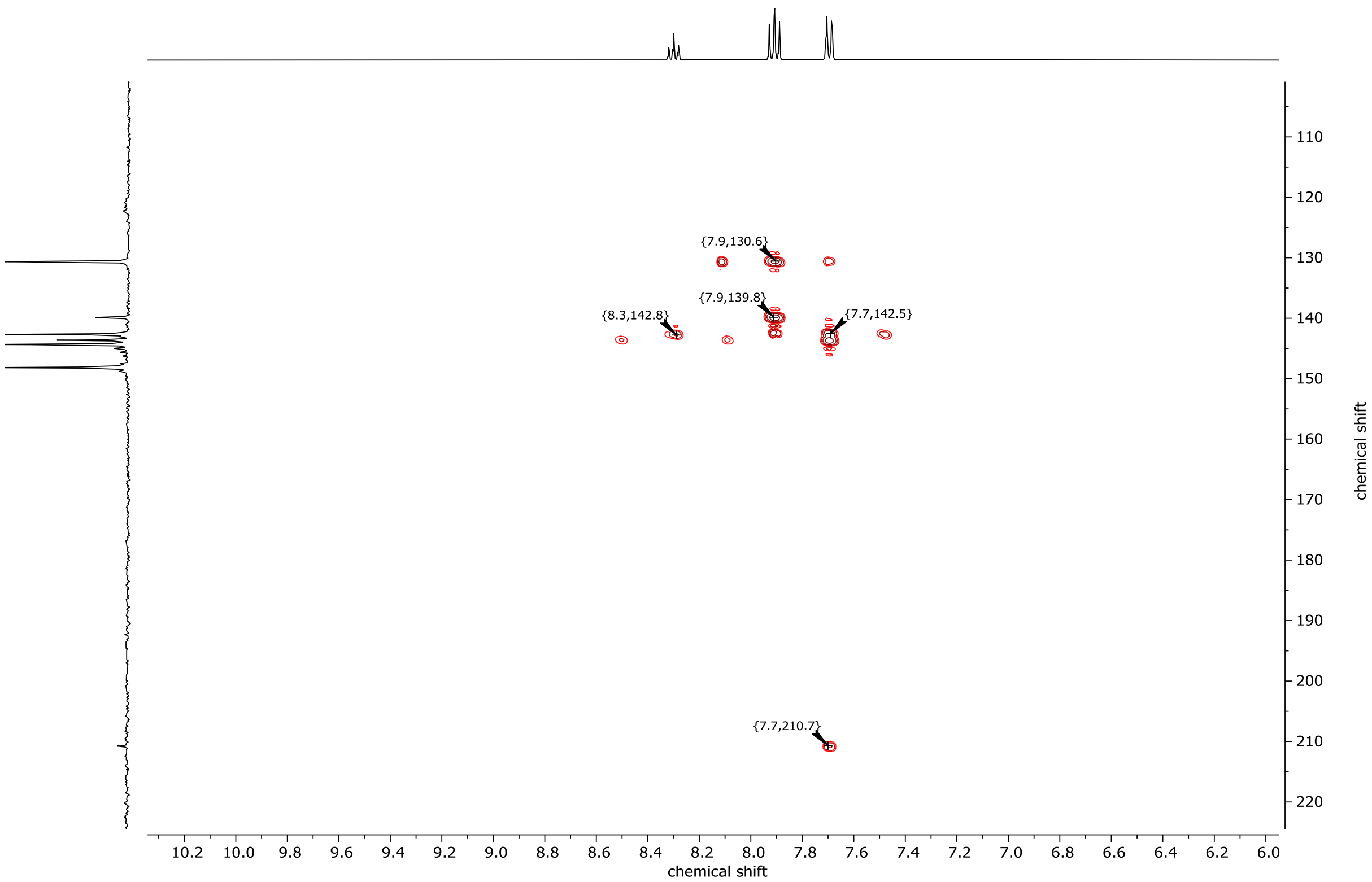

Figure S32. ^{13}C,^{1}H HMBC NMR (CD_2Cl_2, rt, 100.42 MHz, 400 MHz) spectrum of $Ph_3C[BCNB^{Br}]$.

IR Spectra

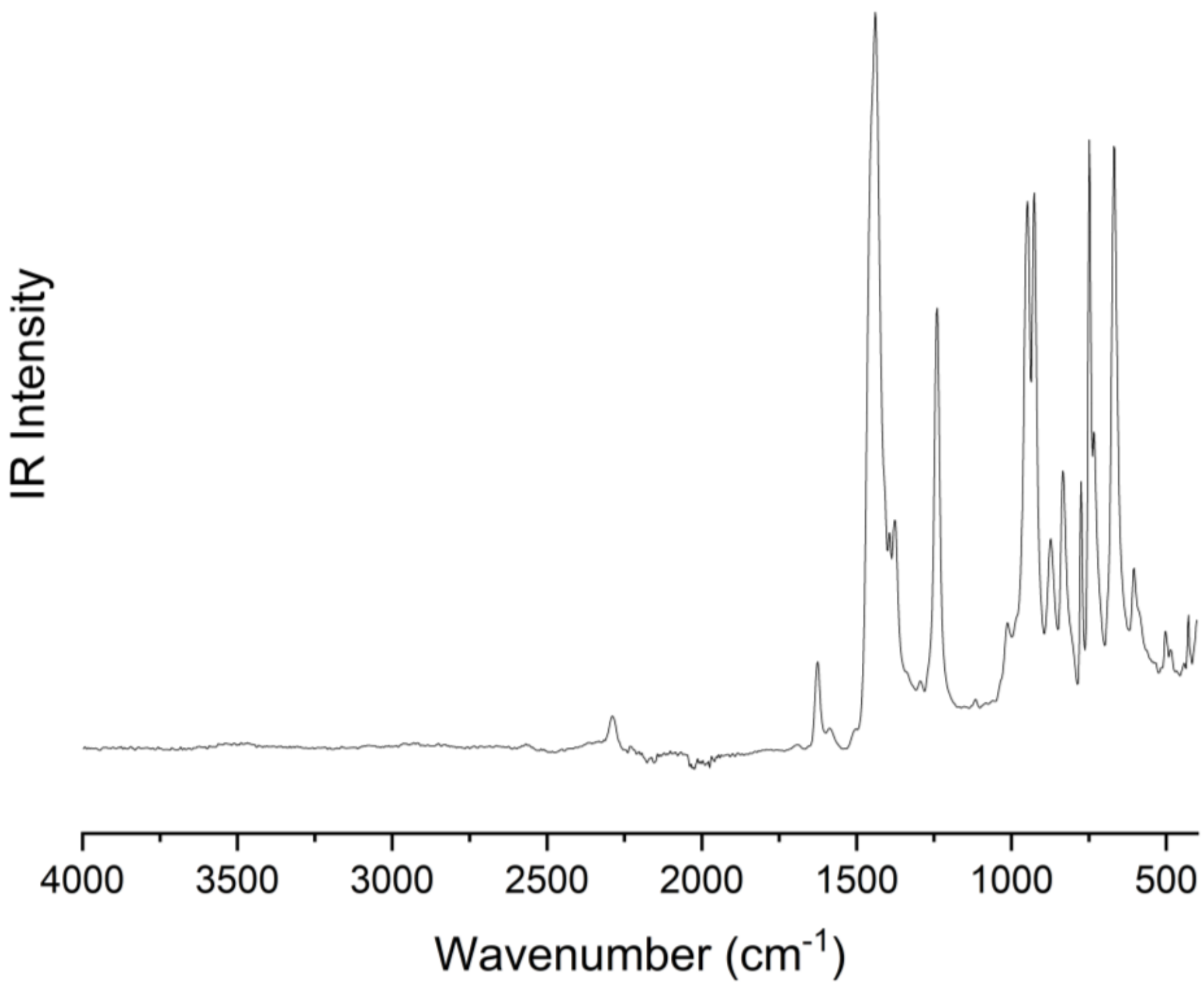

Figure S33. IR (ATR, rt) spectrum of $[Ag(CH_2Cl_2)][BCNB^{Br}]$.

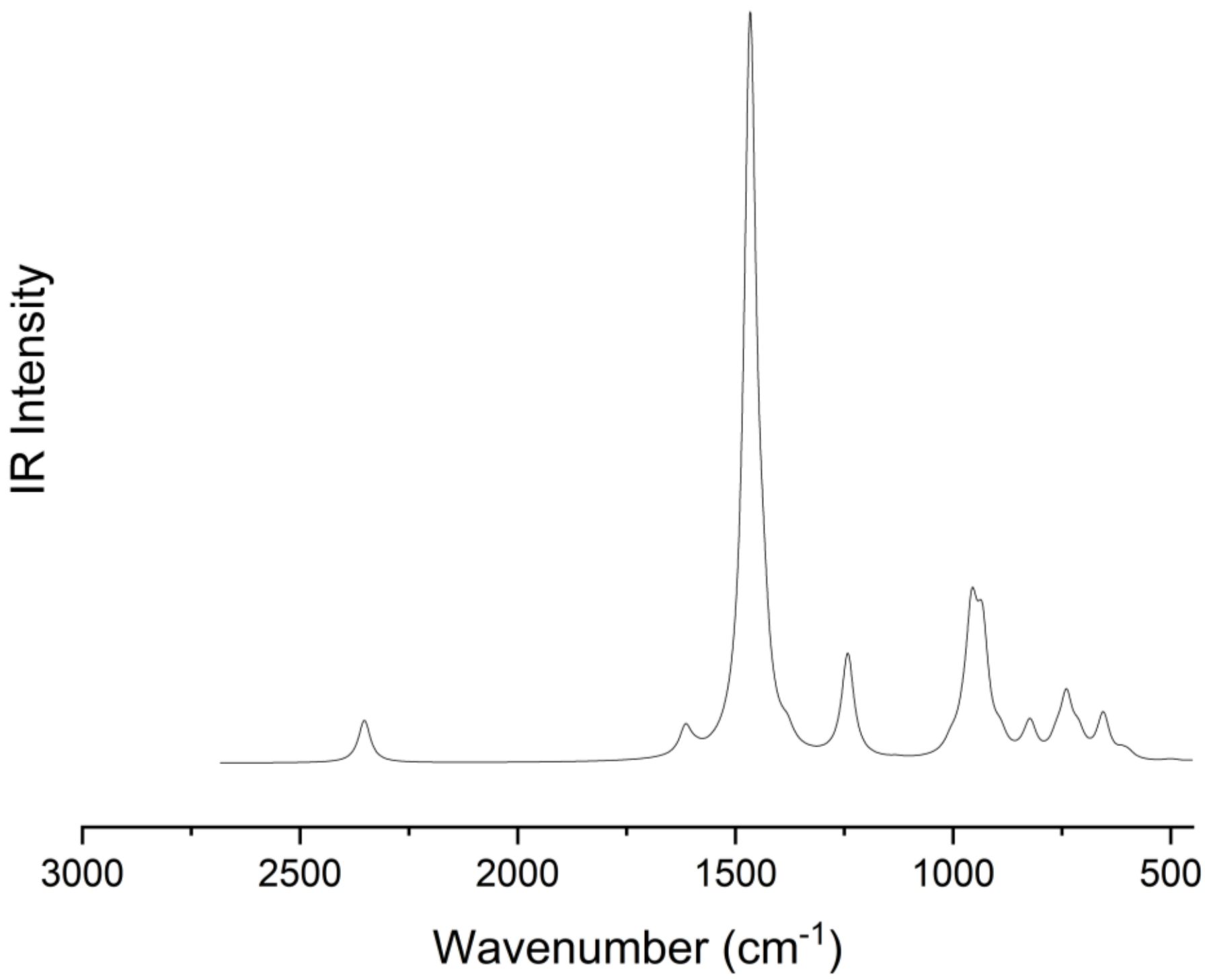

Figure S34. Calculated IR spectrum of $[BCNB^{Br}]^{-}$.

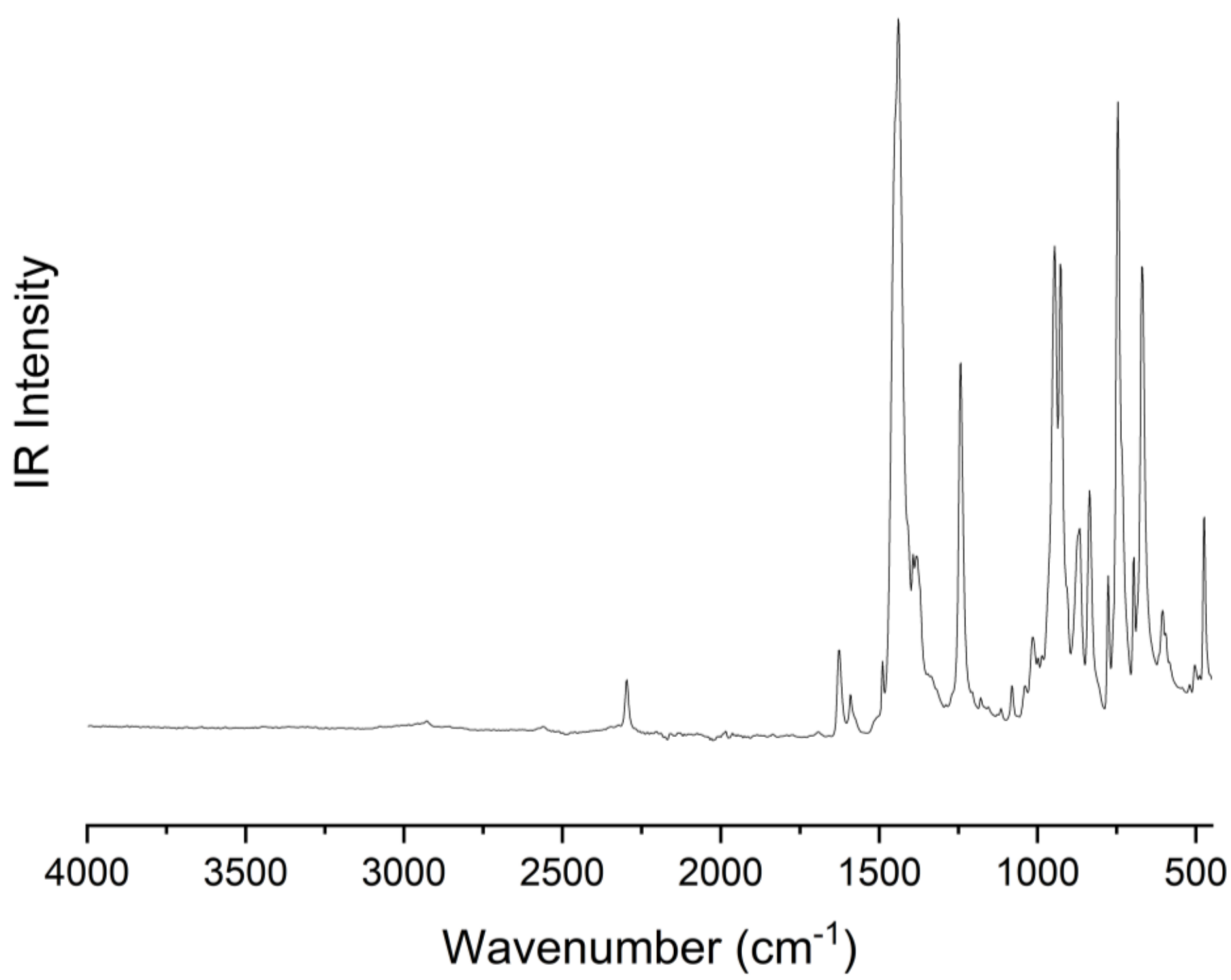

Figure S35. IR (ATR, rt) spectrum of [Ag(toluene)][BCNB^{Br}].

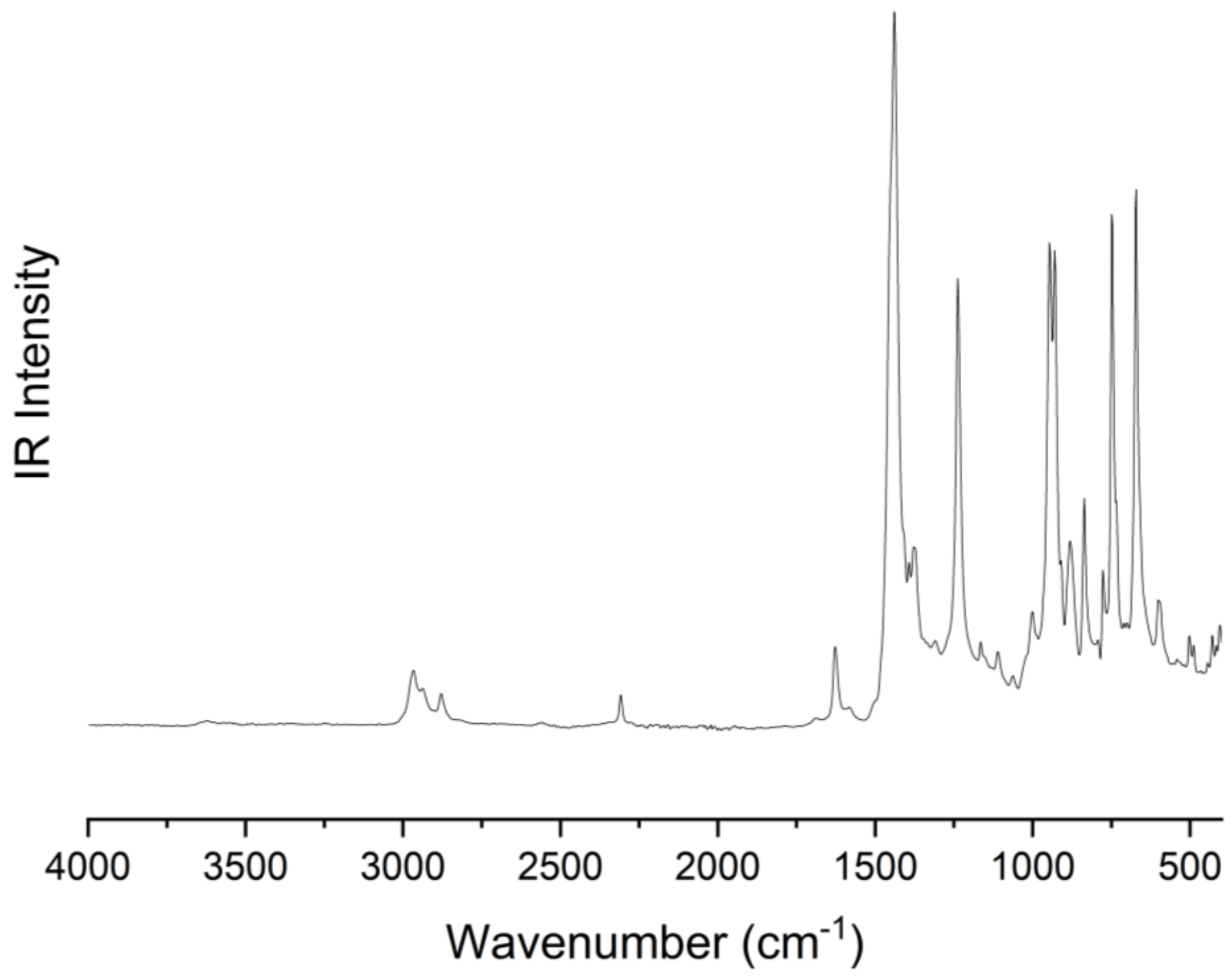

Figure S36. IR (ATR, rt) spectrum of NBu$_4$[BCNB^{Br}].

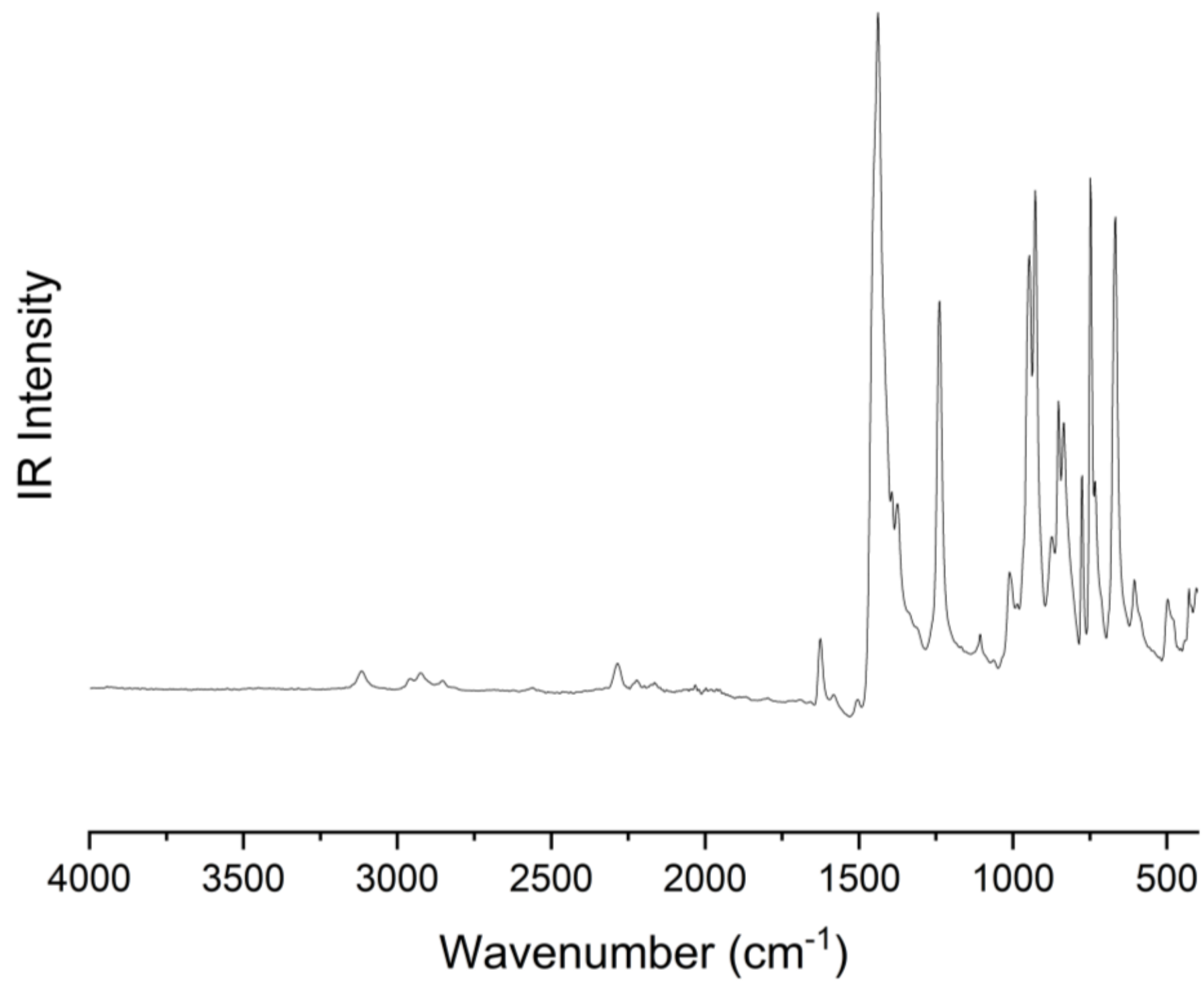

Figure S37. IR (ATR, rt) spectrum of $FeCp_2[BCNB^{Br}]$.

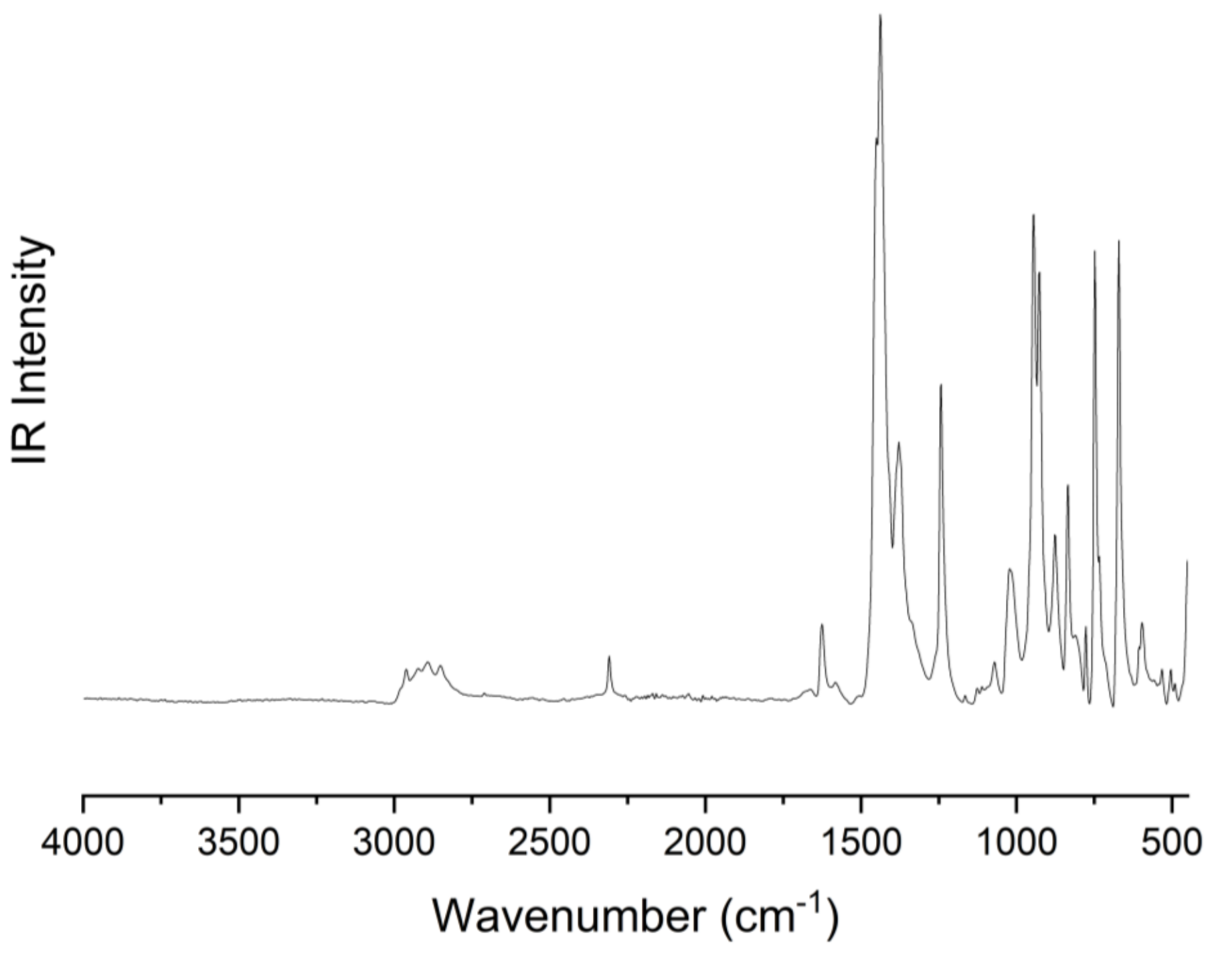

Figure S38. IR (ATR, rt) spectrum of $FeCp_2^*[BCNB^{Br}]$.

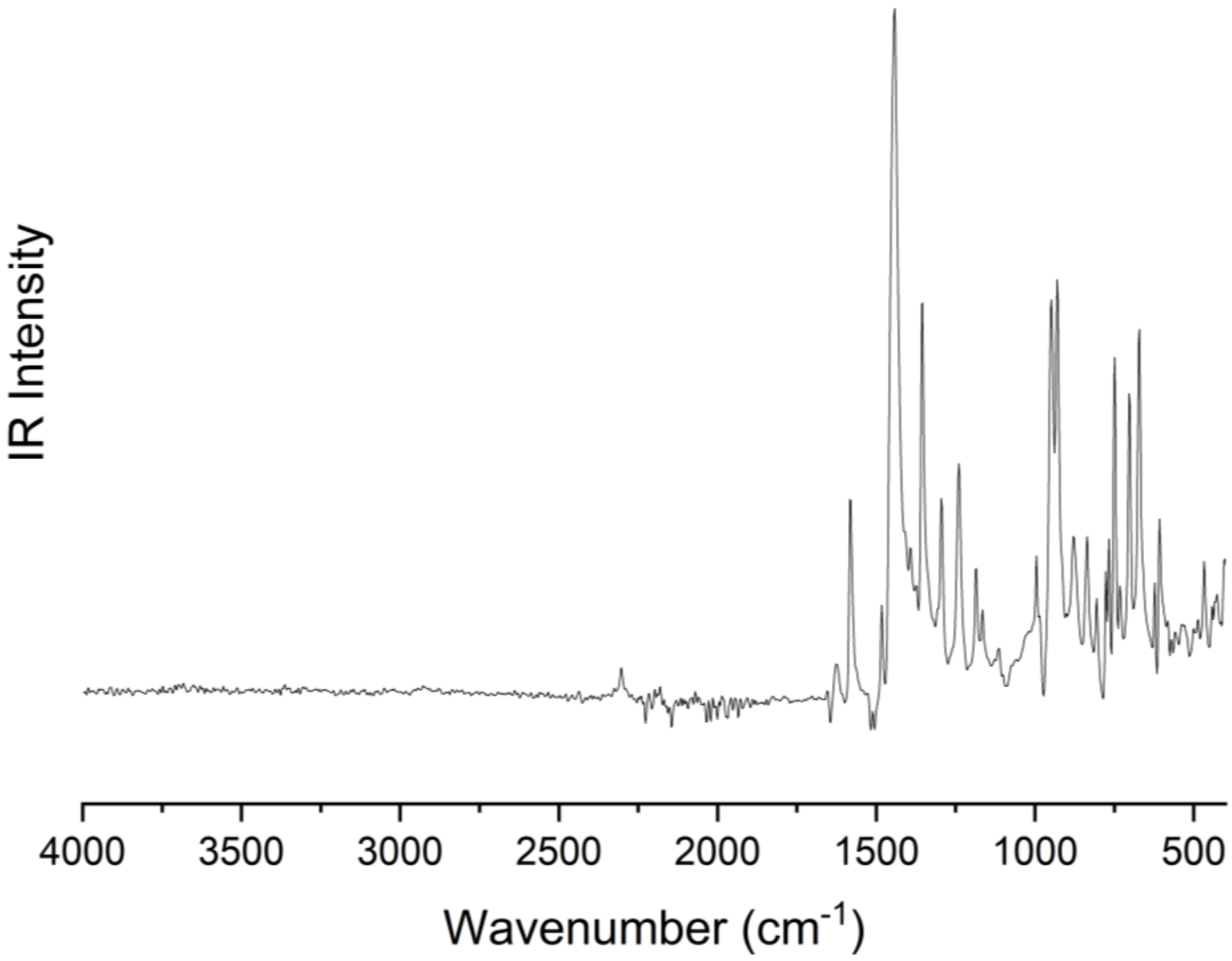

Figure S39. IR (ATR, rt) spectrum of $Ph_3C[BCNB^{Br}]$.

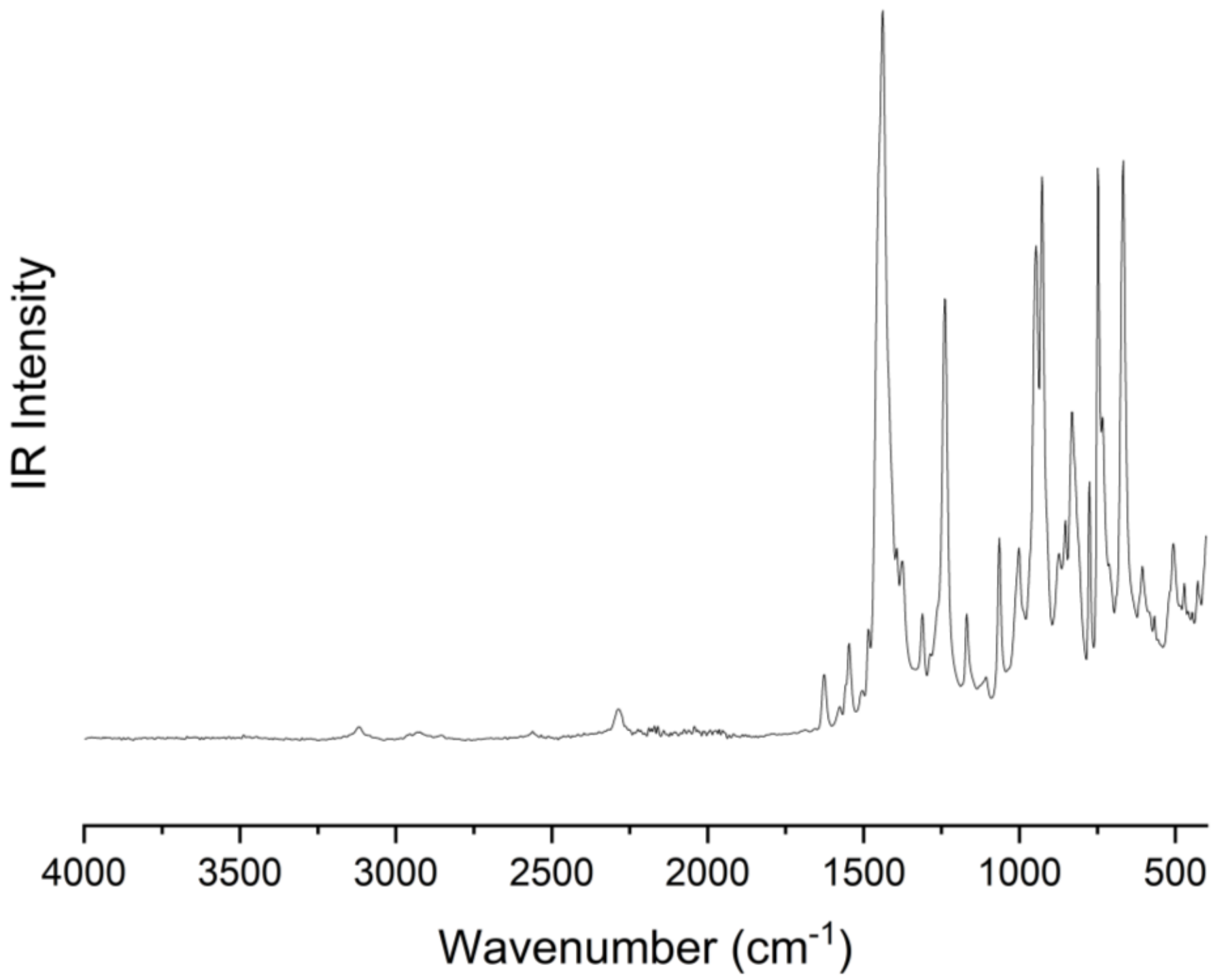

Figure S40. IR (ATR, rt) spectrum of $N(4\text{-}BrC_6H_4)_3[BCNB^{Br}]$.

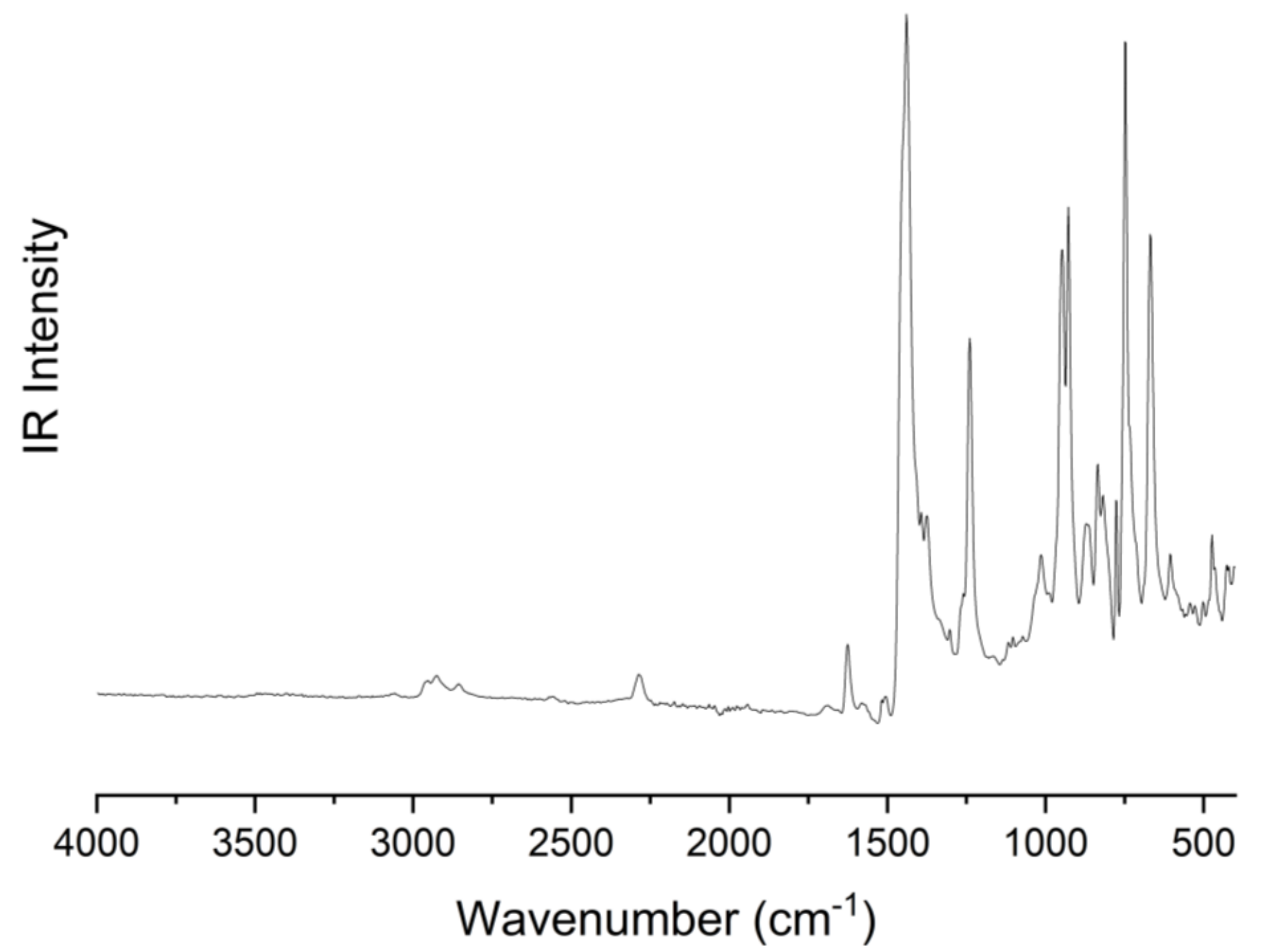

Figure S41. IR (ATR, rt) spectrum of $[C_6H_4S_2C_6H_4][BCNB^{Br}]$.

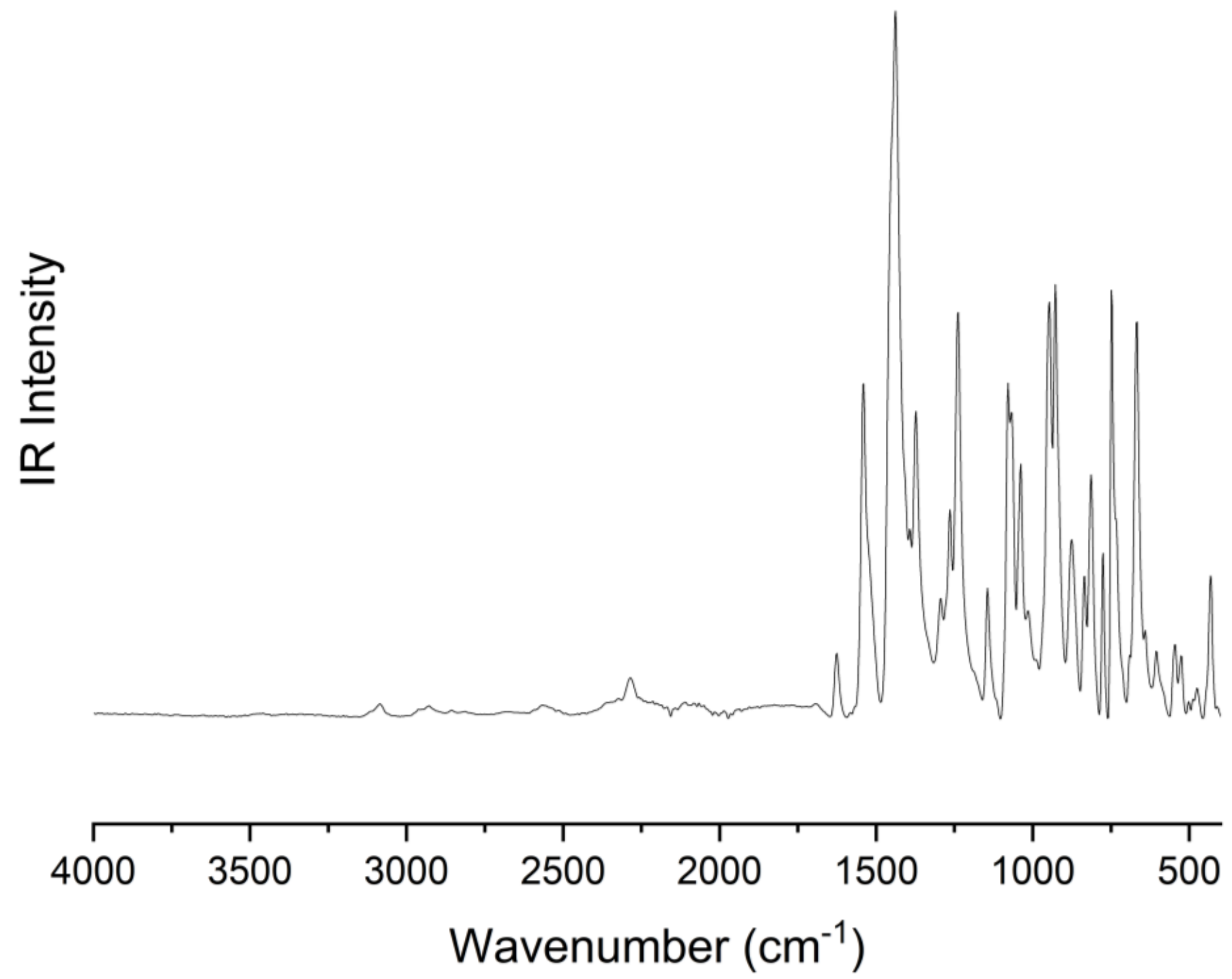

Figure S42. IR (ATR, rt) spectrum of $N(2,4\text{-}Br_2C_6H_3)_3[BCNB^{Br}]$.

Quantum Chemical Calculations

[μ-(CN)(B(C_6F_4Br)$_3$)$_2$]$^-$
anionic, diamagnetic, E = –19351.0988049 Hartree

Table S2. Structure optimized coordinates (x,y,z) for [μ-(CN)(B(C_6F_4Br)$_3$)$_2$]$^-$.

Br1	3.6190920000	0.6679540000	-5.9801110000
Br2	3.4292070000	-5.8875240000	2.5270640000
Br3	-2.9542610000	6.1503400000	-1.8092440000
Br4	-3.3755160000	-4.2231810000	-4.6022970000
Br5	-4.3527710000	-1.4073580000	5.7353800000
Br6	3.6990520000	4.7795030000	3.9309900000
F7	-4.4629860000	4.5484390000	0.3321150000
F8	4.2616490000	-1.9949350000	-0.7898530000
F9	1.0915220000	2.5949650000	0.0168640000
F10	0.9805610000	-1.5340380000	-2.0971870000
F11	-4.5317510000	-1.6676350000	0.5625410000
F12	-4.1935160000	2.0166200000	1.0583590000
F13	0.8698770000	-1.3776390000	2.4729130000
F14	4.2899850000	1.6673470000	-0.9409920000
F15	-2.0185730000	0.4139370000	4.9139560000
F16	-1.0344610000	4.0108370000	-2.8955950000
F17	-4.0449910000	0.4142710000	-2.3873890000
F18	4.8044170000	1.9141350000	3.9223370000
F19	-4.4926750000	-1.3811120000	-4.2854650000
F20	-0.7711080000	1.4571160000	-2.1782050000
F21	-1.1459890000	-2.6300790000	-0.1945130000
F22	-1.1523070000	1.0541060000	2.4740570000
F23	1.5394300000	-1.1772780000	-4.6708780000
F24	4.8534670000	2.0039370000	-3.5053280000
F25	4.2026050000	-0.1282250000	2.3506510000
F26	-1.5881200000	-4.4307560000	-2.1111010000
F27	1.6899240000	4.6372070000	1.6104470000
F28	-5.3962120000	-2.2960860000	2.9878780000
F29	1.3609840000	-3.8061980000	3.4373650000
F30	4.7549170000	-4.4078750000	0.1847660000
N31	0.5766870000	-0.0639050000	0.1418500000
C32	-0.5705240000	-0.0164040000	0.0834840000
C33	1.9184930000	-0.6239740000	-2.4088050000
C34	-2.1827060000	-3.2420920000	-2.1930690000
C35	-3.3983130000	-0.7570890000	-2.2905110000
C36	-2.5325230000	-1.0098600000	-1.2266710000
C37	2.5003970000	-1.5908500000	0.7591090000
C38	-4.3422110000	-1.4870960000	2.8748900000
C39	-3.3771560000	2.4673190000	0.0938790000
C40	-2.7477820000	4.3482440000	-1.2911270000
C41	2.2062430000	-0.4651990000	-3.7638760000
C42	-3.6553000000	-1.6928300000	-3.2955530000
C43	3.2531910000	3.3239280000	2.8163800000
C44	3.5662220000	0.9622430000	-1.8241360000
C45	1.8104000000	-2.1120050000	1.8571150000
C46	3.2018820000	0.4331100000	-4.1551640000
C47	1.9858860000	2.3783390000	0.9957190000
C48	3.7858020000	-3.6799730000	0.7410100000
C49	-2.6281600000	-0.1128600000	3.8536450000
C50	2.0634000000	-3.3758900000	2.3902690000

C51	2.5660730000	0.0893990000	-1.3963140000
C52	-2.7652570000	-0.3157800000	1.4082530000
C53	-3.8636390000	-1.1523880000	1.6054160000
C54	-3.5413520000	3.8041140000	-0.2800410000
C55	-2.4330080000	1.6282750000	-0.4975540000
C56	-1.9553030000	-2.2814990000	-1.2089290000
C57	3.8779980000	2.0867300000	2.9788290000
C58	-1.6716420000	2.2048500000	-1.5178200000
C59	3.8874630000	1.1473870000	-3.1714550000
C60	3.0607890000	-4.1748080000	1.8261850000
C61	-3.7209100000	-0.9689810000	4.0125470000
C62	3.5007110000	-2.4093260000	0.2348420000
C63	-3.0419900000	-2.9464100000	-3.2540830000
C64	2.2969860000	3.4680590000	1.8079640000
C65	-1.8020860000	3.5329410000	-1.9183970000
C66	2.5753320000	1.1189280000	1.1393650000
C67	-2.1810150000	0.1953220000	2.5697180000
C68	3.5367260000	1.0183590000	2.1447210000
B69	-2.1597220000	0.0740470000	-0.0588850000
B70	2.1190320000	-0.1153120000	0.1660770000

Table S3. Calculated IR frequencies for [μ-(CN)(B(C_6F_4Br)$_3$)$_2$]$^-$.

Frequencies in cm^{-1}	Intensities
597.54952367	10.6836
601.10729115	0.1934
602.54304781	1.7495
611.82111508	22.8122
650.68634962	0.4052
651.1348906	3.3756
651.29658972	0.42
651.5613817	5.5858
652.3137855	0.4513
652.84462669	0.5393
654.97572825	75.2882
655.37146557	82.9111
660.98683501	0.6588
662.01602283	4.823
667.08836562	1.0382
689.34336753	2.5738
701.02690263	0.192
711.86557917	38.9457
712.40783214	38.0994
716.15370057	3.6052
716.76916301	1.1379
724.40886617	0.2245
739.20240145	58.3927
739.65626148	69.8015
740.24087343	43.6001
740.56291773	55.7247
760.89048934	0.1275
762.58291434	58.3458
821.91962497	39.8874
822.71216342	39.4486
824.6838869	25.3085
825.17672106	25.3212

838.3538454	9.6034
891.56687883	64.2897
923.9944188	9.1505
932.8017985	429.3467
954.7636724	16.44
955.22681659	3.7051
957.12484705	250.1498
957.57425842	230.0768
968.46196693	18.4286
979.69241568	41.1537
999.54668855	4.221
1003.724077	2.9471
1003.79718976	19.2716
1006.79297543	18.0332
1129.08044939	3.0492
1129.86699182	0.3729
1129.99948452	0.2979
1131.32499178	0.4205
1131.81782594	0.5318
1132.22236387	0.2071
1237.42205122	23.7635
1239.64821871	16.0115
1240.74742457	62.4754
1241.53212951	54.3411
1242.63578403	133.4974
1243.38741415	120.8792
1295.65655798	0.7676
1296.48478242	0.2762
1296.91872019	0.1517
1297.19115226	0.1201
1297.59162837	0.052
1298.22372493	0.0285
1380.13738506	14.9511
1380.65362304	20.6186
1381.2924893	4.63
1382.13560708	10.3195
1384.5535505	3.1106
1385.25837298	3.4072
1436.29136959	21.1302
1436.54465308	8.9289
1437.19115943	77.615
1437.32181464	120.2548
1437.33274287	76.4958
1439.01017782	1.848
1463.83012235	207.4643
1464.21144988	123.883
1464.7058314	1049.6822
1467.05665808	229.4276
1467.9755965	671.7321
1468.59695825	610.4673
1589.63329494	7.6518
1590.51674079	0.1583
1591.5883843	0.6062
1591.79650422	0.5721
1592.01767999	2.1155

1592.21274406	2.8847
1613.79706212	29.2005
1614.06930077	34.389
1614.41948768	12.7209
1614.85265177	18.3005
1616.37283626	7.5053
1617.16585826	8.0724
2352.36910779	166.0282

Crystallographic Data

Table S4. Crystallographic data of [Ag(CH_2Cl_2)][μ-(CN)(B(C_6F_4Br)$_3$)$_2$]•CH_2Cl_2.

Identification code	2539283
Empirical formula	$C_{39}H_4AgB_2Br_6Cl_4F_{24}N$
Formula weight	1693.18
Temperature/K	109.00
Crystal system	triclinic
Space group	*P*1
a/Å	10.4662(5)
b/Å	10.9193(6)
c/Å	11.8727(6)
α/°	99.218(2)
β/°	102.214(2)
γ/°	117.409(2)
Volume/Å^3	1124.20(10)
Z	1
ρ_{calc} g/cm^3	2.501
μ/mm^{-1}	6.155
F(000)	796.0
Crystal size/mm^3	0.507 × 0.511 × 1.010
Radiation	MoK_α (λ = 0.71073)
2Θ range for data collection/°	4.4 to 60.264
Index ranges	-14 ≤ h ≤ 14, -15 ≤ k ≤ 15, -16 ≤ l ≤ 16
Reflections collected	48672
Independent reflections	13051 [R_{int} = 0.0461, R_{sigma} = 0.0443]
Data/restraints/parameters	13051/3/689
Goodness-of-fit on F^2	1.064
Final R indexes [I>=2σ (I)]	R_1 = 0.0620, wR_2 = 0.1587
Final R indexes [all data]	R_1 = 0.0645, wR_2 = 0.1609
Largest diff. peak/hole / e Å^{-3}	1.76/-1.91

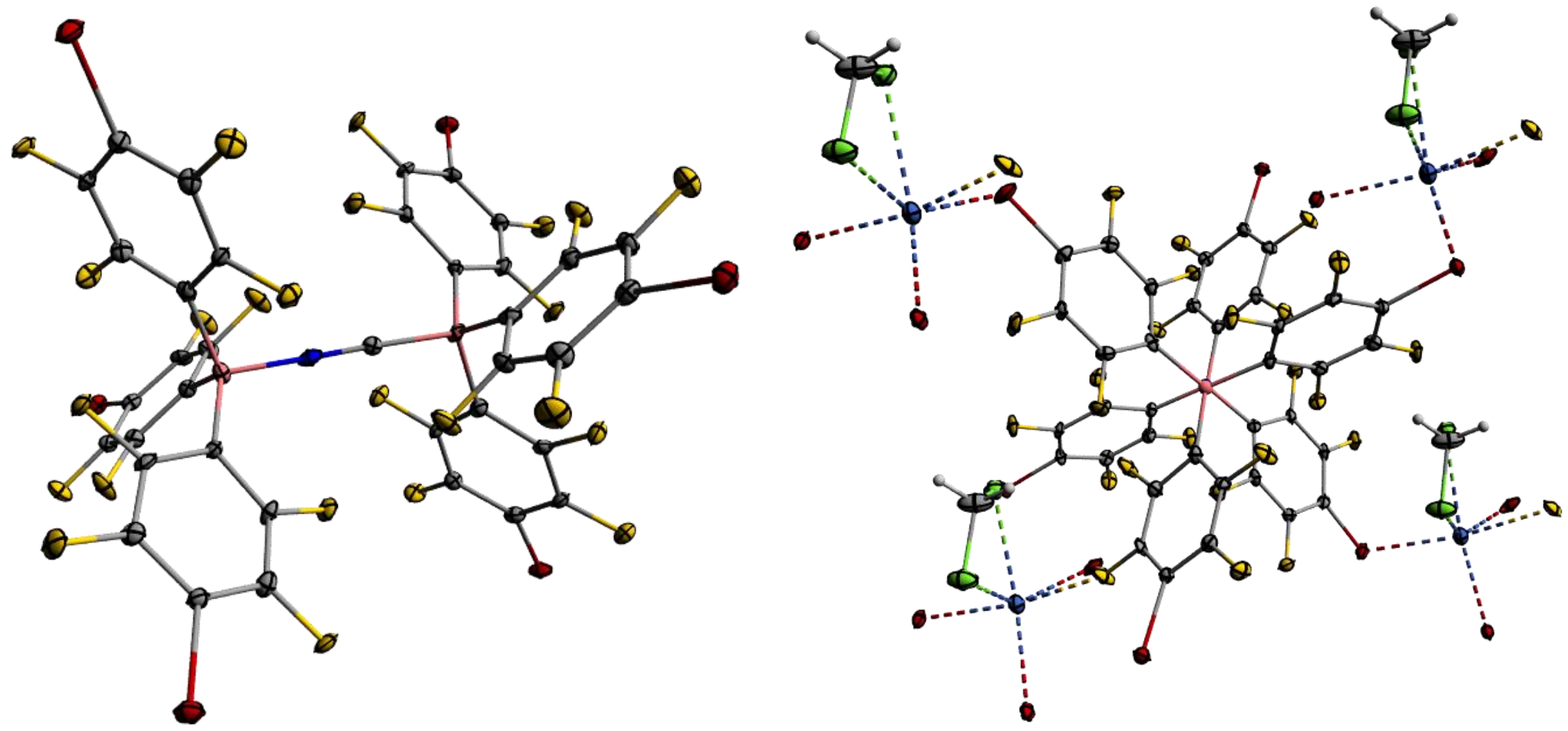

Figure S43. Molecular structure of $[Ag(CH_2Cl_2)][\mu-(CN)(B(C_6F_4Br)_3)_2]\cdot CH_2Cl_2$ in the solid state. The $[BCNB^{Br}]$ anion structure is shown (left, CH_2Cl_2 and Ag^+ are omitted), as well as the full structure with halogen-coordinated Ag^+ cations and co-crystallized CH_2Cl_2 (right). Ellipsoids are depicted with 50% probability level. Color Code: pink – boron, grey – carbon, light grey – hydrogen, green – chlorine, yellow – fluorine, red – bromine, blue – nitrogen, bright blue – silver.

Table S5. Selected structural data of $[Ag(CH_2Cl_2)][\mu-(CN)(B(C_6F_4Br)_3)_2]\cdot CH_2Cl_2$ (*d* in Å, angles in °).

	$[Ag(CH_2Cl_2)][\mu-(CN)(B(C_6F_4Br)_3)_2]\cdot CH_2Cl_2$	contacts
d(C–N)	1.148(11)	
d(B_1–CN)	1.590(12)	
d(B_2–NC)	1.592(12)	
d(B_1–C_{Ph})	1.635(19) – 1.647(17)	
d(B_2–C_{Ph})	1.625(18) – 1.653(17)	
d(C–Br)	1.867(9) – 1.882(9)	
Average B–N–C angle	177.8	
Average Torsion angle C–B–B–C	–59.2	
d(Ag–Br)	2.712(1), 2.797(1), 2.803(2)	3
d(Ag–Cl)	2.857(4), 3.017(4)	2
d(Ag–F)	2.603(6)	1
d(H–F)	2.388(7)	1
d(F–F)	2.86 – 2.92	7
d(Br–F)	3.151(8) – 3.262(6)	4
d(Br–Br)	3.677(1)	1
d(Br–Cl)	3.545(4)	1
d(F–Cl)	3.033(7) – 3.202(7)	6

Table S6. Crystallographic data of $[Ag(toluene)_3][\mu\text{-}(CN)(B(C_6F_4Br)_3)_2]$.

Identification code	2539284
Empirical formula	$C_{58}H_{24}B_2NF_{24}Br_6Ag$
Formula weight	1799.73
Temperature/K	150.00
Crystal system	monoclinic
Space group	$P2_1/n$
a/Å	13.5875(7)
b/Å	22.9153(14)
c/Å	19.0233(12)
α/°	90
β/°	91.267(2)
γ/°	90
Volume/Å^3	5921.7(6)
Z	4
ρ_{calc} g/cm^3	2.019
μ/mm^{-1}	4.506
F(000)	3448.0
Crystal size/mm^3	0.1 × 0.1 × 0.1
Radiation	MoK_α (λ = 0.71073)
2Θ range for data collection/°	4.056 to 54.256
Index ranges	-17 ≤ h ≤ 17, -29 ≤ k ≤ 29, -24 ≤ l ≤ 24
Reflections collected	131007
Independent reflections	13081 [R_{int} = 0.0957, R_{sigma} = 0.0472]
Data/restraints/parameters	13081/54/878
Goodness-of-fit on F^2	1.029
Final R indexes [I>=2σ (I)]	R_1 = 0.0393, wR_2 = 0.0740
Final R indexes [all data]	R_1 = 0.0629, wR_2 = 0.0836
Largest diff. peak/hole / e Å^{-3}	0.83/-0.99

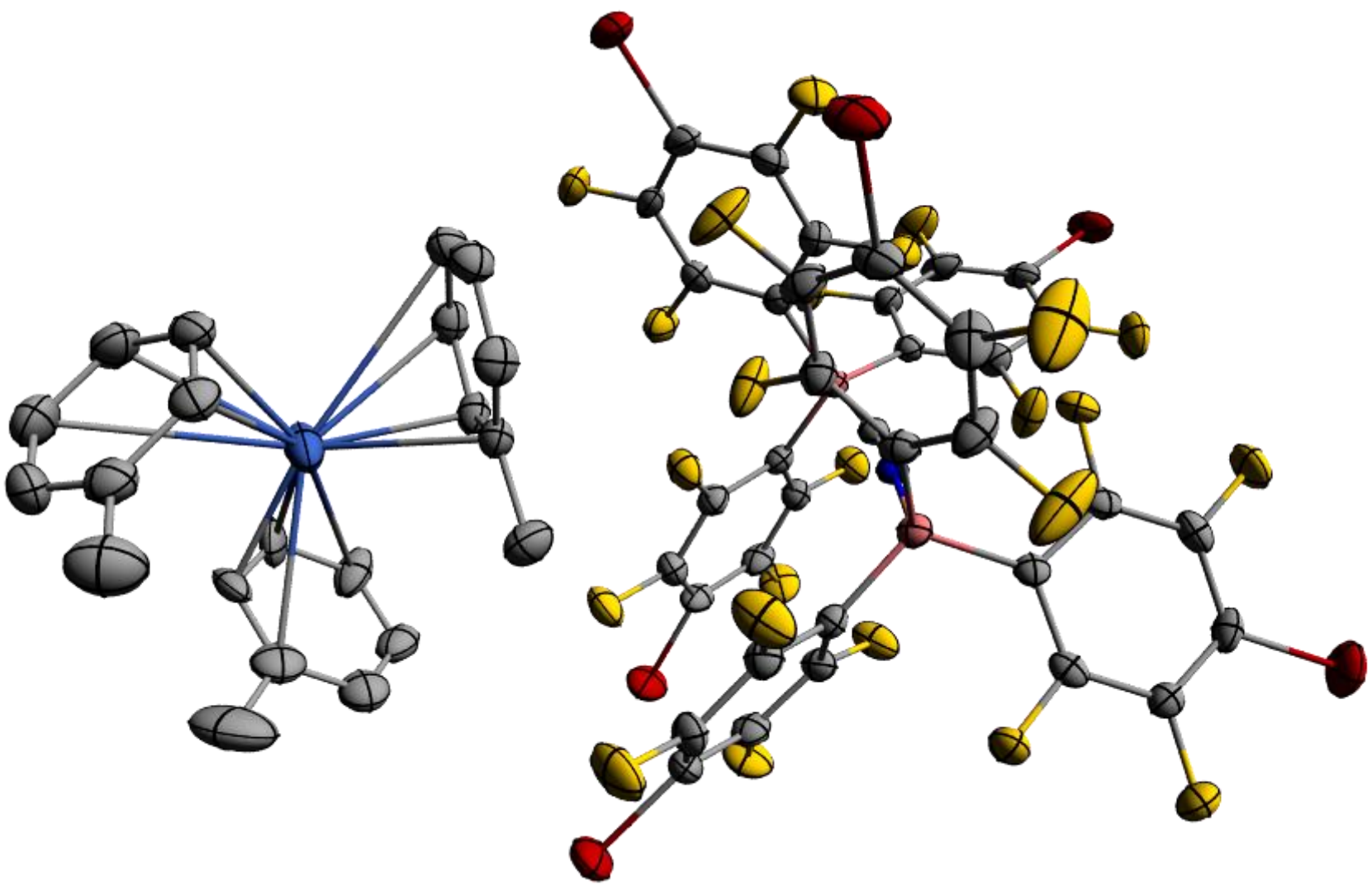

Figure S44. Molecular structure of [Ag(toluene)$_3$][μ-(CN)(B(C_6F_4Br)$_3$)$_2$] in the solid state. Disorder and hydrogen atoms are omitted. Ellipsoids are depicted with 50% probability level. Color Code: pink – boron, grey – carbon, yellow – fluorine, red – bromine, blue – nitrogen, bright blue – silver.

Table S7. Selected structural data of [Ag(toluene)$_3$][μ-(CN)(B(C_6F_4Br)$_3$)$_2$] (*d* in Å, angles in °).

	[Ag(toluene)$_3$][μ-(CN)(B(C_6F_4Br)$_3$)$_2$]	contacts
d(C–N)	1.140(4)	
d(B_1–CN)/*d*(B_2–NC)	1.597(5), 1.589(5)	
d(B_1–C_{Ph})	1.635(5) – 1.641(5)	
d(B_2–C_{Ph})	1.630(5) – 1.638(5)	
d(C–Br)	1.866(4) – 1.88	
Average B–N–C angle	173.3	
Average Torsion angle C–B–B–C	–18.1	
d(Ag–$C_{toluene}$)	2.385(14) – 3.418(4)	12
d($C_{toluene}$–C_{BCFBr})	3.366(6)	1
d(C_{BCFBr}–$H_{toluene}$)	2.735(4), 2.789(4)	2
d($C_{toluene}$–F)	3.159(22), 2.168(19)	2
d($H_{toluene}$–F)	2.302(2) – 2.571(2)	4
d(C–Br)	3.373(4) – 3.472(4)	3
d(C–F)	2.941(4) – 3.152(5)	
d(F–F)	2.697(3) – 2.933(3)	5
d(Br–F)	3.128(2), 3.304(2)	2
d(Br–Br)	3.446(7), 3.635(1)	2

Table S8. Crystallographic data of $NBu_4[\mu\text{-}(CN)(B(C_6F_4Br)_3)_2]$.

Identification code	2539285
Empirical formula	$C_{53.04}H_{34}B_2Br_6F_{24}N_{2.04}$
Formula weight	1656.94
Temperature/K	150.00
Crystal system	monoclinic
Space group	$P2_1/c$
a/Å	14.9104(12)
b/Å	17.3400(11)
c/Å	22.6165(18)
α/°	90
β/°	100.755(3)
γ/°	90
Volume/Å^3	5744.7(7)
Z	4
ρ_{calc}g/cm^3	1.916
μ/mm^{-1}	4.311
F(000)	3210.0
Crystal size/mm^3	0.1 × 0.1 × 0.1
Radiation	MoKα (λ = 0.71073)
2Θ range for data collection/°	2.98 to 50.07
Index ranges	-17 ≤ h ≤ 17, -20 ≤ k ≤ 20, -26 ≤ l ≤ 26
Reflections collected	66522
Independent reflections	10144 [R_{int} = 0.0882, R_{sigma} = 0.0528]
Data/restraints/parameters	10144/256/940
Goodness-of-fit on F^2	1.065
Final R indexes [I>=2σ (I)]	R_1 = 0.0582, wR_2 = 0.1189
Final R indexes [all data]	R_1 = 0.0767, wR_2 = 0.1265
Largest diff. peak/hole / e Å^{-3}	1.36/-1.27

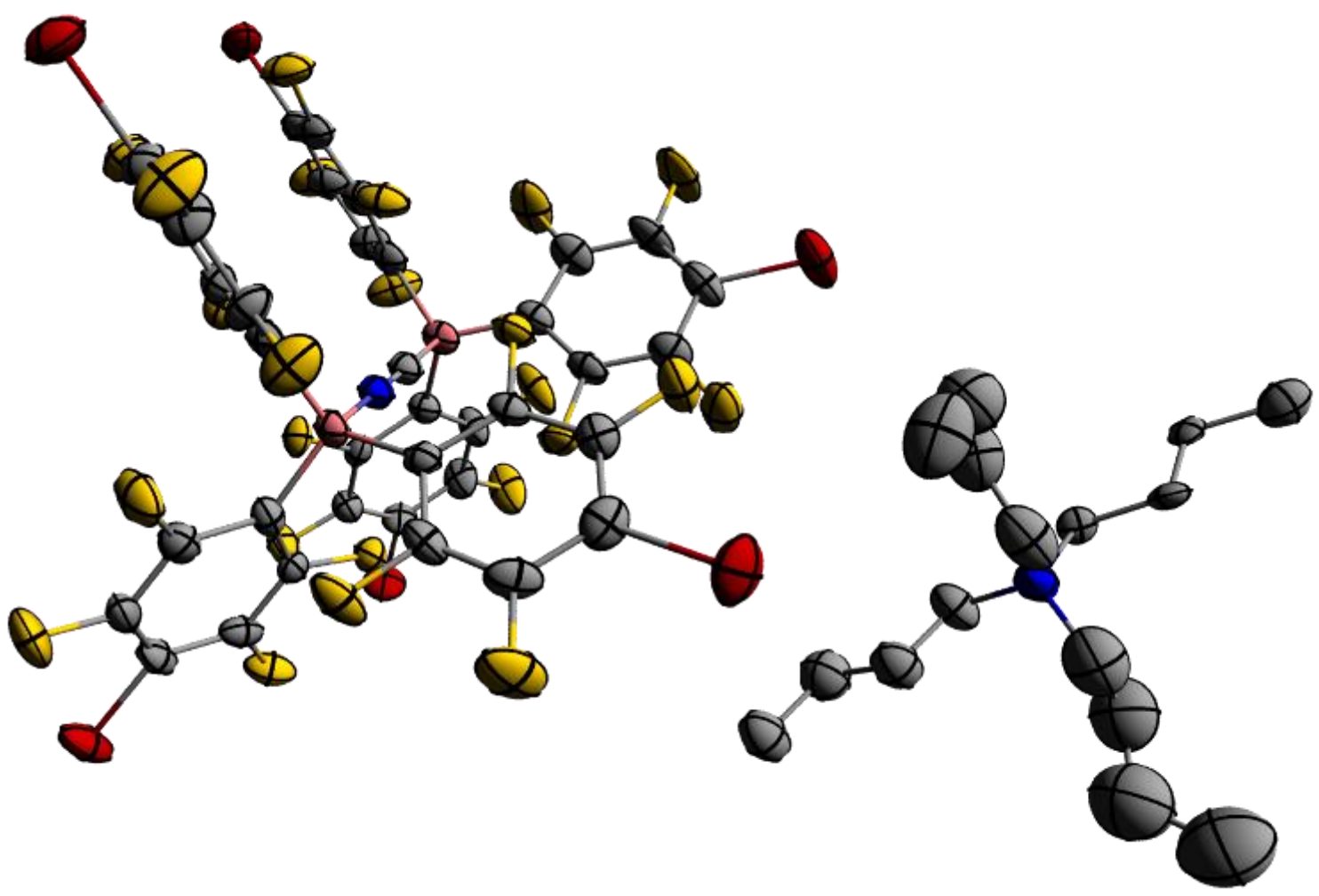

Figure S45. Molecular structure of $NBu_4[\mu\text{-}(CN)(B(C_6F_4Br)_3)_2]$ in the solid state. Disorder and hydrogen atoms are omitted. Ellipsoids are depicted with 50% probability level. Color Code: pink – boron, grey – carbon, yellow – fluorine, red – bromine, blue – nitrogen.

Table S9. Selected structural data of $NBu_4[\mu\text{-}(CN)(B(C_6F_4Br)_3)_2]$ (*d* in Å, angles in °).

	$NBu_4[\mu\text{-}(CN)(B(C_6F_4Br)_3)_2]$	contacts
d(C–N)	1.150(6)	
$d(B_1\text{–}CN)/d(B_2\text{–}NC)$	1.591(7) and 1.584(7)	
$d(B_1\text{–}C_{Ph})$	1.631(9) – 1.64(1)	
$d(B_2\text{–}C_{Ph})$	1.635(1) – 1.649(8)	
d(C–Br)	1.871(7) – 1.877(7)	
Average B–N–C angle	179.1	
Average Torsion angle C–B–B–C	–15.2	
$d(C_{TBA}\text{–}C_{BCFBr})$	3.323(28)	1
$d(C_{BCFBr}\text{–}H_{TBA})$	2.544(6), 2.667(7)	2
$d(C_{TBA}\text{–}F)$	3.109(14), 3.148(16)	2
$d(H_{TBA}\text{–}F)$	2.382(3) – 2.520(4)	4
d(F–F)	2.709(5) – 2.890(4)	3
d(Br–F)	3.049(3) – 3.288(3)	6
d(Br–H)	2.875(1)	1

Table S10. Crystallographic data of $Ph_3C[\mu\text{-}(CN)(B(C_6F_4Br)_3)_2]$.

Identification code	2539286
Empirical formula	$C_{56}H_{15}B_2Br_6F_{24}N$
Formula weight	1658.77
Temperature/K	100.00
Crystal system	triclinic
Space group	$P\bar{1}$
a/Å	13.5680(4)
b/Å	14.5595(5)
c/Å	15.3447(5)
α/°	98.1630(10)
β/°	112.8000(10)
γ/°	94.1560(10)
Volume/Å^3	2738.95(15)
Z	2
ρ_{calc} g/cm^3	2.011
μ/mm^{-1}	4.522
F(000)	1588.0
Crystal size/mm^3	0.541 × 0.496 × 0.493
Radiation	MoK_α (λ = 0.71073)
2Θ range for data collection/°	4.394 to 60.088
Index ranges	-19 ≤ h ≤ 19, -20 ≤ k ≤ 20, -21 ≤ l ≤ 21
Reflections collected	66657
Independent reflections	15997 [R_{int} = 0.0575, R_{sigma} = 0.0570]
Data/restraints/parameters	15997/0/796
Goodness-of-fit on F^2	1.056
Final R indexes [I>=2σ (I)]	R_1 = 0.0412, wR_2 = 0.0911
Final R indexes [all data]	R_1 = 0.0624, wR_2 = 0.0993
Largest diff. peak/hole / e Å^{-3}	1.34/-1.44

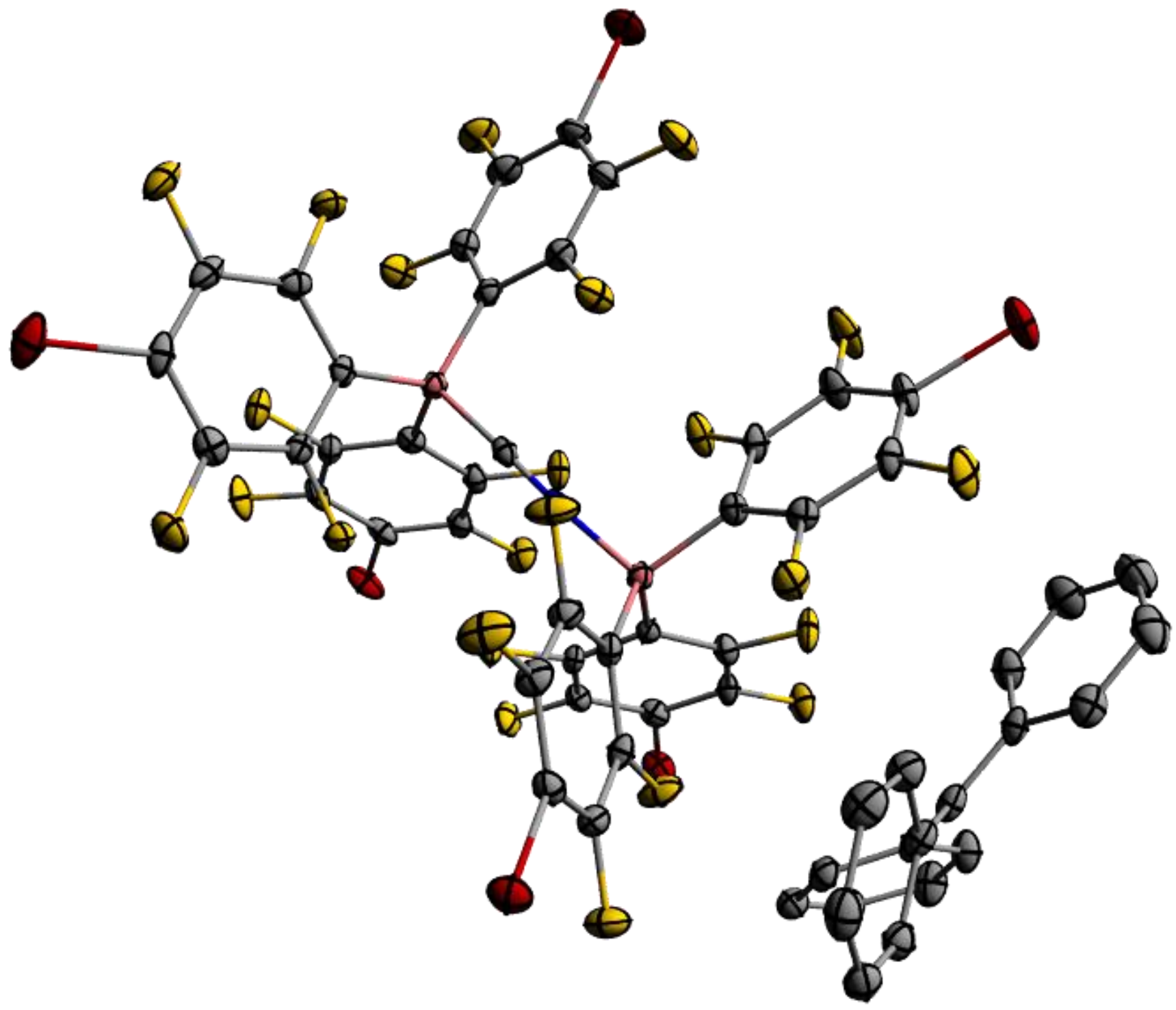

Figure S46. Molecular structure of $Ph_3C[\mu\text{-}(CN)(B(C_6F_4Br)_3)_2]$ in the solid state. Hydrogen atoms are omitted and ellipsoids are depicted with 50% probability level. Color Code: pink – boron, grey – carbon, yellow – fluorine, red – bromine, blue – nitrogen.

Table S11. Selected structural data of $Ph_3C[\mu\text{-}(CN)(B(C_6F_4Br)_3)_2]$ (*d* in Å, angles in °).

	$Ph_3C[\mu\text{-}(CN)(B(C_6F_4Br)_3)_2]$	contacts
d(C–N)	1.131(4)	
d(B_1–NC) and *d*(B_2–CN)	1.601(5) and 1.591(4)	
d(B_1–C_{Ph})	1.622(4) – 1.65	
d(B_2–C_{Ph})	1.633(4) – 1.636(4)	
d(C–Br)	1.867(3) – 1.876(3)	
Average B–N–C angle	173.0	
Average Torsion angle C–B–B–C	13.9	
C^+–C_{Ph}	1.437(4) – 1.446(4)	
C_{Ph}–C_{Ph}	1.373(5) – 1.417(4)	
d(C_{cation}–C_{BCFBr})	3.311(4) – 3.390(4)	2
d(C_{cation}–F)	3.002(4) – 3.152(3)	6
d(H_{cation}–F)	2.382(2) – 2.624(2)	7
d(F–F)	2.668(2) – 2.904(3)	5
d(Br–F)	3.074(1) – 3.262(2)	4
d(Br–Br)	3.561(1), 3.662(1)	2

Table S12. Crystallographic data of $FeCp^*_2[\mu\text{-}(CN)(B(C_6F_4Br)_3)_2]$.

Identification code	2539287
Empirical formula	$C_{57}H_{30}B_2Br_6F_{24}FeN$
Formula weight	1741.75
Temperature/K	100.00
Crystal system	Monoclinic
Space group	*C*2/*c*
a/Å	13.8950(4)
b/Å	23.2920(6)
c/Å	18.1746(6)
α/°	90
β/°	90.6040(10)
γ/°	90
Volume/Å^3	5881.7(3)
Z	4
ρ_{calc} g/cm^3	1.967
μ/mm^{-1}	4.450
F(000)	3364.0
Crystal size/mm^3	0.411 × 0.328 × 0.286
Radiation	MoK_α (λ = 0.71073)
2Θ range for data collection/°	4.066 to 51.364
Index ranges	-16 ≤ h ≤ 14, -28 ≤ k ≤ 28, -22 ≤ l ≤ 21
Reflections collected	28601
Independent reflections	5584 [R_{int} = 0.0427, R_{sigma} = 0.0315]
Data/restraints/parameters	5584/0/416
Goodness-of-fit on F^2	1.050
Final R indexes [I>=2σ (I)]	R_1 = 0.0251, wR_2 = 0.0561
Final R indexes [all data]	R_1 = 0.0306, wR_2 = 0.0585
Largest diff. peak/hole / e Å^{-3}	0.58/-0.55

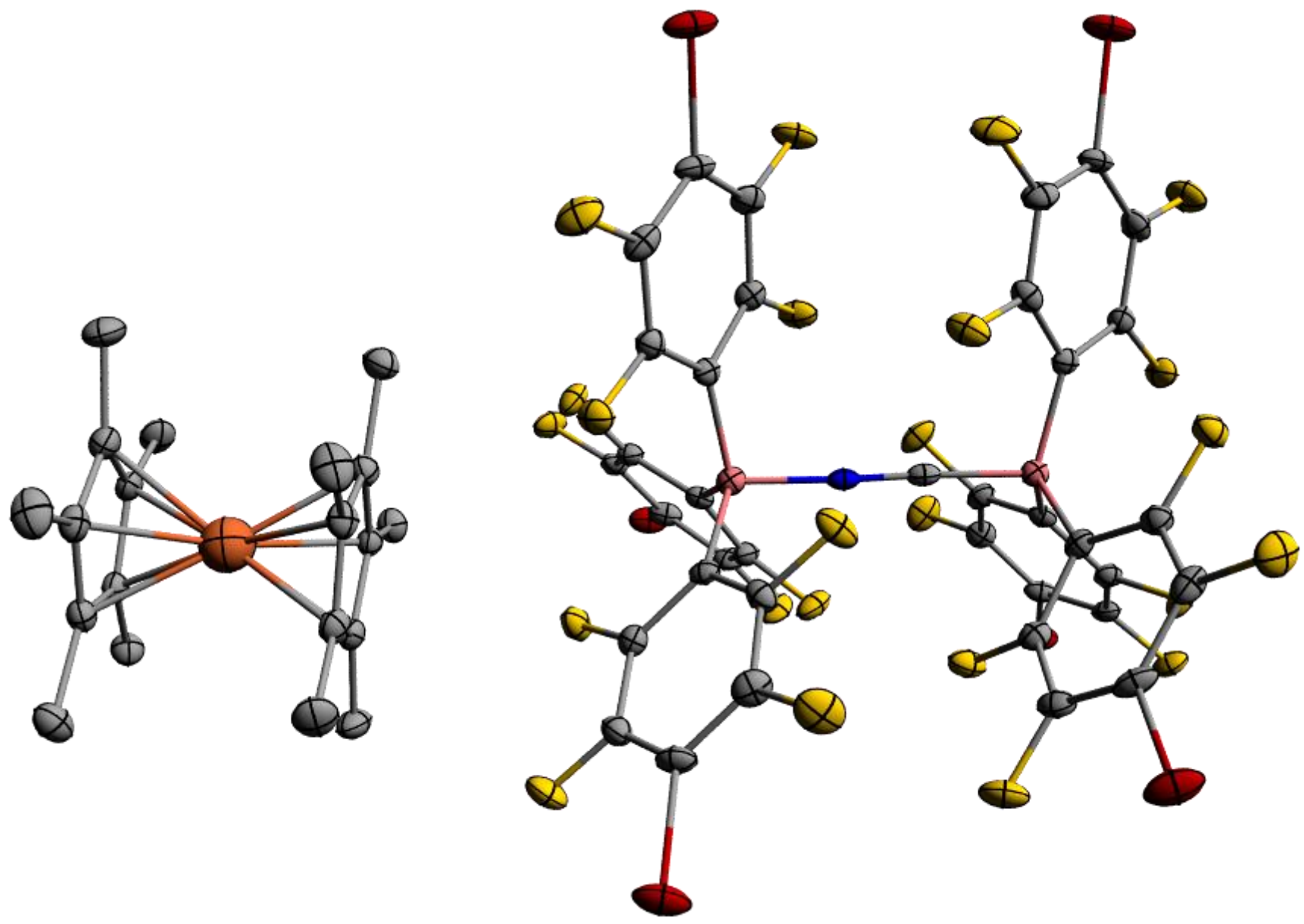

Figure S47. Molecular structure of $FeCp^*_2[\mu\text{-}(CN)(B(C_6F_4Br)_3)_2]$ in the solid state. Hydrogen atoms are omitted and ellipsoids are depicted with 50% probability level. Color Code: pink – boron, grey – carbon, yellow – fluorine, red – bromine, blue – nitrogen, orange – iron.

Table S13. Selected structural data of $FeCp^*_2[\mu\text{-}(CN)(B(C_6F_4Br)_3)_2]$ (*d* in Å, angles in °).

	$FeCp^*_2[\mu\text{-}(CN)(B(C_6F_4Br)_3)_2]$	contacts
d(C–N)	1.139(3)	
d(B–C1|N1)	1.590(4)	
d(B_1–C_{Ph})	1.629(4) – 1.629(4)	
d(C–Br)	1.87 – 1.873(3)	
Average B–N–C angle	177.5	
Average Torsion angle C–B–B–C	–14.6	
Fe–Cp*	1.71	
d(C_{cation}–F)	3.137(3), 3.147(3)	2
d(H_{Cation}–F)	2.593(1) – 2.640(2)	4
d(H_{Cation}–Br)	2.875(1), 2.890(1)	2
d(F–F)	2.8	1
d(Br–F)	3.221(2)	1
d(Br–Br)	3.561(1), 3.633(1), 3.671(1)	2

Table S14. Crystallographic data of $N(4\text{-}BrC_6H_4)_3[\mu\text{-}(CN)(B(C_6F_4Br)_3)_2(CH_2Cl_2)_{4.4}]$.

Identification code	2539288
Empirical formula	$C_{59.4}H_{20.55}B_2Br_9Cl_{8.8}F_{24}N_2$
Formula weight	2270.90
Temperature/K	100.00
Crystal system	triclinic
Space group	$P\bar{1}$
a/Å	11.7051(4)
b/Å	15.6805(6)
c/Å	22.2282(8)
α/°	73.7200(10)
β/°	82.8610(10)
γ/°	68.8990(10)
Volume/Å^3	3652.4(2)
Z	2
ρ_{calc}g/cm^3	2.065
μ/mm^{-1}	5.364
F(000)	2163.0
Crystal size/mm^3	0.254 × 0.178 × 0.046
Radiation	MoKα (λ = 0.71073)
2Θ range for data collection/°	3.856 to 51.424
Index ranges	-13 ≤ h ≤ 14, -19 ≤ k ≤ 19, -27 ≤ l ≤ 27
Reflections collected	67955
Independent reflections	13882 [R_{int} = 0.0545, R_{sigma} = 0.0437]
Data/restraints/parameters	13882/180/1145
Goodness-of-fit on F^2	1.034
Final R indexes [I>=2σ (I)]	R_1 = 0.0395, wR_2 = 0.0788
Final R indexes [all data]	R_1 = 0.0577, wR_2 = 0.0858
Largest diff. peak/hole / e Å^{-3}	1.03/-1.44

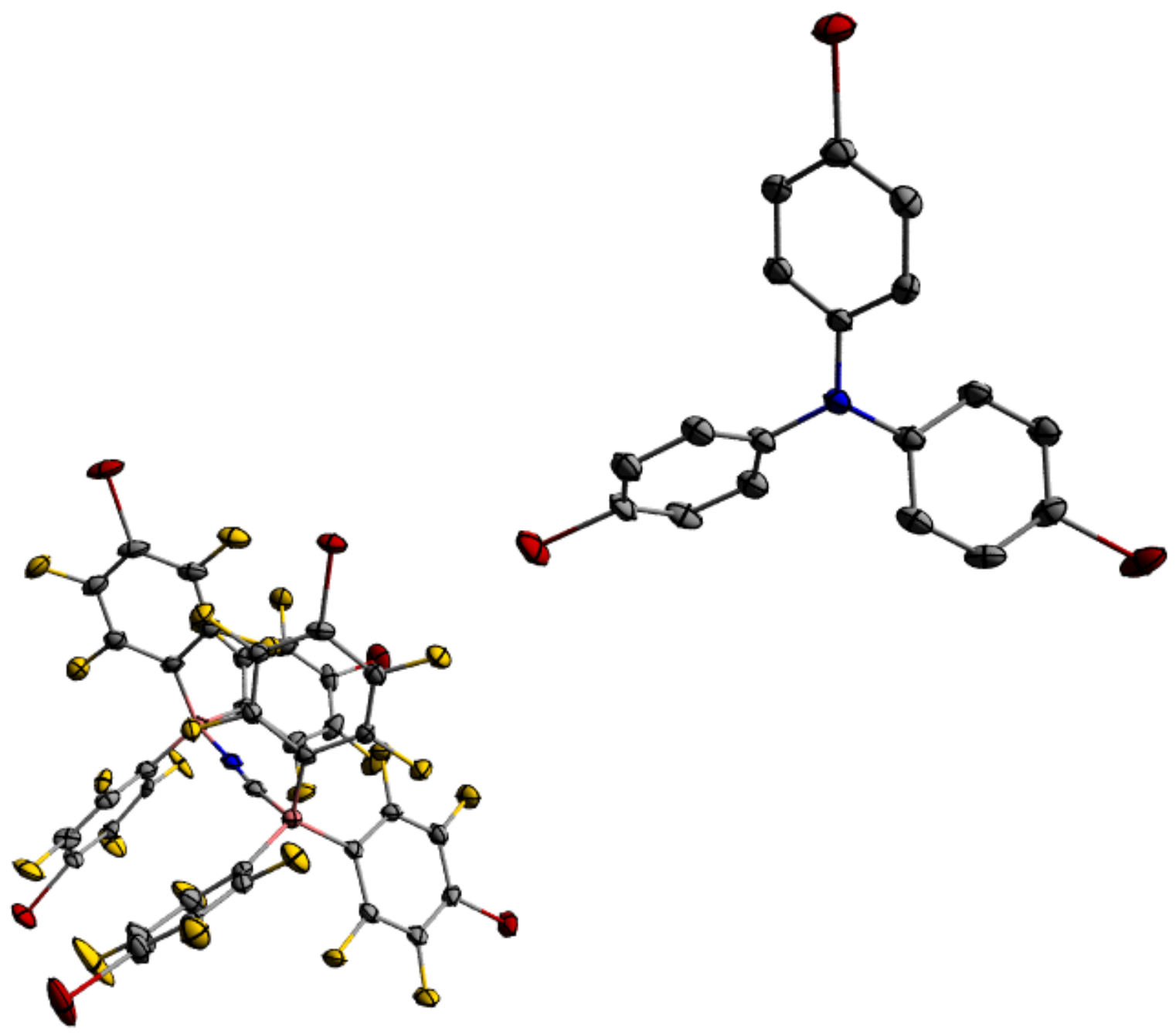

Figure S48. Molecular structure of $N(4\text{-}BrC_6H_4)_3[\mu\text{-}(CN)(B(C_6F_4Br)_3)_2(CH_2Cl_2)_{4.4}]$ in the solid state. Disorder, co-crystallized CH_2Cl_2 and hydrogen atoms are omitted. Ellipsoids are depicted with 50% probability level. Color Code: pink – boron, grey – carbon, yellow – fluorine, red – bromine, blue – nitrogen.

Table S15. Selected structural data of $N(4\text{-}BrC_6H_4)_3[\mu\text{-}(CN)(B(C_6F_4Br)_3)_2(CH_2Cl_2)_{4.4}]$ (*d* in Å, angles in °).

	$N(4\text{-}BrC_6H_4)_3[\mu\text{-}(CN)(B(C_6F_4Br)_3)_2(CH_2Cl_2)_{4.4}]$	contacts
d(C–N)	1.144(6)	
d(B_1–C1|N1) and *d*(B_2–N2|C2)	1.592(4) and 1.596(4)	
d(B_1–C_{Ph})	1.631(7) – 1.638(6)	
d(B_2–C_{Ph})	1.631(7) – 1.64(1)	
d(C–Br)	1.86(2) – 1.879(5)	
Average B–N–C angle	174.0	
Average Torsion angle C–B–B–C	–26.1	
N–C_{Ph}	1.399(7), 1.417(5), 1.42(1)	
d(H_{Cation}–C_{BCFBr})	2.837(37), 2.857(4)	2
d(C_{Cation}–F)	3.169(7)	1
d(C_{Cation}–Br)	3.517(5)	1
d(H_{Cation}–F)	2.450(2) – 2.641(2)	4
d(H_{Cation}–Br)	3.004(1)	1
d(Br_{Cation}–Br)	3.548(1)	1
d(Br_{Cation}–F)	2.980(3) – 3.051(9)	3
d(F–F)	2.689(4), 2.827(3)	2
d(Br–Br)	3.576(7)	1
d(Br–F)	2.965(2) – 3.274(5)	5

Table S16. Crystallographic data of $[C_6H_4S_2C_6H_4][\mu\text{-}(CN)(B(C_6F_4Br)_3)_2]$.

Identification code	2539289
Empirical formula	$C_{49}H_8B_2Br_6F_{24}NS_2$
Formula weight	1631.76
Temperature/K	100.00
Crystal system	triclinic
Space group	$P\bar{1}$
a/Å	12.1574(4)
b/Å	14.4362(5)
c/Å	15.5810(5)
α/°	70.3030(10)
β/°	73.6660(10)
γ/°	83.0380(10)
Volume/Å^3	2469.60(14)
Z	2
ρ_{calc}g/cm^3	2.194
μ/mm^{-1}	5.094
F(000)	1554.0
Crystal size/mm^3	0.944 × 0.329 × 0.2
Radiation	MoKα (λ = 0.71073)
2Θ range for data collection/°	3.43 to 57.72
Index ranges	-15 ≤ h ≤ 16, -19 ≤ k ≤ 19, -21 ≤ l ≤ 21
Reflections collected	53407
Independent reflections	12963 [R_{int} = 0.0614, R_{sigma} = 0.0603]
Data/restraints/parameters	12963/0/751
Goodness-of-fit on F^2	1.062
Final R indexes [I>=2σ (I)]	R_1 = 0.0436, wR_2 = 0.0992
Final R indexes [all data]	R_1 = 0.0654, wR_2 = 0.1080
Largest diff. peak/hole / e Å^{-3}	1.30/-0.73

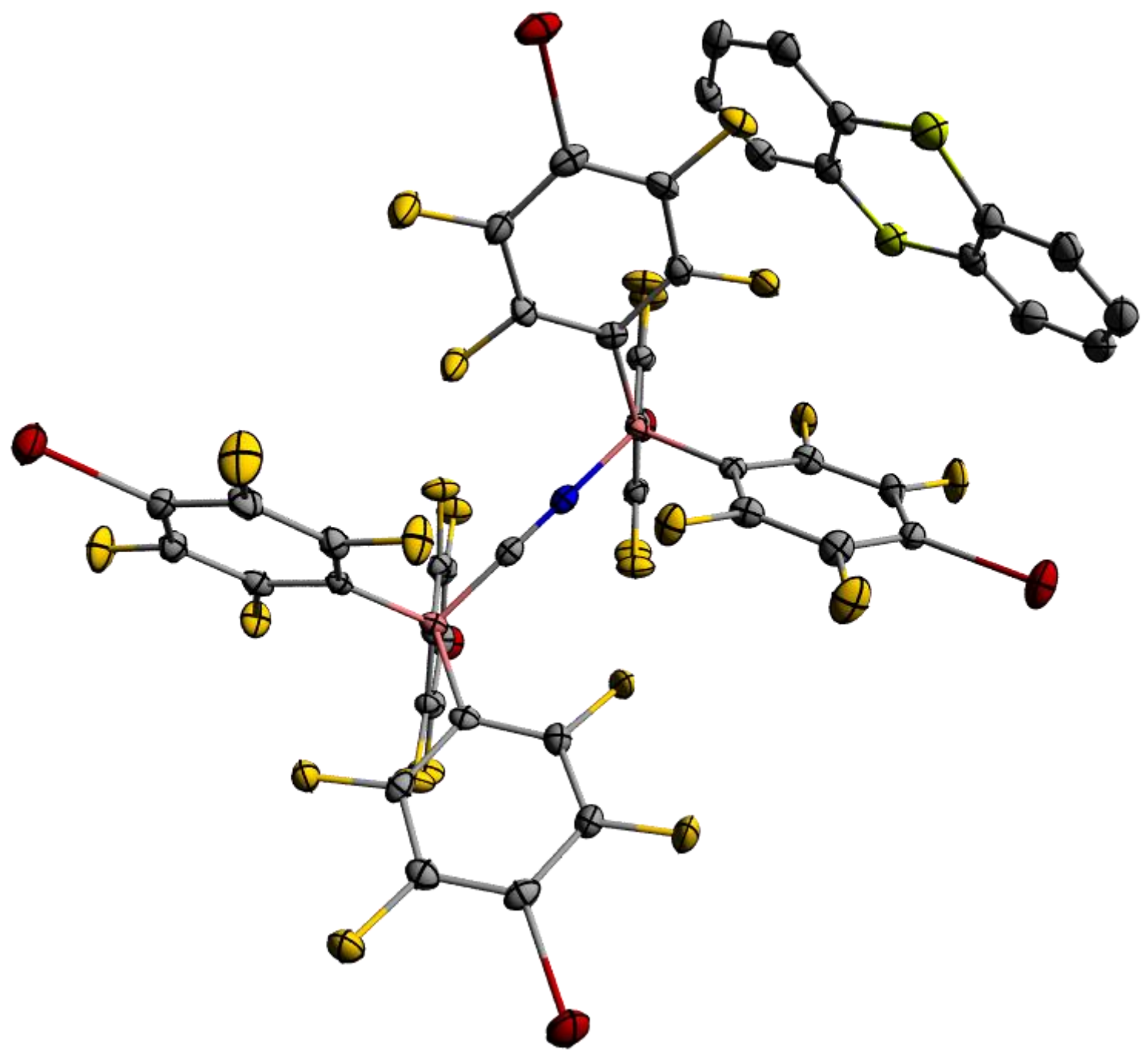

Figure S49. Molecular structure of $[C_6H_4S_2C_6H_4][\mu\text{-}(CN)(B(C_6F_4Br)_3)_2]$ in the solid state. Hydrogen atoms are omitted. Ellipsoids are depicted with 50% probability level. Color Code: pink – boron, grey – carbon, yellow – fluorine, red – bromine, blue – nitrogen, lighter yellow – sulphur.

Table S17. Selected structural data of $[C_6H_4S_2C_6H_4][\mu\text{-}(CN)(B(C_6F_4Br)_3)_2]$ (*d* in Å, angles in °).

	$[C_6H_4S_2C_6H_4][\mu\text{-}(CN)(B(C_6F_4Br)_3)_2]$	contacts
d(C–N)	1.142(5)	
d(B_1–C1|N1) and *d*(B_2–N2|C2)	1.592(6) and 1.591(6)	
d(B_1–C_{Ph})	1.635(5) – 1.641(5)	
d(B_2–C_{Ph})	1.626(5) – 1.640(6)	
d(C–Br)	1.869(4) – 1.877(3)	
Average B–N–C angle	174.2	
Average Torsion angle C–B–B–C	7.7	
d(S–C)	1.720(4) – 1.729(4)	
d($C_{thianthrene}$–$C_{thianthrene}$)	3.377(6)	1
d(S–F)	3.230(3) – 3.258(2)	3
d($C_{Thianthrene}$–F)	2.979(4) – 3.120(6)	4
d($H_{Thianthrene}$–F)	2.481(2) – 2.664(3)	4
d(F–F)	2.642(4) – 2.918(4)	5
d(Br–F)	3.013(2) – 3.273(3)	7
d(Br–Br)	3.629(1)	1

Table S18. Crystallographic data of $N(2,4\text{-}Br_2C_6H_3)_3[\mu\text{-}(CN)(B(C_6F_4Br)_3)_2]$.

Identification code	2539290
Empirical formula	$C_{60.5}H_{22.2}B_2Br_{12}F_{24}N_2$
Formula weight	2213.54
Temperature/K	100.00
Crystal system	triclinic
Space group	$P\bar{1}$
a/Å	13.0905(13)
b/Å	13.2238(13)
c/Å	22.269(2)
α/°	87.250(4)
β/°	77.878(4)
γ/°	60.607(3)
Volume/Å^3	3276.9(6)
Z	2
ρ_{calc}g/cm^3	2.243
μ/mm^{-1}	7.446
F(000)	2090.0
Crystal size/mm^3	1 × 0.621 × 0.308
Radiation	MoKα (λ = 0.71073)
2Θ range for data collection/°	3.9 to 50.876
Index ranges	-15 ≤ h ≤ 15, -15 ≤ k ≤ 15, -26 ≤ l ≤ 26
Reflections collected	79990
Independent reflections	11973 [R_{int} = 0.1018, R_{sigma} = 0.0643]
Data/restraints/parameters	11973/234/808
Goodness-of-fit on F^2	1.122
Final R indexes [I>=2σ (I)]	R_1 = 0.0997, wR_2 = 0.2292
Final R indexes [all data]	R_1 = 0.1295, wR_2 = 0.2507
Largest diff. peak/hole / e Å^{-3}	3.07/-1.76

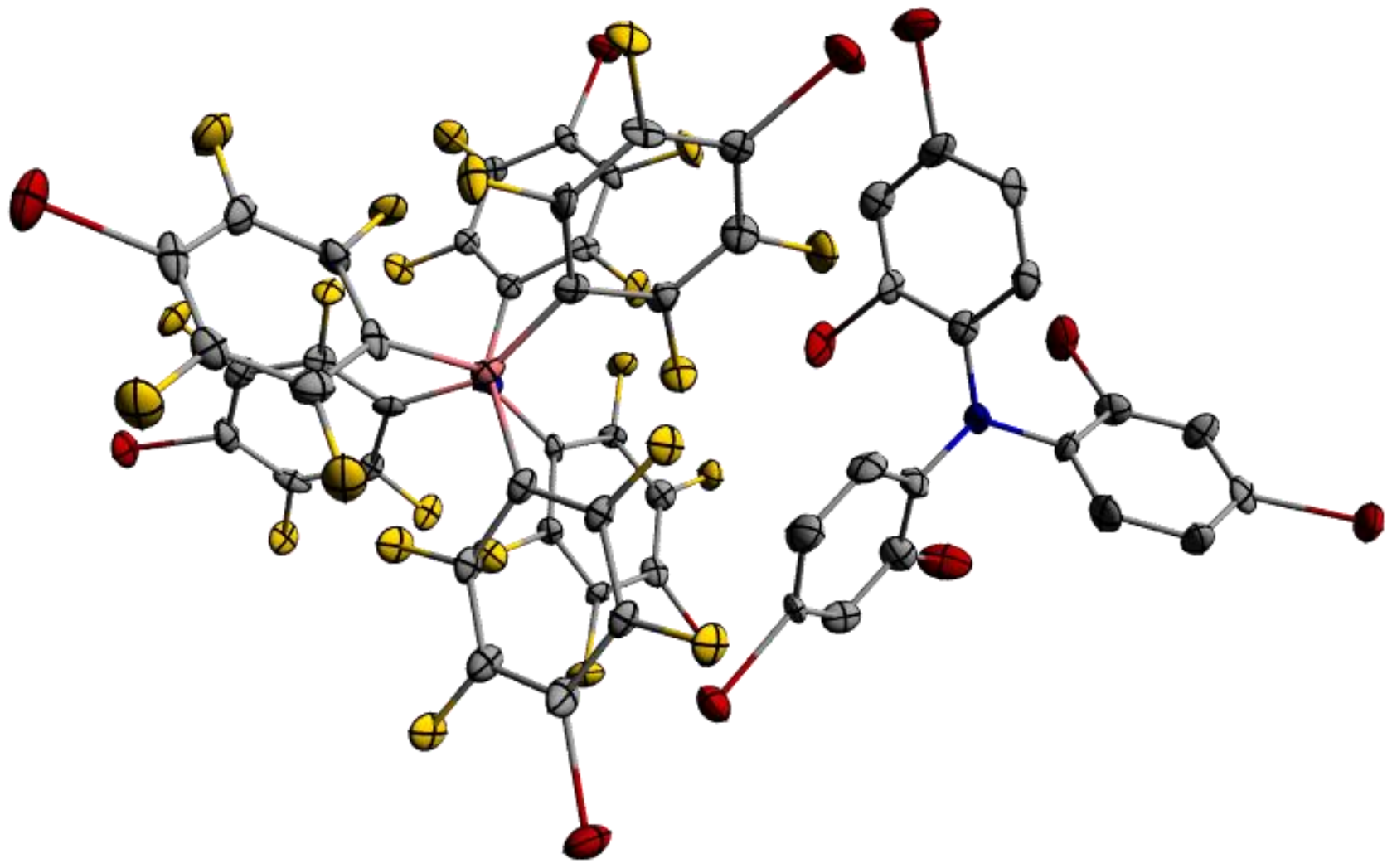

Figure S50. Molecular structure $N(2,4\text{-}Br_2C_6H_3)_3[\mu\text{-}(CN)(B(C_6F_4Br)_3)_2]$ in the solid state. Hydrogen atoms are omitted. Ellipsoids are depicted with 50% probability level. Color Code: pink – boron, grey – carbon, yellow – fluorine, red – bromine, blue – nitrogen.

Table S19. Selected structural data of $N(2,4\text{-}Br_2C_6H_3)_3[\mu\text{-}(CN)(B(C_6F_4Br)_3)_2]$ (*d* in Å, angles in °).

	$N(2,4\text{-}Br_2C_6H_3)_3[\mu\text{-}(CN)(B(C_6F_4Br)_3)_2]$	contacts
d(C–N)	1.124(19)	
d(B_1–N/C)	1.62(2) and 1.669(22)	
d(B_1–C_{Ph})	1.625(21) – 1.67(2)	
d(B_2–C_{Ph})	1.606(26) – 1.659(23)	
d(C–Br)	1.846(14) – 1.883(12)	
Average B–N–C angle	176.5	
Average Torsion angle C–B–B–C	–30.2	
N–C_{Ph}	1.43(2), 1.44(2), 1.44(3)	
d(C_{Cation}–F)	2.99(2) – 3.15(2)	5
d(H_{Cation}–F)	2.358(4) – 2.520(4)	9
d(Br_{Cation}–Br_{cation})	3.634(2), 3.683(3)	2
d(Br_{Cation}–F)	3.194(8) – 3.21(1)	3
d(F–F)	2.54(1) – 2.94(1)	5
d(Br–F)	3.21 – 3.282(7)	2

Comparison structural data

Table S20. Selected structural data of [BCNB^{Br}] salts and [BCNB] salts from literature (d in Å, angles in °).

	d(C–N)	Average B–N–C Angle	Average Torsion angle C–B–B–C
$[Ag(CH_2Cl_2)_2]$	1.148(11)	177.8	–59.2
[Ag(toluene)$_3$]	1.140(4)	173.7	–18.1
[Ag(*o*DFB)$_3$][BCNB][12]	1.143	176.2	–61.0
NBu_4^+	1.150(6)	179.1	–15.2
Tritylium	1.131(4)	173.0	13.9
Tritylium[BCNB][11]	1.144(2)	174.1	–11.5
FeCp*$_2^+$	1.139(3)	177.5	–14.6
Thianthrenium	1.142(5)	174.2	7.7
Magic blue	1.144(6)	174.0	–26.1
Magic green	1.124(19)	176.5	–30.2

Table S21. Selected structural data of Ag salts with [BCNBBr], [BCNB] and [BArF$_{20}$] anions (d in Å).

	**Ag[BCNB^{Br}]**		**Ag[BCNB][12]**	**Ag[BArF$_{20}$][6]**
Solvate	2 CH_2Cl_2	3 toluene	3 *o*DFB	3 toluene
Spacegroup	$P1$	$P2_1/n$	$P\bar{1}$	$P\bar{1}$
cation-anion contacts	Ag–Br<br>2.712<br>2.797<br>2.803<br>AgF<br>2.603	-	Ag–F<br>2.976<br>3.177	
d_{av}(Ag–arene$_{centroid}$)		2.794	2.841	2.801
d_{av}(Ag–arene$_{plane}$)		2.396	2.391	2.368
d(Ag–C)		2.397-3.418	2.382 – 3.316	2.413-3.297

Bromine-Bromine interactions

Table S22. Bromine–bromine interactions of [BCNB^{Br}] salts (*d* in Å).

	Br–Br aggregation	Br–Br contacts	*d*(Br–Br) **(<ΣR$_{vdW}$=3.7 Å)**	Comment
[Ag(CH_2Cl_2)$_2$]	–	1	3.677(1)	–
[Ag(toluene)$_3$]	Two distinct octahedra	2	3.446(1), 3.635(6)	Complete range of Br–Br distances in the octahedra : 3.446(1) – 4.753(1)
NBu_4^+	–	–	–	–
Tritylium	Four out of six Br atoms of each anion form Br–Br contacts with in total two neighbouring anions, resulting in a chain structure of the anions	2	3.561(1), 3.662(1)	Two additional Br–Br contacts of 3.823(1) and 3.978
FeCp*$_2^+$	One (Br)$_6$-octahedron	3	3.561(1), 3.633(1), 3.671(1)	Complete range of Br–Br distances in the octahedron: 3.561(1) – 3.976(1)
Thianthrenium	Depending on the cut-off value either two out of six or all Br atoms of each anion form one Br–Br contact with in total either two or six other neighbouring anions	1	3.629(1)	Two additional Br–Br contacts of 3.808(1) and 3.814(1)
Magic blue	There is one Br–Br contact between two neighbouring anions and depending on the cut-off value either one or three Br atoms of each anion form Br–Br contacts with either one or all Br atoms of the cation	2	3.548(1), 3.576(7)	Two additional Br–Br contacts of 3.819(1) and 3.989(1)
Magic green	(Br)$_6$-octahedron with three Br from cation and three Br-atoms from anion	2	3.634(2), 3.683(3)	3.634(2) – 4.331(2)

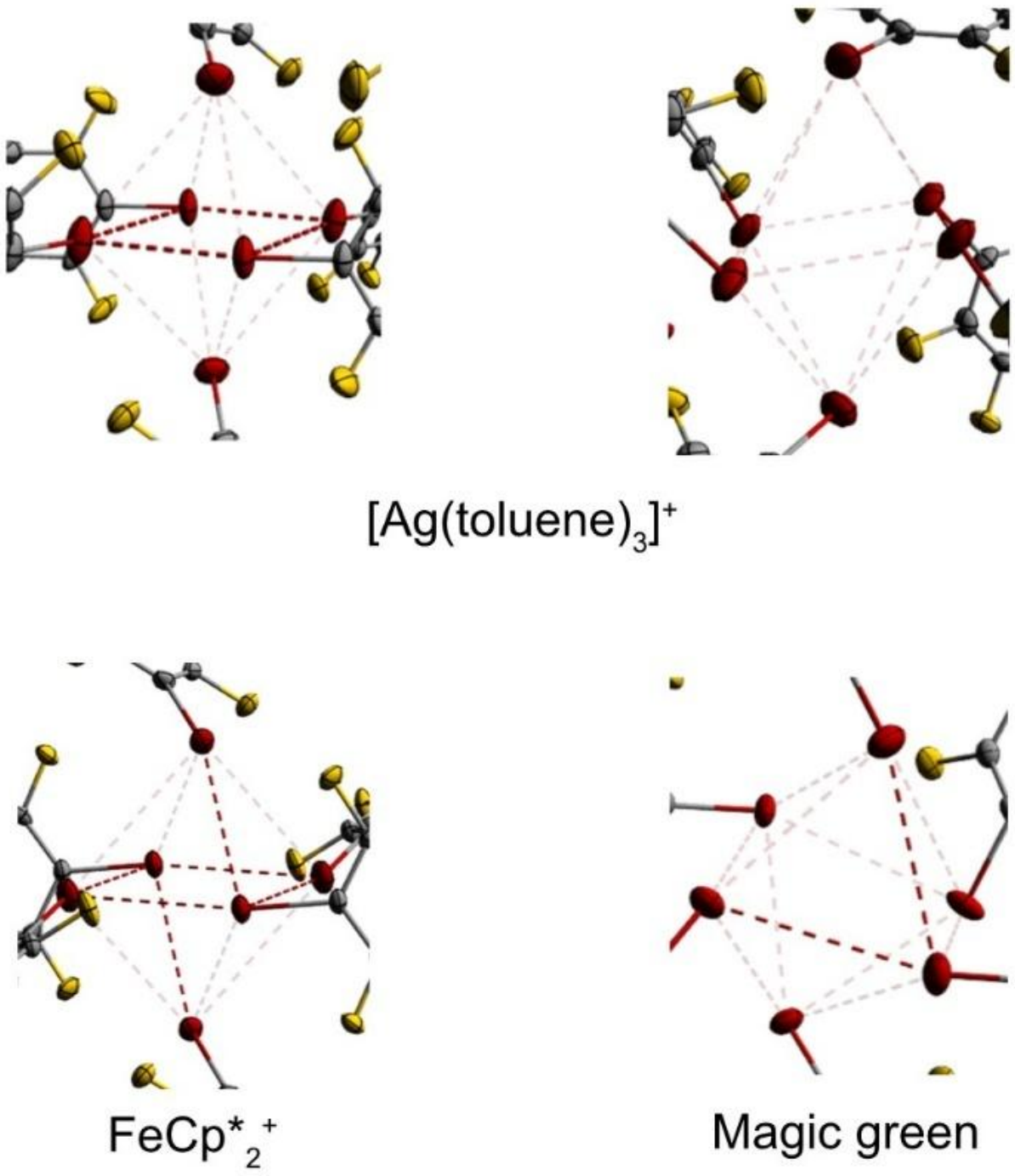

Figure S51. Bromine-Bromine aggregation to $(Br)_6$-octahedra in the solid-state structures of $[Ag(toluene)_3][BCNB^{Br}]$ (top), $FeCp^*_2[BCNB^{Br}]$ (bottom left) and $N(2,4\text{-}Br_2C_6H_3)_3[BCNB^{Br}]$ (bottom right). Intermolecular Br–Br contacts below the sum of the van der Waals radii (ΣR_{vdW}=3.7 Å) are depicted in dashed, red lines; all distances above this threshold are pale red. Hydrogen atoms and disorder are omitted. Ellipsoids are depicted with 50% probability level. Color Code: pink – boron, grey – carbon, yellow – fluorine, red – bromine, blue – nitrogen.

References

[18] A. L. Moshtaha, E. M. Peter, R. Sievers, T. N. Streit, M. Malischewski, "Synthesis of the Soft Lewis Superacid Tris(4-bromo-2,3,5,6-tetrafluorophenyl)borane $B(C_6F_4Br)_3$ via C–Ag to C–B Transmetalation" *Chemistry – A European Journal* **31** (2025): e02240.

[24] G. R. Fulmer, A. J. M. Miller, N. H. Sherden, H. E. Gottlieb, B. M. Stoltz, J. E. Bercaw, K. I. Goldberg, „NMR Chemical Shifts of Trace Impurities: Common Laboratory Solvents, Organics, and Gases in Deuterated Solvents Relevant to the Organometallic Chemist" *Organometallics* **29** (2010): 2176–2179.

[25] R. K. Harris, E. D. Becker, S. M. Cabral de Menezes, P. Granger, R. E. Hoffman, K. W. Zilm, "Further Conventions for NMR Shielding and Chemical Shifts (IUPAC Recommendations 2008)" *Pure and Applied Chemistry* **80** (2008): 59–84.

[26] MestReNova 14.3.0-30573, Mestrelab Research S.L., **2022**.

[27] OriginPro, Version 2024. OriginLab Corporation, Northhampton, MA, USA.

[28] O. V. Dolomanov, L. J. Bourhis, R. J. Gildea, J. A. K. Howard, H. Puschmann, "Olex2: A Complete Structure Solution, Refinement and Analysis Program." *Journal of Applied Crystallography* **42** (2009): 339–341.

[29] G. M. Sheldrick, "SHELXT – Integrated Space-group and Crystal Structure Determination" *Acta Crystallographica* **A71** (2015): 3–8.

[30] G. M. Sheldrick, SHELXL Version 2014/7, Program for Crystal Structure Solution and Refinement, University of Göttingen, Göttingen (Germany), 2014.

[31] G. M. Sheldrick, "A Short History of SHELX" *Acta Crystallographica* **A64** (2008): 112–122.

[32] K. Brandenburg, Diamond: Crystal and Molecular Structure Visualization http://www.crystalimpact.com/diamond

[33] Persistence of Vision Pty. Ltd. Persistence of Vision Raytracer. Ltd., Persistence of Vision Pty. 2004.

[34] Gaussian 16, Revision C.01, M. J. Frisch, G. W. Trucks, H. B. Schlegel, G. E. Scuseria, M. A. Robb, J. R. Cheeseman, G. Scalmani, V. Barone, G. A. Petersson, H. Nakatsuji, X. Li, M.

Caricato, A. V. Marenich, J. Bloino, B. G. Janesko, R. Gomperts, B. Mennucci, H. P. Hratchian, J. V. Ortiz, A. F. Izmaylov, J. L. Sonnenberg, D. Williams-Young, F. Ding, F. Lipparini, F. Egidi, J. Goings, B. Peng, A. Petrone, T. Henderson, D. Ranasinghe, V. G. Zakrzewski, J. Gao, N. Rega, G. Zheng, W. Liang, M. Hada, M. Ehara, K. Toyota, R. Fukuda, J. Hasegawa, M. Ishida, T. Nakajima, Y. Honda, O. Kitao, H. Nakai, T. Vreven, K. Throssell, J. A. Montgomery, Jr., J. E. Peralta, F. Ogliaro, M. J. Bearpark, J. J. Heyd, E. N. Brothers, K. N. Kudin, V. N. Staroverov, T. A. Keith, R. Kobayashi, J. Normand, K. Raghavachari, A. P. Rendell, J. C. Burant, S. S. Iyengar, J. Tomasi, M. Cossi, J. M. Millam, M. Klene, C. Adamo, R. Cammi, J. W. Ochterski, R. L. Martin, K. Morokuma, O. Farkas, J. B. Foresman, and D. J. Fox, Gaussian, Inc., Wallingford CT, 2016.

[35] M. D. Hanwell, D. E. Curtis, D. C. Lonie, T. Vandermeersch, E. Zurek, G. R. Hutchison, “Avogadro: An Advanced Semantic Chemical Editor, Visualization, and Analysis Platform.” *Journal of Cheminformatics* **4** (2012): 17.

[36] G. A. Zhurko, in ChemCraft, http://www.chemcraftprog.com.

[37] M. K. Manoj, B. Brauer, J. M. L. Martin, „Frequency and Zero-Point Vibrational Energy Scale Factors for Double-Hybrid Density Functionals (and Other Selected Methods): Can Anharmonic Force Fields Be Avoided?“ *The Journal of Physical Chemistry A* **119** (2015): 1701−1714.
