# Supplementary material for: Expanding Cyanide‐Bridged Weakly Coordinating Anions Through the Brominated Silver Salt Ag[BCNBBr]
Source: Chemistry. 2026 Jun 27;32(38):e71310. doi: 10.1002/chem.71310 (PMC13660483; doi:10.1002/chem.71310)

checkCIF/PLATON report

Structure factors have been supplied for datablock(s) neuezelle

THIS REPORT IS FOR GUIDANCE ONLY. IF USED AS PART OF A REVIEW PROCEDURE FOR PUBLICATION, IT SHOULD NOT REPLACE THE EXPERTISE OF AN EXPERIENCED CRYSTALLOGRAPHIC REFEREE.

No syntax errors found. CIF dictionary Interpreting this report

Datablock: neuezelle

```
Bond precision:   C-C = 0.0050 A          Wavelength=0.71073

Cell:            a=13.5875(7)     b=22.9153(14)      c=19.0233(12)
                 alpha=90         beta=91.267(2)     gamma=90
Temperature:     150 K
```

	Calculated	Reported
Volume	5921.7(6)	5921.7(6)
Space group	P 21/n	P 1 21/n 1
Hall group	-P 2yn	-P 2yn
Moiety formula	C37 B2 Br6 F24 N, C21 H24 Ag	C21 H24 Ag, B2 Br6 C37 F24 N
Sum formula	C58 H24 Ag B2 Br6 F24 N	C58 H24 Ag B2 Br6 F24 N
Mr	1799.67	1799.73
Dx,g cm-3	2.019	2.019
Z	4	4
Mu (mm-1)	4.506	4.506
F000	3448.0	3448.0
F000’	3440.05	
h,k,lmax	17,29,24	17,29,24
Nref	13101	13081
Tmin,Tmax	0.644,0.637	0.194,0.746
Tmin’	0.631	

```
Correction method= # Reported T Limits: Tmin=0.194 Tmax=0.746
AbsCorr = NONE
```

```
Data completeness= 0.998           Theta(max)= 27.128

                                                      wR2(reflections)=
R(reflections)= 0.0393( 10000)                        0.0836( 13081)
S = 1.029                  Npar= 878
```

The following ALERTS were generated. Each ALERT has the format
test-name_ALERT_alert-type_alert-level.
Click on the hyperlinks for more details of the test.

Alert level C

```
PLAT042_ALERT_1_C Calc. and Reported MoietyFormula Strings  Differ     Please Check
              Calc: C37 B2 Br6 F24 N, C21 H24 Ag
              Rep.: C21 H24 Ag, B2 Br6 C37 F24 N
PLAT910_ALERT_3_C Missing FCF Reflection(s) Below Theta(Min)[Deg]=       2.03 Note
                1  1  0,   0  2  0,  -1  0  1,   1  0  1,   0  1  1,
```

Alert level G

```
PLAT003_ALERT_2_G Number of Uiso or U(i,j)  Restrained non-H-Atoms          9 Report
PLAT083_ALERT_2_G SHELXL Second Parameter in WGHT  Unusually Large      13.42 Why ?
PLAT168_ALERT_4_G The CIF-Embedded .res File Contains EXYZ Records          2 Report
PLAT171_ALERT_4_G The CIF-Embedded .res File Contains EADP Records          1 Report
PLAT186_ALERT_4_G The CIF-Embedded .res File Contains ISOR Records          4 Report
PLAT299_ALERT_4_G Atom Site Occupancy Constrained at ............         0.5 Check
              N1      N2      C1      C2      C3T4    C2T4    C6T4    C4T4
              C5T4    C1T4    C7T4    C6T3    C1T3    C2T3    C3T3    C4T3
              C5T3    C7T3    H3T4    H2T4    H6T4    H4T4    H5T4    H7TG
              H7TH    H7TI    H6T3    H2T3    H3T3    H4T3    H5T3    H7TJ
              H7TK    H7TL
PLAT301_ALERT_3_G Main Residue  Disorder .............(Resd    1)          3% Note
PLAT302_ALERT_4_G Anion/Solvent/Minor-Residue Disorder (Resd    2)         32% Note
PLAT343_ALERT_2_G Unusual          Angle Range in Main Residue for       C6T1 Check
PLAT343_ALERT_2_G Unusual          Angle Range in Main Residue for       C5T2 Check
PLAT434_ALERT_2_G Short Inter HL..HL Contact Br05     ..F123     .       3.13 Ang.
                                                -1+x,y,z  =      1_455 Check
PLAT434_ALERT_2_G Short Inter HL..HL Contact Br13     ..Br22     .       3.45 Ang.
                                         3/2-x,1/2+y,1/2-z  =      2_655 Check
PLAT720_ALERT_4_G Number of Unusual/Non-Standard Labels ..........         62 Note
              Ag01    Br05    C3T1    H3T1    C5T1    H5T1    C1T1    C2T1
              H2T1    C6T1    H6T1    C4T1    H4T1    C2T2    H2T2    C1T2
              C5T2    H5T2    C6T2    H6T2    C4T2    H4T2    C3T2    H3T2
              C7T1    H7TA    H7TB    H7TC    C3T4    H3T4    C2T4    H2T4
              C7T2    H7TD    H7TE    H7TF    C6T4    H6T4    C4T4    H4T4
              C5T4    H5T4    C1T4    C7T4    H7TG    H7TH    H7TI    C6T3
              H6T3    C1T3    C2T3    H2T3    C3T3    H3T3    C4T3    H4T3
              C5T3    H5T3    C7T3    H7TJ    H7TK    H7TL
PLAT779_ALERT_4_G Suspect or Irrelevant (Bond) Angle(s) in CIF ...       0.00 Deg.
              C1   -B1   -N1      1_555   1_555   1_555 ......     #  162 Check
PLAT779_ALERT_4_G Suspect or Irrelevant (Bond) Angle(s) in CIF ...       0.00 Deg.
              N2   -B2   -C2      1_555   1_555   1_555 ......     #  187 Check
PLAT779_ALERT_4_G Suspect or Irrelevant (Bond) Angle(s) in CIF ...       0.00 Deg.
              N2   -N1   -C2      1_555   1_555   1_555 ......     #  256 Check
```

```
PLAT779_ALERT_4_G Suspect or Irrelevant (Bond) Angle(s) in CIF ...       0.00 Deg.
              C1   -C2   -N1      1_555   1_555   1_555 ......     #  259 Check
PLAT779_ALERT_4_G Suspect or Irrelevant (Bond) Angle(s) in CIF ...       0.00 Deg.
              N2   -C1   -C2      1_555   1_555   1_555 ......     #  262 Check
PLAT779_ALERT_4_G Suspect or Irrelevant (Bond) Angle(s) in CIF ...       0.00 Deg.
              C1   -N2   -N1      1_555   1_555   1_555 ......     #  265 Check
PLAT789_ALERT_4_G Atoms with Negative _atom_site_disorder_group  #          2 Check
PLAT822_ALERT_4_G CIF-embedded .res Contains Negative PART Numbers          1 Check
PLAT860_ALERT_3_G Number of Least-Squares Restraints ............          54 Note
PLAT912_ALERT_4_G Missing # of FCF Reflections Above STh/L=  0.600         13 Note
PLAT969_ALERT_5_G The 'Henn et al.' R-Factor-gap value ..........       1.570 Note
              Predicted wR2: Based on SigI**2  5.32 or SHELX Weight  8.12
PLAT978_ALERT_2_G Number C-C Bonds with Positive Residual Density.          4 Info
PLAT994_ALERT_1_G SHELXL .ins Contains no or MERG 0 Instruction ..          ! Note
```

```
   0 ALERT level A = Most likely a serious problem - resolve or explain
   0 ALERT level B = A potentially serious problem, consider carefully
   2 ALERT level C = Check. Ensure it is not caused by an omission or oversight
  26 ALERT level G = General information/check it is not something unexpected

   2 ALERT type 1 CIF construction/syntax error, inconsistent or missing data
   7 ALERT type 2 Indicator that the structure model may be wrong or deficient
   3 ALERT type 3 Indicator that the structure quality may be low
  15 ALERT type 4 Improvement, methodology, query or suggestion
   1 ALERT type 5 Informative message, check
```

Validation response form

Please find below a validation response form (VRF) that can be filled in and pasted into your CIF.

```
# start Validation Reply Form
_vrf_PLAT042_neuezelle
;
PROBLEM: Calc. and Reported MoietyFormula Strings  Differ     Please Check
RESPONSE: ...
;
_vrf_PLAT910_neuezelle
;
PROBLEM: Missing FCF Reflection(s) Below Theta(Min)[Deg]=       2.03 Note
RESPONSE: ...
;
# end Validation Reply Form
```

It is advisable to attempt to resolve as many as possible of the alerts in all categories. Often the minor alerts point to easily fixed oversights, errors and omissions in your CIF or refinement strategy, so attention to these fine details can be worthwhile. It is up to the individual to critically assess their own results and, if necessary, seek expert advice.

PLATON version of 15/01/2026; check.def file version of 02/01/2026

---

duplicate check

No duplication found

Datablock neuezelle - ellipsoid plot

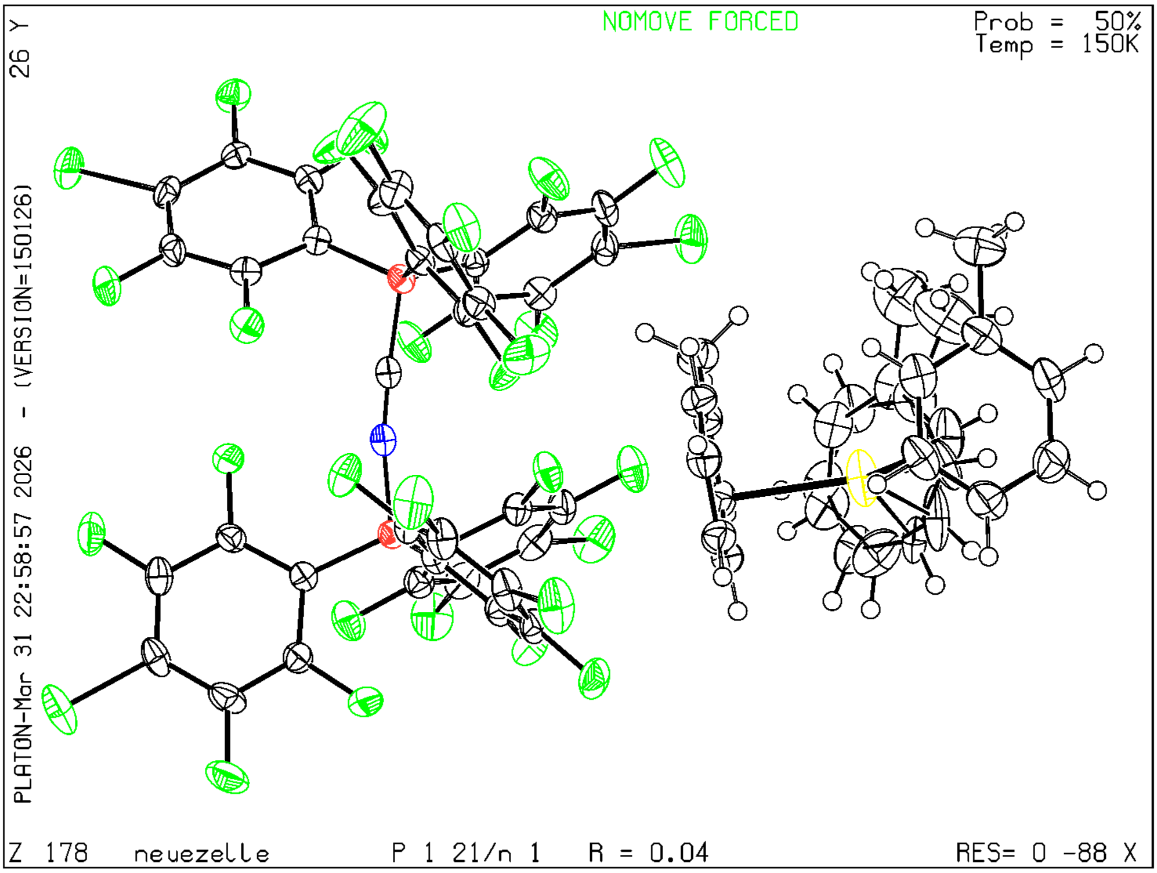

Supplement: Supplementary file 2 [file CHEM-32-e71310-s001.zip › chem71310-sup-0002-cif/ag toluene.pdf]
