# Supplementary material for: Expanding Cyanide‐Bridged Weakly Coordinating Anions Through the Brominated Silver Salt Ag[BCNBBr]
Source: Chemistry. 2026 Jun 27;32(38):e71310. doi: 10.1002/chem.71310 (PMC13660483; doi:10.1002/chem.71310)

checkCIF/PLATON report

Structure factors have been supplied for datablock(s) try1_p1_numabs

THIS REPORT IS FOR GUIDANCE ONLY. IF USED AS PART OF A REVIEW PROCEDURE FOR PUBLICATION, IT SHOULD NOT REPLACE THE EXPERTISE OF AN EXPERIENCED CRYSTALLOGRAPHIC REFEREE.

No syntax errors found. CIF dictionary Interpreting this report

Datablock: try1_p1_numabs

```
Bond precision:   C-C = 0.0147 A           Wavelength=0.71073

Cell:           a=10.4662(5)       b=10.9193(6)       c=11.8727(6)
                alpha=99.218(2)    beta=102.214(2)    gamma=117.409(2)
Temperature:    109 K
```

	Calculated	Reported
Volume	1124.20(11)	1124.20(10)
Space group	P 1	P 1
Hall group	P 1	P 1
Moiety formula	C38 H2 Ag B2 Br6 Cl2 F24 N, C H2 Cl2	C38 H2 Ag B2 Br6 Cl2 F24 N, C H2 Cl2
Sum formula	C39 H4 Ag B2 Br6 Cl4 F24 N	C39 H4 Ag B2 Br6 Cl4 F24 N
Mr	1693.12	1693.18
Dx,g cm-3	2.501	2.501
Z	1	1
Mu (mm-1)	6.155	6.155
F000	796.0	796.0
F000’	794.59	
h,k,lmax	14,15,16	14,15,16
Nref	13288[6644]	13051
Tmin,Tmax		0.295,0.649
Tmin’		

```
Correction method= # Reported T Limits: Tmin=0.295 Tmax=0.649
AbsCorr = NUMERICAL
```

Data completeness= 1.96/0.98 Theta(max)= 30.132

R(reflections)= 0.0620(12257) wR2(reflections)= 0.1609(13051)

S = 1.064 Npar= 689

The following ALERTS were generated. Each ALERT has the format
test-name_ALERT_alert-type_alert-level.
Click on the hyperlinks for more details of the test.

Alert level B

```
PLAT780_ALERT_1_B Coordinates do not Form a Properly Connected Set     Please Do !
PLAT973_ALERT_2_B Check Calcd Positive Resid. Density on       Ag1       1.56 eA-3
```

Alert level C

```
PLAT053_ALERT_1_C Minimum Crystal Dimension Missing (or Error) ...     Please Check
PLAT054_ALERT_1_C Medium  Crystal Dimension Missing (or Error) ...     Please Check
PLAT055_ALERT_1_C Maximum Crystal Dimension Missing (or Error) ...     Please Check
PLAT220_ALERT_2_C NonSolvent   Resd 1  C   Ueq(max)/Ueq(min) Range        3.7 Ratio
PLAT342_ALERT_3_C Low Bond Precision on  C-C Bonds ..............    0.01467 Ang.
PLAT972_ALERT_2_C Check Calcd Resid. Dens.  0.67Ang From Br12           -1.82 eA-3
PLAT972_ALERT_2_C Check Calcd Resid. Dens.  0.73Ang From Ag1            -1.64 eA-3
PLAT972_ALERT_2_C Check Calcd Resid. Dens.  0.75Ang From Br23           -1.61 eA-3
PLAT972_ALERT_2_C Check Calcd Resid. Dens.  0.74Ang From Br11           -1.57 eA-3
PLAT977_ALERT_2_C Check Negative Difference Density on H01A      .      -0.35 eA-3
```

Alert level G

```
PLAT004_ALERT_5_G Polymeric Structure Found with Maximum Dimension          2 Info
PLAT033_ALERT_4_G Flack x Value Deviates > 3.0 * Sigma from Zero .      0.245 Note
PLAT072_ALERT_2_G SHELXL First  Parameter in WGHT  Unusually Large       0.13 Report
PLAT154_ALERT_1_G The s.u.'s on the Cell Angles are Equal ..(Note)      0.002 Degree
PLAT168_ALERT_4_G The CIF-Embedded .res File Contains EXYZ Records          2 Report
PLAT171_ALERT_4_G The CIF-Embedded .res File Contains EADP Records          1 Report
PLAT299_ALERT_4_G Atom Site Occupancy Constrained at ............         0.5 Check
              N1      N2      C1      C2
PLAT301_ALERT_3_G Main Residue  Disorder ..............(Resd    1)         3% Note
PLAT434_ALERT_2_G Short Inter HL..HL Contact Cl08     ..F213     .       3.03 Ang.
                                                    x,y,z  =      1_555 Check
PLAT720_ALERT_4_G Number of Unusual/Non-Standard Labels ..........         10 Note
              Cl08    Cl09    Cl0A    Cl0B    C01X    H01A    H01B    C025
              H02A    H02B
PLAT779_ALERT_4_G Suspect or Irrelevant (Bond) Angle(s) in CIF ...       0.00 Deg.
              C2   -C1   -N2      1_555   1_555   1_555 ......     #    6 Check
PLAT779_ALERT_4_G Suspect or Irrelevant (Bond) Angle(s) in CIF ...       0.00 Deg.
              C2   -N1   -N2      1_555   1_555   1_555 ......     #    9 Check
PLAT779_ALERT_4_G Suspect or Irrelevant (Bond) Angle(s) in CIF ...       0.00 Deg.
              N1   -N2   -C1      1_555   1_555   1_555 ......     #   30 Check
PLAT779_ALERT_4_G Suspect or Irrelevant (Bond) Angle(s) in CIF ...       0.00 Deg.
              N1   -C2   -C1      1_555   1_555   1_555 ......     #   33 Check
PLAT779_ALERT_4_G Suspect or Irrelevant (Bond) Angle(s) in CIF ...       0.00 Deg.
              N1   -B1   -C1      1_555   1_555   1_555 ......     #  122 Check
```

```
PLAT779_ALERT_4_G Suspect or Irrelevant (Bond) Angle(s) in CIF ...       0.00 Deg.
              C2   -B2   -N2      1_555   1_555   1_555 ......     #  138 Check
PLAT789_ALERT_4_G Atoms with Negative _atom_site_disorder_group  #          2 Check
PLAT822_ALERT_4_G CIF-embedded .res Contains Negative PART Numbers          2 Check
PLAT883_ALERT_1_G Absent Datum for _atom_sites_solution_primary ..     Please Do !
PLAT910_ALERT_3_G Missing FCF Reflection(s) Below Theta(Min)[Deg]=       2.20 Note
                0  0  1,
PLAT912_ALERT_4_G Missing # of FCF Reflections Above STh/L=  0.600         24 Note
PLAT933_ALERT_2_G Number of HKL-OMIT Records in Embedded .res File          3 Note
                0  0 -1,   0  0  1,   5 -3-15,
PLAT969_ALERT_5_G The 'Henn et al.' R-Factor-gap value ..........      3.429 Note
              Predicted wR2: Based on SigI**2  4.69 or SHELX Weight 15.12
PLAT978_ALERT_2_G Number C-C Bonds with Positive Residual Density.          0 Info
PLAT994_ALERT_1_G SHELXL .ins Contains no or MERG 0 Instruction ..          ! Note
```

```
   0 ALERT level A = Most likely a serious problem - resolve or explain
   2 ALERT level B = A potentially serious problem, consider carefully
  10 ALERT level C = Check. Ensure it is not caused by an omission or oversight
  25 ALERT level G = General information/check it is not something unexpected

   7 ALERT type 1 CIF construction/syntax error, inconsistent or missing data
  11 ALERT type 2 Indicator that the structure model may be wrong or deficient
   3 ALERT type 3 Indicator that the structure quality may be low
  14 ALERT type 4 Improvement, methodology, query or suggestion
   2 ALERT type 5 Informative message, check
```

Validation response form

Please find below a validation response form (VRF) that can be filled in and pasted into your CIF.

```
# start Validation Reply Form
_vrf_PLAT780_try1_p1_numabs
;
PROBLEM: Coordinates do not Form a Properly Connected Set     Please Do !
RESPONSE: ...
;
_vrf_PLAT973_try1_p1_numabs
;
PROBLEM: Check Calcd Positive Resid. Density on       Ag1       1.56 eA-3
RESPONSE: ...
;
_vrf_PLAT053_try1_p1_numabs
;
PROBLEM: Minimum Crystal Dimension Missing (or Error) ...     Please Check
RESPONSE: ...
;
_vrf_PLAT054_try1_p1_numabs
;
PROBLEM: Medium  Crystal Dimension Missing (or Error) ...     Please Check
RESPONSE: ...
;
_vrf_PLAT055_try1_p1_numabs
;
PROBLEM: Maximum Crystal Dimension Missing (or Error) ...     Please Check
RESPONSE: ...
```

```
;
_vrf_PLAT220_try1_p1_numabs
;
PROBLEM: NonSolvent   Resd 1  C   Ueq(max)/Ueq(min) Range        3.7 Ratio
RESPONSE: ...
;
_vrf_PLAT342_try1_p1_numabs
;
PROBLEM: Low Bond Precision on  C-C Bonds ...............    0.01467 Ang.
RESPONSE: ...
;
_vrf_PLAT972_try1_p1_numabs
;
PROBLEM: Check Calcd Resid. Dens.  0.67Ang From Br12           -1.82 eA-3
RESPONSE: ...
;
_vrf_PLAT977_try1_p1_numabs
;
PROBLEM: Check Negative Difference Density on H01A      .      -0.35 eA-3
RESPONSE: ...
;
# end Validation Reply Form
```

It is advisable to attempt to resolve as many as possible of the alerts in all categories. Often the minor alerts point to easily fixed oversights, errors and omissions in your CIF or refinement strategy, so attention to these fine details can be worthwhile. It is up to the individual to critically assess their own results and, if necessary, seek expert advice.

PLATON version of 15/01/2026; check.def file version of 02/01/2026

---

duplicate check

No duplication found

Datablock try1_p1_numabs - ellipsoid plot

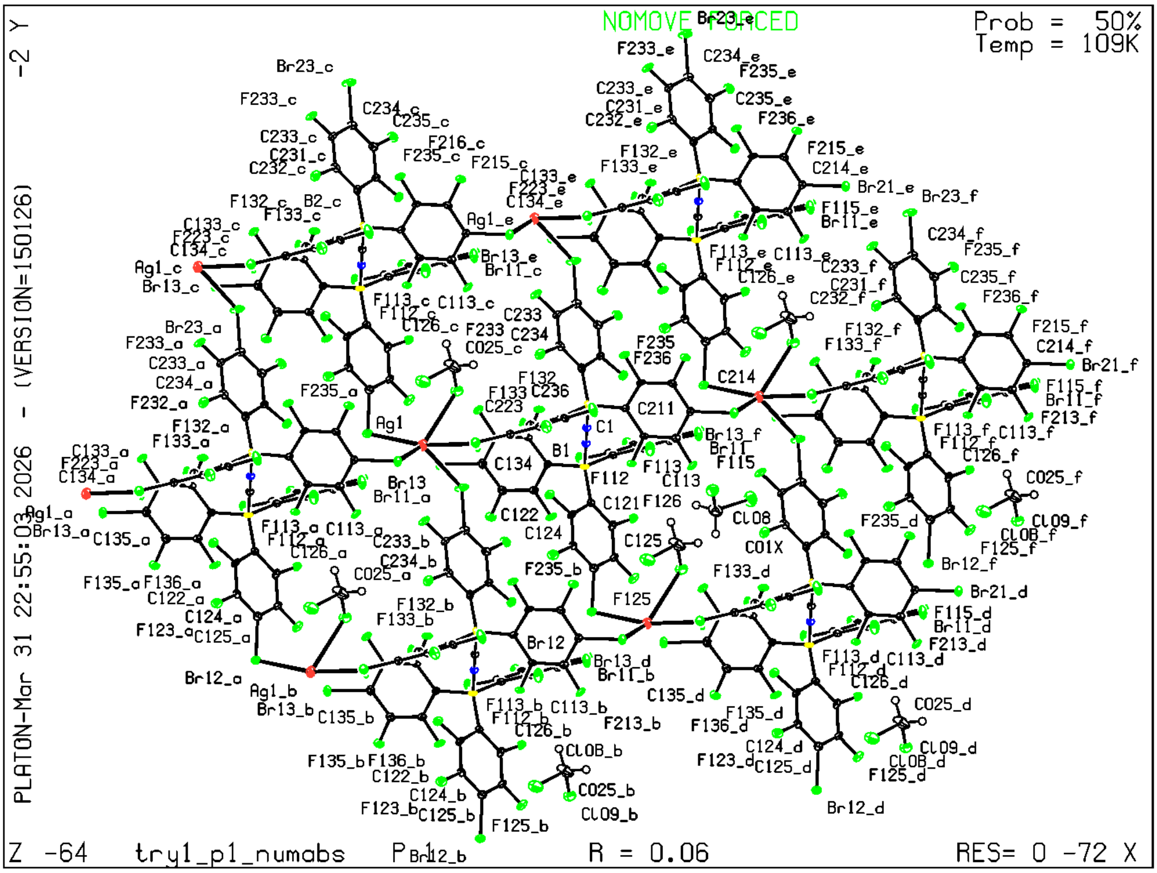

Supplement: Supplementary file 2 [file CHEM-32-e71310-s001.zip › chem71310-sup-0002-cif/AgBCNB dcm.pdf]
