# Supplementary material for: Expanding Cyanide‐Bridged Weakly Coordinating Anions Through the Brominated Silver Salt Ag[BCNBBr]
Source: Chemistry. 2026 Jun 27;32(38):e71310. doi: 10.1002/chem.71310 (PMC13660483; doi:10.1002/chem.71310)

checkCIF/PLATON report

Structure factors have been supplied for datablock(s) c2c_neu

THIS REPORT IS FOR GUIDANCE ONLY. IF USED AS PART OF A REVIEW PROCEDURE FOR PUBLICATION, IT SHOULD NOT REPLACE THE EXPERTISE OF AN EXPERIENCED CRYSTALLOGRAPHIC REFEREE.

No syntax errors found. CIF dictionary Interpreting this report

Datablock: c2c_neu

Bond precision:	C-C = 0.0035 A		Wavelength=0.71073
Cell:	a=13.8950(4)	b=23.2920(6)	c=18.1746(6)
	alpha=90	beta=90.604(1)	gamma=90
Temperature:	100 K		

	Calculated	Reported
Volume	5881.7(3)	5881.7(3)
Space group	C 2/c	C2/c
Hall group	-C 2yc	-C 2yc
Moiety formula	2(C18.50 B Br3 F12 N0.50), C20 H30 Fe	B2 Br6 C37 F24 N, C20 H30 Fe
Sum formula	C57 H30 B2 Br6 F24 Fe N	C57 H30 B2 Br6 F24 Fe N
Mr	1741.69	1741.75
Dx,g cm-3	1.967	1.967
Z	4	4
Mu (mm-1)	4.450	4.450
F000	3364.0	3364.0
F000’	3360.97	
h,k,lmax	16,28,22	16,28,22
Nref	5595	5584
Tmin,Tmax	0.189,0.280	0.237,0.376
Tmin’	0.147	

Correction method= # Reported T Limits: Tmin=0.237 Tmax=0.376
AbsCorr = NUMERICAL

```
Data completeness= 0.998          Theta(max)= 25.682

R(reflections)= 0.0251( 5007)          wR2(reflections)=
                                       0.0585( 5584)
S = 1.051                 Npar= 416
```

The following ALERTS were generated. Each ALERT has the format
test-name_ALERT_alert-type_alert-level.
Click on the hyperlinks for more details of the test.

Alert level A

```
PLAT430_ALERT_2_A Short Inter D...A Contact  N1       ..N1       .       1.14 Ang.
                                              1-x,y,3/2-z  =      2_656 Check
```

Alert level C

```
PLAT042_ALERT_1_C Calc. and Reported MoietyFormula Strings  Differ     Please Check
              Calc: 2(C18.50 B Br3 F12 N0.50), C20 H30 Fe
              Rep.: B2 Br6 C37 F24 N, C20 H30 Fe
PLAT911_ALERT_3_C Missing FCF Refl Between Thmin & STh/L=    0.600          5 Report
                0  0  2,   2  6  2,  -2  0  4,  -2  4  4,   2 26  7,
```

Alert level G

```
PLAT083_ALERT_2_G SHELXL Second Parameter in WGHT  Unusually Large      14.56 Why ?
PLAT168_ALERT_4_G The CIF-Embedded .res File Contains EXYZ Records          1 Report
PLAT171_ALERT_4_G The CIF-Embedded .res File Contains EADP Records          1 Report
PLAT299_ALERT_4_G Atom Site Occupancy Constrained at ............         0.5 Check
              N1      C1
PLAT301_ALERT_3_G Main Residue  Disorder .............(Resd    1)          3% Note
PLAT315_ALERT_2_G Singly Bonded Carbon Detected (H-atoms Missing).          C1 Check
PLAT432_ALERT_2_G Short Inter X...Y Contact  N1       ..B1       .       2.73 Ang.
                                              1-x,y,3/2-z  =      2_656 Check
PLAT432_ALERT_2_G Short Inter X...Y Contact  C1       ..B1       .       2.73 Ang.
                                              1-x,y,3/2-z  =      2_656 Check
PLAT434_ALERT_2_G Short Inter HL..HL Contact Br11     ..Br12     .       3.56 Ang.
                                           -1/2+x,1/2+y,z  =      3_455 Check
PLAT434_ALERT_2_G Short Inter HL..HL Contact F112     ..F113     .       2.80 Ang.
                                          1/2-x,3/2-y,1-z  =      7_566 Check
PLAT720_ALERT_4_G Number of Unusual/Non-Standard Labels ..........         26 Note
              Fe04    C00I    C00L    C00U    C00V    C013    C015    H01A
              H01B    H01C    C016    H01D    H01E    H01F    C017    H01G
              H01H    H01I    C018    H01J    H01K    H01L    C019    H01M
              H01N    H01O
PLAT794_ALERT_5_G Tentative Bond Valency for Fe04      (II)      .       1.83 Info
PLAT910_ALERT_3_G Missing FCF Reflection(s) Below Theta(Min)[Deg]=       2.03 Note
                1  1  0,   0  2  0,
PLAT912_ALERT_4_G Missing # of FCF Reflections Above STh/L=  0.600          4 Note
PLAT913_ALERT_3_G Missing # of Very Strong Reflections in FCF ....          2 Note
                2  6  2,  -2  0  4,
PLAT933_ALERT_2_G Number of HKL-OMIT Records in Embedded .res File          2 Note
                0  0  2,   5 27  2,
PLAT969_ALERT_5_G The 'Henn et al.' R-Factor-gap value ..........      1.612 Note
```

```
              Predicted wR2: Based on SigI**2  3.63 or SHELX Weight  5.57
PLAT978_ALERT_2_G Number C-C Bonds with Positive Residual Density.        11 Info
PLAT994_ALERT_1_G SHELXL .ins Contains no or MERG 0 Instruction ..         ! Note
```

```
   1 ALERT level A = Most likely a serious problem - resolve or explain
   0 ALERT level B = A potentially serious problem, consider carefully
   2 ALERT level C = Check. Ensure it is not caused by an omission or oversight
  19 ALERT level G = General information/check it is not something unexpected

   2 ALERT type 1 CIF construction/syntax error, inconsistent or missing data
   9 ALERT type 2 Indicator that the structure model may be wrong or deficient
   4 ALERT type 3 Indicator that the structure quality may be low
   5 ALERT type 4 Improvement, methodology, query or suggestion
   2 ALERT type 5 Informative message, check
```

It is advisable to attempt to resolve as many as possible of the alerts in all categories. Often the minor alerts point to easily fixed oversights, errors and omissions in your CIF or refinement strategy, so attention to these fine details can be worthwhile. It is up to the individual to critically assess their own results and, if necessary, seek expert advice.

Validation response form

Please find below a validation response form (VRF) that can be filled in and pasted into your CIF.

```
# start Validation Reply Form
_vrf_PLAT430_c2c_neu
;
PROBLEM: Short Inter D...A Contact  N1       ..N1       .       1.14 Ang.
RESPONSE: ...
;
_vrf_PLAT042_c2c_neu
;
PROBLEM: Calc. and Reported MoietyFormula Strings  Differ     Please Check
RESPONSE: ...
;
_vrf_PLAT911_c2c_neu
;
PROBLEM: Missing FCF Refl Between Thmin & STh/L=    0.600          5 Report
RESPONSE: ...
;
# end Validation Reply Form
```

PLATON version of 15/01/2026; check.def file version of 02/01/2026

---

duplicate check

No duplication found

Datablock c2c_neu - ellipsoid plot

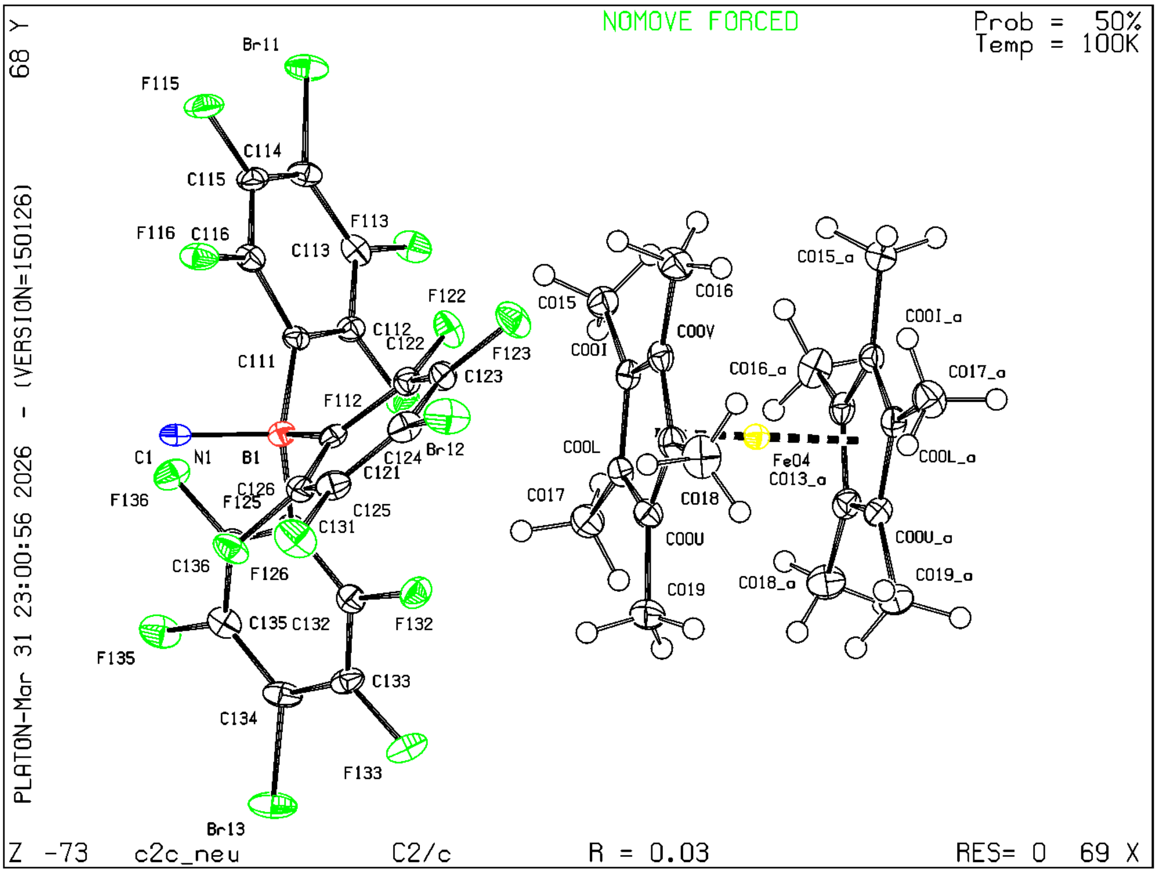

Supplement: Supplementary file 2 [file CHEM-32-e71310-s001.zip › chem71310-sup-0002-cif/ferrocenium.pdf]
