# Supplementary material for: Expanding Cyanide‐Bridged Weakly Coordinating Anions Through the Brominated Silver Salt Ag[BCNBBr]
Source: Chemistry. 2026 Jun 27;32(38):e71310. doi: 10.1002/chem.71310 (PMC13660483; doi:10.1002/chem.71310)

checkCIF/PLATON report

Structure factors have been supplied for datablock(s) mo_mm_tns_am393_0m

THIS REPORT IS FOR GUIDANCE ONLY. IF USED AS PART OF A REVIEW PROCEDURE FOR PUBLICATION, IT SHOULD NOT REPLACE THE EXPERTISE OF AN EXPERIENCED CRYSTALLOGRAPHIC REFEREE.

No syntax errors found. CIF dictionary Interpreting this report

Datablock: mo_mm_tns_am393_0m

Bond precision:	C-C = 0.0063 A		Wavelength=0.71073
Cell:	a=11.7051(4)	b=15.6805(6)	c=22.2282(8)
	alpha=73.720(1)	beta=82.861(1)	gamma=68.899(1)
Temperature:	100 K		

	Calculated	Reported
Volume	3652.4(2)	3652.4(2)
Space group	P -1	P -1
Hall group	-P 1	-P 1
Moiety formula	C37 B2 Br6 F24 N, C18 H11.75 Br3 N, 4(C H2 Cl2) [+ solvent]	B2 Br6 C37 F24 N, C18 H11.75 Br3 N, 4(C H2 Cl2), 0.4[CH2CL2]
Sum formula	C59 H19.75 B2 Br9 Cl8 F24 N2 [+ solvent]	C59.40 H20.55 B2 Br9 Cl8.80 F24 N2
Mr	2236.84	2270.90
Dx,g cm-3	2.034	2.065
Z	2	2
Mu (mm-1)	5.334	5.364
F000	2129.5	2163.0
F000'	2128.15	
h,k,lmax	14,19,27	14,19,27
Nref	13919	13882
Tmin,Tmax	0.331,0.781	0.298,0.623
Tmin'	0.246	

Correction method= # Reported T Limits: Tmin=0.298 Tmax=0.623
AbsCorr = NUMERICAL

Data completeness= 0.997   Theta(max)= 25.712

R(reflections)= 0.0395(10958)   wR2(reflections)= 0.0858(13882)

S = 1.034   Npar= 1145

The following ALERTS were generated. Each ALERT has the format
test-name_ALERT_alert-type_alert-level.
Click on the hyperlinks for more details of the test.

Alert level B

```
PLAT213_ALERT_2_B Atom Br1            has ADP max/min Ratio .....        4.3 prolat
```

Alert level C

```
PLAT077_ALERT_4_C Unit-Cell Contains Non-integer Number of Atoms .     Please Check
PLAT213_ALERT_2_C Atom Br23           has ADP max/min Ratio .....        3.2 prolat
PLAT213_ALERT_2_C Atom F233           has ADP max/min Ratio .....        3.9 prolat
PLAT213_ALERT_2_C Atom F1             has ADP max/min Ratio .....        3.8 prolat
PLAT213_ALERT_2_C Atom F8             has ADP max/min Ratio .....        4.0 oblate
PLAT213_ALERT_2_C Atom C135           has ADP max/min Ratio .....        3.2 oblate
PLAT213_ALERT_2_C Atom C4             has ADP max/min Ratio .....        3.2 prolat
PLAT220_ALERT_2_C NonSolvent   Resd 1 Br   Ueq(max)/Ueq(min) Range        3.3 Ratio
PLAT220_ALERT_2_C NonSolvent   Resd 1  F   Ueq(max)/Ueq(min) Range        4.6 Ratio
PLAT245_ALERT_2_C U(iso) H15B      Smaller than U(eq) C15       by      0.015 Ang**2
PLAT341_ALERT_3_C Low Bond Precision on  C-C Bonds ..............    0.00634 Ang.
PLAT910_ALERT_3_C Missing FCF Reflection(s) Below Theta(Min)[Deg]=      1.93 Note
                1  0  0,   0  1  0,   0 -1  1,   0  0  1,   0  1  1,   0  0  2,
PLAT911_ALERT_3_C Missing FCF Refl Between Thmin & STh/L=    0.600          2 Report
              -11  0  3,  -5  5 22,
```

Alert level G

```
FORMU01_ALERT_2_G  There is a discrepancy between the atom counts in the
            _chemical_formula_sum and the formula from the _atom_site* data.
            Atom count from _chemical_formula_sum:C59.4 H20.55 B2 Br9 Cl8.8 F24 N2
            Atom count from the _atom_site data:  C59 H19.75 B2 Br9 Cl8 F24 N2
CELLZ01_ALERT_1_G Difference between formula and atom_site contents detected.
CELLZ01_ALERT_1_G ALERT: Large difference may be due to a
            symmetry error - see SYMMG tests
           From the CIF: _cell_formula_units_Z    2
           From the CIF: _chemical_formula_sum  C59.40 H20.55 B2 Br9 Cl8.80 F24 N2
           TEST: Compare cell contents of formula and atom_site data

           atom    Z*formula  cif sites diff
           C        118.80    118.00    0.80
           H         41.10     39.50    1.60
           B          4.00      4.00    0.00
           Br        18.00     18.00    0.00
```

```
           Cl        17.60     16.00    1.60
           F         48.00     48.00    0.00
           N          4.00      4.00    0.00
PLAT003_ALERT_2_G Number of Uiso or U(i,j)  Restrained non-H-Atoms         22 Report
PLAT041_ALERT_1_G Calc. and Reported SumFormula    Strings  Differ     Please Check
              Calc: C59 H19.75 B2 Br9 Cl8 F24 N2
              Rep.: C59.40 H20.55 B2 Br9 Cl8.80 F24 N2
PLAT042_ALERT_1_G Calc. and Reported MoietyFormula Strings  Differ     Please Check
              Calc: C37 B2 Br6 F24 N, C18 H11.75 Br3 N, 4(C H2 Cl2
              Rep.: B2 Br6 C37 F24 N, C18 H11.75 Br3 N, 4(C H2 Cl2
                    ), 0.4[CH2CL2]
PLAT051_ALERT_1_G Mu(calc) and Mu(cif) Ratio Differs from 1.0 by .       0.57 %
PLAT083_ALERT_2_G SHELXL Second Parameter in WGHT  Unusually Large       9.69 Why ?
PLAT093_ALERT_1_G No s.u.'s on H-positions, Refinement Reported as      mixed Check
PLAT154_ALERT_1_G The s.u.'s on the Cell Angles are Equal ..(Note)      0.001 Degree
PLAT168_ALERT_4_G The CIF-Embedded .res File Contains EXYZ Records          2 Report
PLAT171_ALERT_4_G The CIF-Embedded .res File Contains EADP Records          1 Report
PLAT186_ALERT_4_G The CIF-Embedded .res File Contains ISOR Records          4 Report
PLAT187_ALERT_4_G The CIF-Embedded .res File Contains RIGU Records          4 Report
PLAT299_ALERT_4_G Atom Site Occupancy Constrained at ............         0.5 Check
              N1      N2      C1      C2
PLAT299_ALERT_4_G Atom Site Occupancy Constrained at ............        0.25 Check
              Br1     F1      F2      F3      C3      C4      C5      C6
PLAT300_ALERT_4_G Atom Site Occupancy of Br12       Constrained at       0.75 Check
PLAT300_ALERT_4_G Atom Site Occupancy of F123       Constrained at       0.75 Check
PLAT300_ALERT_4_G Atom Site Occupancy of F125       Constrained at       0.75 Check
PLAT300_ALERT_4_G Atom Site Occupancy of F126       Constrained at       0.75 Check
PLAT300_ALERT_4_G Atom Site Occupancy of C123       Constrained at       0.75 Check
PLAT300_ALERT_4_G Atom Site Occupancy of C124       Constrained at       0.75 Check
PLAT300_ALERT_4_G Atom Site Occupancy of C125       Constrained at       0.75 Check
PLAT300_ALERT_4_G Atom Site Occupancy of C126       Constrained at       0.75 Check
PLAT300_ALERT_4_G Atom Site Occupancy of H02C       Constrained at       0.75 Check
PLAT301_ALERT_3_G Main Residue  Disorder .............(Resd    1)         27% Note
PLAT302_ALERT_4_G Anion/Solvent/Minor-Residue Disorder (Resd    3)        100% Note
PLAT302_ALERT_4_G Anion/Solvent/Minor-Residue Disorder (Resd    6)        100% Note
PLAT302_ALERT_4_G Anion/Solvent/Minor-Residue Disorder (Resd    7)        100% Note
PLAT302_ALERT_4_G Anion/Solvent/Minor-Residue Disorder (Resd    8)        100% Note
PLAT304_ALERT_4_G Non-Integer Number of Atoms in ..... (Resd    2)      33.75 Check
PLAT304_ALERT_4_G Non-Integer Number of Atoms in ..... (Resd    3)       2.75 Check
PLAT304_ALERT_4_G Non-Integer Number of Atoms in ..... (Resd    6)       2.60 Check
PLAT304_ALERT_4_G Non-Integer Number of Atoms in ..... (Resd    7)       2.40 Check
PLAT304_ALERT_4_G Non-Integer Number of Atoms in ..... (Resd    8)       2.25 Check
PLAT335_ALERT_2_G Check Large C6 Ring C-C Range C131     -C136           0.20 Ang.
PLAT335_ALERT_2_G Check Large C6 Ring C-C Range C131     -C7             0.16 Ang.
PLAT432_ALERT_2_G Short Inter X...Y Contact  F222     ..C02Y     .       2.89 Ang.
                                                    x,y,z  =      1_555 Check
PLAT432_ALERT_2_G Short Inter X...Y Contact  F223     ..C7       .       2.88 Ang.
                                                  1+x,y,z  =      1_655 Check
PLAT434_ALERT_2_G Short Inter HL..HL Contact Br03     ..Cl2      .       3.48 Ang.
                                              2-x,2-y,1-z  =      2_776 Check
PLAT434_ALERT_2_G Short Inter HL..HL Contact Br03     ..Br23     .       3.55 Ang.
                                              2-x,1-y,1-z  =      2_766 Check
PLAT434_ALERT_2_G Short Inter HL..HL Contact Br05     ..F2       .       3.04 Ang.
                                                  x,y,1+z  =      1_556 Check
PLAT434_ALERT_2_G Short Inter HL..HL Contact Br05     ..F126     .       3.05 Ang.
                                                  x,y,1+z  =      1_556 Check
PLAT434_ALERT_2_G Short Inter HL..HL Contact Br06     ..F215     .       2.98 Ang.
```

```
                                                     x,y,z  =      1_555 Check
PLAT434_ALERT_2_G Short Inter HL..HL Contact Br11     ..F213     .       3.03 Ang.
                                                -x,1-y,1-z  =      2_566 Check
PLAT434_ALERT_2_G Short Inter HL..HL Contact Br21     ..F236     .       2.96 Ang.
                                               1-x,1-y,1-z  =      2_666 Check
PLAT434_ALERT_2_G Short Inter HL..HL Contact Br22     ..F1       .       3.19 Ang.
                                                1-x,2-y,-z  =      2_675 Check
PLAT434_ALERT_2_G Short Inter HL..HL Contact Cl0B     ..Cl0E     .       3.29 Ang.
                                                  -1+x,y,z  =      1_455 Check
PLAT434_ALERT_2_G Short Inter HL..HL Contact F113     ..F236     .       2.69 Ang.
                                                  -1+x,y,z  =      1_455 Check
PLAT434_ALERT_2_G Short Inter HL..HL Contact F113     ..F216     .       2.83 Ang.
                                                  -1+x,y,z  =      1_455 Check
PLAT434_ALERT_2_G Short Inter HL..HL Contact F223     ..F4       .       2.72 Ang.
                                                   1+x,y,z  =      1_655 Check
PLAT605_ALERT_4_G Largest Solvent Accessible VOID in the Structure        106 A**3
PLAT720_ALERT_4_G Number of Unusual/Non-Standard Labels ..........         58 Note
              Br03    Br05    Br06    Cl0A    Cl0B    Cl0C    Cl0D    Cl0E
              Cl0F    Cl0G    Cl0H    N013    C01C    C01D    C01K    C01L
              C01Q    H01Q    C01T    H01T    C01U    H01U    C01V    H01V
              C01Z    H01Z    C020    H020    C023    H023    C02A    H02A
              C02B    H02B    C02C    H02C    C02D    C02F    H02F    C02J
              C02K    H02K    C02N    H02D    H02E    C02W    H02G    H02H
              C02X    H02I    H02J    C02Y    H02L    H02M    C02Z    H02N
              H02O    Cl0I
PLAT860_ALERT_3_G Number of Least-Squares Restraints ............        186 Note
PLAT868_ALERT_4_G ALERTS Due to the Use of _smtbx_masks Suppressed          ! Info
PLAT912_ALERT_4_G Missing # of FCF Reflections Above STh/L=  0.600         29 Note
PLAT941_ALERT_3_G Average HKL Measurement Multiplicity ..........         4.9 Low
PLAT969_ALERT_5_G The 'Henn et al.' R-Factor-gap value ..........       1.993 Note
              Predicted wR2: Based on SigI**2  4.31 or SHELX Weight  8.30
PLAT978_ALERT_2_G Number C-C Bonds with Positive Residual Density.          0 Info
PLAT994_ALERT_1_G SHELXL .ins Contains no or MERG 0 Instruction ..          ! Note
```

```
   0 ALERT level A = Most likely a serious problem - resolve or explain
   1 ALERT level B = A potentially serious problem, consider carefully
  13 ALERT level C = Check. Ensure it is not caused by an omission or oversight
  60 ALERT level G = General information/check it is not something unexpected

   8 ALERT type 1 CIF construction/syntax error, inconsistent or missing data
  30 ALERT type 2 Indicator that the structure model may be wrong or deficient
   6 ALERT type 3 Indicator that the structure quality may be low
  29 ALERT type 4 Improvement, methodology, query or suggestion
   1 ALERT type 5 Informative message, check
```

Validation response form

Please find below a validation response form (VRF) that can be filled in and pasted into your CIF.

```
# start Validation Reply Form
_vrf_PLAT213_mo_mm_tns_am393_0m
;
PROBLEM: Atom Br1             has ADP max/min Ratio .....        4.3 prolat
RESPONSE: ...
;
```

```
_vrf_PLAT077_mo_mm_tns_am393_0m
;
PROBLEM: Unit-Cell Contains Non-integer Number of Atoms .     Please Check
RESPONSE: ...
;
_vrf_PLAT220_mo_mm_tns_am393_0m
;
PROBLEM: NonSolvent   Resd 1 Br   Ueq(max)/Ueq(min) Range        3.3 Ratio
RESPONSE: ...
;
_vrf_PLAT245_mo_mm_tns_am393_0m
;
PROBLEM: U(iso) H15B      Smaller than U(eq) C15       by      0.015 Ang**2
RESPONSE: ...
;
_vrf_PLAT341_mo_mm_tns_am393_0m
;
PROBLEM: Low Bond Precision on  C-C Bonds ...............    0.00634 Ang.
RESPONSE: ...
;
_vrf_PLAT910_mo_mm_tns_am393_0m
;
PROBLEM: Missing FCF Reflection(s) Below Theta(Min)[Deg]=       1.93 Note
RESPONSE: ...
;
_vrf_PLAT911_mo_mm_tns_am393_0m
;
PROBLEM: Missing FCF Refl Between Thmin & STh/L=    0.600          2 Report
RESPONSE: ...
;
# end Validation Reply Form
```

It is advisable to attempt to resolve as many as possible of the alerts in all categories. Often the minor alerts point to easily fixed oversights, errors and omissions in your CIF or refinement strategy, so attention to these fine details can be worthwhile. It is up to the individual to critically assess their own results and, if necessary, seek expert advice.

PLATON version of 15/01/2026; check.def file version of 02/01/2026

---

duplicate check

No duplication found

Datablock mo_mm_tns_am393_0m - ellipsoid plot

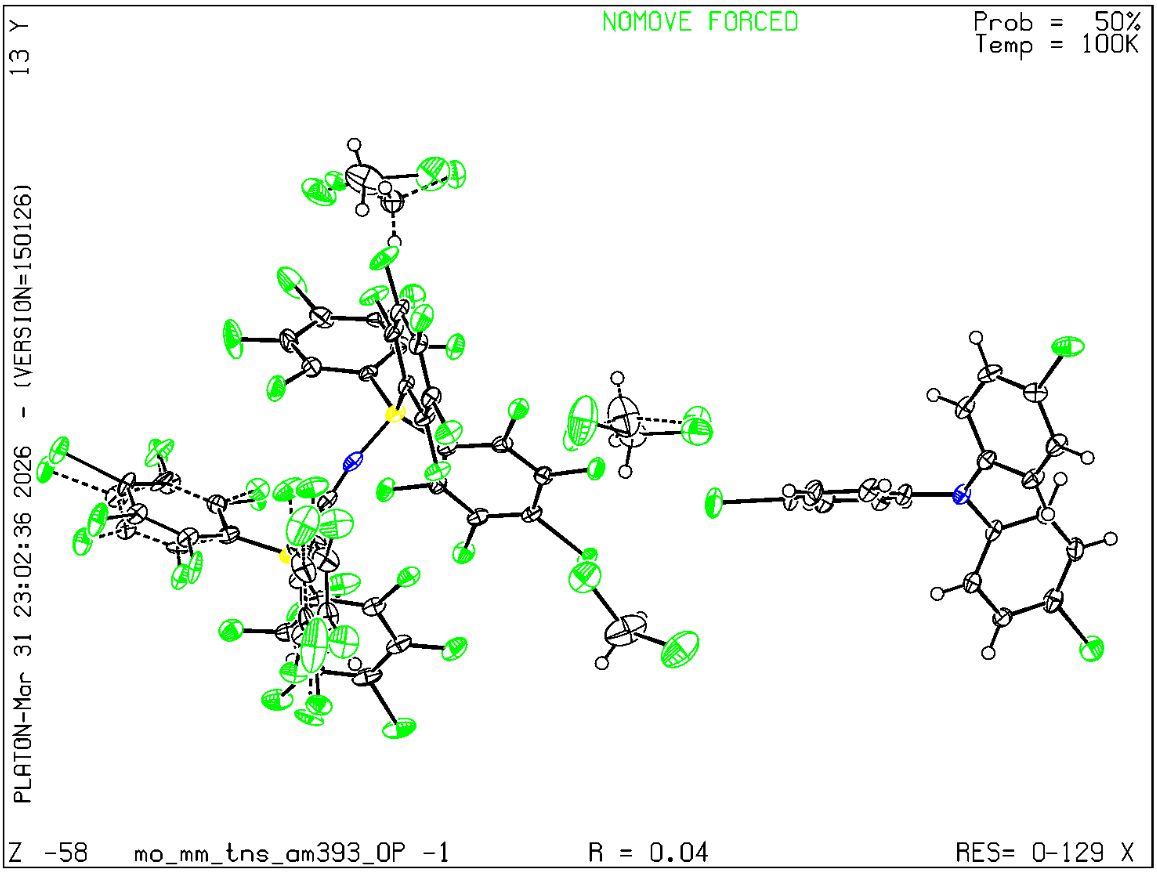

Supplement: Supplementary file 2 [file CHEM-32-e71310-s001.zip › chem71310-sup-0002-cif/magic blue.pdf]
