# Supplementary material for: Expanding Cyanide‐Bridged Weakly Coordinating Anions Through the Brominated Silver Salt Ag[BCNBBr]
Source: Chemistry. 2026 Jun 27;32(38):e71310. doi: 10.1002/chem.71310 (PMC13660483; doi:10.1002/chem.71310)

checkCIF/PLATON report

Structure factors have been supplied for datablock(s) p-1_final3

THIS REPORT IS FOR GUIDANCE ONLY. IF USED AS PART OF A REVIEW PROCEDURE FOR PUBLICATION, IT SHOULD NOT REPLACE THE EXPERTISE OF AN EXPERIENCED CRYSTALLOGRAPHIC REFEREE.

No syntax errors found. CIF dictionary Interpreting this report

Datablock: p-1_final3

Bond precision:	C-C = 0.0211 A		Wavelength=0.71073
Cell:	a=13.0905(13)	b=13.2238(13)	c=22.269(2)
	alpha=87.250(4)	beta=77.878(4)	gamma=60.607(3)
Temperature:	100 K		

	Calculated	Reported
Volume	3276.9(6)	3276.9(6)
Space group	P -1	P -1
Hall group	-P 1	-P 1
Moiety formula	C37 B2 Br6 F24 N, C18 H9 Br6 N [+ solvent]	B2 Br6 C37 F24 N, C18 H9 Br6 N, 1.1[C5H12]
Sum formula	C55 H9 B2 Br12 F24 N2 [+ solvent]	C60.50 H22.20 B2 Br12 F24 N2
Mr	2134.06	2213.54
Dx,g cm-3	2.163	2.243
Z	2	2
Mu (mm-1)	7.442	7.446
F000	1998.0	2090.0
F000’	1992.83	
h,k,lmax	15,15,26	15,15,26
Nref	12096	11973
Tmin,Tmax	0.005,0.101	0.207,0.745
Tmin’	0.000	

```
Correction method= # Reported T Limits: Tmin=0.207 Tmax=0.745
AbsCorr = MULTI-SCAN

Data completeness= 0.990            Theta(max)= 25.438

                                                        wR2(reflections)=
R(reflections)= 0.0997( 9402)                           0.2507( 11973)

S = 1.122                    Npar= 808
```

The following ALERTS were generated. Each ALERT has the format
test-name_ALERT_alert-type_alert-level.
Click on the hyperlinks for more details of the test.

Alert level B

```
PLAT213_ALERT_2_B Atom C236            has ADP max/min Ratio .....        4.1 oblate
PLAT341_ALERT_3_B Low Bond Precision on  C-C Bonds ..............    0.02107 Ang.
PLAT971_ALERT_2_B Check Calcd Resid. Dens.  0.92Ang From C114           3.16 eA-3
PLAT971_ALERT_2_B Check Calcd Resid. Dens.  0.06Ang From F112           3.08 eA-3
PLAT971_ALERT_2_B Check Calcd Resid. Dens.  1.46Ang From F212           2.82 eA-3
PLAT971_ALERT_2_B Check Calcd Resid. Dens.  1.53Ang From F116           2.78 eA-3
```

Alert level C

```
DIFMX02_ALERT_1_C  The maximum difference density is > 0.1*ZMAX*0.75
            The relevant atom site should be identified.
PLAT097_ALERT_2_C Large Reported Max.  (Positive) Residual Density       3.07 eA-3
PLAT213_ALERT_2_C Atom C135            has ADP max/min Ratio .....        3.3 prolat
PLAT434_ALERT_2_C Short Inter HL..HL Contact F135      ..F135     .      2.53 Ang.
                                                 -x,2-y,1-z  =      2_576 Check
PLAT906_ALERT_3_C Large K Value in the Analysis of Variance ......      5.020 Check
PLAT906_ALERT_3_C Large K Value in the Analysis of Variance ......      2.148 Check
PLAT910_ALERT_3_C Missing FCF Reflection(s) Below Theta(Min)[Deg]=       1.95 Note
                1  0  0,   0  1  0,   1  1  0,   0  0  1,   1  0  1,   1  1  1,
                0  0  2,
PLAT911_ALERT_3_C Missing FCF Refl Between Thmin & STh/L=    0.600         35 Report
                2 -1  1,  -8-15  4,  -7-14  4,  -6  0  4,  -2 12  4,  -8-15  5,
              -10  1  5,  -7-14  6,  -7-13  6,  -7-14  7,  -6-13  7,  -5-12  7,
               -3-14  8,  -6-13  8,  -6-12  8,  -5-11  8,  -4-10  8,  -6-12  9,
               -5-12  9,   7 -4 10,  -7  4 10,  -5  8 10,   6  3 12,  -7  3 13,
               -6  3 14,  -3  7 14,  -2 -4 15,  10  3 16,  -6  4 16,  -6  4 17,
                   (    5 More NOT listed: see .ckf listing file)
PLAT971_ALERT_2_C Check Calcd Resid. Dens.  1.90Ang From Br0B           2.46 eA-3
PLAT971_ALERT_2_C Check Calcd Resid. Dens.  2.05Ang From Br09           2.42 eA-3
PLAT971_ALERT_2_C Check Calcd Resid. Dens.  1.44Ang From F236           2.35 eA-3
PLAT971_ALERT_2_C Check Calcd Resid. Dens.  1.10Ang From Br0B           2.34 eA-3
PLAT971_ALERT_2_C Check Calcd Resid. Dens.  0.76Ang From N1             2.30 eA-3
PLAT971_ALERT_2_C Check Calcd Resid. Dens.  1.69Ang From Br08           2.28 eA-3
PLAT971_ALERT_2_C Check Calcd Resid. Dens.  1.47Ang From C215           2.27 eA-3
PLAT971_ALERT_2_C Check Calcd Resid. Dens.  1.44Ang From F223           2.17 eA-3
PLAT971_ALERT_2_C Check Calcd Resid. Dens.  1.54Ang From C234           2.15 eA-3
PLAT971_ALERT_2_C Check Calcd Resid. Dens.  0.68Ang From F122           2.10 eA-3
PLAT971_ALERT_2_C Check Calcd Resid. Dens.  0.78Ang From Br09           1.84 eA-3
```

```
PLAT971_ALERT_2_C Check Calcd Resid. Dens.  1.13Ang From Br12           1.62 eA-3
PLAT971_ALERT_2_C Check Calcd Resid. Dens.  1.27Ang From C01V           1.59 eA-3
PLAT971_ALERT_2_C Check Calcd Resid. Dens.  1.20Ang From Br0C           1.57 eA-3
PLAT971_ALERT_2_C Check Calcd Resid. Dens.  0.98Ang From Br23           1.52 eA-3
PLAT971_ALERT_2_C Check Calcd Resid. Dens.  0.86Ang From C214           1.52 eA-3
PLAT972_ALERT_2_C Check Calcd Resid. Dens.  0.76Ang From Br0B          -1.83 eA-3
PLAT972_ALERT_2_C Check Calcd Resid. Dens.  0.98Ang From Br0C          -1.79 eA-3
PLAT972_ALERT_2_C Check Calcd Resid. Dens.  0.94Ang From Br21          -1.66 eA-3
PLAT972_ALERT_2_C Check Calcd Resid. Dens.  0.79Ang From Br22          -1.62 eA-3
PLAT972_ALERT_2_C Check Calcd Resid. Dens.  0.83Ang From Br05          -1.62 eA-3
PLAT972_ALERT_2_C Check Calcd Resid. Dens.  0.73Ang From Br09          -1.62 eA-3
PLAT972_ALERT_2_C Check Calcd Resid. Dens.  0.87Ang From Br21          -1.61 eA-3
PLAT972_ALERT_2_C Check Calcd Resid. Dens.  0.90Ang From Br21          -1.59 eA-3
PLAT972_ALERT_2_C Check Calcd Resid. Dens.  0.50Ang From Br07          -1.56 eA-3
PLAT972_ALERT_2_C Check Calcd Resid. Dens.  0.85Ang From Br08          -1.55 eA-3
PLAT972_ALERT_2_C Check Calcd Resid. Dens.  0.73Ang From Br09          -1.52 eA-3
PLAT972_ALERT_2_C Check Calcd Resid. Dens.  0.94Ang From Br07          -1.52 eA-3
PLAT977_ALERT_2_C Check Negative Difference Density on H01Z      .     -0.40 eA-3
```

Alert level G

```
FORMU01_ALERT_2_G  There is a discrepancy between the atom counts in the
            _chemical_formula_sum and the formula from the _atom_site* data.
            Atom count from _chemical_formula_sum:C60.5 H22.2 B2 Br12 F24 N2
            Atom count from the _atom_site data:  C55 H9 B2 Br12 F24 N2
CELLZ01_ALERT_1_G Difference between formula and atom_site contents detected.
CELLZ01_ALERT_1_G ALERT: Large difference may be due to a
            symmetry error - see SYMMG tests
           From the CIF: _cell_formula_units_Z    2
           From the CIF: _chemical_formula_sum  C60.50 H22.20 B2 Br12 F24 N2
           TEST: Compare cell contents of formula and atom_site data

           atom    Z*formula  cif sites diff
           C        121.00    110.00   11.00
           H         44.40     18.00   26.40
           B          4.00      4.00    0.00
           Br        24.00     24.00    0.00
           F         48.00     48.00    0.00
           N          4.00      4.00    0.00
PLAT003_ALERT_2_G Number of Uiso or U(i,j)  Restrained non-H-Atoms        27 Report
PLAT041_ALERT_1_G Calc. and Reported SumFormula    Strings  Differ     Please Check
             Calc: C55 H9 B2 Br12 F24 N2
             Rep.: C60.50 H22.20 B2 Br12 F24 N2
PLAT042_ALERT_1_G Calc. and Reported MoietyFormula Strings  Differ     Please Check
             Calc: C37 B2 Br6 F24 N, C18 H9 Br6 N
             Rep.: B2 Br6 C37 F24 N, C18 H9 Br6 N, 1.1[C5H12]
PLAT063_ALERT_4_G Crystal Size Possibly too Large for Beam Size ..       1.00 mm
PLAT083_ALERT_2_G SHELXL Second Parameter in WGHT  Unusually Large     141.14 Why ?
PLAT168_ALERT_4_G The CIF-Embedded .res File Contains EXYZ Records          2 Report
PLAT171_ALERT_4_G The CIF-Embedded .res File Contains EADP Records          6 Report
PLAT186_ALERT_4_G The CIF-Embedded .res File Contains ISOR Records          5 Report
PLAT187_ALERT_4_G The CIF-Embedded .res File Contains RIGU Records          3 Report
PLAT299_ALERT_4_G Atom Site Occupancy Constrained at ............         0.5 Check
             N1      N2      C1      C2
PLAT301_ALERT_3_G Main Residue  Disorder ..............(Resd    1)         3% Note
PLAT432_ALERT_2_G Short Inter X...Y Contact  F212      ..C225     .       2.97 Ang.
                                                1-x,1-y,-z  =      2_665 Check
```

```
PLAT432_ALERT_2_G Short Inter X...Y Contact  F226     ..C235     .       2.93 Ang.
                                              1-x,1-y,-z  =     2_665 Check
PLAT432_ALERT_2_G Short Inter X...Y Contact  F236     ..C213     .       2.93 Ang.
                                              1-x,1-y,-z  =     2_665 Check
PLAT434_ALERT_2_G Short Inter HL..HL Contact Br05     ..F223     .       3.19 Ang.
                                                 1+x,y,z  =     1_655 Check
PLAT434_ALERT_2_G Short Inter HL..HL Contact Br07     ..F215     .       3.20 Ang.
                                                 x,1+y,z  =     1_565 Check
PLAT434_ALERT_2_G Short Inter HL..HL Contact Br08     ..F233     .       3.21 Ang.
                                                   x,y,z  =     1_555 Check
PLAT434_ALERT_2_G Short Inter HL..HL Contact Br12     ..F113     .       3.21 Ang.
                                                -1+x,y,z  =     1_455 Check
PLAT606_ALERT_4_G Solvent Accessible VOID(S) in  Crystal Structure          ! Info
PLAT720_ALERT_4_G Number of Unusual/Non-Standard Labels ..........         34 Note
              Br05    Br07    Br08    Br09    Br0B    Br0C    N015    C01D
              C01H    C01M    H01M    C01Q    C01R    C01V    H01V    C01X
              C01Z    H01Z    C020    C021    C022    H022    C028    C02A
              C02C    H02C    C02H    H02H    C02J    H02J    C02K    H02K
              C02N    H02N
PLAT860_ALERT_3_G Number of Least-Squares Restraints ............        234 Note
PLAT868_ALERT_4_G ALERTS Due to the Use of _smtbx_masks Suppressed          ! Info
PLAT912_ALERT_4_G Missing # of FCF Reflections Above STh/L=  0.600         81 Note
PLAT969_ALERT_5_G The 'Henn et al.' R-Factor-gap value ..........      4.685 Note
              Predicted wR2: Based on SigI**2  5.35 or SHELX Weight 22.34
PLAT978_ALERT_2_G Number C-C Bonds with Positive Residual Density.          0 Info
PLAT994_ALERT_1_G SHELXL .ins Contains no or MERG 0 Instruction ..          ! Note
```

```
   0 ALERT level A = Most likely a serious problem - resolve or explain
   6 ALERT level B = A potentially serious problem, consider carefully
  37 ALERT level C = Check. Ensure it is not caused by an omission or oversight
  29 ALERT level G = General information/check it is not something unexpected

   6 ALERT type 1 CIF construction/syntax error, inconsistent or missing data
  48 ALERT type 2 Indicator that the structure model may be wrong or deficient
   7 ALERT type 3 Indicator that the structure quality may be low
  10 ALERT type 4 Improvement, methodology, query or suggestion
   1 ALERT type 5 Informative message, check
```

Validation response form

Please find below a validation response form (VRF) that can be filled in and pasted into your CIF.

```
# start Validation Reply Form
_vrf_DIFMX02_p-1_final3
;
PROBLEM: The maximum difference density is > 0.1*ZMAX*0.75
RESPONSE: ...
;
_vrf_PLAT213_p-1_final3
;
PROBLEM: Atom C236            has ADP max/min Ratio .....        4.1 oblate
RESPONSE: ...
;
_vrf_PLAT341_p-1_final3
;
```

```
PROBLEM: Low Bond Precision on  C-C Bonds ..............    0.02107 Ang.
RESPONSE: ...
;
_vrf_PLAT971_p-1_final3
;
PROBLEM: Check Calcd Resid. Dens.  0.92Ang From C114            3.16 eA-3
RESPONSE: ...
;
_vrf_PLAT097_p-1_final3
;
PROBLEM: Large Reported Max.  (Positive) Residual Density       3.07 eA-3
RESPONSE: ...
;
_vrf_PLAT434_p-1_final3
;
PROBLEM: Short Inter HL..HL Contact F135     ..F135     .       2.53 Ang.
RESPONSE: ...
;
_vrf_PLAT906_p-1_final3
;
PROBLEM: Large K Value in the Analysis of Variance ......      5.020 Check
RESPONSE: ...
;
_vrf_PLAT910_p-1_final3
;
PROBLEM: Missing FCF Reflection(s) Below Theta(Min)[Deg]=       1.95 Note
RESPONSE: ...
;
_vrf_PLAT911_p-1_final3
;
PROBLEM: Missing FCF Refl Between Thmin & STh/L=    0.600         35 Report
RESPONSE: ...
;
_vrf_PLAT972_p-1_final3
;
PROBLEM: Check Calcd Resid. Dens.  0.76Ang From Br0B           -1.83 eA-3
RESPONSE: ...
;
_vrf_PLAT977_p-1_final3
;
PROBLEM: Check Negative Difference Density on H01Z      .      -0.40 eA-3
RESPONSE: ...
;
# end Validation Reply Form
```

It is advisable to attempt to resolve as many as possible of the alerts in all categories. Often the minor alerts point to easily fixed oversights, errors and omissions in your CIF or refinement strategy, so attention to these fine details can be worthwhile. It is up to the individual to critically assess their own results and, if necessary, seek expert advice.

PLATON version of 15/01/2026; check.def file version of 02/01/2026

---

duplicate check

No duplication found

Datablock p-1_final3 - ellipsoid plot

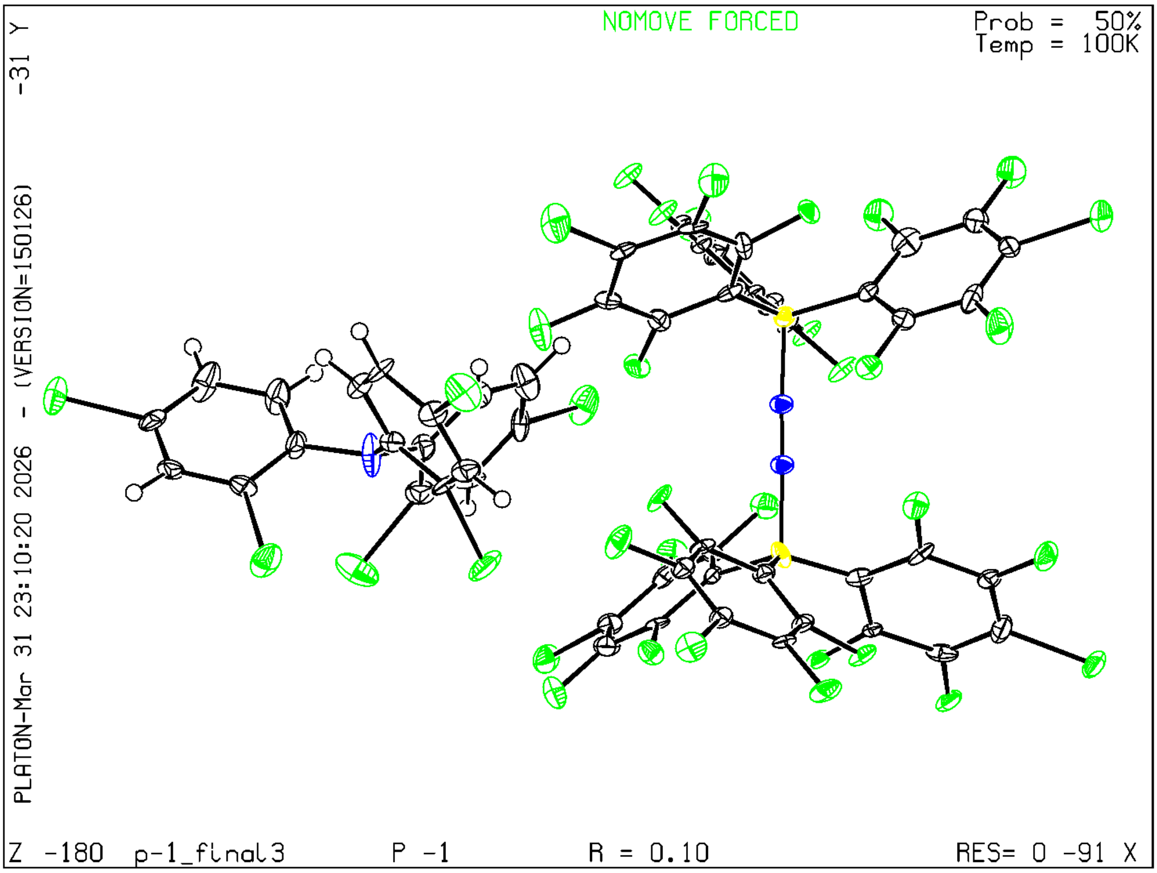

Supplement: Supplementary file 2 [file CHEM-32-e71310-s001.zip › chem71310-sup-0002-cif/magic green.pdf]
