# Supplementary material for: Expanding Cyanide‐Bridged Weakly Coordinating Anions Through the Brominated Silver Salt Ag[BCNBBr]
Source: Chemistry. 2026 Jun 27;32(38):e71310. doi: 10.1002/chem.71310 (PMC13660483; doi:10.1002/chem.71310)

checkCIF/PLATON report

Structure factors have been supplied for datablock(s) p21c_084_disorder2

THIS REPORT IS FOR GUIDANCE ONLY. IF USED AS PART OF A REVIEW PROCEDURE FOR PUBLICATION, IT SHOULD NOT REPLACE THE EXPERTISE OF AN EXPERIENCED CRYSTALLOGRAPHIC REFEREE.

No syntax errors found. CIF dictionary Interpreting this report

Datablock: p21c_084_disorder2

```
Bond precision:   C-C = 0.0082 A           Wavelength=0.71073

Cell:            a=14.9104(12)     b=17.3400(11)       c=22.6165(18)
                 alpha=90          beta=100.755(3)     gamma=90
Temperature:     150 K
```

	Calculated	Reported
Volume	5744.7(7)	5744.7(7)
Space group	P 21/c	P 1 21/c 1
Hall group	-P 2ybc	-P 2ybc
Moiety formula	C37.04 B2 Br6 F24 N1.04, C16 H34 N	B2 Br6 C37.039 F24 N1.039, C16 H34 N
Sum formula	C53.04 H34 B2 Br6 F24 N2.04	C53.04 H34 B2 Br6 F24 N2.04
Mr	1656.86	1656.94
Dx,g cm-3	1.916	1.916
Z	4	4
Mu (mm-1)	4.311	4.311
F000	3210.0	3210.0
F000’	3205.58	
h,k,lmax	17,20,26	17,20,26
Nref	10158	10144
Tmin,Tmax	0.656,0.650	0.615,0.745
Tmin’	0.643	

```
Correction method= # Reported T Limits: Tmin=0.615 Tmax=0.745
AbsCorr = MULTI-SCAN
```

Data completeness= 0.999 Theta(max)= 25.035

R(reflections)= 0.0582(8001) wR2(reflections)= 0.1265(10144)

S = 1.065 Npar= 940

The following ALERTS were generated. Each ALERT has the format
test-name_ALERT_alert-type_alert-level.
Click on the hyperlinks for more details of the test.

Alert level C

```
PLAT042_ALERT_1_C Calc. and Reported MoietyFormula Strings  Differ     Please Check
              Calc: C37.04 B2 Br6 F24 N1.04, C16 H34 N
              Rep.: B2 Br6 C37.039 F24 N1.039, C16 H34 N
PLAT077_ALERT_4_C Unit-Cell Contains Non-integer Number of Atoms .     Please Check
PLAT221_ALERT_2_C Solv./Anion  Resd 3  C   Ueq(max)/Ueq(min) Range        5.4 Ratio
PLAT223_ALERT_4_C Solv./Anion  Resd 3  H   Ueq(max)/Ueq(min) Range        6.7 Ratio
PLAT341_ALERT_3_C Low Bond Precision on  C-C Bonds ...............    0.00825 Ang.
PLAT767_ALERT_4_C INS Embedded LIST 6 Instruction Should be LIST 4     Please Check
PLAT906_ALERT_3_C Large K Value in the Analysis of Variance ......      5.758 Check
PLAT911_ALERT_3_C Missing FCF Refl Between Thmin & STh/L=    0.595         13 Report
                 1  1  0, -10 17  1,    0  0  2, -13 14  2, -15 11  3,  -2  0  6,
               -17  2 12, -15  8 14,  12  5 15, -16  0 16,  11  4 17,  -5 15 18,
                 8  8 19,
```

Alert level G

```
PLAT002_ALERT_2_G Number of Distance or Angle Restraints on AtSite         34 Note
PLAT003_ALERT_2_G Number of Uiso or U(i,j)  Restrained non-H-Atoms         27 Report
PLAT083_ALERT_2_G SHELXL Second Parameter in WGHT  Unusually Large      30.11 Why ?
PLAT168_ALERT_4_G The CIF-Embedded .res File Contains EXYZ Records          2 Report
PLAT171_ALERT_4_G The CIF-Embedded .res File Contains EADP Records          1 Report
PLAT176_ALERT_4_G The CIF-Embedded .res File Contains SADI Records         34 Report
PLAT186_ALERT_4_G The CIF-Embedded .res File Contains ISOR Records         12 Report
PLAT187_ALERT_4_G The CIF-Embedded .res File Contains RIGU Records          3 Report
PLAT191_ALERT_3_G A Non-default SADI Restraint Value has been used     0.0400 Report
PLAT191_ALERT_3_G A Non-default SADI Restraint Value has been used     0.0400 Report
PLAT191_ALERT_3_G A Non-default SADI Restraint Value has been used     0.0400 Report
PLAT191_ALERT_3_G A Non-default SADI Restraint Value has been used     0.0400 Report
PLAT191_ALERT_3_G A Non-default SADI Restraint Value has been used     0.0400 Report
PLAT191_ALERT_3_G A Non-default SADI Restraint Value has been used     0.0400 Report
PLAT191_ALERT_3_G A Non-default SADI Restraint Value has been used     0.0400 Report
PLAT191_ALERT_3_G A Non-default SADI Restraint Value has been used     0.0400 Report
PLAT191_ALERT_3_G A Non-default SADI Restraint Value has been used     0.0400 Report
PLAT191_ALERT_3_G A Non-default SADI Restraint Value has been used     0.0400 Report
PLAT191_ALERT_3_G A Non-default SADI Restraint Value has been used     0.0400 Report
PLAT191_ALERT_3_G A Non-default SADI Restraint Value has been used     0.0400 Report
PLAT191_ALERT_3_G A Non-default SADI Restraint Value has been used     0.0400 Report
PLAT191_ALERT_3_G A Non-default SADI Restraint Value has been used     0.0400 Report
PLAT191_ALERT_3_G A Non-default SADI Restraint Value has been used     0.0400 Report
PLAT191_ALERT_3_G A Non-default SADI Restraint Value has been used     0.0400 Report
PLAT191_ALERT_3_G A Non-default SADI Restraint Value has been used     0.0400 Report
PLAT191_ALERT_3_G A Non-default SADI Restraint Value has been used     0.0400 Report
```

```
PLAT230_ALERT_2_G Hirshfeld Test Diff for    N021     --C4       .         6.2 s.u.
PLAT299_ALERT_4_G Atom Site Occupancy Constrained at ............          0.5 Check
              N2      C1
PLAT300_ALERT_4_G Atom Site Occupancy of N021      Constrained at          0.6 Check
PLAT300_ALERT_4_G Atom Site Occupancy of C020      Constrained at          0.6 Check
PLAT300_ALERT_4_G Atom Site Occupancy of C025      Constrained at          0.6 Check
PLAT300_ALERT_4_G Atom Site Occupancy of C026      Constrained at          0.6 Check
PLAT300_ALERT_4_G Atom Site Occupancy of C4        Constrained at          0.6 Check
PLAT300_ALERT_4_G Atom Site Occupancy of C02B      Constrained at          0.6 Check
PLAT300_ALERT_4_G Atom Site Occupancy of C02D      Constrained at          0.6 Check
PLAT300_ALERT_4_G Atom Site Occupancy of C5A       Constrained at          0.6 Check
PLAT300_ALERT_4_G Atom Site Occupancy of C02E      Constrained at          0.6 Check
PLAT300_ALERT_4_G Atom Site Occupancy of C02F      Constrained at          0.6 Check
PLAT300_ALERT_4_G Atom Site Occupancy of C8        Constrained at          0.6 Check
PLAT300_ALERT_4_G Atom Site Occupancy of C01Z      Constrained at          0.6 Check
PLAT300_ALERT_4_G Atom Site Occupancy of C9        Constrained at          0.6 Check
PLAT300_ALERT_4_G Atom Site Occupancy of C0        Constrained at          0.6 Check
PLAT300_ALERT_4_G Atom Site Occupancy of C02I      Constrained at          0.6 Check
PLAT300_ALERT_4_G Atom Site Occupancy of C11       Constrained at          0.6 Check
PLAT300_ALERT_4_G Atom Site Occupancy of C55       Constrained at          0.6 Check
PLAT300_ALERT_4_G Atom Site Occupancy of H         Constrained at          0.6 Check
PLAT300_ALERT_4_G Atom Site Occupancy of H1        Constrained at          0.6 Check
PLAT300_ALERT_4_G Atom Site Occupancy of Ha        Constrained at          0.6 Check
PLAT300_ALERT_4_G Atom Site Occupancy of H2        Constrained at          0.6 Check
PLAT300_ALERT_4_G Atom Site Occupancy of Hb        Constrained at          0.6 Check
PLAT300_ALERT_4_G Atom Site Occupancy of H3        Constrained at          0.6 Check
PLAT300_ALERT_4_G Atom Site Occupancy of Hc        Constrained at          0.6 Check
PLAT300_ALERT_4_G Atom Site Occupancy of H4        Constrained at          0.6 Check
PLAT300_ALERT_4_G Atom Site Occupancy of Hd        Constrained at          0.6 Check
PLAT300_ALERT_4_G Atom Site Occupancy of H5        Constrained at          0.6 Check
PLAT300_ALERT_4_G Atom Site Occupancy of He        Constrained at          0.6 Check
PLAT300_ALERT_4_G Atom Site Occupancy of H6        Constrained at          0.6 Check
PLAT300_ALERT_4_G Atom Site Occupancy of Hf        Constrained at          0.6 Check
PLAT300_ALERT_4_G Atom Site Occupancy of H7        Constrained at          0.6 Check
PLAT300_ALERT_4_G Atom Site Occupancy of Hg        Constrained at          0.6 Check
PLAT300_ALERT_4_G Atom Site Occupancy of Hh        Constrained at          0.6 Check
PLAT300_ALERT_4_G Atom Site Occupancy of Hi        Constrained at          0.6 Check
PLAT300_ALERT_4_G Atom Site Occupancy of Hj        Constrained at          0.6 Check
PLAT300_ALERT_4_G Atom Site Occupancy of Hk        Constrained at          0.6 Check
PLAT300_ALERT_4_G Atom Site Occupancy of Hl        Constrained at          0.6 Check
PLAT300_ALERT_4_G Atom Site Occupancy of Hm        Constrained at          0.6 Check
PLAT300_ALERT_4_G Atom Site Occupancy of Hn        Constrained at          0.6 Check
PLAT300_ALERT_4_G Atom Site Occupancy of Ho        Constrained at          0.6 Check
PLAT300_ALERT_4_G Atom Site Occupancy of Hp        Constrained at          0.6 Check
PLAT300_ALERT_4_G Atom Site Occupancy of Hq        Constrained at          0.6 Check
PLAT300_ALERT_4_G Atom Site Occupancy of Hr        Constrained at          0.6 Check
PLAT300_ALERT_4_G Atom Site Occupancy of Hs        Constrained at          0.6 Check
PLAT300_ALERT_4_G Atom Site Occupancy of Ht        Constrained at          0.6 Check
PLAT300_ALERT_4_G Atom Site Occupancy of Hu        Constrained at          0.6 Check
PLAT300_ALERT_4_G Atom Site Occupancy of Hv        Constrained at          0.6 Check
PLAT300_ALERT_4_G Atom Site Occupancy of Hw        Constrained at          0.6 Check
PLAT300_ALERT_4_G Atom Site Occupancy of Hx        Constrained at          0.6 Check
PLAT300_ALERT_4_G Atom Site Occupancy of Hy        Constrained at          0.6 Check
PLAT300_ALERT_4_G Atom Site Occupancy of Hz        Constrained at          0.6 Check
PLAT300_ALERT_4_G Atom Site Occupancy of N0        Constrained at          0.4 Check
PLAT300_ALERT_4_G Atom Site Occupancy of C3        Constrained at          0.4 Check
PLAT300_ALERT_4_G Atom Site Occupancy of C4A       Constrained at          0.4 Check
```

```
PLAT300_ALERT_4_G Atom Site Occupancy of C5         Constrained at        0.4 Check
PLAT300_ALERT_4_G Atom Site Occupancy of C5B        Constrained at        0.4 Check
PLAT300_ALERT_4_G Atom Site Occupancy of C6         Constrained at        0.4 Check
PLAT300_ALERT_4_G Atom Site Occupancy of C7         Constrained at        0.4 Check
PLAT300_ALERT_4_G Atom Site Occupancy of C8A        Constrained at        0.4 Check
PLAT300_ALERT_4_G Atom Site Occupancy of C9A        Constrained at        0.4 Check
PLAT300_ALERT_4_G Atom Site Occupancy of C0A        Constrained at        0.4 Check
PLAT300_ALERT_4_G Atom Site Occupancy of C11A       Constrained at        0.4 Check
PLAT300_ALERT_4_G Atom Site Occupancy of C12        Constrained at        0.4 Check
PLAT300_ALERT_4_G Atom Site Occupancy of C02J       Constrained at        0.4 Check
PLAT300_ALERT_4_G Atom Site Occupancy of C02C       Constrained at        0.4 Check
PLAT300_ALERT_4_G Atom Site Occupancy of C02G       Constrained at        0.4 Check
PLAT300_ALERT_4_G Atom Site Occupancy of C15        Constrained at        0.4 Check
PLAT300_ALERT_4_G Atom Site Occupancy of C55A       Constrained at        0.4 Check
PLAT300_ALERT_4_G Atom Site Occupancy of H3A        Constrained at        0.4 Check
PLAT300_ALERT_4_G Atom Site Occupancy of H3B        Constrained at        0.4 Check
PLAT300_ALERT_4_G Atom Site Occupancy of H5A        Constrained at        0.4 Check
PLAT300_ALERT_4_G Atom Site Occupancy of H5B        Constrained at        0.4 Check
PLAT300_ALERT_4_G Atom Site Occupancy of H7A        Constrained at        0.4 Check
PLAT300_ALERT_4_G Atom Site Occupancy of H7B        Constrained at        0.4 Check
PLAT300_ALERT_4_G Atom Site Occupancy of H7C        Constrained at        0.4 Check
PLAT300_ALERT_4_G Atom Site Occupancy of H11A       Constrained at        0.4 Check
PLAT300_ALERT_4_G Atom Site Occupancy of H11B       Constrained at        0.4 Check
PLAT300_ALERT_4_G Atom Site Occupancy of H11C       Constrained at        0.4 Check
PLAT300_ALERT_4_G Atom Site Occupancy of H12A       Constrained at        0.4 Check
PLAT300_ALERT_4_G Atom Site Occupancy of H12B       Constrained at        0.4 Check
PLAT300_ALERT_4_G Atom Site Occupancy of H15A       Constrained at        0.4 Check
PLAT300_ALERT_4_G Atom Site Occupancy of H15B       Constrained at        0.4 Check
PLAT300_ALERT_4_G Atom Site Occupancy of H15C       Constrained at        0.4 Check
PLAT300_ALERT_4_G Atom Site Occupancy of H9AA       Constrained at        0.4 Check
PLAT300_ALERT_4_G Atom Site Occupancy of H9AB       Constrained at        0.4 Check
PLAT300_ALERT_4_G Atom Site Occupancy of H0AA       Constrained at        0.4 Check
PLAT300_ALERT_4_G Atom Site Occupancy of H0AB       Constrained at        0.4 Check
PLAT300_ALERT_4_G Atom Site Occupancy of H5BA       Constrained at        0.4 Check
PLAT300_ALERT_4_G Atom Site Occupancy of H5BB       Constrained at        0.4 Check
PLAT300_ALERT_4_G Atom Site Occupancy of H4AA       Constrained at        0.4 Check
PLAT300_ALERT_4_G Atom Site Occupancy of H4AB       Constrained at        0.4 Check
PLAT300_ALERT_4_G Atom Site Occupancy of H8AA       Constrained at        0.4 Check
PLAT300_ALERT_4_G Atom Site Occupancy of H8AB       Constrained at        0.4 Check
PLAT300_ALERT_4_G Atom Site Occupancy of H02A       Constrained at        0.4 Check
PLAT300_ALERT_4_G Atom Site Occupancy of H02B       Constrained at        0.4 Check
PLAT300_ALERT_4_G Atom Site Occupancy of H02C       Constrained at        0.4 Check
PLAT300_ALERT_4_G Atom Site Occupancy of H02D       Constrained at        0.4 Check
PLAT300_ALERT_4_G Atom Site Occupancy of H02E       Constrained at        0.4 Check
PLAT300_ALERT_4_G Atom Site Occupancy of H02F       Constrained at        0.4 Check
PLAT300_ALERT_4_G Atom Site Occupancy of H55A       Constrained at        0.4 Check
PLAT300_ALERT_4_G Atom Site Occupancy of H55B       Constrained at        0.4 Check
PLAT300_ALERT_4_G Atom Site Occupancy of H55C       Constrained at        0.4 Check
PLAT301_ALERT_3_G Main Residue  Disorder .............(Resd    1)          3% Note
PLAT302_ALERT_4_G Anion/Solvent/Minor-Residue Disorder (Resd    2)        100% Note
PLAT302_ALERT_4_G Anion/Solvent/Minor-Residue Disorder (Resd    3)        100% Note
PLAT304_ALERT_4_G Non-Integer Number of Atoms in ..... (Resd    1)      70.08 Check
PLAT304_ALERT_4_G Non-Integer Number of Atoms in ..... (Resd    2)      30.60 Check
PLAT304_ALERT_4_G Non-Integer Number of Atoms in ..... (Resd    3)      20.40 Check
PLAT434_ALERT_2_G Short Inter HL..HL Contact Br11     ..F222     .       3.05 Ang.
                                             x,1/2-y,-1/2+z  =      4_565 Check
PLAT434_ALERT_2_G Short Inter HL..HL Contact Br12     ..F136     .       3.20 Ang.
```

```
                                                 1-x,1-y,1-z  =      3_666 Check
PLAT434_ALERT_2_G Short Inter HL..HL Contact Br22     ..F212     .       3.20 Ang.
                                                 2-x,1-y,1-z  =      3_766 Check
PLAT434_ALERT_2_G Short Inter HL..HL Contact Br23     ..F122     .       3.13 Ang.
                                               x,1/2-y,1/2+z  =      4_566 Check
PLAT434_ALERT_2_G Short Inter HL..HL Contact F123     ..F225     .       2.71 Ang.
                                                 2-x,1-y,1-z  =      3_766 Check
PLAT434_ALERT_2_G Short Inter HL..HL Contact F133     ..F235     .       2.76 Ang.
                                                  1-x,-y,1-z  =      3_656 Check
PLAT720_ALERT_4_G Number of Unusual/Non-Standard Labels .........          58 Note
              C020    Ha      Hb      N021    C025    Hc      C026    Hd
              C02B    He      Hf      C02D    Hg      Hh      C02E    Hi
              Hj      Hk      C02F    Hl      Hm      Hn      Ho      Hp
              C01Z    Hq      Hr      C0      Hs      Ht      Hu      Hv
              Hw      Hx      C02I    Hy      Hz      H9AA    H9AB    C0A
              H0AA    H0AB    H5BA    H5BB    N0      H4AA    H4AB    H8AA
              H8AB    C02J    H02A    H02B    C02C    H02C    H02D    C02G
              H02E    H02F
PLAT860_ALERT_3_G Number of Least-Squares Restraints ............         256 Note
PLAT909_ALERT_3_G Percentage of I>2sig(I) Data at Theta(Max) Still         53% Note
PLAT910_ALERT_3_G Missing FCF Reflection(s) Below Theta(Min)[Deg]=        1.49 Note
                1  0  0,
PLAT933_ALERT_2_G Number of HKL-OMIT Records in Embedded .res File           2 Note
                1  0  0,   1  1  0,
PLAT969_ALERT_5_G The 'Henn et al.' R-Factor-gap value ..........       2.217 Note
              Predicted wR2: Based on SigI**2  5.71 or SHELX Weight 11.88
PLAT978_ALERT_2_G Number C-C Bonds with Positive Residual Density.           2 Info
PLAT994_ALERT_1_G SHELXL .ins Contains no or MERG 0 Instruction ..           ! Note
```

```
  0 ALERT level A = Most likely a serious problem - resolve or explain
  0 ALERT level B = A potentially serious problem, consider carefully
  8 ALERT level C = Check. Ensure it is not caused by an omission or oversight
150 ALERT level G = General information/check it is not something unexpected

  2 ALERT type 1 CIF construction/syntax error, inconsistent or missing data
 13 ALERT type 2 Indicator that the structure model may be wrong or deficient
 25 ALERT type 3 Indicator that the structure quality may be low
117 ALERT type 4 Improvement, methodology, query or suggestion
  1 ALERT type 5 Informative message, check
```

Validation response form

Please find below a validation response form (VRF) that can be filled in and pasted into your CIF.

```
# start Validation Reply Form
_vrf_PLAT042_p21c_084_disorder2
;
PROBLEM: Calc. and Reported MoietyFormula Strings  Differ     Please Check
RESPONSE: ...
;
_vrf_PLAT077_p21c_084_disorder2
;
PROBLEM: Unit-Cell Contains Non-integer Number of Atoms .     Please Check
RESPONSE: ...
;
```

```
_vrf_PLAT221_p21c_084_disorder2
;
PROBLEM: Solv./Anion  Resd 3  C   Ueq(max)/Ueq(min) Range        5.4 Ratio
RESPONSE: ...
;
_vrf_PLAT223_p21c_084_disorder2
;
PROBLEM: Solv./Anion  Resd 3  H   Ueq(max)/Ueq(min) Range        6.7 Ratio
RESPONSE: ...
;
_vrf_PLAT341_p21c_084_disorder2
;
PROBLEM: Low Bond Precision on  C-C Bonds ...............    0.00825 Ang.
RESPONSE: ...
;
_vrf_PLAT767_p21c_084_disorder2
;
PROBLEM: INS Embedded LIST 6 Instruction Should be LIST 4     Please Check
RESPONSE: ...
;
_vrf_PLAT906_p21c_084_disorder2
;
PROBLEM: Large K Value in the Analysis of Variance ......      5.758 Check
RESPONSE: ...
;
_vrf_PLAT911_p21c_084_disorder2
;
PROBLEM: Missing FCF Refl Between Thmin & STh/L=    0.595         13 Report
RESPONSE: ...
;
# end Validation Reply Form
```

It is advisable to attempt to resolve as many as possible of the alerts in all categories. Often the minor alerts point to easily fixed oversights, errors and omissions in your CIF or refinement strategy, so attention to these fine details can be worthwhile. It is up to the individual to critically assess their own results and, if necessary, seek expert advice.

PLATON version of 15/01/2026; check.def file version of 02/01/2026

---

duplicate check

No duplication found

Datablock p21c_084_disorder2 - ellipsoid plot

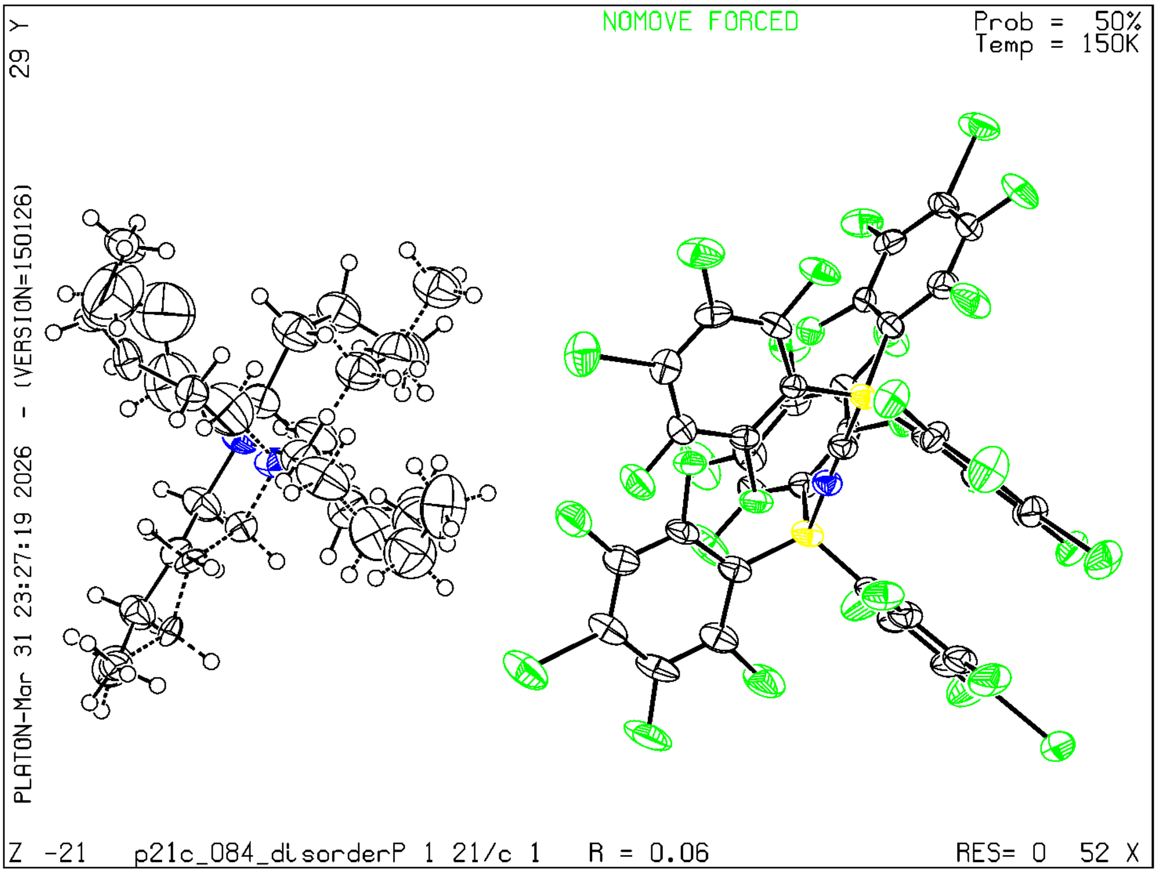

Supplement: Supplementary file 2 [file CHEM-32-e71310-s001.zip › chem71310-sup-0002-cif/tba.pdf]
