# Supplementary material for: Expanding Cyanide‐Bridged Weakly Coordinating Anions Through the Brominated Silver Salt Ag[BCNBBr]
Source: Chemistry. 2026 Jun 27;32(38):e71310. doi: 10.1002/chem.71310 (PMC13660483; doi:10.1002/chem.71310)

checkCIF/PLATON report

Structure factors have been supplied for datablock(s) p-1

THIS REPORT IS FOR GUIDANCE ONLY. IF USED AS PART OF A REVIEW PROCEDURE FOR PUBLICATION, IT SHOULD NOT REPLACE THE EXPERTISE OF AN EXPERIENCED CRYSTALLOGRAPHIC REFEREE.

No syntax errors found. CIF dictionary Interpreting this report

Datablock: p-1

Bond precision: C-C = 0.0057 A Wavelength=0.71073

Cell:	a=12.1574(4)	b=14.4362(5)	c=15.5810(5)
	alpha=70.303(1)	beta=73.666(1)	gamma=83.038(1)
Temperature:	100 K		

	Calculated	Reported
Volume	2469.60(14)	2469.60(14)
Space group	P -1	P-1
Hall group	-P 1	-P 1
Moiety formula	C37 B2 Br6 F24 N, C12 H8 S2	B2 Br6 C37 F24 N, C12 H8 S2
Sum formula	C49 H8 B2 Br6 F24 N S2	C49 H8 B2 Br6 F24 N S2
Mr	1631.70	1631.76
Dx,g cm-3	2.194	2.194
Z	2	2
Mu (mm-1)	5.094	5.094
F000	1554.0	1554.0
F000'	1552.27	
h,k,lmax	16,19,21	16,19,21
Nref	12978	12963
Tmin,Tmax	0.146,0.361	0.192,0.414
Tmin'	0.007	

Correction method= # Reported T Limits: Tmin=0.192 Tmax=0.414
AbsCorr = NUMERICAL

Data completeness= 0.999 Theta(max)= 28.860

R(reflections)= 0.0436(9680) wR2(reflections)= 0.1080(12963)

S = 1.062 Npar= 751

The following ALERTS were generated. Each ALERT has the format
test-name_ALERT_alert-type_alert-level.
Click on the hyperlinks for more details of the test.

Alert level C

PLAT042_ALERT_1_C Calc. and Reported MoietyFormula Strings Differ Please Check
Calc: C37 B2 Br6 F24 N, C12 H8 S2
Rep.: B2 Br6 C37 F24 N, C12 H8 S2
PLAT911_ALERT_3_C Missing FCF Refl Between Thmin & STh/L= 0.600 2 Report
1 0 0, 5 0 3,

Alert level G

PLAT063_ALERT_4_G Crystal Size Possibly too Large for Beam Size .. 0.94 mm
PLAT154_ALERT_1_G The s.u.'s on the Cell Angles are Equal ..(Note) 0.001 Degree
PLAT168_ALERT_4_G The CIF-Embedded .res File Contains EXYZ Records 2 Report
PLAT171_ALERT_4_G The CIF-Embedded .res File Contains EADP Records 1 Report
PLAT299_ALERT_4_G Atom Site Occupancy Constrained at .... 0.5 Check
N1 N2 C1 C2
PLAT301_ALERT_3_G Main Residue Disorder ....(Resd 1) 3% Note
PLAT432_ALERT_2_G Short Inter X...Y Contact F213 ..C122 . 2.87 Ang.
2-x,1-y,1-z = 2_766 Check
PLAT434_ALERT_2_G Short Inter HL..HL Contact Br12 ..F136 . 3.01 Ang.
2-x,2-y,-z = 2_775 Check
PLAT434_ALERT_2_G Short Inter HL..HL Contact Br22 ..F126 . 3.16 Ang.
2-x,2-y,1-z = 2_776 Check
PLAT434_ALERT_2_G Short Inter HL..HL Contact F125 ..F225 . 2.64 Ang.
2-x,2-y,1-z = 2_776 Check
PLAT434_ALERT_2_G Short Inter HL..HL Contact F133 ..F223 . 2.81 Ang.
-1+x,y,z = 1_455 Check
PLAT434_ALERT_2_G Short Inter HL..HL Contact F135 ..F135 . 2.79 Ang.
1-x,2-y,-z = 2_675 Check
PLAT434_ALERT_2_G Short Inter HL..HL Contact F235 ..F236 . 2.79 Ang.
1-x,2-y,1-z = 2_676 Check
PLAT720_ALERT_4_G Number of Unusual/Non-Standard Labels .... 21 Note
S007 S008 C01Z C020 C021 Ha C022 Hb
C023 Hc C024 Hd C025 C026 C027 C029
He C02A Hf C02B Hg
PLAT910_ALERT_3_G Missing FCF Reflection(s) Below Theta(Min)[Deg]= 1.71 Note
0 1 0, 0 0 1,
PLAT912_ALERT_4_G Missing # of FCF Reflections Above STh/L= 0.600 11 Note
PLAT941_ALERT_3_G Average HKL Measurement Multiplicity .... 4.1 Low
PLAT969_ALERT_5_G The 'Henn et al.' R-Factor-gap value .... 1.839 Note
Predicted wR2: Based on SigI**2 5.87 or SHELX Weight 10.17
PLAT978_ALERT_2_G Number C-C Bonds with Positive Residual Density. 0 Info
PLAT994_ALERT_1_G SHELXL .ins Contains no or MERG 0 Instruction .. ! Note

```
   0 ALERT level A = Most likely a serious problem - resolve or explain
   0 ALERT level B = A potentially serious problem, consider carefully
   2 ALERT level C = Check. Ensure it is not caused by an omission or oversight
  20 ALERT level G = General information/check it is not something unexpected

   3 ALERT type 1 CIF construction/syntax error, inconsistent or missing data
   8 ALERT type 2 Indicator that the structure model may be wrong or deficient
   4 ALERT type 3 Indicator that the structure quality may be low
   6 ALERT type 4 Improvement, methodology, query or suggestion
   1 ALERT type 5 Informative message, check
```

Validation response form

Please find below a validation response form (VRF) that can be filled in and pasted into your CIF.

```
# start Validation Reply Form
_vrf_PLAT042_p-1
;
PROBLEM: Calc. and Reported MoietyFormula Strings  Differ     Please Check
RESPONSE: ...
;
_vrf_PLAT911_p-1
;
PROBLEM: Missing FCF Refl Between Thmin & STh/L=    0.600          2 Report
RESPONSE: ...
;
# end Validation Reply Form
```

It is advisable to attempt to resolve as many as possible of the alerts in all categories. Often the minor alerts point to easily fixed oversights, errors and omissions in your CIF or refinement strategy, so attention to these fine details can be worthwhile. It is up to the individual to critically assess their own results and, if necessary, seek expert advice.

PLATON version of 15/01/2026; check.def file version of 02/01/2026

duplicate check

No duplication found

Datablock p-1 - ellipsoid plot

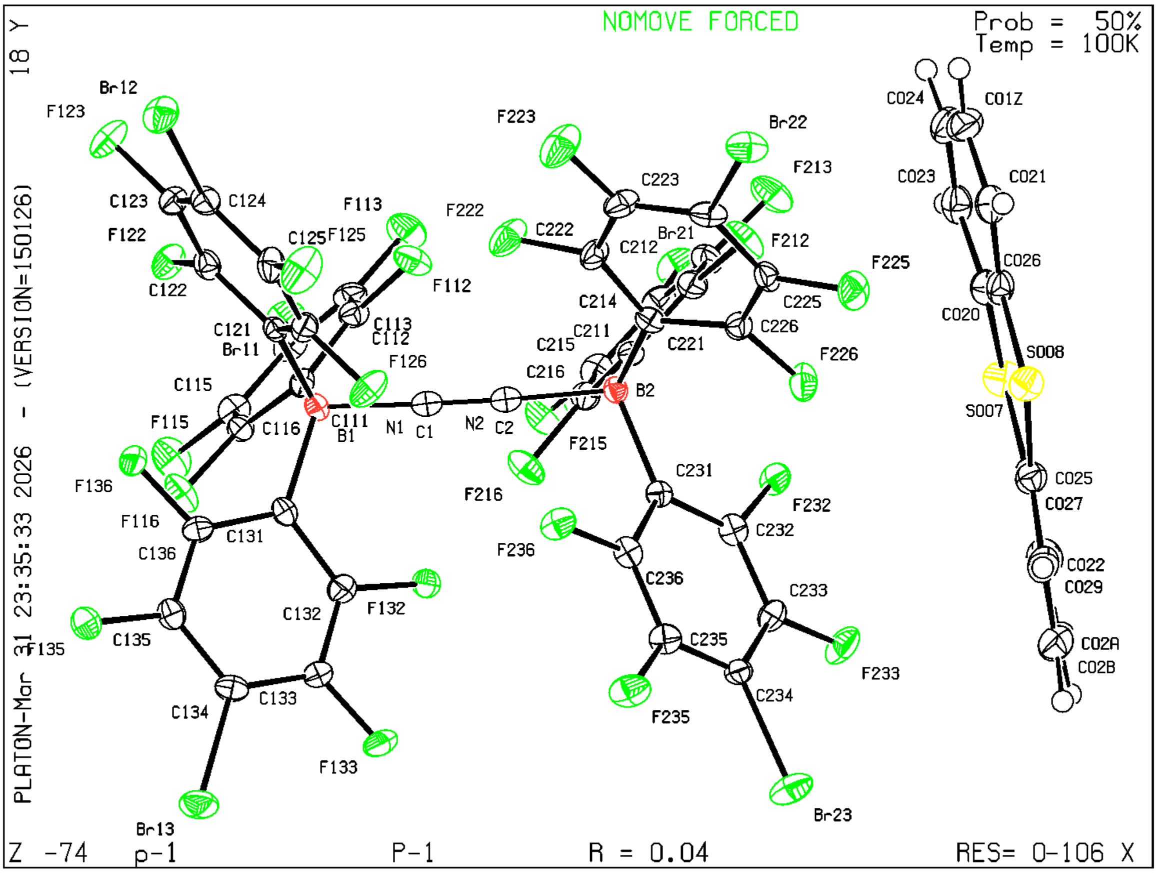

Supplement: Supplementary file 2 [file CHEM-32-e71310-s001.zip › chem71310-sup-0002-cif/thianthrenium.pdf]
