# Supplementary material for: Expanding Cyanide‐Bridged Weakly Coordinating Anions Through the Brominated Silver Salt Ag[BCNBBr]
Source: Chemistry. 2026 Jun 27;32(38):e71310. doi: 10.1002/chem.71310 (PMC13660483; doi:10.1002/chem.71310)

# checkCIF/PLATON report

Structure factors have been supplied for datablock(s) p-1

THIS REPORT IS FOR GUIDANCE ONLY. IF USED AS PART OF A REVIEW PROCEDURE FOR PUBLICATION, IT SHOULD NOT REPLACE THE EXPERTISE OF AN EXPERIENCED CRYSTALLOGRAPHIC REFEREE.

No syntax errors found. CIF dictionary Interpreting this report

## Datablock: p-1

---

```
Bond precision:   C-C = 0.0041 A          Wavelength=0.71073

Cell:           a=13.5680(4)       b=14.5595(5)       c=15.3447(5)
                alpha=98.163(1)    beta=112.800(1)    gamma=94.156(1)
Temperature:    100 K

                Calculated                  Reported
Volume          2738.95(15)                 2738.95(15)
Space group     P -1                        P -1
Hall group      -P 1                        -P 1
Moiety formula  C37 B2 Br6 F24 N, C19 H15   B2 Br6 C37 F24 N, C19 H15
Sum formula     C56 H15 B2 Br6 F24 N        C56 H15 B2 Br6 F24 N
Mr              1658.71                     1658.77
Dx,g cm-3       2.011                       2.011
Z               2                           2
Mu (mm-1)       4.522                       4.522
F000            1588.0                      1588.0
F000'           1585.79
h,k,lmax        19,20,21                    19,20,21
Nref            16031                       15997
Tmin,Tmax       0.100,0.108                 0.428,0.746
Tmin'           0.075

Correction method= # Reported T Limits: Tmin=0.428 Tmax=0.746
AbsCorr = MULTI-SCAN
```

```
Data completeness= 0.998            Theta(max)= 30.044

                                                        wR2(reflections)=
R(reflections)= 0.0412( 12027)                          0.0993( 15997)

S = 1.056                  Npar= 796
```

---

The following ALERTS were generated. Each ALERT has the format
**test-name_ALERT_alert-type_alert-level**.
Click on the hyperlinks for more details of the test.

---

### Alert level C

```
PLAT042_ALERT_1_C Calc. and Reported MoietyFormula Strings  Differ     Please Check
              Calc: C37 B2 Br6 F24 N, C19 H15
              Rep.: B2 Br6 C37 F24 N, C19 H15
PLAT910_ALERT_3_C Missing FCF Reflection(s) Below Theta(Min)[Deg]=       2.20 Note
                1  0  0,  -1  1  0,   0  1  0,   0 -1  1,  -1  0  1,   0  0  1,
PLAT911_ALERT_3_C Missing FCF Refl Between Thmin & STh/L=    0.600          2 Report
                1 -4  3,   1 -3  3,
```

---

### Alert level G

```
PLAT154_ALERT_1_G The s.u.'s on the Cell Angles are Equal ..(Note)      0.001 Degree
PLAT168_ALERT_4_G The CIF-Embedded .res File Contains EXYZ Records          2 Report
PLAT171_ALERT_4_G The CIF-Embedded .res File Contains EADP Records          1 Report
PLAT299_ALERT_4_G Atom Site Occupancy Constrained at ............         0.5 Check
              N1      N2      C1      C2
PLAT301_ALERT_3_G Main Residue  Disorder .............(Resd    1)          3% Note
PLAT432_ALERT_2_G Short Inter X...Y Contact  Br06     ..C01P     .       3.30 Ang.
                                              1-x,1-y,2-z  =      2_667 Check
PLAT434_ALERT_2_G Short Inter HL..HL Contact Br02     ..F009     .       3.14 Ang.
                                              1-x,2-y,1-z  =      2_676 Check
PLAT434_ALERT_2_G Short Inter HL..HL Contact Br03     ..F008     .       3.07 Ang.
                                              1-x,2-y,2-z  =      2_677 Check
PLAT434_ALERT_2_G Short Inter HL..HL Contact Br03     ..Br06     .       3.56 Ang.
                                              1-x,1-y,2-z  =      2_667 Check
PLAT434_ALERT_2_G Short Inter HL..HL Contact Br04     ..F00L     .       3.19 Ang.
                                                 -1+x,y,z  =      1_455 Check
PLAT434_ALERT_2_G Short Inter HL..HL Contact F00A     ..F00U     .       2.81 Ang.
                                              1-x,1-y,1-z  =      2_666 Check
PLAT434_ALERT_2_G Short Inter HL..HL Contact F00E     ..F00P     .       2.82 Ang.
                                               -x,1-y,1-z  =      2_566 Check
PLAT434_ALERT_2_G Short Inter HL..HL Contact F00Q     ..F00T     .       2.74 Ang.
                                              1-x,2-y,2-z  =      2_677 Check
PLAT720_ALERT_4_G Number of Unusual/Non-Standard Labels .........        102 Note
              Br01    Br02    Br03    Br04    Br05    Br06    F007    F008
              F009    F00A    F00B    F00C    F00D    F00E    F00F    F00G
              F00H    F00I    F00J    F00K    F00L    F00M    F00N    F00O
              F00P    F00Q    F00R    F00S    F00T    F00U    C00X    C00Y
              C00Z    C010    C011    C012    C013    C014    C015    C016
              C017    C018    C019    C01A    C01B    C01C    C01D    C01E
              C01F    C01G    C01H    C01I    C01J    C01K    C01L    C01M
              C01N    C01O    C01P    C01Q    C01R    C01S    C01T    H01T
              C01U    C01V    H01V    C01W    H01W    C01X    C01Y    C01Z
```

```
              C020    H020    C021    C022    H022    C023    H023    C024
              C025    H025    C026    C027    C028    H028    C029    H029
              C02A    H02A    C02B    H02B    C02C    H02C    C02D    H02D
              B02E    C02F    H02F    C02G    H02G    B02H
PLAT912_ALERT_4_G Missing # of FCF Reflections Above STh/L=  0.600         28 Note
PLAT913_ALERT_3_G Missing # of Very Strong Reflections in FCF ....          2 Note
                1 -4  3,   1 -3  3,
PLAT933_ALERT_2_G Number of HKL-OMIT Records in Embedded .res File          1 Note
                0 -1  1,
PLAT941_ALERT_3_G Average HKL Measurement Multiplicity ..........         4.2 Low
PLAT969_ALERT_5_G The 'Henn et al.' R-Factor-gap value ..........       1.688 Note
              Predicted wR2: Based on SigI**2  5.89 or SHELX Weight  9.41
PLAT978_ALERT_2_G Number C-C Bonds with Positive Residual Density.          1 Info
PLAT994_ALERT_1_G SHELXL .ins Contains no or MERG 0 Instruction ..          ! Note
```

---

```
   0 ALERT level A = Most likely a serious problem - resolve or explain
   0 ALERT level B = A potentially serious problem, consider carefully
   3 ALERT level C = Check. Ensure it is not caused by an omission or oversight
  21 ALERT level G = General information/check it is not something unexpected

   3 ALERT type 1 CIF construction/syntax error, inconsistent or missing data
  10 ALERT type 2 Indicator that the structure model may be wrong or deficient
   5 ALERT type 3 Indicator that the structure quality may be low
   5 ALERT type 4 Improvement, methodology, query or suggestion
   1 ALERT type 5 Informative message, check
```

---

## Validation response form

Please find below a validation response form (VRF) that can be filled in and pasted into your CIF.

```
# start Validation Reply Form
_vrf_PLAT042_p-1
;
PROBLEM: Calc. and Reported MoietyFormula Strings  Differ     Please Check
RESPONSE: ...
;
_vrf_PLAT910_p-1
;
PROBLEM: Missing FCF Reflection(s) Below Theta(Min)[Deg]=       2.20 Note
RESPONSE: ...
;
_vrf_PLAT911_p-1
;
PROBLEM: Missing FCF Refl Between Thmin & STh/L=    0.600          2 Report
RESPONSE: ...
;
# end Validation Reply Form
```

---

It is advisable to attempt to resolve as many as possible of the alerts in all categories. Often the minor alerts point to easily fixed oversights, errors and omissions in your CIF or refinement strategy, so attention to these fine details can be worthwhile. It is up to the individual to critically assess their own results and, if necessary, seek expert advice.

**PLATON version of 15/01/2026; check.def file version of 02/01/2026**

# duplicate check

## No duplication found

**Datablock p-1** - ellipsoid plot

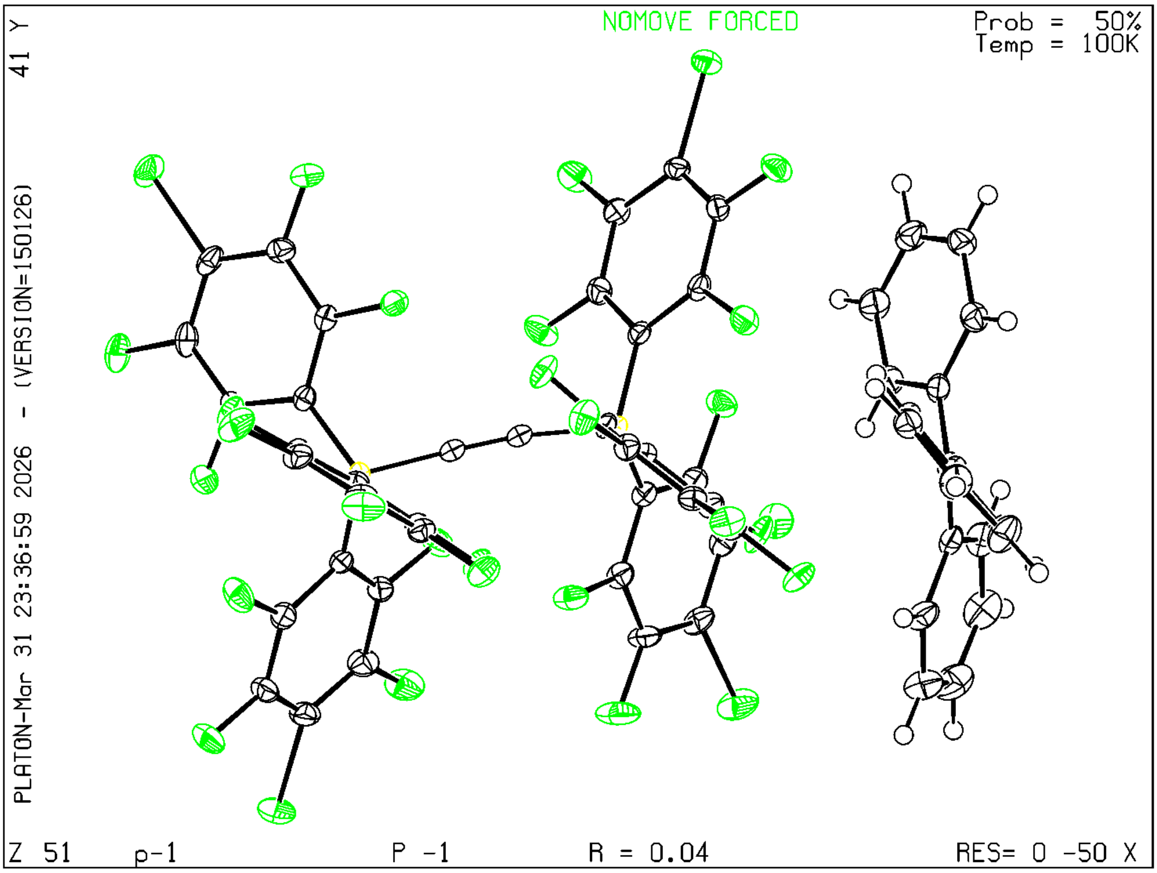

Supplement: Supplementary file 2 [file CHEM-32-e71310-s001.zip › chem71310-sup-0002-cif/tritylium.pdf]
